# A Global Proteomic Approach Sheds New Light on Potential Iron-Sulfur Client Proteins of the Chloroplastic Maturation Factor NFU3

**DOI:** 10.3390/ijms21218121

**Published:** 2020-10-30

**Authors:** Nathalie Berger, Florence Vignols, Brigitte Touraine, Maël Taupin-Broggini, Valérie Rofidal, Vincent Demolombe, Véronique Santoni, Nicolas Rouhier, Frédéric Gaymard, Christian Dubos

**Affiliations:** 1 BPMP, Université de Montpellier, CNRS, INRAE, SupAgro, Montpellier, France; nathalie.berger@inrae.fr (N.B.); florence.vignols@supagro.fr (F.V.); brigitte.touraine@inrae.fr (B.T.); mael.taupin-broggini@etu.umontpellier.fr (M.T.-B.); valerie.rofidal@inrae.fr (V.R.); vincent.demolombe--liozu@inrae.fr (V.D.); veronique.santoni@inrae.fr (V.S.); frederic.gaymard@inrae.fr (F.G.); 2 Université de Lorraine, INRAE, IAM, F-54000 Nancy, France; nicolas.rouhier@univ-lorraine.fr

**Keywords:** chloroplast, quantitative proteomic, iron-sulfur cluster, Arabidopsis, maturation factor, NFU1, NFU2, NFU3

## Abstract

Iron-sulfur (Fe-S) proteins play critical functions in plants. Most Fe-S proteins are synthetized in the cytosol as apo-proteins and the subsequent Fe-S cluster incorporation relies on specific protein assembly machineries. They are notably formed by a scaffold complex, which serves for the de novo Fe-S cluster synthesis, and by transfer proteins that insure cluster delivery to apo-targets. However, scarce information is available about the maturation pathways of most plastidial Fe-S proteins and their specificities towards transfer proteins of the associated SUF machinery. To gain more insights into these steps, the expression and protein localization of the NFU1, NFU2, and NFU3 transfer proteins were analyzed in various *Arabidopsis thaliana* organs and tissues showing quite similar expression patterns. In addition, quantitative proteomic analysis of an *nfu3* loss-of-function mutant allowed to propose novel potential client proteins for NFU3 and to show that the protein accumulation profiles and thus metabolic adjustments differ substantially from those established in the *nfu2* mutant. By clarifying the respective roles of the three plastidial NFU paralogs, these data allow better delineating the maturation process of plastidial Fe-S proteins.

## 1. Introduction

Iron is a transition metal essential for both prokaryotic and eukaryotic organisms. Iron is part of cofactors associated to proteins involved in numerous physiological processes such as respiration or photosynthesis [1]. As a cofactor moiety, iron is found under diverse forms (i.e., as single atom or in complex with other elements) among which are hemes and iron-sulfur (Fe-S) clusters [2]. The predominant forms of Fe-S clusters are the [4Fe-4S] and [2Fe-2S] types coordinated by four cysteine residues whereas [3Fe-4S] and Rieske-type [2Fe-2S] clusters are less frequently encountered [3,4]. In plants, in addition to photosynthesis and respiration, Fe-S clusters are for example necessary for the assimilation of sulfur and nitrogen, the metabolism of chlorophylls or the biosynthesis of branched-chain amino acids [1].

Most of the plant Fe-S proteins are synthetized in the cytosol as apo-proteins (i.e., deprived of Fe-S cluster). Apo-proteins are then targeted to their final destination/cell compartments (i.e., nucleus, mitochondria, and plastids) for their maturation, including in particular the acquisition of their Fe-S cluster. In *Arabidopsis thaliana*, there are three machineries for Fe-S protein maturation: the CIA (cytosolic iron-sulfur protein assembly), the ISC (iron-sulfur cluster) and the SUF (sulfur mobilization) machineries that are respectively localized in the cytosol, the mitochondria and the plastids. The de novo Fe-S cluster synthesis primarily necessitates the activity of cysteine desulfurases that provide sulfur atoms (together with its associated regulatory proteins), of electron donors to reduce the sulfane sulfur species into sulfide, and of iron donors that remain, for the three machineries, to be identified [2]. If the principal steps leading to the synthesis of Fe-S clusters are roughly conserved among the three machineries, the protein composition of these multi-protein complexes differs as a function of the machinery type [2]. After its assembly, the preformed Fe-S cluster is transferred to the apo-targets *via* late-acting maturation factors including so-called Fe-S cluster carrier/transfer proteins [2].

In Arabidopsis, 10 proteins from the SUF machinery have been identified as potential Fe-S cluster carrier proteins. It includes BOLA1, BOLA4, GRXS14, GRXS16, IBA57.2, and SUFA1 as well as HCF101, NFU1, NFU2, and NFU3 [3,5]. Some of these potential Fe-S cluster transfer proteins were characterized at the biochemical level and their capacity to transfer Fe-S cluster, alone or in complex, examined. For instance, GRXS14, GRXS16, and SUFA1 are reported as [2Fe-2S] cluster-incorporating proteins able to transfer their cluster to an apo-ferredoxin [6,7]. A [2Fe-2S] cluster transfer from GRXS14 to SUFA1 was also reported, suggesting an in vivo sequential Fe-S cluster shuttling similar to the one described for mitochondrial orthologs [8,9,10]. NFU1 also interacts with SUFA1, although it is a [4Fe-4S] cluster-containing protein, suggesting that a conversion may be required if an Fe-S cluster exchange occurs [11]. The transfer of [4Fe-4S] cluster from NFU1 to ISPG (1-HYDROXY-2-METHYL-2-(E)-BUTENYL 4-DIPHOSPHATE SYNTHASE) and THIC (4-AMINO-5-HYDROXYMETHYL-2-METHYLPYRIMIDINE PHOSPHATE SYNTHASE), two enzymes respectively involved in isoprenoid and thiamine biosynthesis, was also reported [11]. In addition, the transfer of a [2Fe-2S] cluster from NFU2 to GRXS16 and DHAD (DIHYDROXYACID DEHYDRATASE involved in the synthesis of branched-chain amino acids) and of a [4Fe-4S] cluster from NFU2 to APR1 (ADENOSINE 5′-PHOSPHOSULFATE REDUCTASE 1 involved in sulfate assimilation) was demonstrated [12,13]. Interestingly, among chloroplastic NFU paralogs, NFU2 is to date the only one for which the capacity to bind either a [2Fe-2S] cluster or a [4Fe-4S] cluster has been directly and experimentally determined [12]. The absence of loss-of-function mutants in Arabidopsis for *BOLA1*, *BOLA4* or *IBA57.2* genes or the lack of obvious phenotypes for *grxs14*, *grsx16, sufa1,* and *nfu1* loss-of-function mutants prevented, so far, determining the precise functions of the corresponding proteins *in planta* [14,15,16]. In contrast, Arabidopsis *hcf101*, *nfu2,* and *nfu3* single mutants display strong phenotypes. For instance, *hcf101* mutant is seedling-lethal in the absence of sucrose [17,18]. However, the rescue of *hcf101* growth defects by the exogenous supply of sucrose is only partial. The growth of *nfu2* mutant is also severely impaired. *nfu2* mutants display pale green leaves together with a short root phenotype, which is related to defects in the biosynthesis of branched-chain amino acids [15]. A pale green leaf phenotype with severe growth defects was also observed for *nfu3* while a double *nfu2 nfu3* mutant is lethal [15,19,20] suggesting that they act, at least in part, in a redundant manner. Accordingly, the shoot phenotypes of both *nfu2* and *nfu3* mutants were associated with a defect in photosystem I (PSI) assembly, attributed to defaults in the maturation of PsaA, PsaB, and PsaC (PHOTOSYSTEM I SUBUNIT A, B, and C), three [4Fe-4S] cluster-binding proteins [15,19]. An additional common client protein could be the [3Fe-4S] cluster-containing FD-GOGAT GLU2 (GLUTAMATE SYNTHASE 2) that was found to interact with NFU2 [21] and the abundance of which, observed by western blot, is decreased in the *nfu3* mutant [19]. 

However, among the 53 plastidial proteins for which there is evidence or prediction that they contain Fe-S clusters [2,5], the combined use of in vitro and in vivo approaches allowed identifying 24 proteins as potential NFU2 targets whereas much less potential targets have been identified/confirmed so far for NFU3 [21]. Although NFU2 and NFU3 proteins display a strong sequence identity and the same domain architecture (Figure S1), the proteins may have different properties. This is illustrated by the atypical electrophoretic behavior on SDS PAGE of NFU3 that migrates like a protein of 14 kDa instead of the expected molecular mass of 17 kDa [15,19]. Moreover, protein accumulation analyses as revealed by western blot indicated that NFU3 accumulates only in the aerial tissues whereas NFU2 accumulates throughout the plant body [15,19]. Despite these recent advances, there is still a need to get more insights into the potential redundancy that is predicted to exist between Arabidopsis plastidial NFU isoforms, and in particular between NFU2 and NFU3.

In this study, we aimed at better delineating the expression and the cellular and subcellular protein localization of NFU1, NFU2, and NFU3 within the plant tissues and to decrypt the complex relationships that exist between NFU isoforms and Fe-S client proteins present in plastids. Global label-free quantitative (LFQ) proteomic was used to determine which proteins, including known plastidial Fe-S cluster-containing proteins, and thus which metabolic pathways are altered in *nfu3* mutant. This allowed to pinpoint novel potential targets for NFU3 and to compare them with those of the NFU2 paralog.

## 2. Results

### 2.1. Expression Analysis and Protein Localization of NFU1, NFU2, and NFU3

In previous studies, hypotheses on the role of NFU2 in the maturation of specific Fe-S proteins, notably explaining the root phenotype described for Arabidopsis *nfu2* mutants, were based on Western blot experiments showing that NFU2, but not NFU3, was present in roots [15,22]. However, given the poor and unspecific reactivity of the used NFU3 antibody, it remained possible that NFU3 accumulated at low levels or in specific cells and thus below the detection level of the antibody. Anyway, there is surprisingly very little information available on the expression patterns of *NFU1*, *NFU2,* and *NFU3* throughout the plant body, from seedlings to adult plants. Therefore, to help clarifying the role played by plastidial NFU isoforms, their expression pattern was further investigated using complementary approaches.

**Figure 1:**
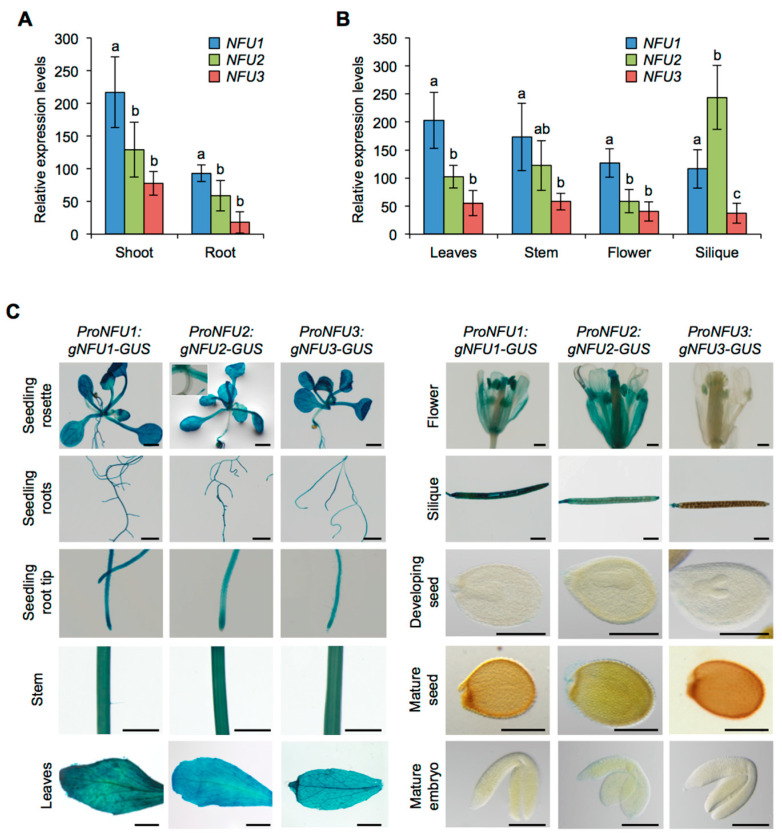
Analysis of *NFU1*, *NFU2,* and *NFU3* gene expression and protein localization in *Arabidopsis thaliana*. Analysis of *NFU1*, *NFU2,* and *NFU3* gene expression in roots and shoots of 7-day-old wild type Arabidopsis seedlings (**A**) and in leaves, stems, flowers and siliques of 4-week-old plants (**B**) by qRT-PCR. (**A**,**B**) Means within each condition with the same letter are not significantly different according to one-way ANOVA followed by post hoc Tukey test, *p* < 0.05 (*n* = 6 technical repeats from three biological replicates from one representative experiment). Error bars show the means ± SD. (**C**) NFU1, NFU2, and NFU3 protein localization revealed by GUS activity in 7-day-old seedlings and in leaves, stem, flowers, siliques, seeds, and embryos of 4-week-old plants. Bars: seedling aerial parts and roots, stems = 0.5 cm; root tips, leaves, flowers and siliques = 0.1 cm; seeds and embryos = 0.05 cm.

First, *NFU1*, *NFU2,* and *NFU3* mRNA accumulation was studied by quantitative reverse transcription-polymerase chain reaction (qRT-PCR) in wild type Arabidopsis plants (Figure 1A,B). For this purpose, aerial parts and roots from one-week-old seedlings were analyzed separately. Leaves, stems, flowers, and siliques from four-week-old plants were also assayed. The qRT-PCR results revealed that *NFU1* was the most expressed gene in comparison with other *NFUs* in all organs tested but the siliques. In seedlings, the three *NFU* genes were more expressed in the aerial tissues than in roots (Figure 1A). Although the expression levels of *NFU3* in seedling aerial tissues and roots seem lower than those of NFU2, they were not statistically different. It was also the case in leaves, stems and flowers (Figure 1B). In contrast, *NFU2* was the most expressed plastidial *NFU* gene in siliques. Altogether these data indicate that all three plastidial *NFU* genes are transcribed throughout the whole plant body, including roots.

Then, in order to get more insight into the tissular and cellular localization of the three NFU isoforms, protein localization studies in Arabidopsis seedlings and mature plants have been carried out using translational fusions between the three genomic sequences of the *NFU* genes (i.e., from about 2 kb upstream from the start codon until the last codon before the stop codon) and either the *uidA* (i.e., GUS, β-GLUCURONIDASE; *ProNFU:gNFU-GUS*) or the *GFP* (i.e., GREEN FLUORESCENT PROTEIN; *ProNFU:gNFU-GFP*) reporter genes.

GUS activity revealed that all three NFU proteins were nearly present in all tested organs in wild type seedlings (i.e., aerial tissues and roots) and in four-week-old plants (i.e., leaves, stems, flowers, and siliques) confirming the results obtained by qRT-PCR (Figure 1C). In particular, it validated the presence of NFU3 in roots. The few differences observed between NFUs were that NFU2 and NFU3 were respectively absent from hypocotyls and flowers (except in anthers). 

**Figure 2:**
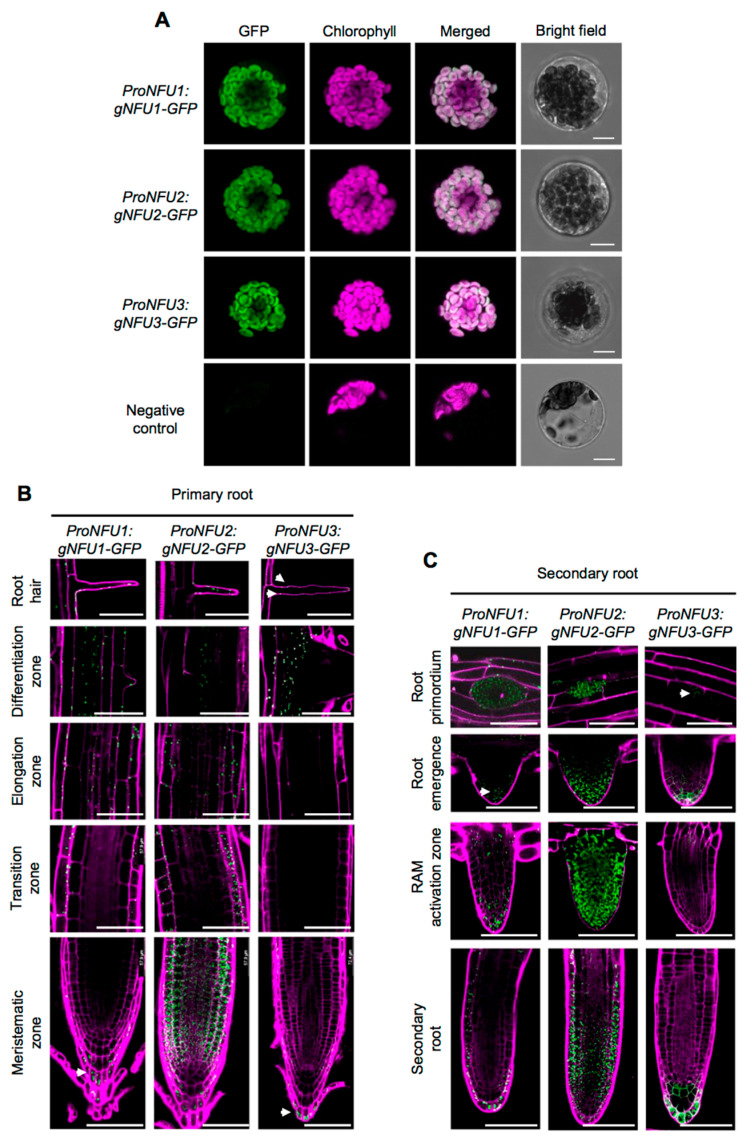
NFU1, NFU2, and NFU3 protein localization in *Arabidopsis thaliana* protoplasts and roots. Subcellular localization of NFU1-GFP, NFU2-GFP, and NFU3-GFP fusion proteins (green) expressed under the control of their own promoter with confocal microscopy in Arabidopsis protoplasts. Negative control: Untransformed protoplast. Bars: 10 µm (**A**). Subcellular localization of NFU1-GFP, NFU2-GFP, and NFU3-GFP fusion proteins expressed under the control of their own promoter with confocal microscopy in the primary (**B**) and secondary (**C**) root of 10-day-old seedlings after propidium iodide staining (magenta). RAM: Root apical meristem. Bars: 60 µm (**B**,**C**). White arrowheads indicate NFU1-GFP and NFU3-GFP weak fluorescence in roots (**B**,**C**).

Prior to analyzing GFP fluorescence in roots, the three *ProNFU:gNFU-GFP* reporter constructs were assayed in wild type Arabidopsis protoplast transfection assays to confirm that the fusion proteins were correctly addressed to the chloroplasts (Figure 2A) [20,21]. 

Similar to the GUS analysis, all three NFUs displayed relatively similar localization patterns within the roots. Concerning the apical and basal meristems, NFU1, NFU2, and NFU3 were all detected in the columella and the lateral root cap, but NFU2 was additionally highly present in the cortex and the epidermis (Figure 2B). At the transition zone, both NFU1 and NFU2 were present but not NFU3, a differential pattern that was conserved up to the elongation zone. The differentiation zone and root hairs appeared as the sole regions of the primary root shared by the three NFUs. Regarding complex secondary structures, differential patterns between NFUs were again observed (Figure 2C). NFU2 was highly abundant in root primordia and during later steps of the secondary root development, while NFU1 and NFU3 remained respectively discrete and almost absent from these structures. In elongated secondary roots, all NFUs retained similar expression patterns to that observed in primary roots.

Altogether these results indicate that *NFU1*, *NFU2,* and *NFU3* are expressed throughout the plant body from the early steps of seedling development, but not in seeds and embryos. It also suggests that the three encoded proteins follow, overall, a pattern of accumulation that is similar to their corresponding transcripts, including a presence in roots where they accumulate with slight specificities depending on the root structures.

### 2.2. Global Analysis of the Biological Function of Proteins Whose Abundance is Affected in nfu3-2 Mutant

In order to uncover the pathways affected in *nfu3* loss-of-function mutants and to identify potential new NFU3 targets, global label free quantitative (LFQ) proteomic analyses were conducted. For this purpose, two-week-old wild type and *nfu3-2* whole seedlings were harvested for total protein extraction and liquid chromatography coupled with mass spectrometry (LC-MS/MS) analysis. In total, 5237 proteins were identified and 2881 were quantified. Among the proteins whose accumulation varied significantly between both genotypes, 775 displayed a logarithmic fold change [log2(FC)] lower than −0.2 or higher than +0.2 between wild type and *nfu3-2* samples with 346 accumulating to a lesser extent and 429 accumulating to a greater extent in *nfu3-2* compared to the wild type (Tables S1 and S2). 

**Figure 3:**
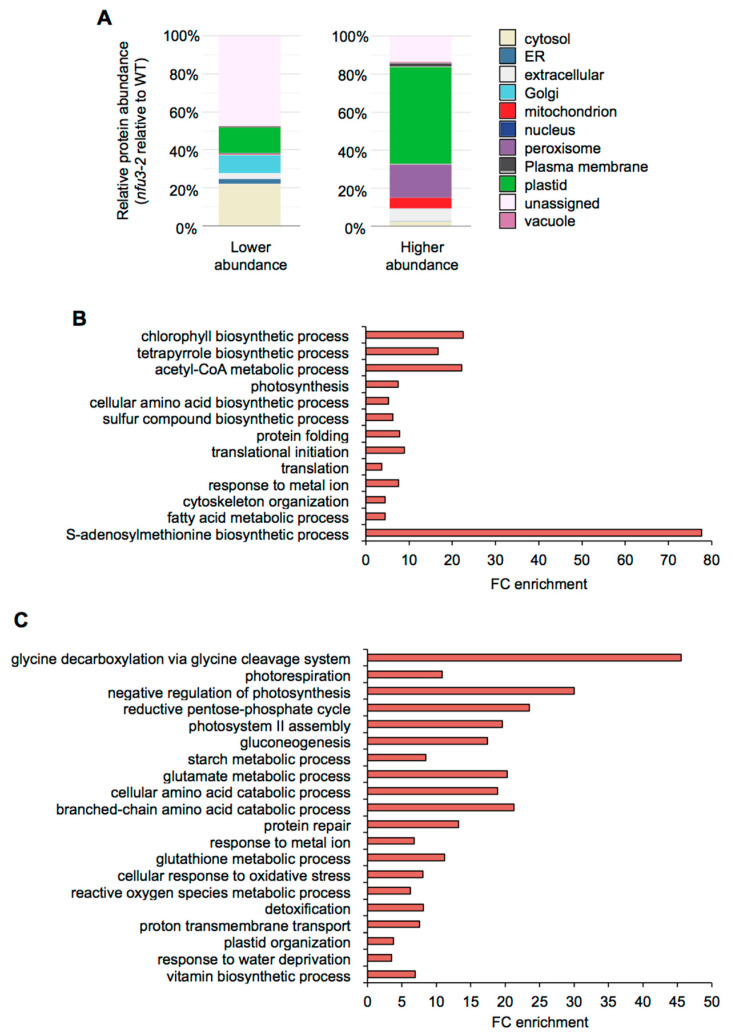
Subcellular localization and gene ontology analysis of proteins whose abundance is modified in the *Arabidopsis thaliana nfu3-2* mutant compared to the wild type. Protein localization was analyzed in silico using the multiple marker abundance profiling method of the SUBA4 bioinformatic platform (http://suba.live/) (**A**). Gene ontology (GO) term enrichment analysis was conducted using the PANTHER gene ontology platform (http://go.pantherdb.org) considering proteins whose abundance was significantly decreased (**B**) or increased (**C**) in *nfu3-2* mutant when compared to the wild type. Significant GO term enrichment was determined using Fisher exact test with Bonferroni correction.

The potential subcellular localization of these 775 proteins was analyzed using the multiple marker abundance profiling method of the SUBA4 bioinformatic platform (http://suba.live/; [23] (Figure 3A, Table S3).

Since NFU3 is a chloroplastic protein involved in the maturation of the PsaA, PsaB, and PsaC subunits of the PSI, one would expect enrichment in plastid-localized proteins, as observed in *nfu2-1* seedlings [21]. Surprisingly, in the list of proteins whose abundance was diminished in the *nfu3-2* mutant, most of the proteins had no assigned subcellular localization (44%). The following major terms for the subcellular localization were then “cytosolic” (22%) and the “plastidial” localization only appeared in third position (14%) followed by “Golgi” (10%), “extracellular” (3%) and “endoplasmic reticulum” (3%). On the contrary, in the list of proteins whose abundance was increased in the *nfu3-2* mutant, “plastidial” localization was greatly represented (51%) followed by “peroxisome” (18%), “extracellular” (10%), and “mitochondrion” (7%). Taken together, these results indicate that among the proteins for which there is a predicted subcellular localization, the *nfu3-2* mutation mainly impacts the accumulation of plastid-localized proteins. However, the absence of NFU3 has also strong consequences on the accumulation of proteins localized in cell compartments other than plastids (e.g., cytosol, Golgi, peroxisome, extracellular, or mitochondrion) indicating profound changes in the cell organization. As an organelle required for the cellular energy metabolism, this is not so unexpected that a malfunctioning of the chloroplastic electron transfer chain has some impact on the mitochondrial and peroxisome metabolisms.

Then, gene ontology (GO) term enrichment analysis for the proteins whose abundance varied in *nfu3-2* was conducted using the PANTHER GO platform (http://go.pantherdb.org) (Figure 3B,C).

The most significantly enriched term among proteins with a decreased accumulation in *nfu3-2* (Figure 3B) is “S-adenosylmethionine biosynthetic process” (GO:0006556). This is likely due to the diminution of the amount of the four S-adenosyl-methionine synthase (SAM) isoforms, SAM1, SAM2, SAM3, and SAM4 (Table S1). The transfer of methyl groups from SAM is catalyzed by many methyltransferases to a large variety of molecules, including hormones, lipids, proteins and nucleic acids [24]. Noteworthy, several radical SAM Fe-S proteins are present in chloroplasts (notably the LIPOATE SYNTHASE) and their activity may be affected in the *nfu3-2* genetic background. It is thus possible that defects in SAM synthesis have important consequences and thus contribute to the strong growth and development defects observed in *nfu3-2* mutant [15,19].

Among other proteins whose accumulation was decreased in *nfu3-2*, the “photosynthesis” (GO: 0015979) term was as expected significantly enriched. This is in agreement with the observed diminution of abundance of PSI reaction center proteins that contain Fe-S cluster (i.e., PsaA, PsaB, and PsaC) and of some other subunits, reflecting the global disorganization of the PSI apparatus (i.e., PsaD-1, PsaD-2, PsaE-1, PsaF, PsaH-1, PsaL, PsaN, and Lhca-3) (Table 1) as reported by previous fluorescence measurements [15,19]. Consequently, terms related to photosynthesis failure were also significantly enriched among proteins that displayed a lower accumulation in *nfu3-2*. It included “chlorophyll and pigment biosynthesis” (GO: 0015995 and GO: 0046148), “acetyl-CoA metabolism” (GO: 0006084), “tetrapyrrole biosynthetic process” (GO: 0033014), “cellular amino acid biosynthetic process” (GO: 0008652), and “sulfur compound biosynthetic process” (GO: 0044272).

Interestingly, the “photosystem II (PSII) assembly” (GO: 0010207) term is significantly enriched among proteins over-accumulated in the *nfu3-2* (reflecting the accumulation of extrinsic proteins, Figure 3C, Table 1), maybe as another consequence of PSI failure [20]. However, the abundance of quantifiable proteins that are part of the PSII reaction center or antenna was not affected in *nfu3-2* (Table 1).

Among other over-accumulated proteins in *nfu3-2*, many terms related to the carbon/energy metabolism were significantly enriched likely as a consequence of photosynthesis defects. It included “glycine decarboxylation via glycine cleavage system” (GO: 0019464), “photorespiration” (GO: 0009853), “negative regulation of photosynthesis” (GO: 1905156), “reductive pentose phosphate cycle” (GO: 0019253), “gluconeogenesis” (GO:0006094), “starch metabolic process” (GO: 0005982), “cellular amino acid catabolic process” (GO: 0009063), and “branched-chain amino acid catabolic process” (GO: 0009083). The “glutamate metabolic process” term (GO: 0006536) was also significantly enriched in *nfu3-2*. Among the proteins related to the “glutamate metabolic process” term were GLU1 (FERREDOXIN-DEPENDENT GLUTAMATE SYNTHASE 1), a Fe-S cluster protein, and GHD1 and GHD2 (GLUTAMATE DEHYDROGENASES 1 and 2). Previous studies suggested that GDHs are involved in supplying an alternative carbon source to the respiratory chain during sugar starvation [25,26]. Similarly, the mitochondrial ISOVALERYL-CoA-DEHYDROGENASE and ETFQO (ELECTRON-TRANSFER FLAVOPROTEIN:UBIQUINONE OXIDOREDUCTASE), which link amino acid degradation to the respiratory chain [27], are also among the most accumulated proteins (Table S1). Hence, these observed variations in protein abundance probably reflect a carbon starvation situation due to the decrease in carbon assimilation related to the defects in the photosynthesis apparatus. Finally, the “proton transmembrane transport” (GO: 1902600) term in which eight subunits of the vacuolar V-type proton ATPase and two subunits of the chloroplastic ATPase were affected (Table 1), also likely reflects a nutrient deficiency response and/or the establishment of a stress response [28], as does most other remaining GO terms i.e., “protein repair” (GO:0030091), “glutathione metabolic process” (GO: 0006749), “cellular response to oxidative stress” (GO:0034599), “reactive oxygen species metabolic process” (GO: 0072593), and “detoxification” (GO:0098754). This seems consistent with the described accumulation of singlet oxygen and superoxide ion in *nfu3* mutants [19].

Previous studies have shown that the fatty acid metabolism was stimulated in *nfu2* [21] and in other photosynthetic mutants [29]. Accordingly, in the *nfu3-2* mutant, several enzymes related to the biosynthesis of fatty acids displayed a higher abundance than in the wild type (Figure S2). However, the GO term “fatty acid metabolic process” (GO: 0006631) was found significantly enriched in lower accumulated proteins in *nfu3-2* mutant, most probably reflecting potential defect in wax biosynthesis. For instance, among the proteins were the KCR1 (β-KETOACYL–CoA REDUCTASE 1), the CER10 (ECERIFERUM 10: ENOYL-CoA REDUCTASE 10) and a putative regulator of very long chain fatty acid (VLCFA) elongation CER2 (ECERIFERUM 2) (Table S1), three proteins implicated in the elongation pathway of waxes [30]. In addition, CER3 (ECERIFERUM 3: A putative very long chain-acyl-CoA reductase) and an LACS2 (ACYL-CoA SYNTHASE) were among the proteins that were not detected in the *nfu3-2* mutant (Table S2).

Finally, the “Plastid organization” (GO: 0009657) term is significantly enrichment among the over accumulated proteins in *nfu3-2* and the “translational initiation” (GO: 0006413), “translation” (GO: 0006412), “protein folding” (GO: 0006457), and “cytoskeleton organization” (GO: 0007010) terms in the lower represented proteins in *nfu3-2* might reflect a more global perturbation of the cell that correlates with the wide distribution of the subcellular localization of the affected proteins.

In summary, all these described proteome changes reflect the establishment of (i) a general stress response but also of (ii) intense catabolic processes to sustain the furniture of carbon skeleton and of (iii) compensatory photorespiratory metabolism to cope with photosynthesis failure. This explains why the accumulation of numerous extra-chloroplastic proteins is modulated in the *nfu3-2* mutant.

### 2.3. The Biological Function of Proteins Whose Abundance is Affected in nfu3-2 and nfu2-1 Does Not Fully Overlap

NFU3 and NFU2 are the closest NFU paralogs and loss-of-function mutation of both genes affects the photosynthetic machinery [15,19,21]. In order to determine to which extent their biological functions overlap, the lists of proteins whose abundance was affected by the *nfu3-2* and *nfu2-1* mutations, as revealed by LFQ proteomic analysis, were compared since a similar study was performed previously using plants harvested at the same developmental stage and grown in the same conditions [21]. 

**Figure 4:**
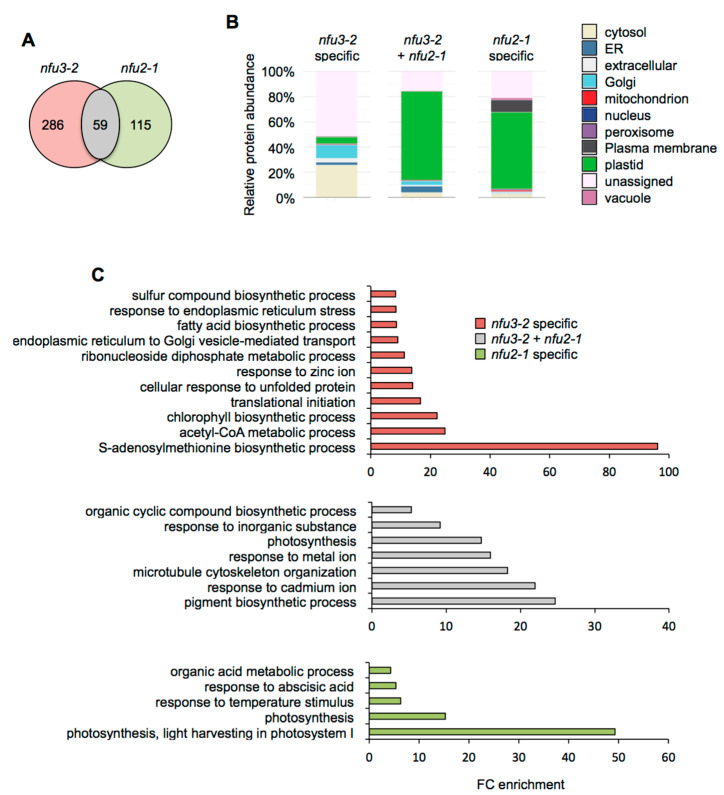
Comparison of proteins whose abundance is decreased in the *Arabidopsis thaliana nfu3-2* and *nfu2-1* mutants when compared to the wild type. Venn diagram comparing the proteins whose abundance is decreased in both *nfu3-2* and *nfu2-1* mutants (whole seedlings). Seedlings were grown as described in [21]. Venn diagram was done with Venny 2.1 (https://bioinfogp.cnb.csic.es/tools/venny/index.html) (**A**). Subcellular localization of proteins whose abundance is decreased in both *nfu3-2* and *nfu2-1* mutants. Protein localization was analyzed in silico using the multiple marker abundance profiling method of the SUBA4 bioinformatic platform (http://suba.live/) (**B**). Gene ontology (GO) analysis of proteins whose abundance is decreased in both *nfu3-2* and *nfu2-1* mutants. GO term enrichment analysis was conducted using the PANTHER gene ontology platform (http://go.pantherdb.org). Significant GO term enrichment was determined using Fisher exact test with Bonferroni correction (**C**).

Considering the 59 proteins whose accumulation is decreased in both mutants (Figure 4A), the major term for the subcellular localization was, as expected, “plastidial” localization (70%) (Figure 4B, Table S4). Accordingly, the GO term analysis showed a significant enrichment of the “photosynthesis” (GO: 0015979) and “pigment biosynthesis process” (GO: 0046148) terms (Figure 4C). This is consistent with the PSI defects observed in both mutants. Then, analyzing the specific proteome changes in the respective mutants may give clues to the establishment of specific responses.

For the 115 proteins whose abundance was specifically decreased in *nfu2-1* (Figure 4A), the major term for the subcellular localization was also “plastidial” localization (61%) (Figure 4B) and was associated with the “photosynthesis” (GO: 0015979) and the “photosynthesis, light harvesting in photosystem I” (GO: 0009768) GO terms. This is tentatively explained by more global and specific PSI defects in the *nfu2* mutant. The presence of the GO term “response to abscisic acid” (GO: 0009737) fits with the described decreased ABA content and sensitivity to dehydration reported for the *nfu2-1* mutant [21].

In contrast, there is no major term for a cell compartment among the 286 proteins whose abundance was specifically decreased in *nfu3-2* (Figure 4A,B). These results, together with the various GO terms that were significantly enriched, confirmed that the *nfu3-2* mutation leads to a global cellular perturbation, the enrichment of the “S-adenosylmethionine biosynthetic process” (GO: 0006556) remaining obviously predominant (Figure 4C).

**Figure 5:**
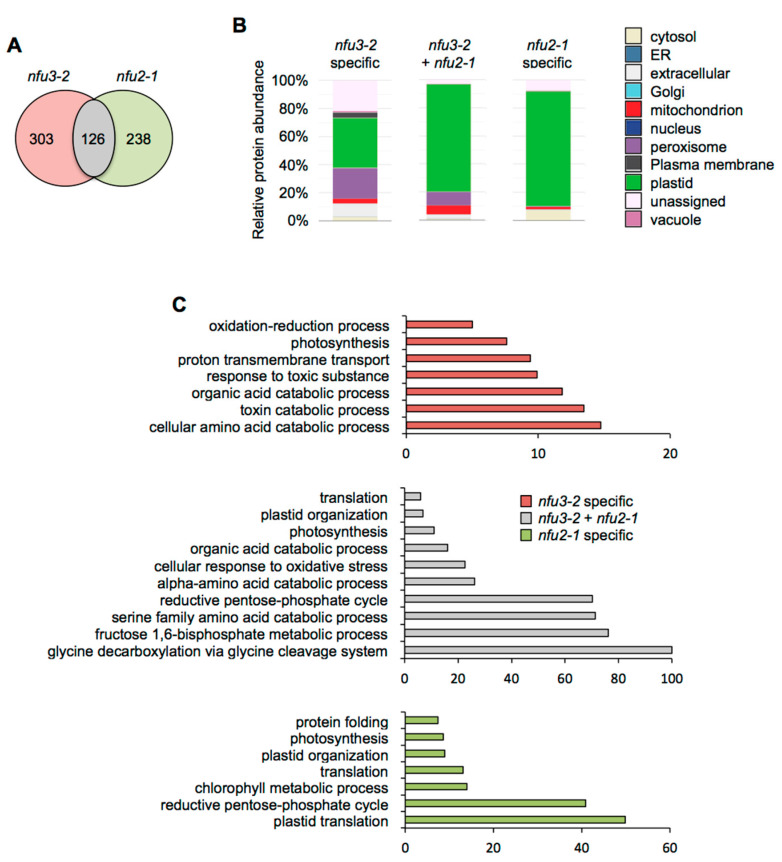
Comparison of proteins whose abundance is increased in the *Arabidopsis thaliana nfu3-2* and *nfu2-1* mutants when compared to the wild type. Venn diagram comparing the proteins whose abundance is increased in both *nfu3-2* and *nfu2-1* mutants (whole seedlings). Seedlings were grown as described in [21]. Venn diagram was done with Venny 2.1 (https://bioinfogp.cnb.csic.es/tools/venny/index.html) (**A**). Subcellular localization of proteins whose abundance is increased in both *nfu3-2* and *nfu2-1* mutants. Protein localization was analyzed in silico using the multiple marker abundance profiling method of the SUBA4 bioinformatic platform (http://suba.live/) (**B**). Gene ontology (GO) analysis of proteins whose abundance is increased in both *nfu3-2* and *nfu2-1* mutants. GO term enrichment analysis was conducted using the PANTHER gene ontology platform (http://go.pantherdb.org). Significant GO term enrichment was determined using Fisher exact test with Bonferroni correction (**C**).

Subcellular localization analysis of the 126 proteins whose abundance was increased in both *nfu3-2* and *nfu2-1* mutants revealed that “plastidial” localization (77%) was once again the main term (Figure 5A,B, Table S5). The terms “peroxisome” (9%) and “mitochondrion” (7%) appeared in second and third positions, respectively. The GO term analysis pointed to the establishment of a response to photosynthesis deficiency with significantly over-represented GO terms including “reductive pentose phosphate cycle” (GO: 0019253), “glycine decarboxylation via glycine cleavage system” (GO: 0019464), “fructose 1,6-bisphosphate metabolic process” (GO: 0030388) as well as “photosynthesis” (GO: 0015979) and “plastid organization” (GO: 0009657) (Figure 5C). In addition the terms “serine family amino acid catabolic process” (GO: 0009071), “alpha-amino acid catabolic process” (GO:1901606) and “organic acid catabolic process” (GO: 0016054) were also significantly enriched, most probably reflecting the energy demand of the young seedlings for ensuring their survival [31].

“Plastidial” localization (82%) was the main term associated with the 238 proteins whose abundance was specifically increased in *nfu2-1* (Figure 5A,B). GO term analysis was in adequacy with this observation (Figure 5C). Subcellular localization and GO term analyses support the fact that the main defects and metabolic adjustments operating in the *nfu2-1* mutant relate to the photosynthetic machinery.

Considering the 303 proteins whose abundance was specifically increased in *nfu3-2* (Figure 5A), subcellular localization analysis showed that the “plastid” (36%) and the “peroxisome” (22%) terms were the most affected (Figure 5B). The GO term analysis of the corresponding proteins suggested that metabolic adjustments to maintain the growth of the plant and to respond to the oxidative stress were operating. 

Overall, it is interesting to note that only a minor part of the proteome changes occurring in the *nfu2* and *nfu3* mutants are common (i.e., those directly related to photosynthesis) and thus that the proteomic and subsequent metabolic adjustments established in these mutants should differ quite considerably.

### 2.4. Quantification of Chloroplastic Fe-S Proteins in the nfu3-2 Mutant

Among the 53 predicted plastidial Fe-S proteins and the 33 proteins that have been detected and quantified (Table 2), the abundance of 18 Fe-S proteins was affected by the *nfu3-2* mutation. Nine proteins displayed a lower abundance in the *nfu3-2* mutant. It included ASE2 (GLUTAMINE PHOSPHORIBOSYL PYROPHOSPHATE AMIDOTRANSFERASE 2), DWAR27.2 (β-CAROTENE ISOMERASE D27.2), HCF101 (HIGH CHLOROPHYLL FLUORESCENCE 101), GLT1 (NADH-dependent GLUTAMATE SYNTHASE 1), NIR (NITRITE REDUCTASE), PetC (PHOTOSYNTHETIC ELECTRON TRANSFER C), PsaA, PsaB and PsaC. Four additional proteins were only detected in wild type plants, namely FdC1 and FdC2, (FERREDOXIN LIKE 1 and 2), HCAR (7-HYDROXYMETHYL CHLOROPHYLL A REDUCTASE) and THIC (4-AMINO-5-HYDROXYMETHYL-2-METHYLPYRIMIDINE PHOSPHATE SYNTHASE), which may indicate that a maturation defect led to protein degradation and to their absence of detection in the *nfu3-2* mutant.

**Figure 6:**
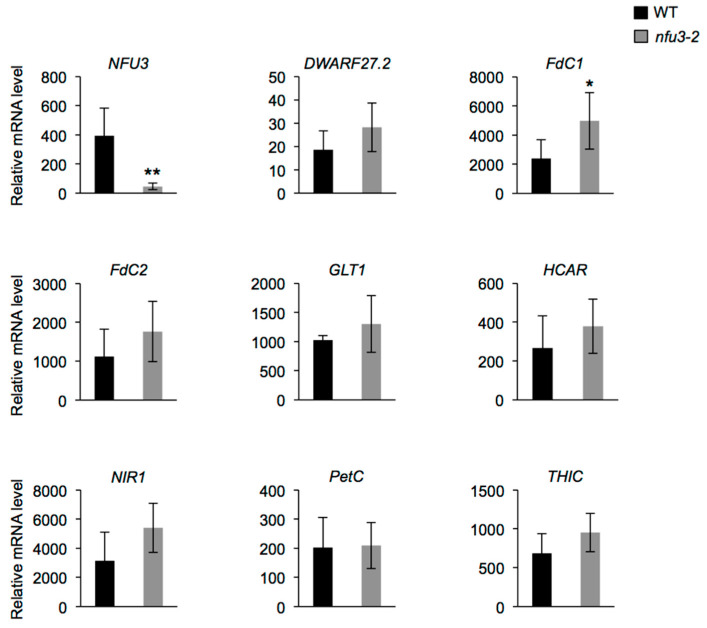
Analysis of *DWARF27.2*, *FdC1*, *FdC2*, *GLT1*, *HCAR*, *NIR*, *PetC,* and *THIC* gene expression in *Arabidopsis thaliana* wild type and *nfu3-2* mutant. Analysis of *DWARF27.2*, *FdC1*, *FdC2*, *GLT1*, *HCAR*, *NIR*, *PetC,* and *THIC* gene expression in 2-week-old wild type and *nfu3-2* Arabidopsis seedlings by qRT-PCR. *t*-test significant difference: * *p* < 0.05; ** *p* < 0.01. (*n* = 6 technical repeats from three biological replicates from one representative experiment). Error bars show the means ± SD.

Among these proteins, eight are novel potential NFU3 targets (i.e., DWARF27.2, FdC1, FdC2, GLT1, HCAR, NIR, PetC, and THIC). mRNA levels were measured (qRT-PCR) in two-week-old wild type and *nfu3-2* seedlings to determine whether or not the protein abundance decrease observed in the *nfu3-2* mutant was due to a decreased gene expression (Figure 6). *NFU3* mRNA decreased accumulation in the mutant was first confirmed. This experiment revealed that *FdC1* transcript accumulation was increased in *nfu3-2* when compared to the wild type, suggesting that a compensatory mechanism occurring at the expression level is at play to maintain FdC1 protein level in the mutant. In contrast no change was observed for the other seven genes between both genotypes. Taken together, these observations support a role for NFU3 in maturating these eight plastidial Fe-S proteins.

In contrast, five proteins displayed an increased accumulation in the *nfu3-2* mutant. These proteins were GLU1 (GLUTAMATE SYNTHASE 1), PSB33 (PHOTOSYSTEM B PROTEIN 33), PAO (PHEOPHORBIDE A OXYGENASE), PTC52 (PROTOCHLOROPHYLLIDE-DEPENDENT TRANSLOCON COMPONENT 52) and TIC55 (TRANSLOCON AT THE INNER ENVELOPE MEMBRANE OF CHLOROPLAST 55). GLU1 contains the less frequently encountered [3Fe-4S] cluster types and PAO, PTC52, and TIC55 a Rieske-type [2Fe-2S] cluster. A recent study suggested that NFU3 can assemble [3Fe-4S] clusters [19] but a firm validation was hampered by the difficulty to express a soluble, non-aggregated recombinant protein [15]. The accumulation of GOGAT in *nfu3-2* was previously described [15,19]. Interestingly, the last three proteins participate in chlorophyll degradation pathway and it is possible that their accumulation only compensates the observed decrease of the [4Fe-4S] cluster-containing HCAR, a protein acting upstream in the chlorophyll degradation pathway.

## 3. Discussion

### 3.1. Plastidial NFUs Display Overlapping Patterns of Expression

Plastidial NFU proteins are present in various photosynthetic organisms, ranging from cyanobacteria (e.g., *Synechocystis* sp. PCC 6803), algae (e.g., *Chlamydomonas reinhardtii*) to land plants [15,22,32]. Previous phylogenetic analysis indicated that NFU1 clusters independently from NFU2 and NFU3 [15,21] suggesting that after the appearance of NFU2 and NFU3 by duplication from an ancestral gene, each protein may have acquired novel independent functions. Previous studies indicated also that NFU1, NFU2, and NFU3 have overlapping client proteins but also specific ones [15,21]. Accordingly, the growth defects observed for *nfu2* and *nfu3* mutants indicate that the functions of plastidial NFU proteins do not fully overlap and thus that NFU2 and NFU3 play at least some independent roles in the maturation of Fe-S proteins in addition to alimenting HCF101 for PSI subunit maturation [15]. Whether NFU1 possesses specific roles and partners remains unclear since there is no phenotype described for a *nfu1* mutant to date [15]. If so, NFU1 should target proteins that are not essential for growth and development. The existence of a root growth phenotype observed only for the *nfu2* mutant was tentatively explained by differential expression patterns of the three proteins [15]. In this former study, western blot analysis revealed that the three NFU proteins accumulated in the aerial tissues whereas NFU2 was the only or predominant one to accumulate in roots. Aware of the usual issues associated to western blot analysis or to differences in plant growth conditions, more thorough, complementary expression studies and protein localization assays were conducted (Figure 1 and Figure 2). These approaches underlined that NFU genes and proteins were expressed to somehow similar levels in most organs tested including roots, with however, differences in a few tissues such as the meristematic zone and the separating cell layer of the roots but also the hypocotyls, the flowers and the silique valves. Thus, these experiments indicated that a localization of the plastidial NFU proteins in specific plant organs or tissues may not fully explain the different phenotypes of the *nfu* mutants.

### 3.2. Proteomic Adjustments in the nfu3 Mutant Suggest both Common and Specific Metabolic and Physiological Adjustments with nfu2

Besides the already described PSI defects and associated photosynthesis deficiency (Table 1), other proteome changes observed in the *nfu3-2* mutant can be summarized as responses to oxidative stress and to carbon shortage (Figure 3). It is noticeable that several of these changes differ quite substantially from those established in a *nfu2* mutant, indicating that the dialog between chloroplasts and other organelles to coordinate the proper cellular responses is different (Figure 4 and Figure 5). Analysis of the subcellular localization of varying proteins underlines the predominance of a chloroplastic response in the case of *nfu2* mutation whereas the *nfu3* mutation generates a larger extra-chloroplastic response. Comparing with *nfu2* or with other known photosynthetic mutants, the most disturbed pathway in *nfu3-2* is the SAM biosynthetic process. Because of the localization of these enzymes that are not predicted to be plastidial, a direct link between SAM-dependent enzymes and NFU3 activity is unlikely. A second striking observation that cannot be directly connected to a chloroplastic Fe-S cluster protein failure either, is the lower accumulation of enzymes implicated in the elongation of waxes, process that takes place in the endoplasmic reticulum. Incidentally, two GO terms related to ER stress and transport stand out from the list of *nfu3-2* specific proteomic changes (Figure 4C). We hypothesize that this can be a consequence of the perturbed acetyl CoA metabolism observed in the GO term analysis.

### 3.3. Variations in Fe-S Proteins

**Figure 7:**
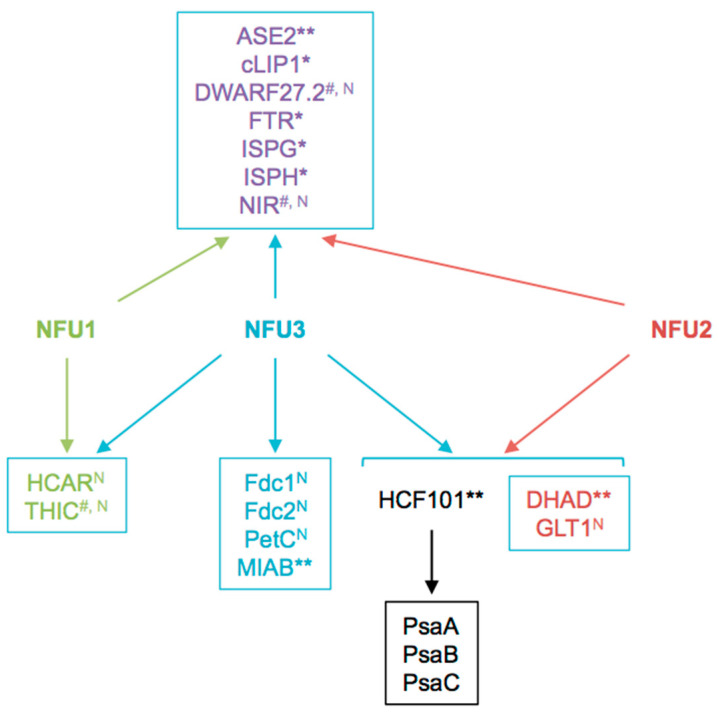
Summary scheme integrating known and newly identified potential client proteins of NFU3 and redundancy with its NFU1 and NFU2 paralogs. Among the 16 NFU3 potential client proteins, eight were newly identified in this study by label-free quantitative proteomic analysis. ^N^: Novel NFU3 potential targets identified in this study. *: Interactions between NFU3 and apo-targets previously confirmed by BiFC (in [21]), **: Interactions between NFU3 and apo-targets previously confirmed by Y2H (in [15,21]), ^#^: Interactions between NFU3 and apo-targets not confirmed by BiFC and/or Y2H in previous studies (in [21]).

This proteomic study allowed us to focus on the Fe-S proteins affected by the *nfu3-2* mutation (Table 2). It is obviously difficult to determine which are direct client proteins of NFU3. Indeed, the assumption is made that apo-proteins will be instable and degraded but this is not true for all proteins and the levels of a given partner protein could stay unchanged despite maturation defects. Moreover, an increase in protein abundance may eventually result from a maturation defect as well if the accumulation of the protein serves for compensating a default in Fe-S cluster maturation. So far, it is not possible to follow Fe-S cluster incorporation in a given protein *in planta*. In this study, we observed that the abundance of 18 proteins was modified in *nfu3-2* mutant seedlings, 13 had decreased abundance or have not been detected specifically in the mutant and are considered as absent. The five proteins displaying an increased accumulation in the *nfu3-2* mutant have not been considered here, but for the reasons explained above, we do not completely exclude them as Fe-S client proteins of NFU3. Among the 13 remaining proteins, eight proteins may be novel targets of NFU3, namely DWARF27.2, GLT1, NIR, PetC, FdC1, FdC2, HCAR, and THIC (Figure 6 and Figure 7). Indeed, NFU3 was already shown to interact with ASE2 and HCF101 in previous studies and to impact the levels of PsaA, PsaB, and PsaC, very likely *via* the intermediate of HCF101 [15,21]. While a decrease of HCF101 abundance has never been observed in *nfu* mutants, this proteomic approach allows unveiling a weak difference in protein abundance between WT and *nfu3-2* seedlings. These data likely confirm HCF101 as a NFU3 partner protein but also the fact that NFU3 is not the sole Fe-S cluster carrier protein alimenting HCF101.

The decreased abundance observed for NIR in this study was not in adequacy with former studies in which NIR activity was not affected in *nfu3* mutant [15]. One explanation might be that protein activity and levels are not correlated. Another might be the developmental stage of the plants (i.e., four-week-old vs. seedlings). Such explanation might also apply to PetC as its abundance was shown to increase in four-week-old *nfu3* mutants and decreased in this study [19]. Interestingly, PetC is a Rieske-type [2Fe-2S] cluster-containing protein but only NFU2 was shown so far to incorporate a [2Fe-2S] cluster [12]. Interaction of NFU3 with DWARF27.2 and THIC was previously tested in yeast two-hybrid (Y2H) and/or bimolecular fluorescence complementation (BiFC) assays without any success [21]. Hence, they could be viewed as indirect targets. Considering that HCF101 was described to act downstream NFU2 and NFU3 in shoots [15], and that HCF101 abundance is decreased in *nfu3-2*, the observed decreased abundance of DWARF27.2 and NIR in *nfu3-2* might be due to a decrease of HCF101 activity. Whether or not NFU3 directly interacts with GLT1, PetC, FdC1, FdC2, and HCAR necessitates further investigations.

In previous studies, NFU3 was found to interact in Y2H or in BiFC experiments with ASE2, FTR (FERREDOXIN-THIOREDOXIN REDUCTASE), IPMI LSU1 (ISOPROPYLMALATE ISOMERASE LARGE SUBUNIT 1), ISPG (4-HYDROXY-2-METHYLBUT-2-ENYL DIPHOSPHATE SYNTHASE), ISPH (4-HYDROXY-2-METHYLBUT-2-ENYL DIPHOSPHATE REDUCTASE), cLIP1 (chloroplastic LIPOIC ACID SYNTHASE) and MIAB (METHYLTHIOTRANSFERASE). LFQ analysis revealed that the protein abundance of FTR, IPMI LSU1, ISPG, and ISPH was not affected by the *nfu3-2* mutation whereas ASE1, cLIP1, and MIAB abundance was not quantifiable. Noteworthy, IPMI LSU1 was shown to interact with NFU2 but its abundance was not affected by the *nfu2-1* mutation [21]. Hence, it may not be surprising that IPMI LSU1 abundance does not vary in the *nfu3-2* mutant. In the case of ISPG and ISPH (two enzymes from the non-mevalonate/methyl-erythritol pathway involved in isoprenoid biosynthesis), their accumulation was not affected by the *nfu2-2* mutation either whereas interactions were demonstrated between these two enzymes and NFU2 [21]. Overall, these data suggest that IPMI LSU1, ISPG, and ISPH get their Fe-S cluster from NFU3 in seedlings and that NFU1 or NFU2 are sufficient to compensate the lack of NFU3 activity. 

In summary, this study allows to go deeper into the NFU1, NFU2, and NFU3 network (Figure 7) and function by showing notably that the three proteins are present in all plant tissues, from the seedling stages to adult plants, and by identifying eight new potential targets of NFU3. It also highlights that it may be necessary to go at the level of the tissular and cellular expression of both the maturation factor(s) and client protein(s) to find specificities in protein-protein interactions and explain some phenotypic differences between *nfu2* and *nfu3* mutants. Finally, the quite different proteome adjustments occurring in the *nfu2* and *nfu3* mutants suggest that different metabolic and physiological adjustments are established and thus clearly indicate that the NFU2 and NFU3 functions are not strictly superimposable. Exploring the proteome changes occurring in the *nfu1* loss-of-function mutant may help expanding our understanding of the respective network of these three NFU paralogs.

## 4. Materials and Methods

### 4.1. Plant Material and Growth Conditions

*nfu3-2* [19] and the wild type Columbia genotypes were germinated and grown under long day conditions (16-h-light/8-h-dark cycle; light intensity: 120 mmoL/cm^2^/s). For proteomic analysis, two-week-old seedlings were grown on half-strength Murashige and Skoog medium containing 0.05% (*w/v*) MES, 1% (*w/v*) sucrose, 0.7% (*w/v*) agar and 50 µM iron provided as Fe(III)-EDTA as described in [21]. For histochemical GUS detection and GFP confocal observation, seedlings were grown under the same condition for 7 and 10 days, respectively. Four-week-old plants were grown on soil at 23 °C with a sunlight intensity limited to 300 µmol.m^−2^.s^−1^ and 16 h of light/8 h of dark. 

### 4.2. Cloning

*NFU1*, *NFU2,* and *NFU3* genomic sequences from about 2 kb upstream from the start codon (2033 bp, 2579 bp, 2595 bp, respectively) and until the last coding codon (without the stop codon) were cloned into the pDONR201 vector and recombined into the pGWB3 and pGWB4 binary vectors [33]. The primers used are described in Table S6. All PCR products were obtained using high-fidelity Phusion DNA polymerase, and each construct was sequenced to ensure its integrity. 

### 4.3. Histochemical GUS Detection and GFP Confocal Observation

Histochemical GUS detection was performed according to [34,35]. The acidified chloral hydrate–glycerol solution was prepared by dissolving 45 g chloral hydrate into a solution consisting of 25 mL 4.2% HCl and 10 mL glycerol. GFP confocal observations were carried out as described in [34]. GUS and GFP images are representative of 20 and 10 independent transgenic lines (at least 20 individuals per line were analyzed), respectively. Images shown in Figure 2C were recorded with maximum Z-stack intensity projection.

### 4.4. Gene Expression Analysis

Total RNAs were extracted using the GenElute^™^ *Mammalian* Total *RNA Purification Kit* (Sigma-Aldrich, Saint-Louis, MO, USA). For each sample, 1 μg of total RNA treated with DNase was reverse transcribed into cDNA using the RevertAid kit (Thermo scientific, Waltham, MA, USA). qRT-PCR analyses were performed using a LightCycler^®^ 480 (Roche, Bâle, Switzerland) and TB Green Premix Ex Taq (2X) (Takara, Kusatsu, Shiga, Japan). PP2AA3 (PROTEIN PHOSPHATASE 2A SUBUNIT A3) was used as a reference gene [36]. Expression levels were calculated using the comparative threshold cycle method. The primers used are described in Table S6.

### 4.5. Liquid Chromatography Coupled with Mass Spectrometry (LC-MS/MS) Analysis

Protein extraction from whole seedlings, trypsin digestion and MS analysis were carried out as described in [21]. Raw mass spectrometric data were analyzed in the Maxquant environment (v.1.5.5.1, Max-Planck-Institute of Biochemistry, Planegg, Germany) [37] and Andromeda was employed for database search [38]. The MS/MS data were matched against the TAIR10 database. For protein identification and quantification, cysteine carbamidomethylation was set up as fixed modification and oxidation of methionine as a variable modification. At least two peptides were required for protein identification and quantification. Up to two missed cleavages was allowed for protease digestion. For other characteristics, Maxquant default parameters were used. Following the quantification step and the label free quantitative (LFQ) normalization, proteins were considered as quantifiable only if they are present in all samples. Data were log2 transformed prior analysis. For statistics, pairwise *t*-tests *p* < 0.05 were carried out. Before statistical treatment, the normal distribution of the logarithmic transformed data was assessed. For the present/absent analysis, a protein was considered as absent in the *nfu3-2* mutant if it was identified with at least 2 peptides in at least 3 replicates achieved with WT plants and not in the 4 replicates achieved with *nfu3-2* plants. All raw MS data and Maxquant files generated have been deposited to the ProteomeXchange Consortium via the PRIDE partner repository with the dataset identifier PXD020228.

## Figures and Tables

**Table 1:** Abundance of photosystem I- and II-associated proteins in the *Arabidopsis thaliana nfu3-2* mutant relative to wild type.

ID	Acronym	log2 (FC) *nfu3-2* Seedlings	log2 (FC) *nfu2-1* Seedling	Function	Name
AtCg00350	PsaA	−2.08 **	−1.68 **	PSI core	Photosystem I subunit A
AtCg00340	PsaB	−1.84 **	−1.81 **	PSI core	Photosystem I subunit B
AtCg01060	PsaC	−1.51 *	−2.1 **	PSI core	Photosystem I subunit C
At1g31330	PsaF	−1.63 **	−2.05 *	PSI core	Photosystem I subunit F
At1g55670	PsaG	n.v.	−1.58 **	PSI core	Photosystem I subunit G
At3g16140	PsaH-1	−1.42 **	n.d.	PSI core	Photosystem I subunit H-1
At1g52230	PsaH-2	n.q.	−2.50 **	PSI core	Photosystem I subunit H-2
AtCg00510	PsaI	n.d.	n.d.	PSI core	Photosystem I subunit I
AtCg00630	PsaJ	n.d.	n.q.	PSI core	Photosystem I subunit J
At1g30380	PsaK	n.v.	−1.64 **	PSI core	Photosystem I subunit K
At4g12800	PsaL	−1.93 **	−1.77 **	PSI core	Photosystem I subunit L
At1g08380	Psa0	n.q.	−1.98 **	PSI core	Photosystem I subunit O
At4g02770	PsaD-1	−1.85 **	n.d.	PSI peripherical	Photosystem I subunit D-1
At1g03130	PsaD-2	**absent**	−1.92 **	PSI peripherical	Photosystem I subunit D-2
At4g28750	PsaE-1	−2.02 **	−2.01 **	PSI peripherical	Photosystem I subunit E-1
At2g20260	PsaE-2	n.v.	−1.00 **	PSI peripherical	Photosystem I subunit E-2
At5g64040	PsaN	−2.26 **	−2.13 **	PSI peripherical	Photosystem I subunit N
At2g46820	PsaP	n.v.	n.v.	PSI peripherical	Photosystem I subunit P
At3g54890	Lhca-1	n.v.	−0.41 *	PSI antenna	Photosystem I light harvesting complex gene 1
At3g61470	Lhca-2	n.v.	−1.00 **	PSI antenna	Photosystem I light harvesting complex gene 2
At1g61520	Lhca-3	−1.06 *	−1.13 **	PSI antenna	Photosystem I light harvesting complex gene 3
At3g47470	Lhca-4	n.v.	−0.49 **	PSI antenna	Photosystem I light harvesting complex gene 4
At1g45474	Lhca-5	n.v.	−2.02 *	PSI antenna	Photosystem I light harvesting complex gene 5
At1g19150	Lhca-6	n.v.	n.v.	PSI antenna	Photosystem I light harvesting complex gene 6
At2g44860	PSA2	n.d.	n.d.	PSI chaperone	Photosystem I assembly 2
At3g55250	PSA3	n.v.	n.v.	PSI chaperone	Photosystem I assembly 3
AtCg00360	Ycf3	n.q.	+0.27 *	PSI chaperone	Hypothetical chloroplast open reading frame 3
AtCg00520	Ycf4	n.v.	n.q.	PSI chaperone	Hypothetical chloroplast open reading frame 4
At1g22700	PYG7	n.v.	+0.49 *	PSI chaperone	Pale yellow green 7
At5g44650	Y3IP1	n.v.	−0.35 **	PSI chaperone	YCF3-interacting protein 1
At4g15510	PPD1	n.v.	n.v.	PSI chaperone	PsbP domain protein 1
AtCg00020	PsbA	n.v.	n.v.	PSII reaction center	Photosystem II subunit A
AtCg00680	PsbB	n.v.	n.v.	PSII reaction center	Photosystem II subunit B
AtCg00280	PsbC	+0.37 *	n.v.	PSII reaction center	Photosystem II subunit C
AtCg00270	PsbD	n.v.	n.v.	PSII reaction center	Photosystem II subunit D
AtCg00580	PsbE	n.v.	n.v.	PSII reaction center	Photosystem II subunit E
AtCg00570	PsbF	n.q.	n.d.	PSII reaction center	Photosystem II subunit F
AtCg00710	PsbH	n.v.	n.v.	PSII reaction center	Photosystem II subunit H
AtCg00080	PsbI	n.d.	n.d.	PSII reaction center	Photosystem II subunit I
AtCg00550	PsbJ	n.d.	n.d.	PSII reaction center	Photosystem II subunit J
AtCg00070	PsbK	n.d.	n.d.	PSII reaction center	Photosystem II subunit K
AtCg00560	PsbL	n.d.	n.v.	PSII reaction center	Photosystem II subunit L
AtCg00220	PsbM	n.d.	n.d.	PSII reaction center	Photosystem II subunit M
AtCg00700	PsbN	n.d.	n.d.	PSII reaction center	Photosystem II subunit N
AtCg00690	PsbT	n.d.	n.d.	PSII reaction center	Photosystem II subunit T
At2g30570	PsbW	n.d.	n.d.	PSII reaction center	Photosystem II subunit W
At1g44575	PSII-S	+1.03 **	+0.73 *	extrinsic	Photosystem II subunit S
At1g51400	PSII-5kD	n.d.	n.d.	extrinsic	Photosystem II-5kDa
At3g21055	PSII-T	n.q.	n.v.	extrinsic	Photosystem II subunit T
At3g55330	PPL1	+0.35 *	n.v.	extrinsic	Photosystem II subunit P-like 1
At2g39470	PPL2	n.v.	n.v.	extrinsic	Photosystem II subunit P-like 2
At1g76450	PsbP family	n.v.	n.v.	extrinsic	Photosystem II subunit PsbP family
At1g69680	PsbP family	n.d.	n.d.	extrinsic	Photosystem II subunit PsbP family
At1g77090	PsbP family	n.v.	n.v.	extrinsic	Photosystem II subunit PsbP family
At5g11450	PsbP family	−0.67 *	−0.41 *	extrinsic	Photosystem II subunit PsbP family
At3g05410	PsbP family	n.d.	n.d.	extrinsic	Photosystem II subunit PsbP family
At3g56650	PPD6	+0.69 *	n.v.	extrinsic	PsbB domain protein 6
At5g66570	PsbO-1	+0.29 *	n.v.	extrinsic	Photosystem II subunit O-1
At3g50820	PsbO-2	+0.31 **	n.v.	extrinsic	Photosystem II subunit O-2
At1g06680	PsbP-1	n.v.	n.v.	extrinsic	Photosystem II subunit P-1
At2g30790	PsbP-2	n.d.	n.d.	extrinsic	Photosystem II subunit P-2
At4g21280	PsQ-1	+0.44 *	n.v.	extrinsic	Photosystem II subunit Q-1
At4g05180	PsbQ-2	n.v.	n.v.	extrinsic	Photosystem II subunit Q-2
At1g79040	PsbR	n.v.	n.v.	extrinsic	Photosystem II subunit R
At2g06520	PsbX	n.d.	n.d.	extrinsic	Photosystem II subunit X
At1g67740	PsbY-1, 2	n.d.	n.d.	extrinsic	Photosystem II subunit Y
AtCg00300	PsbZ	n.d.	n.d.	extrinsic	Photosystem II subunit Z
At1g29920	Lhcb1.1	n.q.	n.d.	PSII antenna	Light harvesting chlorophyll A/B binding protein 1.1
At1g29910	Lhcb1.2	n.d.	n.d.	PSII antenna	Light harvesting chlorophyll A/B binding protein 1.2
At1g29930	Lhcb1.3	n.v.	n.v.	PSII antenna	Light harvesting chlorophyll A/B binding protein 1.3
At2g34430	Lhcb1.4	n.v.	n.v.	PSII antenna	Light harvesting chlorophyll A/B binding protein 1.4
At2g34420	Lhcb1.5	n.v.	n.v.	PSII antenna	Light harvesting chlorophyll A/B binding protein 1.5
At2g05100	Lhcb2.1	n.q.	n.d.	PSII antenna	Light harvesting chlorophyll A/B binding protein 2.1
At2g05070	Lhcb2.2	n.v.	−0.28 *	PSII antenna	Light harvesting chlorophyll A/B binding protein 2.2
At3g27690	Lhcb2.3	n.v.	n.d.	PSII antenna	Light harvesting chlorophyll A/B binding protein 2.3
At5g54270	Lhcb3	n.v.	n.v.	PSII antenna	Light harvesting chlorophyll A/B binding protein 3
At5g01530	Lhcb4.1	n.v.	n.v.	PSII antenna	Light harvesting chlorophyll A/B binding protein 4.1
At3g08940	Lhcb4.2	n.v.	n.v.	PSII antenna	Light harvesting chlorophyll A/B binding protein 4.2
At2g40100	Lhcb4.3	n.v.	+0.83 *	PSII antenna	Light harvesting chlorophyll A/B binding protein 4.3
At4g10340	Lhcb5	n.v.	n.v.	PSII antenna	Light harvesting chlorophyll A/B binding protein 5
At1g15820	Lhcb6	n.v.	n.v.	PSII antenna	Light harvesting chlorophyll A/B binding protein 6

log2 (FC), Logarithmic fold change; n.q., not quantifiable; n.v., no variation; n.d., not detected. Significant differences were assessed by *t*-test: *: *p* < 0.05, **: *p* < 0.01. *nfu2-1* data are from [21].

**Table 2:** Quantification of Fe-S proteins in the *Arabidopsis thaliana nfu3-2* mutant background.

ID	Acronym	log2 (FC) in *nfu3-2* Seedlings	log2 (FC) in *nfu2-1* Seedlings	Fe-S Cluster	Function	Name
At4g04610	APR1	n.d.	n.q.	4Fe4S	Sulfate assimilation	Adenosine 5’-phosphosulfate reductase 1
At1g62180	APR2	n.d.	n.q.	4Fe4S	Sulfate assimilation	Adenosine 5’-phosphosulfate reductase 2
At4g21990	APR3	n.d.	n.q.	4Fe4S	Sulfate assimilation	Adenosine 5’-phosphosulfate reductase 3
At2g16570	ASE1	n.d.	n.q.	4Fe4S	Purine nucleotide biosynthesis	Amidophosphoribosyltransferase 1
At4g34740	ASE2	−0.25 *	n.v.	4Fe4S	Purine nucleotide biosynthesis	Amidophosphoribosyltransferase 2
At4g38880	ASE3	n.d.	n.q.	4Fe4S	Purine nucleotide biosynthesis	Amidophosphoribosyltransferase 3
At1g44446	CAO	n.q.	n.q.	rieske 2Fe/2S	Chlorophyll biosynthesis	Chloropyll a oxygenase
At2g42750	CDJ1	n.d.	n.q.	4Fe4S	Unknown	DNA J protein C77
At5g23240	CDJ2	n.d.	n.q.	4Fe4S	Unknown	DNA J protein C76
At3g05345	CDJ3	n.d.	n.q.	4Fe4S	Unknown	DNA J protein C82
At4g29890	CMO	n.d.	n.q.	rieske 2Fe/2S	Glycine betaine biosynthesis	Choline monooxygenase (putative)
At3g23940	DHAD	n.v.	n.v.	2Fe2S	Branched chain amino acid biosynthesis	Dihydroxyacid dehydratase
At1g03055	DWARF27.1	n.d.	n.q.	4Fe4S (?)	Strigolactone biosynthesis	DWARF27.1
At1g64680	DWARF27.2	−1.05 *	n.q.	4Fe4S (?)	Unknown	DWARF27.2
At4g01995	DWARF27.3	n.d.	n.d.	4Fe4S (?)	Unknown	DWARF27.3
At1g10960	FD1 (PETF2)	n.v.	n.q.	2Fe2S	Electron transport chain	Ferredoxin 1
At1g60950	FD2 (PETF)	n.v.	n.q.	2Fe2S	Photosynthesis	Ferredoxin 2
At2g27510	FD3	n.q.	n.q.	2Fe2S	Electron transport chain	Ferredoxin 3
At5g10000	FD4	n.d.	n.q.	2Fe2S	Electron transport chain	Ferredoxin 4
At1g32550	FdC1	**absent**	n.q.	2Fe2S	Electron transport chain	Ferredoxin like 1
At4g14890	FdC2	**absent**	n.q.	2Fe2S	Electron transport chain	Ferredoxin like 2
At2g04700	FTR	n.v.	+0.31 **	4Fe4S	Ferredoxin-thioredoxin reductase	Ferredoxin-thioredoxin reductase
At1g04620	HCAR	**absent**	n.q.	2x [4Fe4S]	7-hydroxymethyl chlorophyll a (HMCHL) reductase	7-hydroxymethyl chlorophyll a (HMCHL) reductase
At3g24430	HCF101	−0.47 *	n.v.	4Fe4S	Fe-S cluster transfer	High clorophyll fluorescence 101
At5g04140	GLU1	+0.79 **	+0.83 **	3Fe4S	Nitrate assimilation	Glutamate synthase 1 (Fd-GOGAT)
At2g41220	GLU2	n.v.	n.q.	3Fe4S	Nitrate assimilation	Glutamate synthase 2 (Fd-GOGAT)
At5g53460	GLT1	−0.92 **	−0.60 **	3Fe4S	Nitrate assimilation	Glutamate synthase 1 (NADH-dependent)
At1g18500	IPMSI	n.v.	n.v.	3Fe4S	Leucine biosynthesis	Isopropylmalate synthase
At4g13430	IPMI LSU1	n.v.	n.v.	4Fe4S	Leucine biosynthesis	Isopropylmalate isomerase (large subunit 1)
At5g60600	ISPG	n.v.	n.v.	4Fe4S	Isoprenoid precursor biosynthesis	4-Hydroxy-2-methylbut-2-enyl diphosphate synthase
At4g34350	ISPH	n.v.	n.v.	4Fe4S	Isoprenoid precursor biosynthesis	4-Hydroxy-2-methylbut-2-enyl diphosphate reductase
At5g08415	cLIP1	n.d.	n.q.	2x [4Fe4S]	Lipoyl biosynthesis	Lipoyl synthase 1
At4g36390	MIAB	n.d.	n.q.	2x [4Fe4S]	t-RNA maturation	Methylthiotransferase
AtCg00430	NDHK	n.v.	n.v.	4Fe4S	Aerobic respiration	NADH dehydrogenase subunit
AtCg01090	NDHI	n.v.	n.v.	2x [4Fe4S]	Photosynthesis	NADH dehydrogenase subunit
At5g51720	NEET	n.v.	n.v.	2Fe2S	Iron homeostasis	NEET
At4g01940	NFU1	n.v.	n.q.	4Fe4S	Fe-S cluster transfer	NFU domain protein 1
At5g49940	NFU2	n.q.	n.q.	2Fe2S or 4Fe4S	Fe-S cluster transfer	NFU domain protein 2
At4g25910	NFU3	n.d.	n.q.	2Fe2S or 4Fe4S	Fe-S cluster transfer	NFU domain protein 3
At2g15620	NIR	−0.45 **	n.v.	4Fe4S	Nitrate assimilation	Nitrite reductase
At3g44880	PAO	+1.30 *	−0.25 *	rieske 2Fe/2S	Chlorophyll catabolism	Pheophorbide a oxygenase
At4g03280	PetC	−0.46 *	n.v.	rieske 2Fe/2S	Photosynthesis	Photosynthetic electron transfer C
AtCg00350	PsaA	−2.07 **	−1.68 **	4Fe4S	Photosynthesis	PsaA subunit of photosystem I
AtCg00340	PsaB	−1.84 **	−1.81 **	4Fe4S	Photosynthesis	PsaB subunit of photosystem I
AtCg01060	PsaC	−1.51 *	−2.02 **	4Fe4S	Photosynthesis	PsaC subunit of photosystem I
At1g71500	PSB33	+0.73 *	+0.45 **	2Fe2S #	Photosynthesis	Photosystem B protein 33
At4g25650	PTC52	+0.34 **	n.v.	rieske 2Fe/2S	Chlorophyll catabolism	Protochlorophyllide-dependent translocon component 52
At5g04590	SIR1	n.v.	n.v.	4Fe4S	Sulfate assimilation	Sulfite reductase
At1g50170	SIRB	n.q.	n.q.	4Fe4S	Siroheme biosynthesis	Sirohydrochlorin ferrochelatase B
At1g10500	SUFA1	n.d.	n.q.	4Fe4S	Fe-S cluster transfer	SUFA1
At4g04770	SUFB1	n.v.	n.q.	4Fe4S	Fe-S cluster assembly	SUFB1
At5g50210	SUFE3	n.d.	n.q.	4Fe4S	NAD biosynthesis	Sulfur E3
At2g29630	THIC	**absent**	n.q.	4Fe4S	Thiamin biosynthesis	Thiamin C
At2g24820	TIC55	+0.90 **	n.v.	rieske 2Fe/2S	Chloroplast Protein Import	Translocon at the inner envelope membrane of chloroplast 55

log2 (FC), Logarithmic fold change; absent, detected only in WT samples; n.q., not quantifiable; n.v., no variation; n.d., not detected. Significant differences were assessed by *t*-test: *: *p* < 0.05, **: *p* < 0.01. *nfu2-1* data are from [21].

## Supplementary Materials

The following are available online at https://www.mdpi.com/1422-0067/21/21/8121/s1, Figure S1. *Arabidopsis thaliana* NFU1, NFU2 and NFU3 protein sequences. Figure S2. Quantitative analysis of GFP fluorescence in *Arabidopsis thaliana* ProNFU:gNFU-GFP transgenic lines. Table S1. List of significantly varying proteins in the *Arabidopsis thaliana nfu3-2* mutant relative to wild type. Table S2. List of proteins specifically present in the *Arabidopsis thaliana nfu3-2* mutant or in the wild type. Table S3. Detailed localization of variant proteins. Table S4: Detailed localization of proteins under-accumulated specifically in *nfu3-2,* or in *nfu2-1* or in both mutants. Table S5: Detailed localization of proteins over-accumulated specifically in *nfu3-2,* or in *nfu2-1* or in both mutants. Table S6. Primers used in this study.
