# Supplementary material for: A Global Proteomic Approach Sheds New Light on Potential Iron-Sulfur Client Proteins of the Chloroplastic Maturation Factor NFU3
Source: Int J Mol Sci. 2020 Oct 30;21(21):8121. doi: 10.3390/ijms21218121 (PMC7672563; doi:10.3390/ijms21218121)
Supplement: Supplementary file 1 [file ijms-21-08121-s001.zip › ijms-973833 final suppl/Berger_et_al_IJMS_Figure_S1_vIII.pdf]

A

```
NFU1      MMASLATSISGSFRILVKSSS-----------------TRNGFPVISDQNPSFV------  37
NFU2      ------------MQLLTLNPAAISRTPPQAIDPSSSSSLLLPFPQILSSQRALGLVARPC  48
NFU3      -----MGSVSGQTRITTMNLSL-S---TAEKNPNFRSSLLNSKNA---ISDTLGV-SSKC  47
                       ::     :                              ::

NFU1      --LFANKRRHISRTAIFHRSAISGSSQGEKISPLASGVSSGLYSAQTFDLTPQNVDLVLE  95
NFU2      NPLRRGLSRFLSSRQLFRRSKVV------------KAVATP-DPILEVPLTEENVESVLD  95
NFU3      STFLRGQFQRIHFSWLQHTRPLR------------KRTVFG-HVSCVMPLTEENVERVLD  94
            :     : :    : :   :                           ** :**: **:

NFU1      DVRPFLISDGGNVDVVSVEDGVVSLKLQGACTSCPSSSTTMTMGIERVLKEKFGDALKDI  155
NFU2      EIRPYLMSDGGNVALHEIDGNIVRVKLQGACGSCPSSTMTMKMGIERRLMEKIPEIVA-V  154
NFU3      EVRPSLMADGGNVALHEIDGLVVVLKLQGACGSCPSSSMTLKMGIESRLRDKIPEIMS-V  153
          ::** *::***** :  :: :* :****** *****: *: ****  * :*: : :  :

NFU1      RQVFDEEVKQ--ITVEAVNAHLDILRPAIK-NYGGSVEVLSVEGEDCVVKYVGPESIGMG  212
NFU2      EALPD-EETGLELNEENIEKVLEEIRPYLIGTADGSLDLVEIEDPIVKIRITGPAAGVMT  213
NFU3      EQFLESETGGLELNDENIEKVLSELRPYLSGTGGGGLELVEIDGYVVKVRLTGPAAGVMT  213
              : *     :  * ::  *  :** :     * :::: ::     ::  ** :  *

NFU1      IQAAIKEKFKD----ISNVTFTS       231
NFU2      VRVAVTQKLREKIPSIAAVQLI-       235
NFU3      VRVALTQKLRETIPSIGAVQLLE       236
          :: *: :*:::    *  *  :
```

B

	NFU1	NFU2	NFU3
**NFU1**		29%	31%
**NFU2**	50%		54%
**NFU3**	48%	67%	

Proteins sequence identity (pink cells)

Proteins sequence similarity (green cells)

Figure S1. ***Arabidopsis thaliana*** **NFU1, NFU2 and NFU3 protein sequences. (A)** NFU1, NFU2 and NFU3 protein sequences alignment. Protein sequences were obtained from TAIR (https://www.arabidopsis.org/) and were aligned using Clustal Omega Multi Sequence Alignment software (https://www.ebi.ac.uk/Tools/msa/clustalo/). *, identical amino acid in the three isoforms; :, similar amino acids in the three isoforms. Numbers indicate the amino acid length of the three NFU isoforms. **(B)** Overall sequence identity and similarity between the three NFU isoforms. Values were obtained using the BLAST Global Alignment tool (https://blast.ncbi.nlm.nih.gov/Blast.cgi).
