# Supplementary material for: A Global Proteomic Approach Sheds New Light on Potential Iron-Sulfur Client Proteins of the Chloroplastic Maturation Factor NFU3
Source: Int J Mol Sci. 2020 Oct 30;21(21):8121. doi: 10.3390/ijms21218121 (PMC7672563; doi:10.3390/ijms21218121)
Supplement: Supplementary file 1 [file ijms-21-08121-s001.zip › ijms-973833 final suppl/Berger_et_al_IJMS_Figure_S2_vIII.pdf]

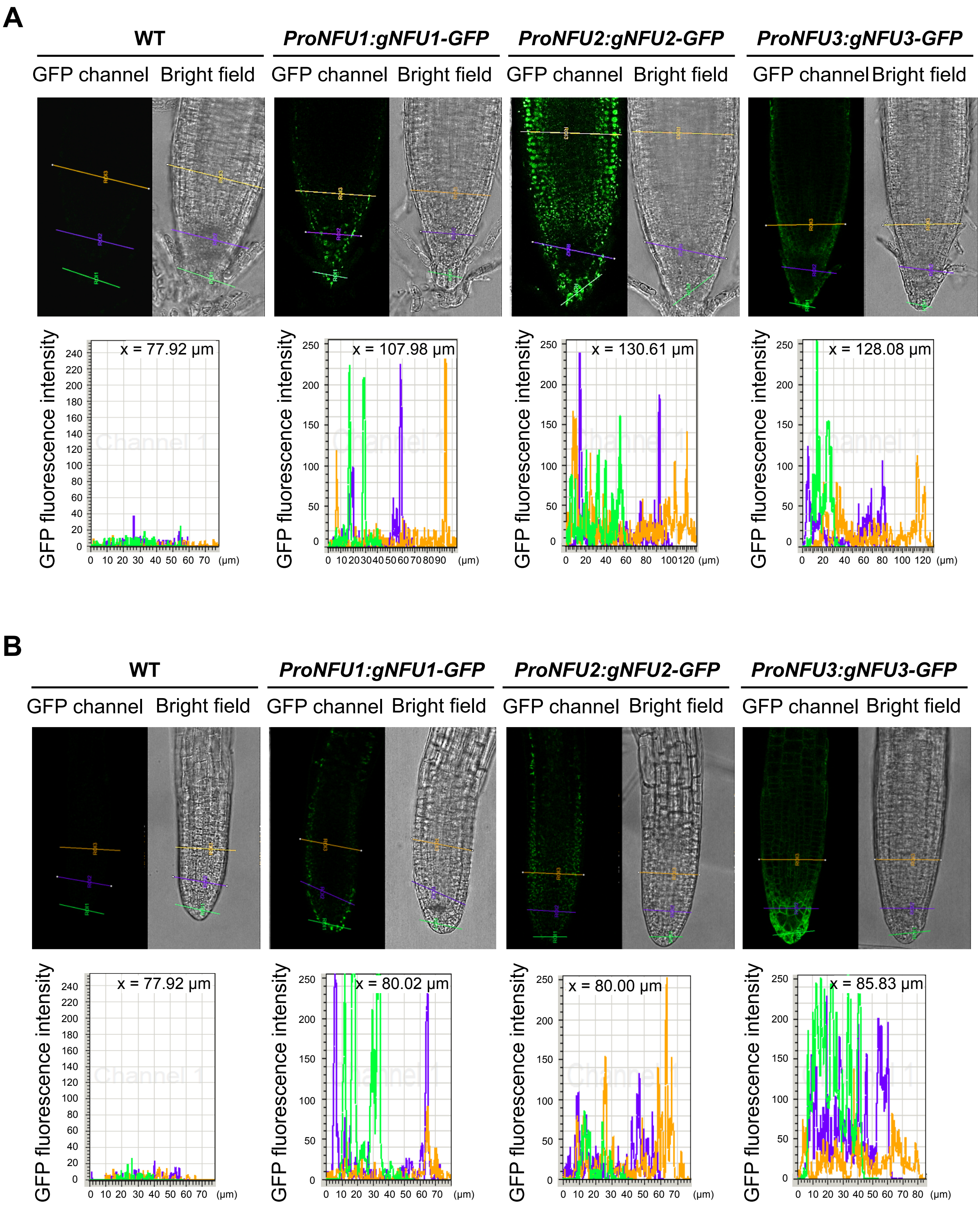

Figure S2. Quantitative analysis of GFP fluorescence in Arabidopsis *ProNFU:gNFU-GFP* transgenic lines. The fluorescence of NFU-GFP fusion proteins was recorded along cross sections at the root tip (green lines), and upper regions (purple and orange lines) of primary **(A)** and secondary **(B)** roots. Top panels show GFP fluorescence and bright field acquisitions, bottom panels show GFP fluorescence intensities along cross sections.
