# Supplementary material for: A Global Proteomic Approach Sheds New Light on Potential Iron-Sulfur Client Proteins of the Chloroplastic Maturation Factor NFU3
Source: Int J Mol Sci. 2020 Oct 30;21(21):8121. doi: 10.3390/ijms21218121 (PMC7672563; doi:10.3390/ijms21218121)
Supplement: Supplementary file 1 [file ijms-21-08121-s001.zip › ijms-973833 final suppl/Berger_et_al_IJMS_Table_S1_vIII.pdf]

Table S1: List of significant variant proteins between WT and *nfu3-2* plants

Protein IDs	Fasta headers	mean_col	mean_nfu3	log2 (FC)	LFQ intensity col-1	LFQ intensity col-2	LFQ intensity col-3	LFQ intensity nfu3-1	LFQ intensity nfu3-2	LFQ intensity nfu3-3	number of peptides	Number of razor + unique peptides	Number of unique peptides	MS/MS count	-Log Student's T-test p-value	Student's T-test Test statistic	Sequence coverage [%]
AT3G01420	ALPHA-DOX1, DOX1, DIOX1, PADOX-1 Peroxidase superfamily protein	29.16	25.38	**-3.78**	29.41	28.88	29.19	23.61	26.23	26.3	29	29	29	82	1.86	-4.2	54.8
AT1G05510	Protein of unknown function (DUF1264)	28.87	25.22	**-3.65**	28.85	28.9	28.86	25.57	25.28	24.81	9	9	9	45	4.09	-16.38	55.2
AT5G52310	COR78, LTI78, RD29A, LTI140 low-temperature-responsive protein 78 (LTI78) / desiccation-responsive protein 29A (RD29A	27.19	24.58	**-2.61**	26.93	26.64	27.99	25.32	24.1	24.32	14	14	14	30	2.02	-4.68	45.5
AT1G55020	LOX1, ATLOX1 lipoxygenase 1	29.28	26.68	**-2.6**	29.04	29.42	29.38	25.96	27.06	27.02	40	40	40	159	2.63	-6.86	53.6
AT5G64040	PSAN photosystem I reaction center subunit PSI-N, chloroplast, pu	33.49	31.22	**-2.26**	33.12	33.66	33.68	30.35	31.55	31.76	15	15	15	340	2.05	-4.75	42.7
AT2G39310	JAL22 jacalin-related lectin 22 |	29.23	26.99	**-2.24**	29.42	29.1	29.16	27.49	26.37	27.11	24	24	20	103	2.55	-6.55	61.6
ATCG00350	PSAA Photosystem I, PsaA/PsaB protein	35.22	33.14	**-2.08**	35.49	35.06	35.1	32.76	33.35	33.32	27	27	27	611	3.05	-8.85	28.9
AT4G28750	PSAE-1 Photosystem I reaction centre subunit IV / PsaE protein	32.57	30.56	**-2.02**	32.34	32.75	32.63	30.41	30.57	30.69	10	6	6	178	3.81	-13.89	60.8
AT4G12800	PSAL photosystem I subunit l	33.43	31.5	**-1.93**	33.31	33.64	33.34	31.23	31.7	31.55	9	9	9	277	3.42	-11.03	27.9
AT4G02770	PSAD-1 photosystem I subunit D-1	34.52	32.67	**-1.85**	34.26	34.62	34.7	32.76	32.62	32.63	18	18	2	656	3.71	-13.08	69.2
ATCG00340	PSAB Photosystem I, PsaA/PsaB protein	34.54	32.7	**-1.84**	34.79	34.42	34.41	32.52	32.76	32.82	20	20	20	535	3.52	-11.76	30.8
AT4G23680	Polyketide cyclase/dehydrase and lipid transport superfamily protein	30.02	28.21	**-1.81**	29.95	29.97	30.15	28.53	27.98	28.11	15	13	10	152	3.28	-10.16	69.5
AT5G02490	ERD2, HSP70T-1 heat shock protein 70 (Hsp 70) family protein	27.71	25.99	**-1.72**	28.2	27.39	27.54	25.51	26.02	26.45	37	5	5	24	2.03	-4.69	47.9
AT3G46780	PTAC16 plastid transcriptionally active 16	32.98	31.29	**-1.69**	33	33.02	32.91	31.41	31.19	31.26	39	39	39	552	4.67	-22.93	65.5
AT1G65010	Plant protein of unknown function (DUF827)	28.6	26.96	**-1.63**	28.99	28.27	28.54	27.45	26.29	27.15	32	32	30	94	1.8	-4.02	33.8
AT1G31330	PSAF photosystem I subunit F	34.85	33.22	**-1.63**	34.8	34.85	34.9	33.41	33.08	33.17	11	11	11	440	4.02	-15.76	47.1
AT4G27440	PORB protochlorophyllide oxidoreductase B	30.68	29.13	**-1.54**	30.76	30.61	30.66	29.48	28.9	29.02	25	21	21	158	2.97	-8.44	71.6
AT1G29660	GDSL-like Lipase/Acylhydrolase superfamily protein	28.85	27.31	**-1.54**	28.86	28.85	28.85	26.98	27.58	27.38	10	10	10	71	3.03	-8.76	46.7
ATCG01060	PSAC iron-sulfur cluster binding;electron carriers;4 iron, 4 sulfur cluster binding	32.39	30.87	**-1.51**	31.51	32.83	32.81	30.66	30.8	31.16	9	9	9	256	1.51	-3.28	91.4
AT4G24510	CER2, VC2, VC-2 HXXXD-type acyl-transferase family protein	28.64	27.15	**-1.49**	28.83	28.55	28.55	26.79	27.4	27.26	11	11	11	78	2.7	-7.17	35.2
AT5G45950	GDSL-like Lipase/Acylhydrolase superfamily protein	28.29	26.82	**-1.47**	28.35	28.38	28.15	27	26.77	26.7	9	9	9	45	3.62	-12.46	30
AT3G16140	PSAH-1 photosystem I subunit H-1	31.8	30.38	**-1.42**	31.46	31.89	32.04	30.72	30.08	30.33	8	8	2	189	2.29	-5.56	56.6
AT5G01600	ATFER1, FER1 ferretin 1	29.23	27.82	**-1.41**	29.51	29.18	29.02	28.26	27.66	27.54	12	12	11	67	2.22	-5.32	47.1
AT4G00400	GPAT8, AtGPAT8 glycerol-3-phosphate acyltransferase 8	28.06	26.67	**-1.39**	28.29	28.03	27.85	26.81	26.56	26.65	21	21	9	76	3.15	-9.43	44.4
AT4G25080	CHLM magnesium-protoporphyrin IX methyltransferase	30.42	29.06	**-1.35**	30.55	30.34	30.37	29.58	28.76	28.86	21	21	21	187	2.15	-5.07	76.3
AT2G35880	TPX2 (targeting protein for Xklp2) protein family	25.67	24.32	**-1.35**	25.99	25.5	25.51	24.21	24.27	24.47	8	8	8	26	2.76	-7.46	21.5
AT2G38750	ANNAT4 annexin 4	28.26	26.92	**-1.34**	28.57	28.18	28.02	27.46	26.6	26.68	12	12	12	69	1.87	-4.21	40.1
AT1G24020	MLP423 MLP-like protein 423	28.72	27.39	**-1.33**	28.47	28.84	28.85	27.6	27.27	27.3	10	10	10	81	2.89	-8.04	66.5
AT5G25460	Protein of unknown function, DUF642	30.18	28.85	**-1.33**	30.7	29.78	30.05	29.18	28.8	28.57	17	17	11	148	1.82	-4.07	53.9
AT1G09310	Protein of unknown function, DUF538	31.46	30.14	**-1.33**	31.47	31.48	31.43	30.7	29.84	29.87	16	16	15	213	2.04	-4.72	78.8
AT1G45201	ATTLL1, TLL1 triacylglycerol lipase-like 1	28.98	27.66	**-1.31**	29.11	28.98	28.84	27.87	27.57	27.55	11	11	11	62	3.25	-9.98	28.2
AT4G04340	ERD (early-responsive to dehydration stress) family protein	25.74	24.43	**-1.31**	25.88	25.71	25.63	24.35	24.17	24.78	6	6	5	19	2.58	-6.67	8.9
AT5G43060	Granulin repeat cysteine protease family protein	28.5	27.2	**-1.3**	28.86	28.42	28.22	26.81	27.52	27.26	8	8	8	42	2.01	-4.64	21.6
AT5G65020	ANNAT2 annexin 2	29.42	28.12	**-1.29**	29.62	29.2	29.43	28.28	28.13	27.96	22	22	22	123	2.97	-8.44	71
AT1G10670	ACLA-1 ATP-citrate lyase A-1	28.13	26.86	**-1.27**	27.95	28.16	28.29	27.05	26.82	26.72	16	12	12	51	3.11	-9.2	44.2
AT3G12110	ACT11 actin-11	26.15	24.89	**-1.27**	25.54	26.33	26.59	25.42	24.81	24.44	23	4	2	28	1.39	-2.97	65.3
AT2G38380	Peroxidase superfamily protein	27.25	26.01	**-1.24**	27.74	26.8	27.21	25.99	26.19	25.85	7	5	5	36	1.9	-4.3	19.5
AT3G14220	GDSL-like Lipase/Acylhydrolase superfamily protein	27.13	25.89	**-1.23**	26.99	27.12	27.26	25.69	25.94	26.05	8	8	7	41	3.16	-9.46	28.9
AT2G04280	unknown protein	24.49	23.29	**-1.2**	24.54	24.64	24.28	23.15	22.89	23.82	6	6	6	16	1.8	-4.01	10.6
AT4G10480	Nascent polypeptide-associated complex (NAC), alpha subunit family protein	29.26	28.09	**-1.17**	29.53	29.2	29.05	28.07	28.08	28.13	10	10	9	100	2.92	-8.2	69.3
AT5G24650	Mitochondrial import inner membrane translocase subunit Tim17/Tim22/Tim23 family protein	28.1	26.93	**-1.17**	28.3	28.17	27.82	26.49	26.95	27.34	7	7	7	37	1.83	-4.11	37.5
AT5G54770	THI1, TZ, THI4 thiazole biosynthetic enzyme, chloroplast (ARA6) (THI1) (THI4)	29.93	28.76	**-1.17**	30.21	29.79	29.78	29.3	28.26	28.72	11	11	11	149	1.6	-3.49	47.3
AT2G05710	ACO3 aconitase 3	32.84	31.68	**-1.16**	32.94	32.71	32.88	31.93	31.52	31.6	48	48	36	813	2.91	-8.15	62.7
AT5G27560	Domain of unknown function (DUF1995)	25.71	24.56	**-1.15**	26.07	25.71	25.34	24.86	24.11	24.71	4	4	4	21	1.67	-3.67	16.4
AT1G77760	NIA1, GNR1, NR1 nitrate reductase 1	28.85	27.7	**-1.14**	28.98	28.68	28.88	27.53	27.92	27.67	37	31	31	124	2.87	-7.95	42.9
AT3G08030	Protein of unknown function, DUF642	30.39	29.26	**-1.13**	30.57	30.22	30.39	29.23	29.3	29.25	18	18	18	141	3.37	-10.73	75.9
AT2G45540	WD-40 repeat family protein / beige-related	27.14	26.02	**-1.11**	27.19	27.09	27.12	26.47	25.28	26.33	30	30	30	73	1.38	-2.96	16.6
AT5G07030	Eukaryotic aspartyl protease family protein	30.22	29.14	**-1.08**	30.34	30.09	30.24	29.38	28.94	29.1	18	18	18	158	2.74	-7.37	51.6
AT3G27530	GC6, MAG4 golgin candidate 6	26.72	25.64	**-1.08**	26.46	26.83	26.88	25.59	25.55	25.79	15	15	15	37	2.67	-7.07	25.2
AT4G05390	RFNR1 root FNR 1	29.18	28.11	**-1.07**	29.29	29.14	29.12	27.78	28.41	28.15	14	14	8	76	2.31	-5.63	42.9
AT1G61520	LHCA3 photosystem I light harvesting complex gene 3	34.45	33.39	**-1.06**	34.6	34.37	34.38	33.94	33.15	33.1	7	7	7	411	1.7	-3.74	34.4
AT3G16450	Mannose-	30.33	29.28	**-1.05**	30.64	30.15	30.22	29.56	29.12	29.17	21	21	21	168	2.14	-5.06	76.3
AT1G64680	unknown protein	25.97	24.92	**-1.05**	26.06	25.58	26.26	24.69	25	25.07	4	4	4	20	1.95	-4.47	17.2
AT1G70940	PIN3, ATPIN3 Auxin efflux carrier family protein	26.23	25.18	**-1.05**	26.68	25.99	26.01	25.29	24.94	25.31	8	8	7	30	1.82	-4.07	20.6
AT5G16590	LRR1 Leucine-rich repeat protein kinase family protein	27.15	26.11	**-1.04**	27.43	26.94	27.08	26.04	26.03	26.27	15	12	12	66	2.48	-6.26	31.2
AT3G49720	unknown protein	27.7	26.68	**-1.03**	27.91	27.61	27.6	26.58	26.7	26.74	10	10	9	66	3.11	-9.18	52.1
AT4G37040	MAP1D methionine aminopeptidase 1D	25.82	24.8	**-1.02**	26.32	25.42	25.74	24.57	24.86	24.97	4	4	4	20	1.62	-3.54	16.3
AT5G65110	ACX2, ATACX2 acyl-CoA oxidase 2	25.54	24.52	**-1.02**	25.75	25.54	25.32	24.51	24.71	24.33	12	12	12	27	2.46	-6.18	24.3
AT3G56940	CRD1 dicarboxylate diiron protein, putative (Crd1)	31.21	30.2	**-1.01**	31.44	31.12	31.07	30.54	30.09	29.98	25	25	25	315	2.09	-4.88	59.9
AT1G06000	UDP-Glycosyltransferase superfamily protein	27.57	26.57	**-1**	27.28	27.79	27.63	26.93	26.24	26.53	15	15	15	53	1.78	-3.97	47.6
AT1G20330	SMT2, CVP1, FRL1 sterol methyltransferase 2	27.23	26.24	**-0.99**	27.52	27.28	26.9	26.65	26.02	26.06	14	14	12	64	1.67	-3.66	49.9
AT5G18660	PCB2 NAD(P)-binding Rossmann-fold superfamily protein	28.45	27.46	**-0.99**	28.58	28.28	28.49	27.68	27.3	27.4	14	14	14	52	2.64	-6.93	51.1
AT3G16400	ATMLP-470, NSP1, ATNSP1 nitrile specifier protein 1	32.53	31.54	**-0.99**	32.74	32.42	32.43	31.38	31.68	31.56	34	34	14	638	2.73	-7.31	73
AT2G40300	ATFER4, FER4 ferritin 4	28.55	27.56	**-0.99**	28.79	28.49	28.37	27.99	27.48	27.22	11	11	11	55	1.73	-3.84	51
AT1G03630	POR C, PORC protochlorophyllide oxidoreductase C	31.23	30.26	**-0.97**	31.17	31.24	31.28	30.72	29.88	30.17	25	25	21	265	1.76	-3.9	71.7
AT4G19490	ATVPS54, VPS54 VPS54	25.52	24.55	**-0.97**	25.41	25.55	25.6	24.81	24.16	24.69	14	14	14	22	2.03	-4.69	20
AT3G15950	NAI2 DNA topoisomerase-related	34.01	33.06	**-0.95**	34.06	33.97	34.02	33.08	33.07	33.04	85	85	84	1329	5.31	-33.21	82.6
AT3G14650	CYP72A11 cytochrome P450, family 72, subfamily A, polypeptide 11	25.41	24.46	**-0.95**	25.87	25.19	25.18	24.42	24.89	24.09	7	7	3	23	1.36	-2.91	15.8

AT4G31830	unknown protein	25.76	24.81	**-0.95**	25.27	25.88	26.12	24.5	24.84	25.09	5	5	5	23	1.45	-3.11	41
AT4G30190	HA2 H(+)-ATPase 2	30.51	29.57	**-0.94**	30.69	30.3	30.53	29.37	29.6	29.73	68	23	21	113	2.47	-6.22	56.2
AT4G36220	FAH1, CYP84A1 ferulic acid 5-hydroxylase 1	27.43	26.49	**-0.94**	27.4	27.36	27.52	26.71	26.14	26.62	14	14	14	51	2.15	-5.07	31.9
AT4G37800	XTH7 xyloglucan endotransglucosylase/hydrolase 7	30.05	29.12	**-0.94**	30.21	29.93	30.03	29.43	28.76	29.17	14	14	14	148	1.94	-4.42	57.7
AT3G29360	UDP-glucose 6-dehydrogenase family protein	29.08	28.15	**-0.93**	29.17	28.94	29.12	28.34	28	28.11	19	19	8	128	2.79	-7.57	51.2
AT5G53460	GLT1 NADH	33.78	32.86	**-0.92**	34.07	33.57	33.71	32.63	33.01	32.94	132	132	132	977	2.09	-4.87	66.3
AT5G64080	Bifunctional inhibitor/lipid-transfer protein/seed storage 2S albumin	28.46	27.56	**-0.91**	28.83	28.42	28.13	27.73	27.49	27.45	3	3	3	30	1.84	-4.14	13.5
AT3G48870	HSP93-III Clp ATPase	29.94	29.03	**-0.9**	29.86	30.01	29.93	29.31	28.76	29.03	62	19	19	189	2.25	-5.41	68
AT4G18480	CHLI1, CH42, CH-42, CHL11, CHLI-1 P-loop containing nucleoside triphosphate hydrolases superfamily protein	32.11	31.21	**-0.9**	32.2	32.02	32.1	31.46	31.09	31.08	26	26	16	362	2.6	-6.74	49.3
AT1G52100	Mannose-binding lectin superfamily protein	31.56	30.68	**-0.89**	31.68	31.53	31.48	30.8	30.64	30.59	32	32	32	406	3.26	-10.07	64.5
AT5G23860	TUB8 tubulin beta 8	28.26	27.37	**-0.88**	28.25	28.22	28.31	27.82	26.98	27.33	31	6	3	60	1.66	-3.64	79.3
AT1G67730	YBR159, KCR1, ATKCR1 beta-ketoacyl reductase 1	28.54	27.68	**-0.87**	28.87	28.34	28.42	28.15	27.42	27.46	13	13	13	66	1.41	-3.02	52.8
AT2G31390	pfkB-like carbohydrate kinase family protein	28.27	27.41	**-0.86**	28.77	28.03	28	27.54	27.31	27.37	14	14	13	75	1.51	-3.27	68
AT3G24590	PLSP1 plastidic type i signal peptidase 1	25.29	24.43	**-0.85**	25.44	25.23	25.2	24.07	24.62	24.61	6	6	6	14	1.91	-4.33	35.1
AT4G39330	ATCAD9, CAD9 cinnamyl alcohol dehydrogenase 9	31.41	30.56	**-0.85**	31.58	31.37	31.29	31	30.35	30.34	22	22	22	289	1.65	-3.61	66.1
AT5G42020	BIP Heat shock protein 70 (Hsp 70) family protein	31.85	31.01	**-0.84**	32.15	31.74	31.66	30.82	31.04	31.17	51	49	5	543	1.99	-4.57	63.3
AT3G26900	SKL1, ATSKL1 shikimate	27.55	26.72	**-0.83**	27.75	27.58	27.32	27.03	26.34	26.8	10	10	10	62	1.6	-3.49	50
AT4G36250	ALDH3F1 aldehyde dehydrogenase 3F1	28.33	27.5	**-0.83**	27.96	28.59	28.44	27.65	27.34	27.52	11	11	11	45	1.78	-3.96	31.6
AT4G01850	SAM-2, MAT2, SAM2, AtSAM2 S-adenosylmethionine synthetase 2	31.31	30.49	**-0.82**	31.47	31.19	31.28	30.61	30.44	30.42	31	15	11	218	2.93	-8.24	87.3
AT2G20190	ATCLASP, CLASP CLIP-associated protein	27.17	26.36	**-0.82**	26.86	27.43	27.22	26.7	26.03	26.34	23	23	23	62	1.49	-3.21	22.5
AT4G14960	TUA6 Tubulin/FtsZ family protein	33.19	32.37	**-0.82**	33.24	33.14	33.18	32.55	32.35	32.2	31	31	2	698	2.85	-7.89	69.1
AT1G08520	ALB1, ALB-1V, V157, PDE166, CHLD ALBINA 1	31.25	30.43	**-0.82**	31.3	31.21	31.23	30.53	30.4	30.37	44	44	44	340	3.87	-14.43	55.9
AT1G29470	S-adenosyl-L-methionine-dependent methyltransferases superfamily protein	28.08	27.27	**-0.81**	28.32	28.02	27.89	27.33	27.34	27.14	29	29	22	94	2.32	-5.68	46.8
AT3G17390	MTO3, SAMS3, MAT4 S-adenosylmethionine synthetase family protein	33.88	33.08	**-0.79**	34.04	33.79	33.8	33.14	33.07	33.04	35	35	19	755	3.05	-8.87	90.8
AT4G24190	SHD, HSP90.7, AtHsp90.7, AtHsp90-7 Chaperone protein htpG family protein	31.62	30.83	**-0.79**	31.6	31.61	31.64	30.94	30.76	30.78	58	57	56	528	3.77	-13.56	69.7
AT1G78060	Glycosyl hydrolase family protein	28.05	27.27	**-0.79**	28.18	28.11	27.87	27.38	27.13	27.3	20	20	20	80	2.59	-6.7	39.8
AT3G16460	Mannose-binding lectin superfamily protein	33.03	32.25	**-0.78**	32.94	33.16	32.99	32.3	32.34	32.09	35	35	35	751	2.8	-7.65	52.5
AT1G12000	Phosphofructokinase family protein	28.05	27.27	**-0.78**	28.21	27.92	28.04	27.12	27.39	27.31	22	22	17	89	2.59	-6.71	47.2
AT4G30950	FAD6, FADC, SFD4 fatty acid desaturase 6	29.24	28.46	**-0.78**	29.47	29.02	29.22	28.47	28.43	28.47	12	12	12	85	2.39	-5.9	28.6
AT3G16420	PBP1, JAL30 PYK10-binding protein 1 c	34.32	33.55	**-0.78**	34.4	34.22	34.36	33.61	33.44	33.59	22	22	17	798	3.25	-9.99	85.9
AT4G37870	PCK1, PEPCK phosphoenolpyruvate carboxykinase 1	28.03	27.26	**-0.77**	27.97	28.01	28.12	27.32	27.57	26.89	22	22	22	79	1.72	-3.81	51.6
AT4G39980	DHS1 3-deoxy-D-arabino-heptulosonate 7-phosphate synthase 1	25.83	25.06	**-0.77**	25.64	25.95	25.89	25.55	24.81	24.8	13	8	8	19	1.36	-2.91	27
AT2G26250	FDH, KCS10 3-ketoacyl-CoA synthase 10	27.5	26.73	**-0.77**	27.6	27.53	27.37	26.76	26.59	26.83	16	16	16	98	2.84	-7.8	34.2
AT5G19940	Plastid-lipid associated protein PAP / fibrillin family protein	29.81	29.04	**-0.77**	29.71	29.89	29.84	29.3	28.86	28.96	16	16	16	94	2.22	-5.32	62.3
AT3G17820	ATGSKB6, GLN1.3, GLN1;3 glutamine synthetase 1.3	29.61	28.84	**-0.76**	29.65	29.58	29.59	29.23	28.64	28.65	15	10	9	46	1.75	-3.87	57.9
AT5G11420	Protein of unknown function, DUF642	28.37	27.62	**-0.75**	28.47	28.21	28.42	27.83	27.54	27.49	14	8	8	54	2.32	-5.66	35.2
AT1G69830	ATAMY3, AMY3 alpha-amylase-like 3	29.79	29.05	**-0.74**	29.91	29.83	29.65	29.39	28.91	28.85	38	38	38	196	1.8	-4.01	49.3
AT5G61790	CNX1, ATCNX1 calnexin 1	30.55	29.8	**-0.74**	30.72	30.47	30.45	29.89	29.89	29.63	25	25	18	246	2.38	-5.89	49.1
AT3G28860	ATMDR1, ATMDR11, PGP19, MDR11, MDR1, ATPGP19, ABCB19, ATABCB19 ATP binding cassette subfamily B19	27.23	26.49	**-0.74**	27.6	26.95	27.16	26.57	26.27	26.64	24	21	21	54	1.53	-3.31	25.6
AT1G13110	CYP71B7 cytochrome P450, family 71 subfamily B, polypeptide 7	27.18	26.44	**-0.74**	27.13	27.24	27.18	26.03	26.65	26.66	14	14	12	45	1.6	-3.5	35.9
AT5G22440	Ribosomal protein L1p/L10e family	26.41	25.67	**-0.73**	26.34	26.42	26.46	25.48	25.76	25.77	11	3	3	18	2.72	-7.25	47.5
AT3G13070	CBS domain-containing protein / transporter associated domain-containing protein	25.28	24.55	**-0.73**	25.66	24.84	25.33	24.5	24.67	24.47	7	7	7	18	1.37	-2.94	12.1
AT3G55620	emb1624 Translation initiation factor IF6	26.89	26.17	**-0.72**	27.1	26.8	26.76	26.25	26	26.25	6	6	6	28	2.24	-5.39	40
AT1G62380	ACO2, ATACO2 ACC oxidase 2	30.82	30.11	**-0.71**	31.04	30.64	30.78	30.19	30.19	29.94	16	16	15	252	2.11	-4.95	71.2
AT5G64940	ATATH13, ATH13, ATOSA1, OSA1 ABC2 homolog 13	27.43	26.73	**-0.71**	27.2	27.68	27.43	26.7	26.9	26.58	16	16	16	57	1.88	-4.26	23
AT1G02500	SAM1, SAM-1, MAT1, AtSAM1 S-adenosylmethionine synthetase 1	29.42	28.71	**-0.71**	29.63	29.39	29.23	28.87	28.71	28.56	30	9	9	106	2.07	-4.84	87
AT2G35630	MOR1, GEM1 ARM repeat superfamily protein	26.16	25.46	**-0.7**	26.52	26.03	25.93	25.39	25.46	25.51	16	16	16	27	1.72	-3.79	12.2
AT4G33030	SQD1 sulfoquinovosyldiacylglycerol 1	29.11	28.4	**-0.7**	29.23	28.96	29.12	28.8	28.34	28.07	18	18	18	119	1.43	-3.07	41.5
AT5G25100	Endomembrane protein 70 protein family	29.09	28.39	**-0.7**	29.46	28.91	28.91	28.33	28.37	28.48	23	23	10	119	1.67	-3.68	27.5
AT3G53710	AGD6 ARF-GAP domain 6	25.03	24.34	**-0.69**	25.17	24.82	25.09	24.19	24.17	24.64	6	6	4	27	1.68	-3.7	14.8
AT2G06850	EXGT-A1, EXT, XTH4 xyloglucan endotransglucosylase/hydrolase 4	30.16	29.48	**-0.69**	30.47	29.87	30.15	29.7	29.32	29.4	17	17	17	154	1.54	-3.34	56.8
AT5G58250	unknown protein	30.24	29.55	**-0.69**	30.24	30.35	30.12	30	29.31	29.33	9	9	9	104	1.36	-2.92	53.6
AT5G42950	GYF domain-containing protein	26.69	26	**-0.69**	26.91	26.33	26.83	26.26	25.74	26.01	24	24	24	56	1.37	-2.94	21.4
AT4G17520	Hyaluronan / mRNA binding family	27.76	27.07	**-0.69**	27.91	27.59	27.77	27.01	27.08	27.12	19	16	16	114	2.67	-7.03	59.2
AT5G27450	MVK, MK mevalonate kinase	25.11	24.43	**-0.68**	24.78	25.19	25.35	24.39	24.25	24.64	6	6	6	25	1.55	-3.36	16.9
AT2G36880	MAT3 methionine adenosyltransferase 3	31.93	31.25	**-0.68**	32.18	31.75	31.87	31.44	31.25	31.07	35	22	19	324	1.82	-4.08	90.5
AT4G18440	L-Aspartase-like family protein	26.2	25.53	**-0.67**	26.41	25.94	26.23	25.42	25.77	25.39	10	10	5	32	1.66	-3.65	25
AT5G08280	HEMC hydroxymethylbilane synthase	32.18	31.51	**-0.67**	32.26	32.12	32.16	31.53	31.45	31.55	29	29	29	375	3.65	-12.65	72.3
AT5G11450	Mog1/PsbP/DUF1795-like photosystem II reaction center PsbP family protein	28.06	27.4	**-0.67**	27.73	28.18	28.27	27.4	27.34	27.45	7	7	7	57	1.77	-3.94	32.3
AT1G50670	OTU-like cysteine protease family protein	26.81	26.15	**-0.66**	27.09	26.56	26.79	25.88	26.26	26.31	5	5	5	25	1.49	-3.22	31.2
AT1G05010	EFE, ACO4, EAT1 ethylene-forming enzyme	30.19	29.52	**-0.66**	30.34	30.14	30.08	29.62	29.47	29.48	15	14	14	199	2.71	-7.21	61.6
AT5G56360	PSL4 calmodulin-binding protein	26.65	25.99	**-0.66**	26.62	26.44	26.9	25.79	26.28	25.91	14	14	14	42	1.53	-3.32	27.7
AT4G31120	PRMT5 SHK1 binding protein 1	26.69	26.04	**-0.66**	26.51	26.62	26.95	25.92	26.21	25.98	9	9	9	29	1.83	-4.11	18.8
AT1G29350	Kinase-related protein of unknown function (DUF1296)	27.05	26.39	**-0.66**	27.3	27.01	26.82	26.4	26.45	26.31	11	11	11	64	1.98	-4.54	21.9
AT5G44320	Eukaryotic translation initiation factor 3 subunit 7 (eIF-3)	28.49	27.84	**-0.65**	28.57	28.65	28.25	28.04	27.95	27.52	18	18	7	85	1.5	-3.23	38.4
AT2G07050	CAS1 cycloartenol synthase 1	28.34	27.7	**-0.65**	28.57	28.19	28.28	27.61	27.74	27.74	21	21	21	105	2.23	-5.36	33.6
AT3G13470	TCP-1/cpn60 chaperonin family protein	30.65	30.01	**-0.64**	30.95	30.54	30.45	30.21	29.89	29.92	56	15	15	215	1.61	-3.51	75.3
AT1G71220	EBS1 UDP-gluc	28.19	27.55	**-0.64**	28.37	28.16	28.06	27.62	27.51	27.51	36	36	36	103	2.56	-6.59	28.1
AT5G67030	ABA1, LOS6, NPQ2, ATABA1, ZEP, IBS3, ATZEP zeaxanthin epoxidase (ZEP) (ABA1)	29.3	28.66	**-0.64**	29.44	29.17	29.3	28.6	28.61	28.78	30	30	30	137	2.52	-6.43	48.6
AT4G15760	MO1 monooxygenase 1	28.21	27.57	**-0.64**	28.3	28.22	28.11	27.32	27.93	27.46	8	8	8	62	1.53	-3.31	26.2
AT2G32920	ATPDIL2-3, PDI9, ATPDI9, PDIL2-3 PDI-like 2-3	27.03	26.4	**-0.63**	26.76	27.25	27.08	26.69	26.2	26.3	8	8	8	32	1.44	-3.09	31.4
AT3G10050	OMR1 L-O-methylthreonine resistant 1	24.82	24.19	**-0.63**	24.67	24.69	25.1	24.1	24.2	24.27	6	6	6	15	1.86	-4.19	16.4
AT1G78570	RHM1, ROL1, ATRHM1 rhamnose biosynthesis 1	28.76	28.13	**-0.63**	28.82	28.71	28.75	28.06	28.15	28.19	26	26	16	110	3.68	-12.88	53.2
AT4G14360	S-adenosyl-L-methionine-dependent methyltransferases superfamily protein	26.11	25.48	**-0.62**	25.91	26.4	26.01	25.61	25.44	25.4	13	11	5	32	1.71	-3.78	30.1
AT3G55360	CER10, ECR, ATTSC13, TSC13 3-oxo-5-alpha-steroid 4-dehydrogenase family protein	30.06	29.44	**-0.62**	29.87	30.14	30.19	29.41	29.39	29.52	12	12	12	69	2.35	-5.77	29.4

AT3G20370	TRAF-like family protein	30.57	29.95	**-0.62**	30.79	30.46	30.45	30.17	29.95	29.73	14	14	14	171	1.68	-3.69	43.3
AT1G14670	Endomembrane protein 70 protein family	28.77	28.15	**-0.62**	29.07	28.59	28.65	28.27	28.05	28.13	17	17	7	74	1.72	-3.8	27
AT5G62700	TUB2 tubulin beta chain 2	33.09	32.47	**-0.62**	33.07	33.11	33.09	32.54	32.44	32.44	32	32	5	889	4.27	-18.15	81.8
AT3G14930	HEME1 Uroporphyrinogen	30.31	29.69	**-0.62**	30.55	30.14	30.23	29.97	29.56	29.53	19	19	19	162	1.5	-3.25	47.1
AT4G11820	MVA1, FKP1 hydroxymethylglutaryl-Co	26.77	26.16	**-0.62**	26.78	26.73	26.81	26.27	26.3	25.9	10	10	10	51	2.03	-4.7	27.6
AT1G56340	CRT1, CRT1a, AtCRT1a calreticulin 1a	29.98	29.36	**-0.61**	29.92	30	30.01	29.34	29.41	29.33	28	28	17	183	4.01	-15.6	65.2
AT1G54010	GDSL-like Lipase/Acylhydrolase superfamily protein	32.86	32.25	**-0.61**	32.73	33.04	32.8	32.28	32.33	32.13	19	19	18	467	2.27	-5.48	57
AT3G53750	ACT1, AAc1 actin 1	27.66	27.05	**-0.61**	27.73	27.55	27.71	27.42	26.97	26.76	23	7	5	56	1.41	-3.03	69.8
AT2G30520		27.9	27.29	**-0.61**	27.8	27.97	27.94	27.24	27.16	27.48	19	19	19	66	2.32	-5.68	39.1
AT1G76860	Small nuclear ribonucleoprotein family protein	25.86	25.25	**-0.61**	26.14	25.58	25.88	25.18	25.17	25.41	4	4	4	13	1.55	-3.37	36.7
AT3G44340	CEF clone eighty-four	27.49	26.89	**-0.6**	27.46	27.64	27.39	26.99	26.9	26.79	18	18	12	76	2.51	-6.4	21.4
AT5G17920	ATCIMS, ATMETS, ATMS1 Cobalamin-independent synthase family protein	35.88	35.28	**-0.6**	35.91	35.89	35.84	35.21	35.27	35.36	66	66	33	2241	3.65	-12.63	75.9
AT2G28790	Pathogenesis-related thaumatin superfamily protein	26.77	26.17	**-0.6**	26.84	26.85	26.63	26.35	25.88	26.29	6	6	6	30	1.67	-3.68	38.6
AT1G01090	PDH-E1 ALPHA pyruvate dehydrogenase E1 alpha	30.41	29.81	**-0.6**	30.66	30.29	30.28	29.93	29.81	29.69	25	25	25	222	1.86	-4.2	56.5
AT5G22650	HD2B, HDT02, HDT2, ATHD2B, HDA4, HD2 histone deacetylase 2B	27.79	27.19	**-0.6**	27.67	27.68	28.02	27.1	27.05	27.43	8	8	8	54	1.65	-3.61	28.8
AT2G30050	transducin family protein / WD-40 repeat family protein	26.22	25.62	**-0.59**	26.14	26.08	26.43	25.88	25.4	25.59	9	8	8	32	1.55	-3.36	47
AT2G17840	ERD7 Senescence/dehydration-associated protein-related	26.72	26.13	**-0.59**	26.87	26.59	26.69	26.15	26.11	26.12	11	11	11	49	2.74	-7.36	28.3
ATCG00190	RPOB RNA polymerase subunit beta	29.05	28.46	**-0.59**	28.84	29.22	29.08	28.41	28.4	28.56	31	31	31	149	2.07	-4.83	35.1
AT1G26850	S-adenosyl-L-methionine-dependent methyltransferases superfamily protein	26.77	26.19	**-0.58**	26.7	26.67	26.93	26.43	26.06	26.06	14	14	14	35	1.78	-3.95	28.9
AT3G09260	PYK10, PSR3.1, BGLU23, LEB Glycosyl hydrolase superfamily protein	35.79	35.21	**-0.58**	35.76	35.86	35.75	35.12	35.39	35.11	40	40	33	1867	2.36	-5.82	71.4
AT1G35720	ANNAT1, OXY5, ATOXY5 annexin 1	29.17	28.59	**-0.58**	29.53	28.9	29.08	28.66	28.7	28.42	17	17	17	99	1.31	-2.79	57.7
AT1G18540	Ribosomal protein L6 family protein	30.73	30.15	**-0.57**	30.93	30.6	30.64	30.29	30.07	30.1	23	11	11	114	2	-4.6	70
AT5G54900	ATRBP45A, RBP45A RNA-binding protein 45A	26.14	25.58	**-0.57**	26.02	26.12	26.29	25.62	25.5	25.62	4	4	4	27	2.49	-6.31	13.4
AT1G21750	ATPDIL1-1, PDIL1-1 PDI-like 1-1	30.35	29.79	**-0.56**	30.48	30.23	30.34	29.8	29.79	29.78	39	39	33	289	2.84	-7.8	73.7
AT2G20760	Clathrin light chain protein	27.35	26.79	**-0.56**	27.67	27.18	27.2	26.9	26.72	26.75	11	11	11	45	1.54	-3.34	25.7
AT5G56010	HSP81-3, Hsp81.3, AtHsp90-3, AtHsp90.3 heat shock protein 81-3	27.52	26.96	**-0.56**	27.82	27.4	27.33	26.94	26.84	27.09	66	5	3	57	1.52	-3.29	67.4
AT5G13520	peptidase M1 family protein	27	26.44	**-0.56**	27.13	26.78	27.1	26.49	26.25	26.59	12	12	12	32	1.7	-3.75	27.4
ATCG00720	PETB photosynthetic electron transfer B	33.52	32.96	**-0.56**	33.4	33.64	33.53	33.21	32.8	32.88	9	9	9	350	1.78	-3.96	37.2
AT2G22125	CSI1 binding	28.99	28.43	**-0.56**	29.01	29.1	28.85	28.46	28.52	28.31	52	52	52	181	2.35	-5.77	32.3
AT1G53310	ATPPC1, PEPC1, ATPEPC1, PPC1 phosphoenolpyruvate carboxylase 1	30.19	29.63	**-0.56**	30.22	30.18	30.16	29.8	29.62	29.48	64	40	24	196	2.37	-5.86	67.1
AT5G26667	PYR6 P-loop containing nucleoside triphosphate hydrolases superfamily protein	27.76	27.21	**-0.55**	28.03	27.6	27.65	27.38	27.19	27.06	11	11	10	58	1.56	-3.39	59.4
AT1G11660	heat shock protein 70 (Hsp 70) family protein	27.35	26.8	**-0.55**	27.28	27.41	27.35	27.11	26.82	26.47	18	18	18	52	1.35	-2.89	32.1
AT2G21060	ATGRP2B, ATCSP4, GRP2B glycine-rich protein 2B	26.61	26.07	**-0.54**	26.37	26.61	26.86	25.94	25.95	26.32	5	5	4	27	1.34	-2.87	44.3
AT2G34860	EDA3 DnaJ/Hsp40 cysteine-rich domain superfamily protein	25.93	25.39	**-0.54**	25.74	26.04	26.02	25.15	25.57	25.46	4	4	4	19	1.57	-3.41	26.3
AT3G59780	Rhodanese/Cell cycle control phosphatase superfamily protein	29.4	28.86	**-0.54**	29.25	29.66	29.29	29.01	28.65	28.92	24	24	24	163	1.49	-3.21	41.3
AT4G31480	Coatomer, beta subunit	30.59	30.06	**-0.54**	30.88	30.36	30.54	30.06	29.91	30.2	41	41	3	294	1.43	-3.07	47.4
AT1G27090	glycine-rich protein	27.73	27.19	**-0.54**	27.7	27.71	27.78	27.45	26.9	27.23	16	16	16	66	1.51	-3.27	53.8
AT4G22690	CYP706A2 cytochrome P450, family 706, subfamily A, polypeptide 2	27.09	26.55	**-0.54**	27.07	26.89	27.31	26.51	26.43	26.71	13	13	13	45	1.65	-3.62	26.6
AT3G52140	tetratricopeptide repeat (TPR)-containing protein	30.18	29.64	**-0.53**	30.38	30.04	30.11	29.79	29.51	29.62	52	52	52	290	1.82	-4.07	44.7
AT2G30970	ASP1 aspartate aminotransferase 1	31.68	31.15	**-0.53**	31.91	31.57	31.57	31.39	31.03	31.03	29	29	29	344	1.49	-3.21	80.9
AT2G38550	Transmembrane proteins 14C	28.05	27.52	**-0.53**	27.93	28.15	28.07	27.39	27.62	27.54	10	10	10	57	2.32	-5.67	34.6
AT3G19820	DWF1, DIM, EVE1, DIM1, CBB1 cell elongation protein / DWARF1 / DIMINUTO (DIM)	30.45	29.91	**-0.53**	30.78	30.24	30.31	30.03	29.84	29.88	26	26	26	294	1.38	-2.95	43.1
AT5G13490	AAC2 ADP/ATP carrier 2	29.49	28.96	**-0.53**	29.69	29.44	29.34	28.75	29.11	29.02	19	8	7	85	1.63	-3.56	40.5
AT2G03120	ATSPP, SPP signal peptide peptidase	26.15	25.62	**-0.53**	26.2	26.19	26.06	25.72	25.55	25.59	6	6	6	22	2.81	-7.66	15.4
AT5G16730	Plant protein of unknown function (DUF827)	27.86	27.33	**-0.53**	28.2	27.65	27.73	27.22	27.46	27.31	26	26	26	76	1.34	-2.87	38.9
AT5G23040	CDF1 Protein of unknown function (DUF3353)	27.54	27.01	**-0.53**	27.77	27.49	27.36	26.94	27.02	27.09	5	5	5	46	1.82	-4.07	22.1
AT1G70770	Protein of unknown function DUF2359, transmembrane	27.92	27.4	**-0.53**	28.02	27.88	27.86	27.25	27.51	27.42	15	15	12	64	2.35	-5.76	28.4
AT1G79690	atnudt3, NUDT3 nudix hydrolase homolog 3	28.84	28.32	**-0.52**	28.97	28.92	28.63	28.47	28.07	28.42	30	30	30	134	1.48	-3.18	46.1
AT3G48730	GSA2 glutamate-1-semialdehyde 2,1-aminomutase 2	32.17	31.65	**-0.52**	32.27	32.13	32.12	31.84	31.57	31.54	22	22	13	438	2.09	-4.87	74.2
AT3G51160	MUR1, MUR_1, GMD2 NAD(P)-binding Rossmann-fold superfamily protein	27.71	27.2	**-0.51**	27.92	27.49	27.73	27.15	27.34	27.11	13	13	10	70	1.64	-3.6	45.6
AT1G14830	ADL1C, ADL5, DRP1C, DL1C DYNAMIN-like 1C	28.24	27.73	**-0.51**	28.29	28.26	28.16	27.79	27.73	27.66	28	27	21	89	3.14	-9.35	57.2
AT3G48560	CSR1, ALS, AHAS, TZP5, IMR1 chlorsulfuron/imidazolinone resistant 1	28.48	27.97	**-0.51**	28.52	28.43	28.48	28.17	27.83	27.91	22	22	22	97	2.07	-4.82	43.9
AT1G20050	HYD1 C-8,7 sterol isomerase	25.33	24.82	**-0.51**	25.41	25.51	25.06	24.74	24.75	24.96	3	3	3	13	1.52	-3.28	17
AT3G62360	Carbohydrate-binding-like fold	29.28	28.78	**-0.5**	29.6	29.05	29.19	28.9	28.7	28.74	43	43	43	177	1.34	-2.86	42.2
AT3G10370	SDP6 FAD-dependent oxidoreductase family protein	26.29	25.8	**-0.5**	26.51	26.26	26.11	25.88	25.71	25.8	17	17	17	32	1.77	-3.93	39.1
AT5G36230	ARM repeat superfamily protein	28.03	27.53	**-0.5**	28.29	27.81	27.99	27.73	27.42	27.46	11	11	6	68	1.37	-2.94	39.2
AT4G19210	ATRLI2, RLI2 RNAse l inhibitor protein 2	29.02	28.52	**-0.5**	29.19	28.93	28.93	28.33	28.61	28.63	18	18	18	133	1.74	-3.86	36.7
AT4G21150	HAP6 riboph	29.39	28.9	**-0.49**	29.38	29.37	29.43	29.04	28.91	28.75	28	28	28	157	2.35	-5.77	45.6
AT3G44330	INVOLVED IN: protein processing;LOCATED IN: mitochondrion, endoplasmic reticulum, plasma membrane, vacuole	26.8	26.31	**-0.49**	26.7	26.73	26.99	26.13	26.37	26.44	14	14	14	54	1.7	-3.75	34.5
AT1G07890	APX1, MEE6, CS1, ATAPX1, ATAPX01 ascorbate peroxidase 1	33.58	33.09	**-0.49**	33.52	33.65	33.57	33.23	33.03	33.02	24	24	23	509	2.44	-6.09	89.6
AT5G53560	ATB5-A, B5 #2, ATCB5-E, CB5-E cytochrome B5 isoform E	27.67	27.18	**-0.49**	27.68	27.64	27.68	27.26	27.15	27.13	9	9	9	59	3.51	-11.67	76.1
AT3G24503	ALDH2C4, ALDH1A, REF1 aldehyde dehydrogenase 2C4	29.86	29.37	**-0.48**	29.98	29.75	29.84	29.5	29.43	29.19	27	27	27	177	1.89	-4.26	67.1
AT5G20950	Glycosyl hydrolase family protein	29.32	28.84	**-0.48**	29.48	29.24	29.24	28.9	28.83	28.78	23	23	22	137	2.31	-5.62	45.4
AT5G61780	Tudor2, AtTudor2, TSN2 TUDOR-SN protein 2	31.27	30.79	**-0.48**	31.39	31.29	31.13	30.91	30.7	30.76	60	60	45	383	2.09	-4.89	62
AT1G10840	TIF3H1 translation initiation factor 3 subunit H1	28.24	27.76	**-0.48**	28.31	28.14	28.27	28.08	27.58	27.61	12	12	12	59	1.31	-2.8	44.2
AT5G47770	FPS1 farnesyl diphosphate synthase 1	27.74	27.26	**-0.47**	27.73	27.77	27.71	27.23	27.28	27.27	11	11	7	54	4.44	-20.1	29.4
AT4G34830	MRL1 Pentatricopeptide repeat (PPR) superfamily protein	28.2	27.73	**-0.47**	28.24	28.16	28.2	27.92	27.78	27.48	25	25	25	125	1.65	-3.61	32.4
AT3G01120	MTO1, CGS, ATCYS1, CGS1 Pyridoxal phosphate (PLP)-dependent transferases superfamily protein	28.39	27.92	**-0.47**	28.43	28.33	28.41	28.18	27.74	27.84	13	13	13	68	1.58	-3.44	32
AT1G79560	EMB156, EMB36, EMB1047, FTSH12 FTSH protease 12	27.33	26.86	**-0.47**	27.28	27.59	27.12	26.91	26.8	26.87	22	22	22	67	1.55	-3.36	27.2
AT1G48600	PMEAMT, AtPMEAMT S-adenosyl-L-methionine-dependent methyltransferases su	29.16	28.7	**-0.47**	29.25	29.06	29.18	28.96	28.47	28.66	22	22	11	162	1.42	-3.06	52.2
AT3G24430	HCF101 ATP binding	28.18	27.71	**-0.47**	28.1	28.19	28.25	27.98	27.6	27.55	16	16	16	89	1.51	-3.26	45.3
AT4G13930	SHM4 serine hydroxymethyltransferase 4	32.36	31.89	**-0.47**	32.29	32.48	32.3	31.9	31.89	31.88	34	34	34	487	2.83	-7.75	85.6
AT5G19690	STT3A staurosporin and temperature sensitive 3-like A	27.06	26.59	**-0.46**	27.29	26.91	26.97	26.57	26.66	26.54	12	12	12	52	1.72	-3.8	15.8
AT1G62390	Phox2 Octicosapeptide/Phox/Bem1p (PB1) domain-containing protein / tetratricopeptide repeat (TPR)-containing protein	28.83	28.37	**-0.46**	28.98	28.73	28.79	28.55	28.21	28.35	27	27	27	151	1.67	-3.68	56.1
AT5G12950	Putative glycosyl hydrolase of unknown function (DUF1680)	26.16	25.7	**-0.46**	26.12	26.19	26.18	25.62	25.73	25.75	15	15	15	34	3.18	-9.61	23.8

AT5G60980	Nuclear transport factor 2 (NTF2) family protein with RNA binding	27.37	26.91	**-0.46**	27.51	27.38	27.22	26.94	26.9	26.88	14	14	14	50	2.23	-5.35	44.2
AT4G03280	PETC, PGR1 photosynthetic electron transfer C	33.66	33.2	**-0.46**	33.75	33.71	33.51	33.4	33.11	33.08	15	15	15	320	1.64	-3.58	53.8
AT3G20810	JMJD5 2-oxoglutarate (2OG) and Fe(II)-dependent oxygenase superfamily protein	26.01	25.55	**-0.46**	26.29	25.88	25.86	25.63	25.6	25.43	8	8	8	38	1.39	-2.98	32.2
AT5G20290	Ribosomal protein S8e family protein	32.24	31.79	**-0.46**	32.36	32.11	32.27	31.84	31.73	31.8	22	22	22	370	2.37	-5.86	62.2
AT5G56950	NFA03, NFA3, NAP1;3 nucleosome assembly protein 1;3	26.71	26.25	**-0.45**	26.6	26.84	26.68	26.28	26.39	26.09	14	10	10	27	1.82	-4.07	37.4
AT1G16920	RAB11, ATRABA1B, RABA1b RAB GTPase homolog A1B	28.62	28.16	**-0.45**	28.4	28.64	28.81	28.16	28.34	27.99	12	12	6	50	1.36	-2.91	55.1
AT2G15620	NIR1, NIR, ATHNIR nitrite reductase 1	32.4	31.95	**-0.45**	32.42	32.32	32.46	32	31.91	31.94	48	48	48	637	3.15	-9.41	73.2
AT5G10360	EMB3010 Ribosomal protein S6e	32.27	31.82	**-0.45**	32.22	32.34	32.26	31.65	31.81	32	19	19	8	321	1.88	-4.23	56.6
AT1G36730	Translation initiation factor IF2/IF5	25.89	25.44	**-0.45**	25.6	26.12	25.96	25.48	25.48	25.35	9	9	9	31	1.33	-2.84	24.4
AT5G36890	BGLU42 beta glucosidase 42	26.57	26.12	**-0.45**	26.57	26.77	26.36	26.07	26.25	26.03	10	10	10	23	1.54	-3.33	30.2
AT2G01720	Ribophorin I	26.55	26.1	**-0.45**	26.79	26.53	26.33	26.23	26.05	26.02	9	9	9	47	1.39	-2.98	29.1
AT2G34640	PTAC12, HMR plastid transcriptionally active 12	26.28	25.83	**-0.45**	26.3	26.17	26.36	25.71	25.82	25.97	10	10	10	35	2.05	-4.76	25.6
AT3G09820	ADK1 adenosine kinase 1	31.66	31.22	**-0.44**	31.86	31.57	31.56	31.4	31.23	31.02	26	26	10	369	1.41	-3.04	76.5
AT4G15000	Ribosomal L27e protein family	30.66	30.21	**-0.44**	30.63	30.75	30.59	30.07	30.3	30.27	8	8	5	107	2.18	-5.18	44.3
AT3G57290	EIF3E, TIF3E1, ATEIF3E-1, INT-6, ATINT6, INT6 eukaryotic translation initiation factor 3E	29.6	29.15	**-0.44**	29.62	29.54	29.63	29.19	29.28	28.99	26	26	26	170	2.12	-4.97	69.2
AT5G05010	clathrin adaptor complexes medium subunit family protein	29.43	28.99	**-0.44**	29.44	29.5	29.36	29.06	28.95	28.96	17	17	17	206	2.97	-8.44	43.3
AT1G09210	CRT1b, AtCRT1b calreticulin 1b	28.49	28.05	**-0.44**	28.31	28.69	28.48	28.22	27.97	27.96	27	16	16	70	1.46	-3.15	65.1
AT1G58080	ATATP-PRT1, HISN1A, ATP-PRT1 ATP phosphoribosyl transferase 1	27.71	27.27	**-0.43**	27.81	27.68	27.62	27.45	27.25	27.11	8	6	6	50	1.73	-3.84	30.7
AT5G44340	TUB4 tubulin beta chain 4	29.49	29.06	**-0.43**	29.49	29.49	29.49	29.07	29.12	28.98	30	6	6	142	3.29	-10.21	83.6
AT5G27640	TIF3B1, EIF3B, ATEIF3B-1, EIF3B-1, ATTIF3B1 translation initiation factor 3B1	30.19	29.76	**-0.43**	30.23	30.2	30.14	29.84	29.72	29.72	34	34	6	225	3.08	-9.05	48.2
AT5G51430	EYE conserved oligomeric Golgi complex component-related / COG complex component-related	24.77	24.34	**-0.43**	24.79	24.63	24.89	24.29	24.26	24.47	9	9	9	20	1.9	-4.3	15
AT5G03300	ADK2 adenosine kinase 2	28.46	28.04	**-0.43**	28.69	28.38	28.32	28.07	28.14	27.9	25	9	9	80	1.46	-3.15	75.9
AT4G30720	FAD/NAD(P)-binding oxidoreductase family protein	26.84	26.41	**-0.42**	26.98	26.79	26.75	26.59	26.22	26.43	17	17	17	55	1.5	-3.24	29.1
AT4G15110	CYP97B3 cytochrome P450, family 97, subfamily B, polypeptide 3	27.64	27.22	**-0.42**	27.54	27.84	27.56	27.28	27.29	27.1	15	15	15	56	1.68	-3.69	31.6
AT2G18020	EMB2296 Ribosomal protein L2 family	32.31	31.89	**-0.42**	32.41	32.18	32.34	32.08	31.87	31.73	14	14	5	260	1.57	-3.41	56.6
AT5G56630	PFK3 phosphofructokinase 3	24.75	24.33	**-0.42**	24.85	24.76	24.65	24.32	24.27	24.41	8	6	5	19	2.39	-5.91	18.4
ATCG00490	RBCL ribulose-bisphosphate carboxylases	39.35	38.93	**-0.42**	39.47	39.34	39.26	38.99	38.85	38.96	71	71	66	11365	2.33	-5.71	76.6
AT4G12650	Endomembrane protein 70 protein family	28.06	27.64	**-0.42**	27.98	28.29	27.91	27.69	27.69	27.55	17	17	14	85	1.54	-3.34	26.2
AT1G67430	Ribosomal protein L22p/L17e family protein	31.66	31.25	**-0.42**	31.64	31.58	31.77	31.24	31.12	31.38	14	14	2	180	1.95	-4.45	61.1
AT4G20890	TUB9 tubulin beta-9 chain	30.13	29.71	**-0.42**	30.03	30.19	30.16	29.75	29.67	29.72	30	8	4	118	2.86	-7.9	84.2
AT5G58590	RANBP1 RAN binding protein 1	25.52	25.1	**-0.41**	25.46	25.7	25.38	25.11	25.3	24.9	5	4	4	27	1.3	-2.78	43.4
AT5G07350	Tudor1, AtTudor1, TSN1 TUDOR-SN protein 1	30.56	30.15	**-0.41**	30.66	30.47	30.54	30.23	30.08	30.14	59	44	44	311	2.38	-5.89	64.2
AT1G11650	ATRBP45B, RBP45B RNA-binding (RRM/RBD/RNP motifs) family protein	28.27	27.86	**-0.41**	28.26	28.21	28.33	27.98	27.91	27.7	8	8	8	67	1.96	-4.48	25.7
AT3G04870	ZDS, PDE181, SPC1 zeta-carotene desaturase	29.06	28.65	**-0.41**	29.16	29	29.01	28.71	28.56	28.69	23	23	23	143	2.36	-5.81	47
AT4G34200	EDA9 D-3-phosphoglycerate dehydrogenase	30.36	29.96	**-0.4**	30.37	30.34	30.35	30.06	29.85	29.96	27	27	23	226	2.53	-6.47	53.1
AT2G23350	PAB4, PABP4 poly(A) binding protein 4	29.79	29.39	**-0.4**	29.84	29.83	29.7	29.52	29.28	29.38	29	29	28	179	2.06	-4.78	51.1
AT2G21160	Translocon-associated protein (TRAP), alpha subunit	27.5	27.11	**-0.4**	27.5	27.39	27.62	27.22	26.9	27.19	4	4	4	20	1.5	-3.24	17.8
AT1G74030	ENO1 enolase 1	27.13	26.73	**-0.4**	27.22	27.03	27.14	26.71	26.85	26.64	10	10	10	61	2.05	-4.75	27.5
AT3G04400	|	31.02	30.62	**-0.4**	31.14	30.91	31.01	30.73	30.61	30.53	16	16	16	312	1.98	-4.53	77.1
AT5G16620	PDE120, TIC40, ATTIC40 hydroxyproline-rich glycoprotein family protein	27.66	27.26	**-0.4**	27.67	27.74	27.55	27.3	27.33	27.15	13	13	13	97	2.16	-5.11	38.5
AT1G12270	Hop1 stress-inducible protein, putative	28.14	27.75	**-0.39**	28.31	28.08	28.04	27.59	27.76	27.9	31	31	24	129	1.49	-3.22	60.8
AT4G11420	EIF3A, ATEIF3A-1, EIF3A-1, ATTIF3A1, TIF3A1 eukaryotic translation initiation factor 3A	31.57	31.18	**-0.39**	31.73	31.47	31.52	31.23	31.13	31.19	62	62	62	432	1.99	-4.58	57.8
AT5G52970	thylakoid lumen 15.0 kDa protein	27.1	26.72	**-0.39**	27.13	27.22	26.96	26.74	26.72	26.68	6	6	6	34	2.13	-5.01	33.2
AT5G60640	ATPDIL1-4, PDIL1-4 PDI-lik	29.38	29	**-0.39**	29.4	29.32	29.43	29.02	29.02	28.94	23	23	23	148	3.14	-9.39	43.2
AT5G11880	Pyridoxal-dependent decarboxylase family protein	30.62	30.23	**-0.39**	30.73	30.56	30.57	30.42	30.12	30.17	21	21	9	183	1.63	-3.56	54.8
AT1G08200	AXS2 UDP-D-apiose/UDP-D-xylose synthase 2	29.46	29.07	**-0.39**	29.54	29.42	29.41	29.24	29.06	28.92	20	20	6	145	1.7	-3.76	62.2
AT1G56070	LOS1 Ribosomal protein S5/Elongation factor G/III/V family protein	34.45	34.07	**-0.38**	34.55	34.43	34.36	34.06	34.03	34.12	77	77	49	2156	2.46	-6.2	76.9
AT3G25230	ROF1, ATFKBP62, FKBP62 rotamase FKBP 1	29.68	29.3	**-0.38**	29.91	29.52	29.62	29.26	29.24	29.4	28	28	27	188	1.41	-3.03	55.4
AT1G31230	AK-HSDH I, AK-HSDH aspartate kinase-homoserine dehydrogenase i	29.68	29.29	**-0.38**	29.8	29.65	29.58	29.5	29.28	29.1	41	41	32	185	1.33	-2.84	54.7
AT5G16390	CAC1, CAC1A, BCCP, BCCP1 chloroplastic acetylcoenzyme A carboxylase 1	28.09	27.7	**-0.38**	28.11	28.09	28.06	27.65	27.58	27.88	8	8	8	62	1.89	-4.28	26.4
AT2G40730	Protein kinase family protein with ARM repeat domain	25.74	25.36	**-0.38**	25.94	25.72	25.55	25.37	25.42	25.28	11	11	11	32	1.45	-3.12	19.7
AT4G31790	Tetrapyrrole (Corrin/Porphyrin) Methylases	25.21	24.83	**-0.38**	25.32	25.23	25.09	24.67	24.98	24.84	3	3	3	13	1.56	-3.38	12.6
AT2G27860	AXS1 UDP-D-apiose/UDP-D-xylose synthase 1	26.93	26.55	**-0.38**	26.94	26.98	26.87	26.44	26.76	26.44	19	5	5	36	1.57	-3.42	65.6
AT3G11400	EIF3G1, ATEIF3G1 eukaryotic translation initiation factor 3G1	28.16	27.77	**-0.38**	28.29	28.14	28.04	27.89	27.75	27.68	12	12	12	50	1.77	-3.93	52
AT4G34110	PAB2, PABP2, ATPAB2 poly(A) binding protein 2	29.42	29.04	**-0.38**	29.35	29.44	29.48	29.14	28.98	29	25	24	22	153	2.39	-5.92	46.4
AT4G16660	heat shock protein 70 (Hsp 70) family protein	28.35	27.97	**-0.38**	28.51	28.26	28.28	28.02	28.06	27.83	21	21	21	87	1.61	-3.52	30.3
AT1G31910	GHMP kinase family protein	25.36	24.98	**-0.38**	25.32	25.2	25.55	25.02	24.93	24.98	7	7	7	15	1.6	-3.5	21.2
AT5G02500	HSC70-1, HSP70-1, AT-HSC70-1, HSC70 heat shock cognate protein 70-1	33.98	33.6	**-0.38**	34.13	33.9	33.91	33.53	33.59	33.69	63	63	21	1441	1.9	-4.31	85.9
AT4G34450	coatomer gamma-2 subunit, putative / gamma-2 coat protein, putative / gamma-2 COP, putative	31.09	30.72	**-0.37**	31.08	31.16	31.04	30.93	30.6	30.62	49	49	49	402	1.52	-3.3	74.7
AT5G05980	ATDFB, DFB, FPGS1 DHFS-FPGS homolog B	25.83	25.46	**-0.37**	25.73	25.95	25.83	25.6	25.24	25.56	7	7	6	28	1.34	-2.87	17.5
AT5G38470	RAD23D Rad23 UV excision repair protein family	27.53	27.16	**-0.37**	27.59	27.44	27.55	26.94	27.22	27.32	8	8	5	63	1.42	-3.05	19.3
AT5G28500	unknown protein	29.5	29.13	**-0.37**	29.54	29.49	29.46	29.17	29.15	29.06	13	13	13	160	3.04	-8.83	38.9
AT5G63840	RSW3, PSL5 Glycosyl hydrolases family 31 protein	28.81	28.44	**-0.37**	28.95	28.66	28.82	28.29	28.52	28.51	31	31	31	124	1.52	-3.29	40
AT5G58710	ROC7 rotamase CYP 7	27.17	26.8	**-0.36**	27	27.24	27.27	26.93	26.73	26.76	11	10	8	50	1.6	-3.48	50.5
AT5G25757	RNA polymerase I-associated factor PAF67	28.76	28.4	**-0.36**	28.86	28.77	28.65	28.38	28.48	28.34	22	22	22	116	2.08	-4.86	55.4
AT3G52930	Aldolase superfamily protein	33.78	33.42	**-0.36**	33.83	33.72	33.8	33.37	33.49	33.42	34	34	21	894	2.74	-7.37	85.8
AT5G47010	UPF1, LBA1, ATUPF1 RNA helicase, putative	27.6	27.24	**-0.36**	27.81	27.51	27.47	27.29	27.16	27.27	24	24	24	82	1.47	-3.17	27.8
AT4G12730	FLA2 FASCICLIN-like arabinogalactan 2	29.73	29.37	**-0.36**	29.61	29.89	29.68	29.46	29.38	29.28	11	11	11	131	1.66	-3.64	36
AT5G41670	6-phosphogluconate dehydrogenase family protein	30.66	30.3	**-0.35**	30.85	30.57	30.56	30.28	30.34	30.3	28	28	14	315	1.65	-3.61	63.2
AT5G49460	ACLB-2 ATP citrate lyase subunit B 2	27.5	27.15	**-0.35**	27.5	27.53	27.48	27.01	27.2	27.24	27	7	7	57	2.1	-4.93	53.3
AT2G21870	MGP1 copper ion binding;cobalt ion binding;zinc ion binding	29.38	29.03	**-0.35**	29.5	29.28	29.36	29.14	28.87	29.08	20	20	20	151	1.57	-3.42	62.1
AT5G47210	Hyaluronan / mRNA bindin	30.6	30.25	**-0.35**	30.74	30.43	30.64	30.12	30.29	30.34	27	27	22	332	1.46	-3.14	62.2
AT3G54470	uridine 5-monophosphate synthase / UMP synthase (PYRE-F) (UMPS)	29.11	28.76	**-0.35**	29.02	29.19	29.13	28.97	28.62	28.69	22	22	22	125	1.41	-3.02	59
AT3G43300	ATMIN7, BEN1 HOPM interactor 7	30.32	29.97	**-0.35**	30.52	30.16	30.27	29.95	29.93	30.02	74	74	74	371	1.49	-3.21	48.1
AT3G07100	ERMO2, SEC24A Sec23/Sec24 protein transport family protein	28.17	27.82	**-0.35**	28.25	28.09	28.17	27.93	27.8	27.73	26	26	26	123	2.05	-4.75	29.4

AT2G36250	FTSZ2-1, ATFTSZ2-1 Tubulin/FtsZ family protein	28.05	27.7	**-0.35**	28.15	27.99	28	27.9	27.65	27.55	14	14	7	124	1.4	-3	42.5
AT1G09780	Phosphoglycerate mutase, 2,3-bisphosphoglycerate-independent	30.86	30.52	**-0.35**	30.86	30.87	30.86	30.42	30.64	30.49	36	23	23	181	2.22	-5.32	72.7
AT1G80480	PTAC17 plastid transcriptionally active 17	29.3	28.95	**-0.35**	29.16	29.37	29.37	29.07	29.01	28.78	15	15	13	97	1.44	-3.09	46.4
AT1G62750	ATSCO1, ATSCO1/CPEF-G, SCO1 Translation elongation factor EFG/EF2 protein	33.19	32.84	**-0.34**	33.15	33.28	33.13	33.03	32.74	32.76	55	55	55	1076	1.55	-3.37	73.4
AT2G29560	ENOC cytosolic enolase	26.42	26.08	**-0.34**	26.5	26.4	26.36	26.12	25.98	26.14	9	9	9	40	2.14	-5.05	25.9
AT4G14210	PDS3, PDS, PDE226 phytoene desaturase 3	28.28	27.94	**-0.34**	28.37	28.23	28.24	28.1	27.88	27.85	21	21	21	105	1.68	-3.7	46.5
AT2G20610	SUR1, HLS3, RTY, ALF1, RTY1 Tyrosine transaminase family protein	26.31	25.98	**-0.33**	26.44	26.19	26.3	26.13	25.83	25.98	12	12	11	41	1.38	-2.97	29.9
AT1G56050	GTP-binding protein-related	28.25	27.92	**-0.33**	28.39	28.19	28.17	27.98	27.89	27.88	13	13	13	67	1.91	-4.35	45.1
AT5G19990	RPT6A, ATSUG1 regulatory particle triple-A ATPase 6A	30	29.67	**-0.33**	30.19	29.92	29.89	29.75	29.64	29.62	26	26	2	163	1.48	-3.2	68.5
AT2G34480	Ribosomal protein L18ae/LX family protein	31.59	31.26	**-0.33**	31.42	31.67	31.67	31.09	31.34	31.34	17	17	7	303	1.33	-2.84	68.5
AT5G56680	SYNC1, EMB2755, SYNC1 ARATH Class II aminoacyl-tRNA and biotin synthetases superfamily protein	29.31	28.98	**-0.33**	29.46	29.16	29.3	28.99	28.91	29.05	26	26	26	141	1.56	-3.4	45.5
AT1G77510	ATPDIL1-2, PDI6, ATPDI6, PDIL1-2 PDI-like 1-2	28.2	27.87	**-0.32**	28.23	28.11	28.24	27.91	27.7	28	26	20	19	80	1.52	-3.3	58.1
AT5G20890	TCP-1/cpn60 chaperonin family protein	29.95	29.63	**-0.32**	30.04	29.96	29.87	29.8	29.6	29.5	29	29	29	227	1.48	-3.19	67.2
AT2G05920	Subtilase family protein	28.29	27.98	**-0.32**	28.38	28.3	28.2	28.07	27.96	27.89	19	19	19	104	1.91	-4.32	45.8
AT4G38630	RPN10, MCB1, ATMCB1, MBP1 regulatory particle non-ATPase 10	28.26	27.95	**-0.31**	28.15	28.35	28.28	28.01	27.95	27.88	11	11	11	83	2	-4.61	37.6
AT2G43710	SSI2, FAB2 Plant stearoyl-acyl-carrier-protein desaturase family protein	29.23	28.92	**-0.31**	29.22	29.25	29.21	29.08	28.91	28.76	20	20	13	106	1.53	-3.32	45.6
AT1G09270	IMPA-4 importin alpha isoform 4 |	26.99	26.68	**-0.31**	26.81	27.15	26.99	26.68	26.7	26.66	9	8	8	36	1.42	-3.06	33.8
AT1G43170	RP1 ribosomal protein 1	33.04	32.74	**-0.3**	33.2	32.91	33.02	32.74	32.75	32.73	39	39	28	712	1.62	-3.55	73.3
ATCG00540	PETA photosynthetic electron transfer A	34.38	34.08	**-0.3**	34.43	34.34	34.38	34.22	33.95	34.07	23	23	23	732	1.64	-3.6	71.6
AT5G19780	TUA3 tubulin alpha-3	31.67	31.37	**-0.3**	31.64	31.74	31.62	31.48	31.43	31.19	29	9	7	320	1.46	-3.15	64.4
AT1G14320	SAC52, RPL10A Ribosomal protein L16p/L10e family protein	31.53	31.23	**-0.29**	31.44	31.52	31.62	31.28	31.14	31.28	18	18	3	190	1.86	-4.19	51.8
AT2G41740	VLN2, ATVLN2 villin 2	29.03	28.74	**-0.29**	29.16	28.98	28.96	28.79	28.61	28.82	39	34	34	198	1.49	-3.21	50.4
AT1G48410	AGO1 Stabilizer of iron transporter SufD / Polynucleotidyl transferase	28.49	28.2	**-0.29**	28.55	28.42	28.5	28.17	28.23	28.19	27	27	27	129	2.66	-7.01	28.5
AT3G06650	ACLB-1 ATP-citrate lyase B-1	30.59	30.3	**-0.29**	30.59	30.61	30.57	30.35	30.35	30.2	30	30	10	282	2.26	-5.46	60.2
AT5G02870	Ribosomal protein L4/L1 family	32.8	32.52	**-0.29**	32.96	32.72	32.72	32.54	32.53	32.49	32	32	12	625	1.61	-3.5	61.7
AT4G27500	PPI1 proton pump interactor 1	28.39	28.1	**-0.29**	28.38	28.39	28.39	28.2	28.09	28.01	22	22	22	106	2.15	-5.09	43
AT3G54400	Eukaryotic aspartyl protease family protein	29.94	29.65	**-0.29**	29.97	29.96	29.87	29.66	29.63	29.66	11	11	11	122	2.96	-8.43	33.6
AT1G20010	TUB5 tubulin beta-5 chain	31.26	30.97	**-0.28**	31.26	31.23	31.29	31.13	30.83	30.96	31	15	8	262	1.5	-3.24	79.5
AT3G09200	Ribosomal protein L10 family protein	32.15	31.86	**-0.28**	32.18	32.18	32.07	32.01	31.76	31.82	19	19	5	364	1.53	-3.32	85
AT2G10940	Bifunctional inhibitor/lipid-transfer protein/seed storage 2S albumin su	32.66	32.39	**-0.28**	32.74	32.56	32.68	32.4	32.33	32.42	9	9	9	191	2	-4.6	61.2
AT1G20950	Phosphofructokinase family protein	29.71	29.44	**-0.28**	29.81	29.65	29.68	29.43	29.4	29.48	32	32	19	202	2.09	-4.88	68.9
AT2G17980	ATSLY1 Sec1/munc18-like (SM) proteins superfamily	26.99	26.72	**-0.27**	26.89	27.06	27.04	26.85	26.59	26.73	16	16	16	46	1.37	-2.93	36.8
AT1G49760	PAB8, PABP8 poly(A) binding protein 8	29.1	28.83	**-0.27**	29.12	29.09	29.09	28.74	28.8	28.95	24	22	22	148	1.91	-4.33	49.3
AT4G27640	ARM repeat superfamily protein	27.55	27.28	**-0.27**	27.62	27.5	27.52	27.26	27.38	27.21	23	23	23	80	1.9	-4.29	27.7
AT4G14160	Sec2	28.18	27.92	**-0.27**	28.29	28.09	28.16	27.95	27.95	27.85	19	19	15	112	1.77	-3.93	38.3
AT2G27530	PGY1 Ribosomal protein L1p/L10e family	31.4	31.14	**-0.26**	31.42	31.32	31.48	31.12	31.12	31.18	13	13	6	220	2.17	-5.14	60.2
AT1G68560	ATXYL1, XYL1, TRG1 alpha-xylosidase 1	30.59	30.33	**-0.26**	30.61	30.57	30.59	30.45	30.2	30.33	35	35	34	335	1.6	-3.5	55.3
AT4G17870	PYR1, RCAR11 Polyketide cyclase/dehydrase and lipid transport superfamily protein	26.92	26.67	**-0.25**	27.04	26.83	26.9	26.68	26.72	26.6	10	10	9	61	1.6	-3.49	62.3
AT3G11710	ATKRS-1 lysyl-tRNA synthetase 1	29.56	29.31	**-0.25**	29.71	29.53	29.44	29.31	29.27	29.34	34	34	34	220	1.44	-3.1	63.6
AT3G07170	Sterile alpha motif (SAM) domain-containing protein	23.66	23.4	**-0.25**	23.72	23.55	23.69	23.46	23.47	23.29	4	4	4	12	1.52	-3.29	25.1
AT4G34740	ATASE2, CIA1, ATPURF2, ASE2 GLN phosphoribosyl pyrophosphate amidotransferase 2	28.76	28.51	**-0.25**	28.89	28.7	28.71	28.5	28.58	28.45	24	24	24	101	1.57	-3.42	49.7
AT3G48110	EDD1, EDD glycine-tRNA ligases	30.23	29.98	**-0.24**	30.14	30.26	30.27	30.1	29.85	30	42	42	42	220	1.38	-2.96	45.9
AT1G22300	GRF10, GF14 EPSILON general regulatory factor 10	30.42	30.18	**-0.24**	30.47	30.39	30.39	30.33	30.05	30.14	26	25	22	225	1.32	-2.83	73.2
AT4G31180	Class II aminoacyl-tRNA and biotin synthetases superfamily protein	28.5	28.27	**-0.23**	28.57	28.43	28.51	28.2	28.29	28.33	24	24	20	147	1.85	-4.16	50
AT5G58290	RPT3 regulatory particle triple-A ATPase 3	29.09	28.86	**-0.23**	29.19	29.06	29.02	28.81	28.86	28.91	22	21	21	111	1.75	-3.88	72.5
AT3G11830	TCP-1/cpn60 chaperonin family protein	29.66	29.44	**-0.23**	29.67	29.68	29.65	29.52	29.46	29.34	29	29	29	181	1.9	-4.3	63.4
AT3G04120	GAPC, GAPC-1, GAPC1 glyceraldehyde-3-phosphate dehydrogenase C subunit 1	30.76	30.53	**-0.22**	30.87	30.68	30.71	30.58	30.55	30.47	39	5	5	96	1.58	-3.43	87.9
AT4G02450	HSP20-like chaperones superfamily protein	29.3	29.08	**-0.22**	29.3	29.23	29.36	29.05	29.01	29.16	13	13	13	91	1.7	-3.75	47.1
AT4G29040	RPT2a regulatory particle AAA-ATPase 2A	29.18	28.96	**-0.22**	29.26	29.22	29.07	29.03	28.95	28.91	22	22	2	119	1.55	-3.35	57.6
AT5G46580	pentatricopeptide (PPR) repeat-containing protein	28.17	27.95	**-0.22**	28.23	28.17	28.11	27.91	27.93	28.01	25	25	25	89	2.07	-4.81	47.5
AT5G27470	seryl-tRNA synthetase / serine--tRNA ligase	30.72	30.5	**-0.22**	30.73	30.7	30.73	30.66	30.43	30.42	29	29	29	273	1.3	-2.78	53.7
AT1G80410	EMB2753 tetratricopeptide repeat (TPR)-containing protein	30.3	30.09	**-0.21**	30.36	30.25	30.29	30.17	30.07	30.03	53	53	53	415	1.81	-4.04	65
AT3G11910	UBP13 ubiquitin-specific protease 13	27.53	27.34	**-0.19**	27.54	27.51	27.54	27.45	27.22	27.34	46	20	20	83	1.32	-2.81	49
AT2G44160	MTHFR2 methylenetetrahydrofolate reductase 2	30.05	29.86	**-0.19**	30.09	30.05	30	29.91	29.82	29.85	37	30	30	181	2.14	-5.05	73.2
AT3G59920	ATGDI2, GDI2 RAB GDP dissociation inhibitor 2	30.04	29.85	**-0.19**	30.07	30.06	29.98	29.93	29.81	29.8	26	26	17	168	1.68	-3.7	68.7
AT3G06860	MFP2, ATMFP2 multifunctional protein 2	30.17	30	**-0.17**	30.22	30.2	30.1	29.95	30	30.05	38	38	38	234	1.65	-3.62	58.9
AT3G01670	unknown protein	25.1	24.96	**-0.14**	25.06	25.12	25.12	24.92	24.96	24.99	6	6	6	15	2.16	-5.1	9.2
AT2G30490	ATC4H, C4H, CYP73A5, REF3 cinnamate-4-hydroxylase	29.2	29.06	**-0.14**	29.12	29.21	29.26	29.05	29.07	29.05	25	25	25	177	1.53	-3.3	56.4
AT2G44100	ATGDI1, AT-GDI1, GDI1 guanosine nucleotide diphosphate dissociation inhibitor 1 |	28.16	28.03	**-0.13**	28.14	28.18	28.18	27.98	28.09	28.02	20	11	10	75	1.74	-3.85	54.8
AT3G16480	MPPalpha mitochondrial processing peptidase alpha subunit	25.95	25.9	**-0.06**	25.96	25.96	25.94	25.92	25.9	25.86	12	3	3	10	1.49	-3.22	26.1
AT1G59610	ADL3, CF1, DRP2B, DL3 dynamin-like 3	28.23	28.31	**0.08**	28.22	28.25	28.22	28.26	28.31	28.36	43	20	20	127	1.31	2.8	56
AT3G03250	UGP, UGP1, AtUGP1 UDP-GLUCOSE PYROPHOSPHORYLASE 1	29.92	30.03	**0.11**	29.97	29.9	29.89	30.05	30.06	29.99	30	15	15	192	1.48	3.2	72.7
AT5G63890	ATHDH, HISN8, HDH histidinol dehydrogenase	28.48	28.64	**0.16**	28.51	28.45	28.49	28.62	28.74	28.56	14	14	14	103	1.37	2.95	43.1
AT5G19620	EMB213, OEP80, ATOEP80, TOC75 outer envelope protein of 80 kDa	27.4	27.57	**0.17**	27.45	27.34	27.4	27.62	27.49	27.6	19	19	19	69	1.58	3.43	33.3
AT1G51650	ATP synthase epsilon chain, mitochondrial	26.52	26.7	**0.18**	26.52	26.44	26.6	26.69	26.64	26.77	5	5	5	42	1.4	3.01	77.1
ATCG00480	ATPB, PB ATP synthase subunit beta	36.72	36.92	**0.2**	36.68	36.74	36.73	37.02	36.93	36.8	50	50	49	4473	1.44	3.1	94.8
AT4G39710	FKBP16-2 FK506-binding protein 16-2	27.78	28	**0.22**	27.86	27.76	27.72	28.04	27.86	28.09	6	6	6	67	1.3	2.78	42.9
AT3G27850	RPL12-A, RPL12 ribosomal protein L12-A	32.87	33.09	**0.22**	32.95	32.75	32.91	33.08	33.08	33.12	13	13	13	282	1.67	3.68	59.4
AT2G26930	ATCDPMEK, PDE277, ISPE, CDPMEK 4-(cytidine 5-phospho)-2-C-methyl-D-erithritol kinase	27.08	27.3	**0.23**	27.11	27.04	27.07	27.18	27.39	27.34	8	8	8	54	1.62	3.55	29.2
AT5G50920	CLPC, ATHSP93-V, HSP93-V, DCA1, CLPC1 CLPC homologue 1	34.72	34.96	**0.24**	34.73	34.75	34.67	34.99	34.93	34.95	91	91	48	2465	2.93	8.24	75.1
AT4G26970	ACO2 aconitase 2	30.05	30.29	**0.24**	30.12	30.03	30	30.43	30.24	30.2	38	35	35	346	1.41	3.02	50.2
AT2G39480	PGP6 P-glycoprotein 6	25.32	25.57	**0.25**	25.33	25.3	25.33	25.53	25.46	25.71	9	9	4	28	1.51	3.26	8.5
AT4G26910	Dihydrolipoamide	27.6	27.85	**0.25**	27.66	27.57	27.57	27.9	27.86	27.78	11	11	6	63	2.28	5.52	35.3
AT3G02880	Leucine-rich repeat protein kinase family protein	27.79	28.04	**0.25**	27.76	27.89	27.73	27.92	28.11	28.09	16	16	13	99	1.47	3.16	31.6
AT2G32080	PUR ALPHA-1 purin-rich alpha 1	26.31	26.56	**0.25**	26.26	26.26	26.4	26.65	26.47	26.56	11	11	11	55	1.64	3.6	43.1

AT4G35760	NAD(P)H dehydrogenase (quinone)s	26.59	26.85	**0.25**	26.59	26.53	26.65	26.82	26.84	26.88	6	6	6	49	2.57	6.62	21.3
AT5G14740	CA2, CA18, BETA C	36.02	36.28	**0.26**	36.01	36.03	36.02	36.34	36.2	36.3	33	33	23	2325	2.41	6.01	82.6
AT5G35790	G6PD1 glucose-6-phosphate dehydrogenase 1	28.26	28.53	**0.27**	28.28	28.18	28.31	28.64	28.53	28.41	18	18	13	95	1.63	3.56	37.7
ATCG00120	ATPA ATP synthase subunit alpha	36.06	36.33	**0.27**	35.95	36.18	36.04	36.33	36.34	36.3	33	33	32	2296	1.75	3.89	60.6
AT4G33090	APM1, ATAPM1 aminopeptidase M1	30.8	31.09	**0.28**	30.8	30.84	30.77	31.23	31.02	31.01	54	54	54	502	1.71	3.79	65.6
AT1G12050	fumarylacetoacetase, putative	26.93	27.21	**0.28**	26.89	26.99	26.91	27.2	27.17	27.27	11	11	11	52	2.58	6.67	33.7
AT3G58730	vacuolar ATP synthase subunit D (VATD) / V-ATPase D subunit / vacuolar proton pump D subunit (VATPD)	29.54	29.83	**0.29**	29.49	29.64	29.51	29.78	29.76	29.95	17	17	17	108	1.7	3.75	51
AT5G66570	PSBO-1, OEE1, OEE33, OE33, PSBO1, MSP-1 PS II oxygen-evolving complex 1	33.74	34.03	**0.29**	33.66	33.69	33.87	34.13	33.88	34.1	22	10	10	480	1.31	2.8	61.7
AT4G10060	Beta-glucosidase, GBA2 type family protein	27.43	27.72	**0.29**	27.4	27.43	27.46	27.84	27.73	27.6	16	16	15	90	1.81	4.06	21.6
AT5G42270	VAR1, FTSH5 FtsH extracellular protease family	30.68	30.98	**0.3**	30.68	30.76	30.62	31.09	30.85	31	44	14	14	378	1.64	3.59	57.4
AT3G18890	NAD(P)-binding Rossmann-fold superfamily protein	31.01	31.3	**0.3**	31.04	30.97	31.01	31.37	31.15	31.39	36	36	36	436	1.72	3.8	71.5
AT3G56290	unknown protein	25.98	26.29	**0.31**	25.95	26.07	25.93	26.14	26.35	26.39	8	8	8	28	1.59	3.46	53.2
AT3G50820	PSBO2, PSBO-2, OEC33 photosystem II subunit O-2	35.28	35.59	**0.31**	35.3	35.29	35.26	35.66	35.5	35.62	23	23	11	1145	2.54	6.52	58
AT4G11150	TUF, emb2448, TUFF, VHA-E1 vacuolar ATP synthase subunit E1	30.92	31.23	**0.31**	30.92	30.81	31.04	31.27	31.11	31.32	26	26	18	291	1.54	3.35	85.2
AT1G30400	ATMRP1, EST1, ABCC1 multidrug resistance-associated protein 1	28.92	29.23	**0.31**	28.82	29.03	28.92	29.31	29.2	29.19	40	40	32	173	1.93	4.4	35
AT2G24270	ALDH11A3 aldehyde d	32.01	32.32	**0.31**	31.9	32.01	32.12	32.43	32.19	32.34	33	33	33	539	1.54	3.33	84.3
AT1G12840	DET3, ATVHA-C vacuolar ATP synthase subunit C (VATC) / V-ATPase C subunit / vacuolar proton pump C subunit (DET3)	30.66	30.97	**0.31**	30.75	30.55	30.68	31.11	31	30.81	29	29	29	301	1.35	2.9	76.3
AT5G27380	GSH2, GSHB glutathione synthetase 2	29.61	29.92	**0.31**	29.56	29.62	29.65	29.81	30	29.95	25	25	25	204	2.17	5.13	56.8
AT3G17240	mtLPD2 lipoamide dehydrogenase 2	28.55	28.86	**0.31**	28.64	28.43	28.57	28.9	28.81	28.87	33	18	18	125	2.05	4.76	66.1
AT1G05560	UGT1, UGT75B1 UDP-glucosyltransferase 75B1	28.38	28.7	**0.32**	28.26	28.46	28.43	28.69	28.82	28.59	11	10	10	92	1.6	3.48	25.4
AT5G58090	O-Glycosyl hydrolases family 17 protein	26.61	26.93	**0.32**	26.66	26.71	26.47	27.05	26.79	26.95	8	8	8	38	1.4	3	19.3
AT1G20620	CAT3, SEN2, ATCAT3 catalase 3	31.98	32.3	**0.32**	32.04	31.88	32.01	32.27	32.36	32.27	28	23	21	539	2.28	5.53	65.7
AT1G50250	FTSH1 FTSH protease 1	32.87	33.19	**0.32**	32.94	32.83	32.84	33.36	33.01	33.2	47	47	17	708	1.4	3	60.5
AT2G24200	Cytosol aminop	30.77	31.1	**0.33**	30.71	30.75	30.86	31.03	31.23	31.04	28	28	20	268	1.83	4.1	80.8
AT1G06410	ATTPS7, TPS7, ATTPSA trehalose-phosphatase/synthase 7	29.56	29.9	**0.33**	29.53	29.72	29.44	30.02	29.92	29.75	37	37	34	221	1.39	2.98	48.2
AT4G20360	ATRAB8D, ATRABE1B, RABE1b RAB GTPase homolog E1B	34.52	34.86	**0.34**	34.68	34.45	34.44	35.02	34.8	34.76	39	39	38	1660	1.41	3.03	77.3
AT1G13900	Purple acid phosphatases superfamily protein	25.21	25.56	**0.34**	25.11	25.3	25.24	25.4	25.73	25.54	6	6	6	26	1.42	3.05	12.2
AT1G68830	STN7 STT7 homolog STN7	28.08	28.43	**0.34**	28.09	28.16	28	28.58	28.46	28.24	19	19	19	94	1.48	3.2	33.5
AT5G64570	XYL4, ATBXL4 beta-D-xylosidase 4	29.07	29.42	**0.34**	28.98	29.17	29.06	29.39	29.49	29.37	21	20	19	179	2.19	5.2	38
AT4G25650	ACD1-LIKE, PTC52, TIC55-IV ACD1-like	26.7	27.04	**0.34**	26.75	26.59	26.76	27.04	27.08	27.01	10	10	10	59	2.38	5.87	22.9
AT2G21330	FBA1 fructose-bisphos	33.4	33.75	**0.35**	33.55	33.3	33.34	33.83	33.64	33.77	29	14	14	591	1.65	3.63	77.4
AT1G76160	sks5 SKU5 similar 5	30	30.35	**0.35**	29.97	29.94	30.09	30.22	30.52	30.31	14	14	10	225	1.61	3.51	33.6
AT1G59870	PEN3, PDR8, ATPDR8, ABCG36, ATABCG36 ABC-2 and Plant PDR ABC-type transporter family protein	31.55	31.9	**0.35**	31.78	31.47	31.4	31.81	31.96	31.93	83	83	78	649	1.31	2.8	49.3
AT3G55330	PPL1 PsbP-like protein 1	30.32	30.67	**0.35**	30.4	30.2	30.35	30.8	30.59	30.62	14	14	14	122	1.78	3.96	59.6
AT1G79440	ALDH5F1, SSADH1, SSADH aldehyde dehydrogenase 5F1	28.63	28.99	**0.35**	28.71	28.5	28.7	29.07	29.02	28.88	22	22	22	137	1.78	3.98	48.7
AT5G19220	ADG2, APL1 ADP glucose pyrophosphorylase large subunit 1	32.01	32.37	**0.35**	32.01	31.96	32.08	32.5	32.21	32.4	39	39	39	619	1.74	3.86	68.4
AT4G23100	R	30.51	30.87	**0.36**	30.62	30.45	30.47	30.93	30.97	30.71	20	20	20	211	1.66	3.66	49.4
AT2G44350	ATCS, CSY4 Citrate synthase family protein	29.86	30.22	**0.36**	29.99	29.83	29.75	30.29	30.28	30.07	24	24	24	205	1.6	3.48	60.5
AT5G49910	CPHSC70-2EAT SHOCK PROTEIN 70-2, HSC70-7, cpHsc70-2 chloroplast heat shock protein 70-2	31.7	32.06	**0.36**	31.79	31.79	31.52	32.14	31.89	32.15	63	24	24	449	1.37	2.94	61.8
AT4G38810	Calcium-binding EF-hand family protein	25.84	26.2	**0.36**	25.84	25.74	25.94	26.38	26.16	26.07	8	8	8	18	1.53	3.33	26.4
AT3G55400	OVA1 methionyl-tRNA synthetase / methionine--tRNA ligase / MetRS (cpMetRS)	28.5	28.86	**0.36**	28.59	28.48	28.44	29.03	28.77	28.78	17	17	17	108	1.7	3.76	35.9
AT1G55850	ATCSLE1, CSLE1 cellulose synthase like E1	27.43	27.8	**0.36**	27.48	27.48	27.34	27.83	27.63	27.93	22	22	22	68	1.69	3.72	33.6
AT5G22510	INV-E, At-A/N-InvE alkaline/neutral invertase	25.95	26.31	**0.37**	25.95	25.8	26.09	26.36	26.34	26.24	12	12	12	32	1.82	4.09	20.6
AT1G30360	ERD4 Early-responsive to dehydration stress protein (ERD4)	31.44	31.81	**0.37**	31.65	31.47	31.21	31.83	31.74	31.85	35	35	35	400	1.31	2.79	48.2
AT5G07020	proline-rich family protein	28.71	29.07	**0.37**	28.86	28.76	28.5	29.16	29.05	29.01	6	6	6	82	1.48	3.19	35.3
AT3G60750	Transketolase	35.72	36.08	**0.37**	35.72	35.74	35.68	36.14	35.91	36.2	48	48	35	2875	1.83	4.11	77.3
ATCG00280	PSBC photosystem II reaction center protein C	36.04	36.41	**0.37**	36.19	36.03	35.9	36.47	36.27	36.5	21	21	21	1654	1.54	3.35	36.2
AT5G48300	ADG1, APS1 ADP glucose pyrophosphorylase 1	30.85	31.22	**0.38**	30.95	30.74	30.84	31.44	31.1	31.14	25	25	25	426	1.43	3.08	56.9
AT3G23700	Nucleic acid-binding proteins superfamily	28.92	29.3	**0.38**	29.16	28.76	28.85	29.4	29.27	29.23	17	17	17	138	1.35	2.89	54.1
AT4G33010	AtGLDP1, GLDP1 glycine decarboxylase P-protein 1	35.13	35.51	**0.38**	35.01	35.26	35.11	35.64	35.39	35.51	65	65	36	2693	1.7	3.76	68.6
AT3G08740	elongation factor P (EF-P) family protein	29.74	30.13	**0.39**	29.69	29.8	29.75	30.37	30	30.02	15	15	15	133	1.45	3.13	61.9
AT5G64250	Aldolase-type TIM barrel family protein	27	27.39	**0.39**	27.17	26.78	27.04	27.45	27.39	27.32	12	12	12	50	1.48	3.2	54.9
AT4G25130	PMSR4 peptide met sulfoxide reductase 4	29.68	30.07	**0.39**	29.65	29.7	29.68	30.07	30.06	30.08	6	6	5	86	4.71	23.48	31.4
AT5G40950	RPL27 ribosomal protein large subunit 27	31.07	31.47	**0.4**	30.96	31.2	31.04	31.64	31.34	31.42	8	8	8	133	1.62	3.55	27.8
AT5G51820	PGM, ATPGMP, PGM1, STF1 phosphoglucomutase	31.28	31.68	**0.4**	31.21	31.35	31.28	31.91	31.51	31.62	33	33	33	411	1.48	3.19	63.7
AT3G26720	Glycosyl hydrolase family 38 protein	27.04	27.44	**0.4**	26.93	27.06	27.13	27.63	27.33	27.36	20	20	20	69	1.62	3.54	25.1
AT3G54050	HCEF1 high cyclic electron flow 1	33.03	33.43	**0.41**	33.07	33.01	32.99	33.64	33.2	33.45	20	20	20	793	1.45	3.13	49.9
AT4G04850	KEA3, ATKEA3 K+ efflux antiporter 3	27.66	28.06	**0.41**	27.88	27.48	27.62	28.03	28.17	27.99	15	15	15	46	1.48	3.19	22.2
AT3G08510	phospholipase C 2	29.11	29.51	**0.41**	29.08	29.2	29.04	29.69	29.35	29.49	28	28	21	149	1.68	3.7	56.8
AT5G32470	Haem oxygenase-like, multi-helical	25.53	25.93	**0.41**	25.44	25.58	25.56	25.88	25.84	26.08	12	12	12	29	2.01	4.63	25
AT5G49970	ATPPOX, PPOX pyridoxin (pyrodoxamine) 5-phosphate oxidase	27.04	27.45	**0.41**	27.05	27.05	27.03	27.61	27.32	27.42	16	16	16	66	2.04	4.74	35.5
AT3G23070	ATCFM3A, CFM3A CRM family member 3A	24.3	24.71	**0.41**	24.32	24.19	24.39	24.66	24.73	24.74	10	10	5	17	2.53	6.45	11.6
AT3G55260	HEXO1, ATHEX2 beta-hexosaminidase 1	27.49	27.9	**0.42**	27.42	27.57	27.47	27.96	27.91	27.83	13	12	12	59	2.73	7.3	28.5
AT1G42550	PMI1 plastid movement impaired1	29.59	30.01	**0.42**	29.63	29.57	29.57	30.2	29.82	30.01	41	41	41	305	1.69	3.73	60.9
AT1G78850	D-mannose binding lectin protein with Apple-like carbohydrate-binding domain	30.1	30.52	**0.42**	30.15	30.02	30.14	30.44	30.62	30.52	22	22	22	239	2.53	6.44	52.4
AT1G03230	Eukaryotic aspartyl protease family protein	30.31	30.73	**0.42**	30.5	30.28	30.15	30.86	30.78	30.55	19	19	14	241	1.39	2.99	52.3
AT4G36760	ATAPP1, APP1 aminopeptidase P1	28.33	28.75	**0.42**	28.48	28.29	28.2	28.77	28.76	28.71	16	16	16	98	2.15	5.07	38.1
AT4G35000	APX3 ascorbate peroxidase 3	30.26	30.69	**0.43**	30.32	30.15	30.32	30.95	30.55	30.57	26	26	26	216	1.4	3.01	67.6
AT3G55610	P5CS2 delta 1-pyrroline-5-carboxylate synthase 2	28.59	29.02	**0.43**	28.59	28.56	28.62	29.12	28.97	28.96	26	26	15	132	2.8	7.64	44.1
AT3G18060	transducin family protein / WD-40 repeat family protein	28.33	28.76	**0.43**	28.52	28.33	28.13	28.87	28.7	28.69	22	22	22	141	1.55	3.36	51.6
AT5G58330	la	31.37	31.81	**0.43**	31.52	31.31	31.29	32.05	31.72	31.64	26	26	26	350	1.38	2.95	61.4
AT2G26080	AtGLDP2, GLDP2 glycine decarboxylase P-protein 2	32.06	32.5	**0.43**	31.93	32.25	32.01	32.67	32.43	32.38	59	30	30	514	1.54	3.34	59.5
AT3G26060	ATPRX Q Thioredoxin superfamily protein	32.73	33.17	**0.43**	32.68	32.83	32.68	33.13	33.11	33.26	21	21	21	402	2.51	6.39	56.5
AT1G58270	ZW9 TRAF-like family protein	26.92	27.35	**0.44**	27.07	26.69	26.99	27.33	27.35	27.37	10	10	10	67	1.71	3.78	22.7
AT5G13980	Glycosyl hydrolase	28.92	29.36	**0.44**	28.89	28.89	28.97	29.24	29.36	29.47	37	37	36	168	2.4	5.97	41.3

AT1G09830	Glycinamide ribonucleotide (GAR) synthetase	27.45	27.88	**0.44**	27.55	27.39	27.4	28.08	27.69	27.88	13	13	13	73	1.61	3.51	30.8
AT4G10300	RmlC-like cupins superfamily protein	28.4	28.84	**0.44**	28.42	28.45	28.33	28.81	28.82	28.89	8	8	8	47	3.25	10	53.7
AT4G21280	PSBQ, PSBQA, PSBQ-1 photosystem II subunit QA	35.14	35.59	**0.44**	34.93	35.28	35.21	35.41	35.57	35.77	26	26	24	1208	1.37	2.95	62.3
AT5G13370	Auxin-responsive GH3 family protein	28.13	28.58	**0.44**	28.13	28.05	28.21	28.58	28.61	28.54	18	18	15	82	3.03	8.75	41.8
AT1G48030	mtLPD1 mitochondrial lipoamide dehydrogenase 1	32.16	32.6	**0.45**	32.26	32.06	32.15	32.6	32.5	32.71	40	40	25	711	2.21	5.29	74.4
AT1G72160	Sec14p-like phosphatidylinositol transfer family protein	28.76	29.21	**0.45**	29.05	28.6	28.63	29.29	29.16	29.17	26	26	22	140	1.4	3.01	63.3
AT3G12010	FUNCTIONS IN: molecular_function unknownCONTAINS InterPro DOMAIN/s: Colon cancer-associated Mic1-like	24.7	25.15	**0.45**	24.59	24.84	24.68	25.3	25.08	25.07	5	5	5	17	1.89	4.27	10.1
AT4G17300	NS1, OVA8, ATNS1 Class II aminoacyl-tRNA and biotin synthetases superfamily protein	28.57	29.02	**0.45**	28.34	28.76	28.6	29.1	29.01	28.97	16	16	16	95	1.62	3.55	30.7
AT4G10750	Phosphoenolpyruvate carboxylase family protein	25.71	26.17	**0.45**	25.85	25.46	25.84	26.28	26.1	26.12	6	6	6	20	1.49	3.22	20.7
AT1G34000	OHP2 one-helix protein 2	29.37	29.83	**0.46**	29.5	29.29	29.33	29.65	29.76	30.07	9	9	9	120	1.48	3.19	59.9
AT5G44020	HAD superfamily, subfamily IIIB acid phosphatase	31.33	31.79	**0.46**	31.3	31.42	31.27	32.04	31.77	31.56	19	19	16	348	1.44	3.1	66.5
AT1G35670	ATCDPK2, CPK11, ATCPK11, CDPK2 calcium-dependent protein kinase 2	26.29	26.75	**0.46**	26.14	26.34	26.39	26.87	26.64	26.73	9	9	5	45	2	4.6	28.3
AT3G12580	HSP70, ATHSP70 heat shock protein 70	29.17	29.63	**0.46**	29.23	29.13	29.14	29.71	29.54	29.64	54	21	18	150	2.85	7.87	75.2
AT1G54500	Rubredoxin-like superfamily protein	28.72	29.19	**0.47**	28.65	28.79	28.72	29.44	28.88	29.24	11	11	11	107	1.3	2.78	56.4
AT2G47730	ATGSTF8, ATGSTF5, GST6, GSTF8 glutathione S-transferase phi 8	32.23	32.69	**0.47**	32.38	32.15	32.15	32.89	32.63	32.56	18	17	17	380	1.67	3.67	74.9
AT4G18240	ATSS4, SSIV, SS4 starch synthase 4	27.72	28.19	**0.47**	27.53	27.93	27.71	28.18	28.14	28.25	20	20	20	94	1.75	3.87	25.7
AT3G51550	FER Malectin/receptor-like protein kinase family protein	25.72	26.19	**0.47**	25.77	25.69	25.69	26.27	26.33	25.96	10	10	10	42	1.81	4.04	15.5
AT4G25370	Double Clp-N motif protein	28.39	28.87	**0.47**	28.39	28.21	28.58	29.01	28.78	28.81	13	13	13	96	1.69	3.72	51.3
AT3G06810	IBR3 acyl-CoA dehydrogenase-related	26.72	27.19	**0.47**	26.73	26.91	26.52	27.21	27.34	27.02	14	14	14	61	1.52	3.29	23.3
AT3G48170	ALDH10A9 aldehyde dehydrogenase 10A9	27.82	28.3	**0.48**	27.79	27.82	27.85	28.36	28.26	28.28	11	11	11	70	3.79	13.72	38.2
AT1G20810	FKBP-like peptidyl-prolyl cis-trans isomerase family protein	26.38	26.86	**0.48**	26.25	26.39	26.5	26.99	26.72	26.88	10	10	10	38	1.96	4.47	37.9
AT1G80460	NHO1, GLI1 Actin-like ATPase superfamily protein	28.4	28.88	**0.48**	28.43	28.43	28.33	28.8	28.99	28.86	18	18	18	89	2.74	7.34	47.7
AT5G41790	CIP1 COP1-interactive protein 1	30.6	31.09	**0.49**	30.93	30.48	30.4	31.04	31.16	31.07	101	101	100	460	1.34	2.87	66
AT2G47400	CP12-1, CP12 CP12 domain-containing protein 1	29.88	30.37	**0.49**	29.71	29.95	29.99	30.18	30.33	30.61	7	7	7	131	1.48	3.18	55.6
AT5G39590	TLD-domain containing nucleolar protein	26.23	26.72	**0.49**	26.17	26.12	26.39	26.68	27	26.48	12	12	12	51	1.33	2.84	32.1
AT2G31170	SYCO ARATH Cysteinyl-tRNA synthetase, class Ia family protein	27.01	27.5	**0.49**	27.04	27.14	26.84	27.77	27.36	27.37	11	11	11	53	1.42	3.05	25.8
AT1G20340	DRT112, PETE2 Cupredoxin superfamily protein	31.83	32.32	**0.49**	32.1	31.76	31.61	32.48	32.18	32.3	2	2	2	177	1.36	2.91	38.9
AT4G24620	PGI1, PGI phosphoglucose isomerase 1	30.31	30.8	**0.49**	30.27	30.32	30.34	30.77	30.84	30.8	39	39	39	300	4.25	17.95	75.9
AT1G65930	cICDH cytosolic NADP+-dependent isocitrate dehydrogenase	33.34	33.84	**0.5**	33.52	33.24	33.27	33.93	33.87	33.72	43	43	35	1012	1.99	4.57	86.6
AT5G27390	Mog1/PsbP/DUF1795-like photosystem II reaction center PsbP family protein	26.14	26.64	**0.5**	25.92	26.32	26.17	26.72	26.61	26.58	6	6	6	23	1.8	4.02	37.3
AT5G42980	ATTRX3, ATH3, ATTRXH3, TRXH3, TRX3 thioredoxin 3	31.98	32.48	**0.5**	31.88	31.98	32.07	32.76	32.33	32.34	14	14	14	282	1.52	3.29	87.3
AT4G29900	ACA10, CIF1, ATACA10 autoinhibited Ca(2+)-ATPase 10	27.19	27.7	**0.51**	27.35	27.12	27.12	27.82	27.68	27.59	22	21	20	60	2.11	4.96	28.1
AT3G26450	Polyketide cyclase/dehydrase and lipid transport superfamily protein	29.76	30.27	**0.51**	29.91	29.61	29.77	30.51	30	30.29	16	14	11	203	1.4	2.99	96.7
AT3G22200	POP2 Pyridoxal phosphate (PLP)-dependent transferases superfamily protein |	31.04	31.54	**0.51**	31.09	31	31.02	31.47	31.75	31.41	21	21	21	322	2.06	4.78	64.9
AT3G06050	PRXIIF, ATPRXIIF peroxiredoxin IIF	29.46	29.97	**0.51**	29.66	29.3	29.41	30.24	29.82	29.84	12	12	12	112	1.35	2.89	68.2
AT4G12420	SKU5 Cupredoxin superfamily protein	30.59	31.1	**0.51**	30.53	30.61	30.63	31.15	31.15	31	23	23	23	265	2.97	8.46	35.6
AT1G75280	NmrA-like negative transcriptional regulator family protein	28.91	29.42	**0.51**	29.19	28.75	28.78	29.55	29.31	29.39	13	13	13	88	1.5	3.23	45.2
AT1G78900	VHA-A vacuolar ATP synthase subunit A	33.19	33.7	**0.51**	33.15	33.23	33.18	33.75	33.68	33.66	56	56	56	1086	3.81	13.88	85.9
AT5G42240	scpl42 serine carboxypeptidase-like 42	27.87	28.38	**0.51**	28.03	27.87	27.7	28.48	28.27	28.38	16	16	16	79	1.94	4.43	37.6
AT5G28050	Cytidine/deoxycytidylate deaminase family protein	28.3	28.81	**0.51**	28.22	28.26	28.41	29.04	28.64	28.75	10	10	10	90	1.77	3.94	74.1
ATCG00820	RPS19 ribosomal protein S19	29.82	30.34	**0.51**	29.82	30.05	29.6	30.55	30.17	30.3	6	6	6	93	1.41	3.02	68.5
AT5G60360	AALP, ALP aleurain-li	27.99	28.51	**0.52**	28.03	27.97	27.96	28.71	28.45	28.36	11	11	10	55	2.05	4.76	51.1
AT5G38990	Malectin/receptor-like protein kinase family protein	24.65	25.17	**0.52**	24.76	24.57	24.63	25.28	25.13	25.1	7	7	7	22	2.61	6.78	8.6
AT4G17560	Ribosomal protein L19 family protein	29.32	29.84	**0.52**	29.23	29.38	29.35	29.98	29.92	29.62	11	4	4	62	1.9	4.3	44.4
AT3G53180	glutamate-ammonia ligases;catalytics;glutamate-ammonia ligases	27.81	28.33	**0.52**	27.82	27.75	27.87	28.55	28.22	28.23	27	27	27	94	1.99	4.56	40.7
AT4G30920	Cytosol aminopeptidase family protein	29.38	29.91	**0.52**	29.17	29.62	29.36	30.08	29.75	29.88	23	16	9	114	1.5	3.24	51.1
AT3G12780	PGK1 phosphoglycerate kinase 1	35.71	36.23	**0.52**	35.84	35.71	35.57	36.44	36.11	36.15	41	41	23	2431	1.8	4.03	78.8
AT3G24170	ATGR1, GR1 |	29.99	30.52	**0.53**	29.96	29.99	30.03	30.43	30.62	30.51	24	24	24	223	3.08	9.03	57.9
AT4G11600	ATGPX6, PHGPX, LSC803, GPX6 glutathione peroxidase 6	28.7	29.22	**0.53**	28.79	28.63	28.67	29.34	29.1	29.22	8	8	8	70	2.51	6.37	43.1
AT4G04320	malonyl-CoA decarboxylase family protein	24.4	24.94	**0.53**	24.56	24.15	24.51	24.98	24.86	24.96	8	8	8	29	1.76	3.9	19.7
AT1G16880	uridylyltransferase-related	32.13	32.66	**0.53**	32.31	32	32.07	32.87	32.48	32.63	11	11	11	257	1.64	3.59	37.9
AT1G49750	Leucine-rich repeat (LRR) family protein	27.5	28.03	**0.53**	27.44	27.66	27.4	28.26	27.92	27.92	9	9	9	63	1.72	3.81	21.5
AT1G23440	Peptidase C15, pyroglutamyl peptidase I-like	26.69	27.22	**0.53**	26.9	26.54	26.63	27.27	27.32	27.08	9	9	8	40	1.8	4.01	48.8
AT1G20630	CAT1 catalase 1	28.3	28.83	**0.53**	28.43	28.24	28.23	28.75	29.02	28.73	22	9	9	91	2.04	4.73	48.6
AT4G34240	ALDH3I1, ALDH3 aldehyde dehydrogenase 3I1	26.29	26.83	**0.53**	26.32	26.27	26.28	26.91	26.89	26.68	12	12	12	39	2.67	7.07	24.5
AT3G13790	ATBFRUCT1 Glycosyl hydrolases family 32 protein	27.93	28.47	**0.54**	27.93	27.91	27.96	28.12	28.72	28.56	18	18	18	77	1.39	2.98	38.2
AT3G62030	ROC4 rotamase CYP 4	32.82	33.36	**0.54**	32.95	32.78	32.74	33.7	33.1	33.28	17	17	16	712	1.34	2.86	61.9
AT4G19410	Pectinacetylesterase family protein	30.23	30.77	**0.54**	30.51	30.07	30.11	30.89	30.8	30.63	22	22	18	256	1.54	3.34	74.2
AT5G35970	P-loop containing nucleoside triphosphate hydrolases superfamily protein	29.84	30.39	**0.54**	29.66	30.06	29.8	30.67	30.16	30.33	47	47	47	365	1.33	2.85	56.5
AT2G21410	VHA-A2 vacuolar proton ATPase A2	27.96	28.51	**0.55**	28.12	27.98	27.76	28.61	28.45	28.46	24	11	11	62	2.04	4.74	33.4
AT5G11520	ASP3, YLS4 aspartate aminotransferase 3	30.04	30.59	**0.55**	30.06	30.01	30.04	30.7	30.65	30.41	21	16	16	217	2.43	6.06	61.7
AT4G39730	Lipase/lipooxygenase, PLAT/LH2 family protein	28.13	28.68	**0.55**	27.94	28.19	28.26	28.59	28.65	28.81	4	3	3	37	2.02	4.66	27.1
AT1G18170	FKBP-like peptidyl-prolyl cis-trans isomerase family protein	27.5	28.05	**0.55**	27.34	27.52	27.62	28.28	27.97	27.9	8	8	8	48	1.74	3.87	38.5
AT5G16970	AT-AER, AER alkenal reductase	31.28	31.84	**0.56**	31.56	31.19	31.1	32.04	31.82	31.65	25	25	14	440	1.43	3.08	93
AT4G24220	VEP1, AWI31 NAD(P)-binding Rossmann-fold superfamily protein	28.55	29.11	**0.56**	28.92	28.32	28.42	29.21	29.15	28.96	20	20	20	133	1.31	2.79	58.7
AT3G52500	Eukaryotic aspartyl protease family protein	27.73	28.29	**0.56**	27.61	27.76	27.82	28.29	28.4	28.18	10	10	10	76	2.49	6.29	22.8
AT5G54960	PDC2 pyruvate decarboxylase-2	27.09	27.65	**0.56**	27.26	26.77	27.24	27.55	27.69	27.71	10	10	10	65	1.54	3.34	22.2
AT1G33590	Leucine-rich repeat (LRR) family protein	30.68	31.24	**0.56**	30.52	30.82	30.71	31.27	31.34	31.12	20	20	20	214	2.16	5.12	60
AT4G30910	Cytosol aminopeptidase family protein	25.33	25.9	**0.56**	25.49	25.23	25.28	25.89	25.91	25.89	19	8	8	24	2.63	6.89	47.3
AT5G10470	KCA1, KAC1 kinesin like protein for actin based chloroplast movement 1	29.92	30.48	**0.56**	30.06	29.86	29.86	30.62	30.36	30.47	57	57	2	339	2.29	5.55	53.3
AT2G43820	GT, UGT74F2, ATSAGT1, SGT1, SAGT1 UDP-glucosyltransferase 74F2	28.66	29.23	**0.57**	28.75	28.61	28.62	29.28	29.3	29.1	13	13	13	119	2.73	7.31	33.9
AT5G50640	CBS / octicosapeptide/Phox/Bemp1 (PB1) domains-containing protein	24.68	25.25	**0.57**	24.88	24.68	24.46	25.25	25.25	25.23	5	5	3	21	2.02	4.66	12.4
AT3G10740	ASD1, ARAF1, ARAF, ATASD1 alpha-L-arabinofuranosidase 1	27.75	28.32	**0.57**	27.77	27.8	27.68	28.29	28.2	28.48	17	17	17	91	2.5	6.36	40.4
AT5G08410	FTRA2 ferredoxin/thioredoxin reductase subunit A (variable subunit) 2	27.25	27.83	**0.58**	27.02	27.44	27.3	28.12	27.55	27.82	8	8	8	42	1.32	2.81	41.8
AT1G70290	ATTPS8, TPS8, ATTPSC trehalose-6-phosphatase synthase S8	27.35	27.93	**0.58**	27.21	27.33	27.51	27.97	28.09	27.73	21	21	14	95	1.88	4.26	33.1

AT3G63140	CSP41A chloroplast stem-loop binding protein of 41 kDa	32.81	33.39	**0.58**	32.88	32.76	32.78	33.75	33.16	33.25	30	30	30	576	1.43	3.08	73.2
AT3G61220	NAD(P)-binding Rossmann-fold superfamily protein	28.92	29.5	**0.58**	29.04	28.77	28.95	29.77	29.33	29.39	18	18	17	120	1.65	3.62	67.6
AT5G23120	HCF136 photosystem II stability/assembly factor, chloroplast (HCF136)	31.89	32.48	**0.58**	32	31.86	31.83	32.85	32.34	32.23	32	32	32	619	1.38	2.95	71.7
AT1G63770	Peptidase M1 family protein	32.14	32.72	**0.58**	32.06	32.17	32.19	32.92	32.61	32.63	66	66	66	885	2.26	5.45	79
AT3G42050	vacuolar ATP synthase subunit H family protein	30.42	31.01	**0.59**	30.62	30.32	30.31	31.28	30.93	30.83	22	22	22	249	1.6	3.49	57.4
AT3G44890	RPL9 ribosomal protein L9	32.04	32.63	**0.59**	31.71	32.24	32.18	32.82	32.43	32.65	16	16	16	289	1.36	2.92	59.4
AT4G28730	Glutaredoxin family protein	26.22	26.81	**0.6**	26.13	26.25	26.27	26.61	26.78	27.05	5	5	5	15	1.95	4.45	39.1
AT5G42650	AOS, CYP74A, DDE2 allene oxide synthase	30.05	30.65	**0.6**	30.09	29.99	30.06	30.94	30.41	30.59	33	33	33	289	1.7	3.74	68.5
AT1G76180	ERD14 Dehydrin family protein	27.43	28.03	**0.6**	27.59	27.19	27.53	27.91	28.06	28.13	8	7	7	69	1.9	4.31	48.1
AT3G48990	AMP-dependent synthetase and ligase family protein	31.92	32.52	**0.6**	32.12	31.73	31.91	32.48	32.65	32.43	26	26	26	479	2	4.6	53.1
AT4G00490	BAM2, BMY9 beta-amylase 2	26.42	27.03	**0.6**	26.28	26.31	26.69	27.24	26.81	27.04	12	12	12	57	1.53	3.33	25.1
AT1G74970	RPS9, TWN3 ribosomal protein S9	31.08	31.69	**0.61**	30.94	31.22	31.08	32.03	31.42	31.61	8	8	8	110	1.44	3.09	38.5
AT3G19170	PREP1 presequence protease 1	32.57	33.18	**0.61**	32.61	32.55	32.56	33.38	33.01	33.16	83	83	64	1350	2.28	5.53	75.3
AT3G01520	Adenine nucleotide alpha hydrolases-like superfamily protein	26.28	26.89	**0.61**	26.25	26.29	26.29	27.21	26.74	26.72	9	9	9	49	1.74	3.85	47.4
AT5G11720	Glycosyl hydrolases family 31 protein	25.63	26.24	**0.61**	25.88	25.42	25.58	26.24	26.15	26.33	15	14	14	41	1.88	4.24	21
AT4G34138	UGT73B1 UDP-glucosyl transferase 73B1	28.33	28.95	**0.62**	28.37	28.34	28.29	28.87	29.11	28.88	18	18	18	92	2.78	7.54	34.2
AT1G78620	Protein of unknown function DUF92, transmembrane	25.11	25.73	**0.62**	25.48	24.93	24.93	25.81	25.67	25.72	4	4	4	11	1.51	3.27	11.7
AT5G36160	Tyrosine transaminase family protein	26.64	27.26	**0.62**	26.58	26.55	26.8	27.17	27.3	27.33	10	10	10	58	2.6	6.76	33.1
AT1G49630	ATPREP2, PREP2 |	24.42	25.05	**0.63**	24.46	24.55	24.24	25.17	24.99	24.98	24	5	5	11	2.34	5.73	25
AT4G31500	CYP83B1, SUR2, RNT1, RED1, ATR4 cytochrome P450, family 83, subfamily B, polypeptide 1	24.41	25.04	**0.63**	24.49	24.37	24.37	25.09	25.18	24.87	6	6	6	19	2.49	6.3	17.4
AT1G23740	Oxidoreductase, zinc-binding dehydrogenase family protein	31.68	32.31	**0.63**	31.69	31.63	31.71	32.61	32.12	32.19	27	27	27	478	1.82	4.07	67.4
AT5G34850	ATPAP26, PAP26 purple acid phosphatase 26	28.8	29.43	**0.63**	28.87	28.67	28.86	29.35	29.57	29.38	19	19	19	170	2.56	6.57	56.8
AT1G16720	HCF173 high chlorophyll fluorescence phenotype 173	29.99	30.63	**0.64**	30.09	29.96	29.93	30.71	30.55	30.63	37	37	37	283	3.12	9.26	72.2
AT2G14170	ALDH6B2 aldehyde dehydrog	28.47	29.11	**0.64**	28.75	28.26	28.41	29.06	29.16	29.11	19	19	19	126	1.89	4.27	53
AT2G35840	Sucrose-6F-phosphate phosphohydrolase family protein	28.17	28.81	**0.64**	28.35	28.11	28.05	28.75	28.87	28.8	21	21	21	112	2.58	6.65	49.8
AT3G01910	SOX, AT-SO, AtSO sulfite oxidase	28.76	29.4	**0.64**	28.96	28.83	28.49	29.67	29.29	29.22	19	19	19	108	1.49	3.22	61.8
AT3G63190	RRF, HFP108, cpRRF, AtcpRRF ribosome recycling factor, chloroplast precursor	30.67	31.31	**0.64**	30.59	30.69	30.73	31.55	31.18	31.2	19	19	19	230	2.15	5.08	49.8
AT4G05160	AMP-dependent synthetase and ligase family protein	27.7	28.34	**0.64**	27.99	27.55	27.56	28.38	28.27	28.37	14	14	14	110	1.92	4.36	31.8
AT5G17170	ENH1 rubredoxin family protein	30.4	31.04	**0.64**	30.37	30.51	30.32	31.43	30.82	30.87	11	11	11	165	1.47	3.17	49.1
AT4G10120	ATSPS4F Sucrose-phosphate synthase family protein	27.34	27.98	**0.64**	27.38	27.19	27.45	28.34	27.74	27.86	26	24	24	76	1.5	3.23	31.7
AT4G25450	ATNAP8, NAP8	29.02	29.67	**0.65**	28.89	29.05	29.12	29.83	29.65	29.52	21	21	21	168	2.33	5.71	39.4
AT3G03980	NAD(P)-binding Rossmann-fold superfamily protein	26.65	27.3	**0.65**	26.89	26.72	26.34	27.59	27.12	27.2	7	6	4	33	1.39	2.99	33.7
AT1G76030	ATPase, V1 complex, subunit B protein	32.25	32.9	**0.65**	32.17	32.3	32.28	32.88	32.98	32.84	39	39	10	636	3.37	10.77	86.8
AT1G12250	Pentapeptide repeat-containing protein	28.1	28.75	**0.65**	28.39	28	27.91	29.02	28.54	28.7	12	12	12	127	1.48	3.19	67
AT3G52960	Thioredoxin superfamily protein	31.61	32.27	**0.65**	31.43	31.61	31.8	32.36	32.14	32.31	14	14	14	358	2.2	5.23	53.4
AT1G49670	NQR ARP protein (REF)	27.78	28.43	**0.65**	27.85	27.97	27.51	28.6	28.34	28.35	20	20	20	83	1.8	4.02	41.3
AT1G31190	IMPL1 myo-inositol monophosphatase like 1	28.22	28.87	**0.65**	28.17	28.27	28.21	28.89	28.93	28.79	10	10	10	58	3.67	12.83	44.2
AT2G35370	GDCH glycine decarboxylase complex H	29.36	30.02	**0.66**	29.19	29.3	29.6	30.36	29.69	30.01	7	3	3	80	1.34	2.87	57
AT3G06510	SFR2 Glycosyl hydrolase superfamily protein	27.63	28.29	**0.66**	27.65	27.6	27.66	28.46	28.12	28.28	22	22	22	86	2.55	6.53	40.4
AT1G10360	ATGSTU18, GST29, GSTU18 glutathione S-transferase TAU 18	25.29	25.96	**0.66**	25.01	25.21	25.66	25.76	25.97	26.13	5	5	5	27	1.4	3.01	22.5
ATCG00770	RPS8 ribosomal protein S8	31.49	32.15	**0.66**	31.18	31.64	31.64	32.42	31.93	32.11	12	12	12	208	1.47	3.17	73.9
AT3G62700	ATMRP10, MRP10, ABCC14 multidrug resistance-associated protein 10	28.42	29.08	**0.67**	28.86	28.17	28.22	29.04	29.07	29.14	33	33	25	120	1.38	2.96	25.2
AT3G12345	unknown protein	29.3	29.96	**0.67**	29.4	29.31	29.17	30.18	29.7	30.01	4	4	4	77	1.9	4.3	16.7
AT3G04790	Ribose 5-phosphate isomerase, type A protein	33.12	33.79	**0.67**	33.24	33.14	32.98	34.24	33.52	33.62	21	21	21	459	1.32	2.82	75.4
AT1G07110	F2KP, ATF2KP, FKFBP fructose-2,6-bisphosphatase	28.44	29.11	**0.67**	28.6	28.27	28.43	29.27	29.01	29.06	33	33	33	155	2.27	5.5	57.3
AT2G41560	ACA4 autoinhibited Ca(2+)-ATPase, isoform 4	29.49	30.17	**0.68**	29.96	29.27	29.26	30.06	30.19	30.26	46	46	32	276	1.32	2.82	47.1
AT5G16150	GLT1, PGLCT plastid	28.02	28.7	**0.68**	28.01	28	28.05	28.65	28.77	28.68	7	7	7	51	4.24	17.92	24.9
AT4G37930	SHM1, STM, SHMT1 serine transhydroxymethyltransferase 1	34.81	35.49	**0.68**	34.76	34.81	34.85	35.5	35.46	35.5	44	44	31	1683	4.65	22.62	79.7
AT1G43670	Inositol monophosphatase family protein	31.28	31.96	**0.68**	31.48	31.18	31.19	32.26	31.89	31.74	26	26	26	508	1.7	3.76	64.5
AT1G08980	ATAMI1, AMI1, ATTOC64-I, TOC64-I amidase 1	28.11	28.79	**0.68**	28.33	27.95	28.04	29.2	28.64	28.54	17	17	17	113	1.36	2.9	57.4
AT1G47128	RD21, RD21A Granulin repeat cysteine protease family protein	30.74	31.42	**0.68**	30.72	30.66	30.83	31.47	31.47	31.32	14	14	14	186	3.18	9.59	32
AT2G35410	RNA-binding (RRM/RBD/RNP motifs) family protein	29.13	29.81	**0.68**	29.09	29.12	29.18	29.99	29.7	29.75	15	15	15	146	2.73	7.29	52.6
AT3G56650	Mog1/PsbP/DUF1795-like photosystem II reaction center PsbP family protein	29.05	29.74	**0.69**	29.14	29	29.01	30.18	29.41	29.61	12	12	12	119	1.37	2.93	55.7
AT2G44920	Tetratricopeptide repeat (TPR)-like superfamily protein	29.02	29.71	**0.69**	28.77	29.29	29.02	29.51	29.69	29.95	8	8	8	113	1.6	3.49	50.4
AT1G65590	HEXO3, ATHEX1 beta-hexosaminidase 3	27.58	28.27	**0.69**	27.16	27.76	27.82	28.43	28.23	28.16	18	18	17	56	1.42	3.05	42.2
AT1G31160	HINT 2 HISTIDINE TRIAD NUCLEOTIDE-BINDING 2	25.5	26.19	**0.69**	25.21	25.7	25.59	26.23	25.94	26.41	4	4	4	23	1.59	3.46	31
AT1G02475	Polyketide cyclase/dehydrase and lipid transport superfamily protein	27.11	27.81	**0.7**	26.76	27.14	27.44	27.84	27.62	27.97	7	7	7	43	1.46	3.15	29.2
AT1G66200	ATG	30.83	31.53	**0.7**	31.06	30.72	30.72	31.76	31.43	31.4	26	22	7	298	1.92	4.37	70.2
AT5G59250	Major facilitator superfamily protein	27.81	28.51	**0.7**	27.91	27.71	27.81	28.42	28.57	28.56	7	7	7	48	3.13	9.33	12.4
AT5G62530	ALDH12A1, ATP5CDH, P5CDH aldehyde dehydrogenase 12A1	28.53	29.24	**0.71**	28.55	28.56	28.48	29.5	29.07	29.14	21	21	21	124	2.19	5.23	41
AT3G23570	alpha/beta-Hydrolases superfamily protein	25.18	25.88	**0.71**	25.27	25.34	24.91	26.27	25.61	25.77	6	6	6	17	1.39	2.98	42.7
AT3G47800	Galactose mutarotase-like superfamily protein	29.25	29.97	**0.72**	29.27	29.25	29.24	30.12	29.92	29.88	11	11	11	145	3.16	9.46	47.2
AT4G34180	Cyclase family protein	27	27.72	**0.72**	27.12	26.64	27.25	28	27.7	27.47	5	5	5	23	1.39	2.98	29
AT1G02816	Protein of unknown function, DUF538	26.27	26.99	**0.72**	26.34	26.24	26.21	26.72	27.04	27.21	3	3	2	19	2.08	4.87	21.1
AT3G63520	CCD1, ATCCD1, ATNCED1, NCED1 carotenoid cleavage dioxygenase 1	29.74	30.46	**0.72**	29.8	29.84	29.59	30.69	30.37	30.33	31	31	31	235	2.2	5.24	71
AT3G43520	Transmembrane proteins 14C	26.03	26.75	**0.73**	26.26	25.65	26.17	27.03	26.62	26.61	2	2	2	31	1.45	3.12	19.2
AT1G71500	Rieske (2Fe-2S) domain-containing protein	30.88	31.61	**0.73**	31.01	30.77	30.87	32.05	31.34	31.44	20	20	20	282	1.45	3.13	61.7
AT1G12780	UGE1, ATUGE1 UDP-D-glucose/UDP-D-galactose 4-epimerase 1	29.31	30.04	**0.73**	29.44	29.24	29.26	30.33	29.94	29.84	29	29	21	240	1.96	4.48	84.9
AT1G06690	NAD(P)-linked oxidoreductase superfamily protein	29.05	29.78	**0.73**	29.3	28.9	28.96	30.21	29.67	29.46	18	18	18	127	1.33	2.83	53.3
AT2G43945	unknown protein	27.15	27.88	**0.73**	27.06	27.14	27.24	28.19	27.66	27.77	11	6	6	48	1.9	4.31	55
AT1G18270	ketose-bisphosphate aldolase class-II family protein	30.4	31.13	**0.73**	30.71	30.22	30.26	31.17	31.11	31.09	73	73	73	378	1.97	4.51	64.7
AT4G20850	TPP2 tripeptidyl peptidase ii	31.01	31.74	**0.73**	31.21	30.91	30.91	31.92	31.63	31.66	72	72	72	724	2.24	5.4	61.2
AT5G18170	GDH1 glutamate dehydrogenase 1	27.86	28.59	**0.73**	27.59	27.96	28.02	28.56	28.58	28.62	16	11	8	57	2.23	5.36	51.3
AT3G12800	SDRB, DECR short-chain dehydrogenase-reductase B	27.87	28.61	**0.73**	27.92	27.89	27.81	28.31	28.83	28.69	12	12	12	56	2.02	4.68	33.9
AT3G14415	Aldolase-type	32.68	33.41	**0.74**	32.76	32.71	32.57	33.71	33.34	33.2	36	15	15	580	1.98	4.54	89.1

AT3G27820	ATMDAR4, MDAR4 monodehydroascorbate reductase 4	28.14	28.88	**0.74**	28.14	28.19	28.08	28.99	28.78	28.87	18	18	18	106	3.31	10.38	44.3
AT1G12900	GAPA-2 glyceraldehyde 3-phosphate dehydrogenase A subunit 2	27.27	28.01	**0.74**	27.37	27.25	27.2	28.48	27.96	27.61	26	1	1	80	1.34	2.87	72.6
ATCG00750	RPS11 ribosomal protein S11	29.4	30.14	**0.74**	29	29.7	29.5	30.16	30.21	30.05	8	8	8	121	1.58	3.45	55.1
AT5G14120	Major facilitator superfamily protein	27.88	28.62	**0.74**	28.26	27.79	27.57	28.7	28.72	28.44	9	9	9	67	1.54	3.34	19.9
AT4G39080	VHA-A3 vacuolar proton ATPase A3	30.96	31.7	**0.74**	30.95	30.93	31	31.73	31.76	31.62	37	37	24	342	3.98	15.37	42.1
AT3G54440	glycoside hydr	27.64	28.39	**0.75**	27.91	27.52	27.49	28.41	28.31	28.45	28	28	28	115	2.21	5.29	28.2
AT2G43180		23.91	24.66	**0.75**	23.68	23.88	24.16	24.6	24.75	24.62	5	5	5	14	2.14	5.04	18
AT2G04690	Pyridoxamine 5-phosphate oxidase family protein	26.1	26.85	**0.75**	26.29	26.08	25.94	26.94	26.6	27.02	8	8	8	37	1.96	4.49	43.8
AT3G48420	Haloacid dehalogenase-like hydrolase (HAD) superfamily protein	31.42	32.17	**0.75**	31.6	31.34	31.3	32.61	31.92	31.97	23	23	23	257	1.45	3.12	60.8
AT3G07470	Protein of unknown function, DUF538	25.96	26.71	**0.75**	25.51	26.25	26.13	26.53	26.88	26.73	6	4	4	26	1.4	2.99	33.7
AT2G26740	ATSEH, SEH soluble epoxide hydrolase	28.64	29.39	**0.76**	28.97	28.48	28.46	29.75	29.27	29.17	10	10	10	73	1.44	3.09	42.7
AT5G51970	GroES-like zinc-binding alcohol dehydrogenase family protein	30.92	31.69	**0.76**	31.11	30.88	30.78	31.91	31.66	31.5	25	25	25	325	2.12	4.98	88.2
AT4G21860	MSRB2 methi	27.35	28.12	**0.76**	27.2	27.26	27.59	28.3	27.87	28.18	12	12	12	85	1.92	4.36	45.5
AT5G49360	BXL1, ATBXL1 beta-xylosidase 1	31.73	32.49	**0.76**	31.81	31.57	31.8	32.5	32.53	32.44	42	42	40	572	3.13	9.31	71.6
AT4G30310	FGGY family of carbo	26.29	27.05	**0.76**	26.23	26.4	26.23	27.06	27.05	27.03	13	13	13	48	3.77	13.62	35.8
AT5G64290	DCT, DIT2.1 dicarboxylate transport 2.1	29.83	30.6	**0.76**	30.3	29.59	29.61	30.53	30.45	30.81	7	7	6	87	1.38	2.95	14
AT3G56310	Melibiase family protein	26.81	27.57	**0.77**	26.97	26.68	26.77	27.67	27.6	27.44	8	8	8	55	2.66	7.02	24.7
AT1G80380	P-loop containing nucleoside triphosphate hydrolases superfamily protein	31.55	32.32	**0.77**	31.65	31.54	31.47	32.56	32.3	32.1	23	23	23	441	2.22	5.31	67.6
AT2G35780	scpl26 serine carboxypeptidase-like 26	27.16	27.93	**0.77**	26.96	27.09	27.43	28.13	27.93	27.74	10	10	10	54	1.88	4.25	29.6
AT2G41680	NTRC NADPH-dependent thioredoxin reductase C	30.04	30.82	**0.78**	30.06	30.02	30.05	30.8	30.84	30.81	22	22	22	249	5.82	44.64	45.4
ATCG00810	RPL22 ribosomal protein L22	30.42	31.2	**0.78**	30.08	30.59	30.57	31.48	30.79	31.32	10	10	10	181	1.37	2.94	51.9
AT3G51840	ACX4, ATSCX, ATG6 acyl-CoA oxidase 4	28.88	29.67	**0.79**	29.1	28.88	28.66	29.71	29.81	29.49	14	14	14	177	2.11	4.94	54.6
AT4G19880	Glutathi	28.34	29.12	**0.79**	28.66	28.18	28.17	29.19	29.39	28.79	15	15	15	117	1.53	3.32	58.4
AT1G09010	glycoside hydrolase family 2 protein	27.09	27.88	**0.79**	27.49	26.92	26.85	28.21	27.58	27.85	17	17	17	68	1.35	2.9	26.8
AT5G04140	GLU1, GLS1, GLUS, FD-GOGAT glutamate synthase 1	35.82	36.61	**0.79**	35.94	35.85	35.67	36.44	36.62	36.78	126	126	104	3632	2.51	6.38	81.6
AT4G22890	PGR5-LIKE A PGR5-LIKE A	29.67	30.47	**0.8**	29.81	29.59	29.62	30.93	30.18	30.29	14	14	13	202	1.51	3.27	56.2
AT1G32080	membrane protein, putative	29.76	30.56	**0.8**	30.1	29.61	29.58	30.72	30.34	30.62	6	6	6	64	1.76	3.92	17
AT5G41210	ATGSTT1, GST10, GSTT1 glutathione S-transferase THETA 1	27.53	28.33	**0.8**	27.67	27.43	27.5	28.73	28.39	27.88	10	10	10	72	1.44	3.11	47.3
AT5G17380	Thiamine pyrophosphate dependent pyruvate decarboxylase family protein	29.78	30.58	**0.8**	29.97	29.71	29.65	30.45	30.71	30.57	35	35	35	225	2.54	6.5	63.1
AT1G07040	unknown protein	26.04	26.84	**0.8**	26.32	25.94	25.86	26.94	26.75	26.84	10	10	10	30	2.2	5.26	35.6
AT1G08550	NPQ1, AVDE1 non-photochemical quenching 1	29.11	29.91	**0.8**	29.12	29.15	29.06	30.11	29.77	29.85	15	15	15	140	2.77	7.49	49.4
AT3G21790	UDP-Glycosyltransferase superfamily protein	25.44	26.25	**0.81**	25.34	25.44	25.55	26.19	26.23	26.33	5	5	5	19	3.45	11.28	15.6
AT1G03220	Eukaryotic aspartyl protease family protein	28.66	29.47	**0.81**	28.92	28.57	28.48	29.47	29.56	29.37	17	12	12	108	2.32	5.67	43.6
AT2G42490	Copper amine oxidase family protein	27.44	28.25	**0.81**	27.77	27.17	27.37	28.12	28.34	28.28	21	21	21	76	1.89	4.29	35.3
AT1G68010	HPR hydroxypyruvate reductase	33.86	34.67	**0.81**	33.97	33.81	33.78	34.93	34.55	34.52	40	40	40	1232	2.29	5.57	87.8
AT2G44310	Calcium-binding EF-hand family protein	26.54	27.36	**0.82**	26.52	26.35	26.75	27.53	27.19	27.35	6	6	6	24	2.26	5.45	62.7
AT4G29010	AIM1 Enoyl-CoA hydratase/isomerase family	29.87	30.69	**0.82**	29.9	29.81	29.91	30.63	30.73	30.71	41	41	41	293	4.21	17.52	67.5
AT4G14870	SECE1 secE/sec61-gamma protein transport protein	25.66	26.48	**0.82**	25.37	25.88	25.73	26.82	26.32	26.29	4	4	4	33	1.63	3.56	34.5
AT3G10060	FKBP-like peptidyl-prolyl cis-trans isomerase family protein	28.64	29.46	**0.82**	28.82	28.58	28.51	29.86	29.22	29.29	10	10	10	94	1.68	3.69	40.9
AT3G27890	NQR NADPH:quinone oxidoreductase	28.03	28.86	**0.82**	28.18	28.02	27.9	29.41	28.66	28.5	8	8	8	64	1.32	2.81	58.2
AT4G13500	unknown protein	27.5	28.33	**0.83**	27.83	27.31	27.36	28.4	28.19	28.41	4	4	3	35	2	4.61	24
AT3G56460	GroES-like zinc-binding alcohol dehydrogenase family protein	28.17	29	**0.83**	28.28	28.11	28.12	29.06	29.06	28.89	12	12	12	115	3.35	10.64	44.8
AT3G14067	Subtilase family protein	29.25	30.08	**0.83**	29.18	29.32	29.23	30.28	29.97	29.99	19	19	19	169	2.79	7.58	40.5
AT5G46110	APE2, TPT Glucose-6-phosphate/phosphate translocator-related	31.89	32.72	**0.83**	32.35	31.83	31.49	32.75	32.67	32.75	8	8	8	218	1.52	3.29	26.3
AT3G54660	GR, EMB2360, ATGR2 glutathione reductase	30.44	31.28	**0.84**	30.57	30.35	30.41	31.3	31.26	31.28	26	26	26	279	3.63	12.48	59.3
AT2G22990	SNG1, SCPL8 sinapoylglucose 1	28.97	29.81	**0.84**	29.03	28.87	29.01	29.78	29.83	29.82	12	12	12	91	4.03	15.84	43.3
AT2G45740	PEX11D peroxin 11D	29.68	30.52	**0.84**	29.82	29.65	29.58	30.96	30.26	30.34	12	12	10	146	1.66	3.65	53.8
AT3G13450	DIN4 Transketolase family protein	26.18	27.03	**0.85**	26.53	25.96	26.05	27.42	26.84	26.82	7	7	7	28	1.47	3.18	34.1
AT2G28605	Photosystem II reaction center PsbP family protein	25.95	26.8	**0.85**	25.76	25.83	26.25	27.3	26.64	26.46	6	6	6	26	1.34	2.85	39.2
AT2G34460	NAD(P)-binding Rossmann-fold superfamily protein	29.44	30.29	**0.85**	29.49	29.37	29.45	30.48	30.13	30.26	18	18	18	198	2.84	7.8	60
AT2G30930	unknown protein	28.83	29.68	**0.85**	28.75	28.81	28.93	30.22	29.54	29.29	11	11	11	106	1.39	2.99	67.1
AT1G32470	Single hybrid motif superfamily protein	31.18	32.03	**0.85**	30.63	31.52	31.4	32.13	31.92	32.05	7	7	3	258	1.4	3.01	56
AT1G74790	catalytics	27.7	28.56	**0.86**	27.88	27.49	27.73	28.53	28.73	28.41	12	12	11	83	2.36	5.81	18.4
AT1G71810	Protein kinase superfamily protein	24.83	25.68	**0.86**	24.49	25.15	24.85	26	25.69	25.36	7	7	7	18	1.49	3.23	12.3
AT1G57770	FAD/NAD(P)-binding oxidoreductase family protein	28.09	28.95	**0.86**	27.88	28.08	28.31	29.24	28.73	28.87	21	21	21	123	1.91	4.34	53.8
AT1G06430	FTSH8 FTSH protease 8	28.93	29.79	**0.86**	29.04	28.83	28.91	29.97	29.57	29.82	42	17	16	133	2.52	6.43	64.1
AT4G18810	NAD(P)-binding Rossmann-fold superfamily protein	29.42	30.29	**0.86**	29.33	29.63	29.31	30.51	30.1	30.25	25	25	25	142	2.26	5.46	53.9
AT3G16530	Legume lectin family protein	27.18	28.04	**0.86**	27.44	26.88	27.21	28.11	28.05	27.96	9	6	6	46	2.16	5.13	53.3
AT4G24810	Protein kinase super	26.2	27.06	**0.87**	25.95	26.6	26.04	27.4	27.06	26.73	5	5	5	30	1.43	3.06	17.6
AT2G34310	unknown protein	23.54	24.41	**0.87**	23.5	23.27	23.87	24.41	24.4	24.44	5	5	5	27	2.11	4.95	31
AT5G39050	HXXXD-type acyl-transferase family protein	26.97	27.84	**0.87**	26.77	26.73	27.41	27.79	28.08	27.65	12	12	12	59	1.57	3.41	36
AT2G37760	NAD(P)-linked oxidoreductase superfamily protein	28.71	29.58	**0.87**	28.86	28.5	28.77	29.67	29.51	29.56	18	18	15	115	2.77	7.47	76.2
AT2G29360	NAD(P)-binding Rossmann-fold superfamily protein	25.73	26.61	**0.87**	26.13	25.63	25.43	26.86	26.49	26.46	7	6	4	27	1.64	3.59	34.3
AT4G32770	VTE1, ATSDX1 tocopherol cyclase, chloroplast / vitamin E deficient 1 (VTE1) / sucrose export defective 1 (SXD1)	26.35	27.22	**0.87**	26.42	26.17	26.45	27.38	27.1	27.18	13	13	13	48	2.67	7.04	35.7
AT3G08920	Rhodanese/Cell cycle control phosphatase superfamily protein	27.55	28.43	**0.88**	27.7	27.63	27.33	28.67	28.23	28.39	9	9	9	72	2.17	5.14	58.9
AT1G06570	PDS1, HPD phytoene desaturation 1	27.7	28.58	**0.89**	27.73	27.75	27.6	28.62	28.78	28.34	13	13	13	75	2.5	6.35	38.9
AT1G17290	AlaAT1 alanine aminotransferas	30.48	31.37	**0.89**	30.51	30.49	30.44	31.41	31.34	31.35	26	26	17	284	5.03	28.2	57.1
AT4G34120	LEJ1, CDCP1 Cystathionine beta-synthase (CBS) family protein	28.76	29.65	**0.89**	28.9	28.74	28.66	29.94	29.43	29.59	11	11	11	93	2.22	5.31	42
AT4G27520	ENODL2, AtENODL2 early nodulin-like protein 2	31.71	32.61	**0.9**	31.93	31.64	31.56	32.51	32.54	32.78	16	16	16	421	2.5	6.34	28.1
AT2G24820	TIC55-II translocon at the inner envelope membrane of chloroplasts 55-II	30.34	31.24	**0.9**	30.32	30.4	30.31	31.42	31.19	31.11	30	30	30	242	3.14	9.38	62.5
AT5G08740	NDC1 NAD(P)H dehydrogenase C1	27.21	28.11	**0.91**	27.4	27.21	27.01	28.46	28.03	27.85	16	16	16	55	1.89	4.28	41.8
AT1G01820	PEX11C peroxin 11c	25.92	26.83	**0.91**	26.16	25.55	26.06	26.8	26.79	26.9	9	7	7	41	2.03	4.7	43.4
AT5G59750	DHBP synthase RibB-like alpha/beta domain;GTP cyclohydrolase II	26.2	27.11	**0.91**	25.94	26.38	26.27	27.37	27.06	26.89	12	10	10	46	2.03	4.71	26.5
AT5G40370	Glutaredoxin family protein	28.46	29.38	**0.92**	28.66	28.29	28.44	29.21	29.52	29.4	9	9	9	89	2.56	6.58	87.4
AT3G58010	PGL34 plastoglobulin 34kD	27.01	27.93	**0.92**	27.01	27.11	26.92	28.39	27.8	27.6	10	10	10	36	1.7	3.75	49.7

AT1G27970	NTF2B nuclear transport factor 2B	28.81	29.73	**0.92**	28.33	29.15	28.94	29.5	29.87	29.83	6	6	6	73	1.56	3.39	68.3
AT5G54500	FQR1 flavodoxin-like quinone reductase 1	30.1	31.03	**0.93**	30.39	29.89	30.02	31.24	31.07	30.77	13	13	13	159	1.99	4.57	68.1
AT3G26070	Plastid-lipid associated protein PAP / fibrillin family protein	29.27	30.19	**0.93**	29.26	29.1	29.44	30.61	30.02	29.95	19	19	13	135	1.79	3.99	59.9
AT4G33640	unknown protein	26.69	27.62	**0.93**	26.43	26.62	27.01	28.13	27.33	27.39	3	3	3	28	1.4	3	42.1
AT3G10130	SOUL heme-binding family protein	24.24	25.17	**0.93**	24.1	24.21	24.41	25.46	24.9	25.16	5	5	5	12	2.13	5.01	18.4
AT3G14420	Aldolase-type	34.98	35.92	**0.94**	35.1	34.97	34.87	36.19	35.85	35.71	38	38	17	2257	2.4	5.97	88.3
AT4G15530	PPDK pyruvate orthoph	31.08	32.02	**0.94**	31.1	31.12	31.03	32.07	32.08	31.93	49	49	49	585	4.17	17.12	60.7
AT5G48180	NSP5 nitrile specifier protein 5	28.31	29.25	**0.94**	28.24	28.23	28.46	28.97	29.34	29.44	15	15	15	128	2.39	5.91	57.4
AT1G21670		28.55	29.5	**0.94**	28.74	28.56	28.36	29.37	29.5	29.62	29	29	28	169	2.7	7.18	46.5
AT1G17100	SOUL heme-binding family protein	28.93	29.88	**0.95**	29	28.88	28.92	30.11	29.75	29.78	10	10	10	111	2.83	7.76	49.1
AT5G65010	ASN2 asparagine synthetase 2	31.2	32.15	**0.95**	31.47	31.03	31.11	31.92	32.34	32.2	30	28	19	432	2.18	5.17	62.1
AT4G20260	ATPCAP1, PCAP1 plasma-membrane associated cation-binding protein 1	31.26	32.21	**0.95**	31.38	31.2	31.19	32.05	32.32	32.26	22	22	22	314	3.13	9.3	80.9
AT1G12240	ATBETAFRUCT4, VAC-INV Glycosyl hydrolases family 32 protein	29.68	30.64	**0.95**	29.73	29.81	29.51	30.94	30.56	30.41	17	17	14	183	2.22	5.31	29.5
AT3G52230	unknown protein	27.75	28.71	**0.96**	27.72	27.78	27.74	29.03	28.48	28.62	7	7	7	48	2.36	5.8	58.6
AT1G54100	ALDH7B4 aldehyde dehydrogenase 7B4	29.59	30.55	**0.96**	29.6	29.4	29.76	30.45	30.75	30.45	26	26	26	216	2.57	6.62	67.3
AT1G03090	MCCA methylcrotonyl-CoA carboxylase alpha chain, mitochondr	29.74	30.7	**0.96**	29.89	29.79	29.54	30.6	30.56	30.94	34	34	34	263	2.42	6.05	68.7
AT5G67370	Protein of unknown function (DUF1230)	24.29	25.25	**0.97**	24.63	24.3	23.93	25.48	25.18	25.09	2	2	2	12	1.83	4.11	13.1
AT3G23920	BAM1, BMY7, TR-BAMY beta-amylase 1	25.43	26.4	**0.97**	25.45	25.37	25.48	26.42	26.32	26.47	11	11	11	33	4.2	17.49	29
AT2G21960	unknown protein	27.43	28.4	**0.97**	26.81	27.63	27.84	28.66	28.37	28.17	12	12	12	77	1.32	2.81	37
AT5G57655	xylose isomerase family protein	30.25	31.23	**0.98**	30.38	30.09	30.29	31.19	31.32	31.18	40	40	40	291	3.23	9.9	75.1
AT4G25900	Galactose mutarotase-like superfamily protein	26.47	27.45	**0.98**	26.69	26.47	26.26	27.62	27.34	27.39	9	9	9	46	2.52	6.44	32.4
AT1G20816	unknown protein	24.07	25.05	**0.98**	23.92	24.29	24.01	25.64	24.77	24.75	4	4	4	12	1.45	3.12	29.3
AT1G21680	DPP6 N-terminal domain-like protein	28.12	29.09	**0.98**	28.32	28.04	27.98	29.11	29.1	29.07	25	24	24	106	3.13	9.31	48
AT4G02520	ATGSTF3, GST16, GSTF3 glutathione S-transferase F3	31.59	32.57	**0.99**	31.83	31.44	31.48	32.72	32.62	32.38	19	19	17	327	2.45	6.16	93.4
AT4G34030	MCCB 3-methylcrotonyl-CoA carboxylase	27.99	28.98	**0.99**	27.81	28	28.17	28.99	29.03	28.92	24	24	23	96	3.07	9	52.8
AT3G48000	ALDH2B4, ALDH2, ALDH2A aldehyde dehydrogenase 2B4	30.54	31.54	**1**	30.63	30.4	30.59	31.48	31.66	31.47	26	26	26	302	3.35	10.59	55.9
AT1G54220	Dihydrolipoamide acetyltransferase, long form protein	26.01	27.01	**1**	25.41	26.24	26.37	26.77	26.89	27.35	15	11	11	31	1.34	2.86	33.8
AT5G64260	EXL2 EXORDIUM like 2	28.39	29.39	**1**	28.77	28.15	28.25	29.61	29.32	29.24	10	10	9	65	1.97	4.5	33.4
AT1G69410	ATELF5A-3, ELF5A-3 eukaryotic elongation factor 5A-3	26.16	27.16	**1**	26.3	26.11	26.07	27.33	26.88	27.27	11	7	7	30	2.52	6.43	71.5
AT4G08850	Leucine-rich repeat receptor-like protein kinase family protein	27.45	28.45	**1**	27.71	27.13	27.51	28.26	28.44	28.66	19	19	19	66	2.09	4.9	24.4
AT1G73990	SPPA, SPPA1 signal peptide peptidase	27.98	28.98	**1.01**	28.1	27.8	28.02	29.11	28.92	28.92	21	21	21	82	3.08	9.01	32.5
AT2G35490	Plastid-lipid associated protein PAP / fibrillin family protein	29.94	30.96	**1.02**	30.08	29.91	29.84	31.16	30.91	30.81	13	13	13	170	2.92	8.18	43.6
AT4G02530	chloroplast thylakoid lumen protein	30.8	31.82	**1.02**	30.77	31.02	30.62	32.3	31.55	31.62	11	11	11	150	1.73	3.84	52.3
AT4G02620	vacuolar ATPase subunit F family protein	28.37	29.39	**1.03**	27.73	28.67	28.7	29.5	29.34	29.35	6	6	6	65	1.47	3.18	42.2
AT1G44575	NPQ4, PSBS Chlorophyll A-B binding family protein	33.38	34.4	**1.03**	33.41	33.38	33.34	34.75	34.11	34.36	11	11	11	606	2.25	5.42	40.8
AT5G44130	FLA13 FASCICLIN-like arabinogalactan protein 13 precursor	28.67	29.7	**1.03**	28.75	28.63	28.64	29.54	29.83	29.73	9	9	8	105	3.42	11.07	32
AT4G21445	unknown protein	26.98	28.02	**1.04**	27.07	27.01	26.85	28.21	27.88	27.95	4	4	4	34	3.04	8.85	37
AT2G44050	COS1 6,7-dimethyl-8-ribityllumazine synthase / DMRL synthase / lumazine synthase / riboflavin synthase	28.47	29.52	**1.05**	28.62	28.42	28.37	30.01	29.2	29.34	7	7	7	62	1.79	4.01	25.6
AT5G58270	STA1, ATATM3, ATM3 ABC transporter of the mitochondrion 3	24.88	25.93	**1.05**	24.45	25.04	25.15	26.09	25.76	25.95	14	14	14	37	1.95	4.45	28.8
AT1G10370	GST30, ATGSTU17, GST30B, ERD9 Glutathione S-transferase family protein	28.03	29.08	**1.05**	28.34	28.11	27.65	29.72	28.79	28.75	9	9	9	83	1.31	2.8	56.4
AT5G19860	Protein of unknown function, DUF538	27.37	28.42	**1.05**	26.98	27.84	27.3	28.27	28.57	28.43	6	6	6	40	1.78	3.96	40.3
AT2G17695	CONTAINS InterPro DOMAIN/s: Domain of unknown function DUF1990 (InterPro:IPR018960).	26.67	27.72	**1.05**	26.74	26.67	26.6	28.33	27.4	27.44	11	11	11	49	1.58	3.45	54.6
AT5G63620	GroES-like zinc-binding alcohol dehydrogenase family protein	26.38	27.44	**1.06**	25.86	26.44	26.85	27.26	27.52	27.53	10	10	10	43	1.61	3.52	29
AT3G23490	CYN cyanase	27.73	28.8	**1.07**	27.71	27.53	27.95	29.16	28.62	28.63	12	12	12	75	2.11	4.96	69.6
AT1G70580	AOAT2, GGT2 alanine-2-oxoglutarate aminotransferase 2	27.26	28.34	**1.07**	27.53	27.08	27.19	28.67	28.1	28.24	31	9	9	54	2.1	4.92	63.8
AT5G56870	BGAL4 beta-galactosidase 4	28.09	29.17	**1.08**	28.22	27.82	28.24	28.88	29.18	29.44	27	26	19	121	2.15	5.1	57
AT3G47430	PEX11B peroxin 11B	25.79	26.87	**1.08**	26.12	25.68	25.58	27.18	26.87	26.56	3	3	3	26	1.93	4.39	17.2
AT1G32220	NAD(P)-binding Rossmann-fold superfamily protein	28.08	29.16	**1.08**	28.22	27.85	28.16	29.53	28.99	28.96	8	8	8	62	2.11	4.94	34.5
AT3G62410	CP12-2, CP12 CP12 domain-containing protein 2	28.25	29.35	**1.09**	28.43	28.44	27.89	29.45	29.3	29.29	6	6	6	89	2.36	5.79	54.2
AT1G03600	PSB27 photosystem II family protein	31.24	32.34	**1.1**	31.46	31.22	31.05	32.69	31.91	32.42	7	7	7	160	1.89	4.28	39.7
AT3G45140	LOX2, ATLOX2 lipoxygenase 2	30.68	31.81	**1.13**	30.58	30.79	30.68	32.15	31.53	31.75	48	48	48	534	2.39	5.91	69.5
AT4G00165	Bifunctional inhibitor/lipid-transfer protein/seed storage 2S albumin superf	27.6	28.74	**1.14**	27.61	27.8	27.39	28.94	28.46	28.82	4	4	4	35	2.44	6.12	31.2
AT2G13360	AGT, AGT1, SGAT alanine:glyoxylate aminotransferase	33.87	35.01	**1.14**	33.92	33.91	33.79	35.24	34.94	34.85	27	27	27	1049	3.11	9.22	85
AT5G51070	ERD1, CLPD, SAG15 Clp ATPase	29.02	30.16	**1.14**	28.84	29.23	28.99	30.24	30.26	29.97	44	42	42	244	2.8	7.63	56.1
AT5G25940	early nodulin-related	26.16	27.3	**1.14**	25.99	26.55	25.94	27.98	27.02	26.9	4	4	4	28	1.35	2.89	43.5
AT2G25080	ATGPX1, GPX1 glutathione peroxidase 1	31.13	32.28	**1.15**	31.26	31	31.14	32.72	32.11	32.02	20	20	14	211	2.1	4.91	60.2
AT3G44300	NIT2, AtNIT2 nitrilase 2	25.2	26.35	**1.16**	25.15	25.23	25.22	26.14	26.68	26.24	10	5	4	33	2.64	6.9	33.9
AT4G20830	FAD-binding Berberine family protein	26.77	27.94	**1.16**	26.85	26.45	27.01	27.71	28.24	27.86	18	18	15	67	2.14	5.03	32.2
AT3G23400	FIB4 Plastid-lipid associated protein PAP / fibrillin family protein	31.5	32.67	**1.18**	31.65	31.39	31.46	33.05	32.4	32.57	16	16	16	329	2.31	5.65	64.1
AT4G14070	AAE15 acyl-activating enzyme 15	27.57	28.75	**1.18**	27.57	27.59	27.56	28.9	28.58	28.77	27	27	27	108	3.61	12.37	46.2
AT5G09660	PMDH2 p	33.23	34.41	**1.18**	33.41	33.06	33.22	34.87	34.07	34.28	23	23	22	796	1.98	4.56	85.6
AT1G23310	GGT1, AOAT1, GGAT1 glutamate:glyoxylate aminotransferase	34.25	35.44	**1.19**	34.3	34.23	34.22	35.53	35.4	35.38	44	44	22	1966	4.66	22.83	82.5
AT5G57170	Tautomerase/MIF superfamily protein	26.27	27.45	**1.19**	25.85	26.56	26.38	27.93	27.42	27.02	3	3	3	17	1.6	3.5	42.6
AT1G33600	Leucine-rich repeat (LRR) family protein	27.26	28.45	**1.19**	27.22	27.33	27.22	28.91	28.29	28.14	14	14	14	56	2.12	4.97	31.2
AT2G41100		25.43	26.63	**1.2**	24.69	25.51	26.09	26.74	26.6	26.54	6	6	6	30	1.36	2.91	24.6
AT2G46910	Plastid-lipid associated protein PAP / fibrillin family protein	26.64	27.84	**1.2**	26.7	26.52	26.71	28.28	27.66	27.59	10	10	10	38	2.21	5.29	46.1
AT1G78140	S-adenosyl-L-methionine-dependent methyltransferases superfamily protein	26.13	27.34	**1.21**	26.27	26.03	26.09	27.79	27.11	27.12	7	7	7	35	2.15	5.09	29.3
AT4G30690	Translation initiation factor 3 protein	25.35	26.56	**1.22**	25.41	25.27	25.36	26.81	26.23	26.65	12	7	7	23	2.62	6.84	47.7
AT1G71480	Nuclear transport factor 2 (NTF2) family protein	24.99	26.21	**1.22**	25.57	24.75	24.65	26.85	25.77	26.02	3	3	3	20	1.31	2.79	15.7
AT4G35090	CAT2 catalase 2	34.31	35.54	**1.23**	34.35	34.26	34.3	35.55	35.53	35.53	36	36	22	1427	5.9	46.79	83.5
AT3G16910	AAE7, ACN1 acyl-activating enzyme 7	28.56	29.8	**1.23**	28.53	28.53	28.63	29.91	29.62	29.86	24	24	24	159	3.68	12.87	54.5
AT1G69840	SPFH/Band 7/PHB domain-containing membrane-associated protein family	25.7	26.94	**1.24**	26.02	25.44	25.66	27.24	26.8	26.78	13	11	10	44	2.27	5.5	45.5
AT1G78830	Curculin-like (mannose-binding) lectin family protein	30.1	31.36	**1.26**	30.15	30.15	30.01	31.51	31.33	31.24	22	22	15	261	3.77	13.59	55.6
AT3G25770	AOC2 allene oxide cyclase 2	28.82	30.09	**1.27**	29.08	28.64	28.75	30.34	30.04	29.9	13	13	8	172	2.63	6.86	53
AT2G47840	Uncharacterised conserved protein ycf60	26.32	27.61	**1.29**	25.76	26.55	26.64	27.89	27.54	27.39	4	4	4	35	1.82	4.09	25.5

AT4G26530	Aldolase superfamily protein	31.45	32.75	**1.3**	31.53	31.46	31.36	33.43	32.34	32.48	25	22	22	436	1.69	3.73	79.3
AT1G27020	unknown protein	25.36	26.65	**1.3**	25.42	25.39	25.26	26.31	27.13	26.52	6	6	6	22	2.18	5.17	33.1
AT5G07440	GDH2 glutamate dehydrogenase 2 ch	31.23	32.53	**1.3**	31.54	31.05	31.1	32.6	32.62	32.38	28	28	21	411	2.76	7.43	74.9
AT3G44880	ACD1, LLS1, PAO Pheophorbide a oxygenase family protein with Rieske [2Fe-2S] domain	26	27.3	**1.3**	26.2	26.06	25.73	26.76	27.86	27.28	13	13	13	55	1.7	3.76	33.9
AT1G73650	Protein of unk	26.38	27.68	**1.31**	26.66	26.31	26.17	27.86	27.65	27.54	4	4	4	38	2.75	7.38	14.4
AT5G02940	Protein of unknown function (DUF1012)	26.86	28.17	**1.31**	26.96	26.74	26.89	28.31	27.87	28.33	20	20	16	61	2.88	7.99	32.6
AT3G47340	ASN1, DIN6, AT-ASN1 glutamine-dependent asparagine synthase 1	29.97	31.28	**1.31**	30	29.95	29.97	31.35	31.22	31.28	31	31	29	249	5.27	32.53	64
AT5G16370	AAE5 acyl activating enzyme 5	26.98	28.3	**1.31**	27.11	26.84	26.99	28.18	28.42	28.29	9	9	9	64	3.66	12.74	23
AT3G28270	Protein of unknown function (DUF677)	28.67	29.99	**1.32**	28.81	28.49	28.7	29.92	30.05	29.99	19	19	19	171	3.74	13.34	50.5
AT2G29340	NAD-dependent epimerase/dehydratase family protein	28.65	29.99	**1.35**	28.62	28.59	28.73	30.03	30.02	29.93	10	10	9	90	4.87	25.71	41
AT5G14780	FDH formate dehydrogenase	31.14	32.5	**1.37**	31.39	30.97	31.05	32.66	32.55	32.3	29	29	29	473	2.94	8.3	81.8
AT3G51600	LTP5 lipid transfer protein 5	30.22	31.61	**1.39**	29.4	30.47	30.81	31.58	31.24	32.02	4	4	4	50	1.35	2.9	25.4
AT2G25450	2-oxoglutarate (2OG) and Fe(II)-dependent oxygenase superfamily protein	30.22	31.61	**1.4**	30.15	30.11	30.38	31.41	31.68	31.74	22	22	22	290	3.35	10.62	63.5
AT1G75220	Major facilitator superfamily protein	26.71	28.11	**1.4**	26.87	26.63	26.64	28.02	28.17	28.13	8	8	6	50	3.98	15.34	17
AT3G19930	STP4, ATSTP4 sugar transporter 4	25.43	26.86	**1.43**	25.33	25.61	25.36	26.86	27.16	26.57	3	3	3	20	2.76	7.46	8.9
AT1G11260	STP1, ATSTP1 sugar transporter 1	29.5	30.94	**1.44**	29.58	29.44	29.48	31.33	30.49	31	7	7	7	128	2.36	5.82	14.4
ATCG00140	ATPH ATP synthase subunit C family protein	28.08	29.54	**1.45**	28.12	28.18	27.95	29.98	29.08	29.55	2	2	2	33	2.26	5.45	59.3
AT3G45300	IVD, ATIVD, IVDH isovaleryl-CoA-dehydrogenase	28.65	30.15	**1.49**	28.91	28.45	28.6	30.22	30.27	29.95	20	20	20	193	3.06	8.9	66.7
AT3G13750	BGAL1 beta galactosidase 1	29.27	30.78	**1.52**	29.43	29.16	29.21	30.91	30.7	30.73	32	32	30	344	3.86	14.32	55.4
AT1G21400	Thiamin diphosphate-binding fold (THDP-binding) superfamily protein	25.81	27.34	**1.52**	25.97	25.74	25.73	27.37	27.4	27.24	12	12	12	46	4.06	16.14	35.4
AT4G08870	Arginase/deacetylase superfamily protein	29.1	30.66	**1.56**	29.48	28.85	28.97	31.08	30.56	30.34	14	14	10	123	2.23	5.34	50.9
AT5G20250	DIN10 Raffinose s	30.27	31.91	**1.63**	30.38	30.14	30.3	31.89	31.95	31.88	46	46	44	514	4.64	22.57	60
AT4G33150	lysine-ketoglutarate reductase/saccharopine dehydrogenase bifunctional enzyme c	26.83	28.54	**1.71**	26.82	27.04	26.64	28.52	28.64	28.47	32	32	32	85	3.79	13.73	39.6
AT2G28190	CSD2, CZSOD2 copper/zinc superoxide dismutase 2	29.26	30.98	**1.72**	29.03	29.22	29.53	30.96	30.99	30.98	6	6	6	127	3.55	11.95	45.8
AT1G52400	BGL1, BGLU18, ATBG1 beta g	26.65	28.4	**1.75**	26.66	26.92	26.36	29.16	27.65	28.38	15	13	13	53	1.7	3.75	39.6
AT1G02930	ATGSTF6, GST1, ERD11, ATGSTF3, GSTF6, ATGST1 glutathione S-transferase 6	27.98	29.73	**1.76**	28.41	27.39	28.13	29.81	29.92	29.47	16	7	7	50	2.21	5.27	76.4
AT4G22240	Plastid-lipid associated protein PAP / fibrillin family protein	28.38	30.17	**1.79**	28.74	28.17	28.22	30.6	29.86	30.04	14	13	13	94	2.47	6.23	56.1
AT2G43400	ETFQO electron-transfer flavoprotein:ubiquinone oxidoreductase	25.17	26.99	**1.82**	24.57	25.26	25.68	26.73	27.27	26.96	17	17	17	38	2.14	5.06	46.8
AT4G01870	tolB protein-related	28.99	30.82	**1.83**	29.14	28.9	28.94	30.76	30.8	30.89	24	24	24	181	4.59	21.89	50
AT4G04020	FIB fibrillin	29.66	31.52	**1.86**	29.8	29.48	29.69	32.08	31.03	31.45	18	18	17	159	2.36	5.81	70.1
AT3G01290	SPFH/Band 7/PHB domain-containing membrane-associated protein family	29.19	31.05	**1.86**	29.67	28.72	29.17	31.16	31.1	30.89	16	16	12	125	2.53	6.47	54
AT3G22060	Receptor-like protein kinase-related family protein	27.37	29.25	**1.88**	27.79	27.18	27.14	29.63	29.07	29.06	13	13	13	69	2.58	6.66	53.2
AT4G13340	Leucine-rich repeat (LRR) family protein	26.68	28.6	**1.93**	26.4	27.25	26.39	28.49	28.91	28.42	13	13	7	38	2.4	5.97	22
AT4G20860	FAD-binding Berberine family protein	27.14	29.07	**1.93**	27.44	26.83	27.15	28.93	29.32	28.96	18	18	18	72	3.06	8.95	37.9
AT2G37770	NAD(P)-linked oxidoreductase superfamily protein	25.33	27.47	**2.14**	25.63	24.85	25.51	27.58	27.73	27.1	14	13	12	38	2.64	6.93	54.9
AT1G02920	ATGSTF7, GST11, ATGSTF8, GSTF7, ATGST11 glutathione S-transferase 7	28.74	31.28	**2.54**	29.39	28.05	28.79	31.53	31.34	30.97	19	18	10	166	2.43	6.06	89.5
AT4G19170	NCED4, CCD4 nine-cis-epoxycarotenoid dioxygenase 4	24.68	27.33	**2.65**	24.34	24.48	25.23	27.95	26.54	27.5	14	14	14	42	2.22	5.3	32.9
AT2G41040	S-adenosyl-L-methionine-dependent methyltransferases superfamily protein	24.57	27.28	**2.71**	24.65	24.02	25.03	27.74	26.93	27.15	6	6	6	30	2.68	7.11	25.9
AT2G05520	GRP-3, AT	26.14	29.04	**2.9**	24.39	27.16	26.86	29.6	28.11	29.41	4	4	4	41	1.36	2.91	47.6
AT2G05380	GRP3S glycine-rich protein 3 short isoform	28.69	31.74	**3.05**	26.66	29.81	29.6	31.59	31.62	32	5	5	5	98	1.39	2.97	64.7
AT4G30270	MERI5B, MERI-5, XTH24, SEN4 xyloglucan endotransglucosylase/hydrolase 24	28.66	32.01	**3.35**	28.66	28.59	28.74	32.07	31.97	31.99	14	14	12	259	6.44	63.62	57.6
AT5G20230	ATBCB, BCB, SAG14 blue-copper-binding protein	26.58	30.13	**3.55**	26.68	26.38	26.67	28.65	30.89	30.86	5	5	5	99	2.04	4.74	21.4
AT5G25980	TGG2, BGLU37 gluc	25.49	29.63	**4.15**	23.54	26.44	26.47	29.98	29.21	29.7	21	20	18	96	1.85	4.16	51.2
AT1G75750	GASA1 GAST1 protein homolog 1	27.67	32	**4.32**	25.24	28.76	29.02	30.76	32.61	32.61	7	7	7	138	1.47	3.16	48.5
