# Supplementary material for: A Global Proteomic Approach Sheds New Light on Potential Iron-Sulfur Client Proteins of the Chloroplastic Maturation Factor NFU3
Source: Int J Mol Sci. 2020 Oct 30;21(21):8121. doi: 10.3390/ijms21218121 (PMC7672563; doi:10.3390/ijms21218121)
Supplement: Supplementary file 1 [file ijms-21-08121-s001.zip › ijms-973833 final suppl/Berger_et_al_IJMS_Table_S2_vIII.pdf]

Table S2: List of proteins absent in *nfu3-2* (green) or absent in WT (red)

Protein IDs	Fasta headers	mean_Col	mean_nfu3	LFQ intensity col-1	LFQ intensity col-2	LFQ intensity col-3	LFQ intensity nfu3-1	LFQ intensity nfu3-2	LFQ intensity nfu3-3	number of peptides	Number of razor + unique peptides	Number of unique peptides	MS/MS count	Sequence coverage [%]
AT1G03130	PSAD-2 photosystem I subunit D-2	26.64	n.d.	26.79	26.5	26.64	n.d.	n.d.	n.d.	18	2	2	6	69.1
AT1G04620	coenzyme F420 hydrogenase family / dehydrogenase, beta subunit family	26.51	n.d.	26.5	26.75	26.62	n.d.	n.d.	n.d.	4	4	4	10	11.5
AT1G07645	ATDSI-1VOC, DSI-1VOC dessication-induced 1VOC superfamily protein	24.37	n.d.	24.15	24.6	24.37	n.d.	n.d.	n.d.	5	5	5	8	67.2
AT1G09040	unknown protein	25.94	n.d.	25.7	n.d.	25.7	n.d.	n.d.	n.d.	2	2	2	6	2.2
AT1G13220	LINC2 nuclear matrix constituent protein-related	22.82	n.d.	22.39	n.d.	22.39	n.d.	n.d.	n.d.	3	3	3	5	3.1
AT1G13690	ATE1 ATPase E1	22.42	n.d.	n.d.	22.77	22.77	n.d.	n.d.	n.d.	3	3	3	9	17.5
AT1G14710	hydroxyproline-rich glycoprotein family protein	23.47	n.d.	23.16	23.77	23.47	n.d.	n.d.	n.d.	3	3	3	8	4.8
AT1G16900	Alg9-like mannosyltransferase family	23.55	n.d.	23.38	23.72	23.55	n.d.	n.d.	n.d.	2	2	2	3	4.4
AT1G30890	Integral membrane HRF1 family protein	24.9	n.d.	24.85	24.94	24.9	n.d.	n.d.	n.d.	3	3	2	7	11.9
AT1G30910	Molybdenum cofactor sulfurase family protein	24.35	n.d.	n.d.	24.24	24.24	n.d.	n.d.	n.d.	5	5	5	11	21.1
**AT1G32550**	**FdC1 2Fe-2S ferredoxin-like superfamily protein**	**26.38**	**n.d.**	**26.2**	**26.3**	**26.25**	**n.d.**	**n.d.**	**n.d.**	**6**	**6**	**6**	**20**	**47**
AT1G32900	UDP-Glycosyltransferase superfamily protein	26.82	n.d.	26.87	26.96	26.92	n.d.	n.d.	n.d.	10	10	10	34	23.6
AT1G33040	NACA5 nascent polypeptide-associated complex subunit alpha-like protein 5	24.79	n.d.	24.84	24.72	24.78	n.d.	n.d.	n.d.	3	3	3	10	20.1
AT1G48230	nodulin MtN21 /EamA-like transporter family protein	23.3	n.d.	n.d.	23.07	23.07	n.d.	n.d.	n.d.	3	3	3	9	9.8
AT1G48760	delta-ADR delta-adaptin	24.62	n.d.	24.97	n.d.	24.97	n.d.	n.d.	n.d.	4	4	4	11	5.8
AT1G49430	LACS2, LRD2 long-chain acyl-CoA synthetase 2	25.14	n.d.	25.41	24.91	25.16	n.d.	n.d.	n.d.	10	10	10	16	21.8
AT1G49600	ATRBP47A, RBP47A RNA-binding protein 47A	24.26	n.d.	25.03	23.5	24.26	n.d.	n.d.	n.d.	5	5	5	8	15.3
AT1G54000	GDSL-like Lipase/Acylhydrolase superfamily protein	26.16	n.d.	25.84	26.14	25.99	n.d.	n.d.	n.d.	6	4	4	23	21.5
AT1G62330	O-fucosyltransferase family protein	22.6	n.d.	n.d.	22.5	22.5	n.d.	n.d.	n.d.	3	3	3	6	4
AT1G64200	VHA-E3 vacuolar H+-ATPase subunit E isoform 3	25.81	n.d.	25.36	26.4	25.88	n.d.	n.d.	n.d.	10	2	2	7	43.9
AT1G66100	Plant thionin	24.08	n.d.	24.11	24.05	24.08	n.d.	n.d.	n.d.	2	2	2	12	23.9
AT1G66270	BGLU21 Glycosyl hydrolase superfamily protein	27.17	n.d.	27.1	26.86	26.98	n.d.	n.d.	n.d.	22	15	11	28	49.4
AT1G66280	BGLU22 Glycosyl hydrolase superfamily protein	26.63	n.d.	26.72	26.55	26.64	n.d.	n.d.	n.d.	15	7	7	16	39.3
AT1G70830	MLP28 MLP-like protein 28	26.61	n.d.	26.48	26.43	26.45	n.d.	n.d.	n.d.	9	8	8	13	50.1
AT1G72410	COP1-interacting protein-related	24.36	n.d.	24.33	24.14	24.24	n.d.	n.d.	n.d.	3	3	3	6	3.4
AT1G72440	EDA25, SWA2 CCAAT-binding factor	23.98	n.d.	24.94	n.d.	24.94	n.d.	n.d.	n.d.	3	3	3	6	4.9
AT1G72970	HTH, EDA17 Glucose-methanol-choline (GMC) oxidoreductase family protein	25.73	n.d.	25.64	25.67	25.66	n.d.	n.d.	n.d.	7	7	7	11	15.5
AT1G73180	Eukaryotic translation initiation factor eIF2A family protein	24.28	n.d.	24.31	24.24	24.28	n.d.	n.d.	n.d.	3	3	3	4	6.6
AT1G75680	AtGH9B7, GH9B7 glycosyl hydrolase 9B7	25.27	n.d.	n.d.	25.23	25.23	n.d.	n.d.	n.d.	7	7	7	14	17.9
AT1G76790	O-methyltransferase family protein	25.89	n.d.	25.89	25.9	25.89	n.d.	n.d.	n.d.	5	5	4	11	19.3
AT1G77120	ADH1, ADH, ATADH, ATADH1 alcohol dehydrogenase 1	24.93	n.d.	25.11	24.58	24.84	n.d.	n.d.	n.d.	3	3	3	6	14.8
AT1G78240	TSD2, QUA2, OSU1 S-adenosyl-L-methionine-dependent methyltransferases superfam	22.63	n.d.	22.46	22.79	22.63	n.d.	n.d.	n.d.	2	2	2	6	3.2
AT1G79530	GAPCP-2 glyceraldehyde-3-phosphate dehydrogenase of plastid 2	25.09	n.d.	24.74	25.37	25.06	n.d.	n.d.	n.d.	6	4	4	12	15.2
AT2G01110	APG2, UNE3, PGA2, TATC Sec-independent periplasmic protein translocase	25.93	n.d.	n.d.	26.42	26.42	n.d.	n.d.	n.d.	3	3	3	6	10
AT2G01190	Octicosapeptide/Phox/Bem1p family protein	23.08	n.d.	22.84	23.32	23.08	n.d.	n.d.	n.d.	2	2	2	6	3.2
AT2G02850	ARPN plantacyanin	23.23	n.d.	22.69	23.76	23.23	n.d.	n.d.	n.d.	2	2	2	7	17.1
AT2G16920	UBC23, PFU2 ubiquitin-conjugating enzyme 23	23.92	n.d.	23.92	24.4	24.16	n.d.	n.d.	n.d.	3	3	3	9	4.8
AT2G17560	HMGB4, NFD4, NF	24.42	n.d.	23.82	25.02	24.42	n.d.	n.d.	n.d.	3	3	3	7	23.1
AT2G21490	LEA dehydrin LEA	25.58	n.d.	25.54	25.86	25.7	n.d.	n.d.	n.d.	3	3	3	14	37.3
AT2G23940	Protein of unknown function (DUF788)	23.05	n.d.	22.55	23.19	22.87	n.d.	n.d.	n.d.	2	2	2	10	9.8
AT2G25870	haloacid dehalogenase-like hydrolase family protein	25.22	n.d.	25.51	25.48	25.49	n.d.	n.d.	n.d.	6	6	6	13	18.5
AT2G26140	ftsh4 FTSH protease 4	24.8	n.d.	25.11	24.71	24.91	n.d.	n.d.	n.d.	5	4	4	10	9.9
AT2G27460	sec23/sec24 transport family protein	24.39	n.d.	24.44	24.4	24.42	n.d.	n.d.	n.d.	4	4	4	13	6.4
AT2G29630	PY, THIC thiaminC	28	n.d.	28.3	27.99	28.14	n.d.	n.d.	n.d.	19	19	19	50	38.7
AT2G32120	HSP70T-2 heat-shock protein 70T-2	24.84	n.d.	24.7	24.9	24.8	n.d.	n.d.	n.d.	5	5	5	9	10.8
AT2G32720	B5 #4, ATCB5-B, CB5-B cyto	22.82	n.d.	n.d.	23	23	n.d.	n.d.	n.d.	2	2	2	3	25.4
AT2G33740	CUTA Nitrogen regulatory PII-like, alpha/beta	24.57	n.d.	n.d.	24.67	24.67	n.d.	n.d.	n.d.	3	3	3	4	22.4
AT2G34790	MEE23, EDA28 FAD-binding Berberine family protein	28.36	n.d.	n.d.	28.31	28.31	n.d.	n.d.	n.d.	2	2	2	6	6.6
AT2G35920	RNA helicase family protein	22.83	n.d.	22.86	23.02	22.94	n.d.	n.d.	n.d.	5	5	5	8	5.8
AT2G36230	APG10, HISN3 Aldolase-type TIM barrel family protein	24.7	n.d.	n.d.	24.69	24.69	n.d.	n.d.	n.d.	2	2	2	6	10.9
AT2G36300	Integral membrane Yip1 family protein	24.24	n.d.	24.25	24.22	24.24	n.d.	n.d.	n.d.	2	2	2	19	12.5
AT2G37550	ASP1, AGD7 ARF-GAP domain 7	23.77	n.d.	23.63	23.91	23.77	n.d.	n.d.	n.d.	6	4	4	8	16.2
AT2G37640	ATEXPA3, ATEXP3, ATHEXP ALPHA 1.9, EXP3 Barwin-like endoglucanases superfamily protein	25.26	n.d.	25.27	25.25	25.26	n.d.	n.d.	n.d.	3	3	3	13	16
AT2G39930	ISA1, ATISA1 isoamylase 1	22.53	n.d.	22.32	22.74	22.53	n.d.	n.d.	n.d.	3	3	3	9	4.2
AT2G42320	nucleolar protein gar2-related	23.43	n.d.	23.24	23.48	23.36	n.d.	n.d.	n.d.	3	3	3	6	7.5
AT2G43610	Chitinase family protein	24.43	n.d.	24.6	24.27	24.43	n.d.	n.d.	n.d.	6	6	6	10	27.8
AT2G45730	eukaryotic initiation factor 3 gamma subunit family protein	22.54	n.d.	22.68	n.d.	22.68	n.d.	n.d.	n.d.	2	2	2	6	7.2
AT2G47000	MDR4, PGP4, ABCB4, ATPGP4 ATP binding cassette subfamily B4	24.22	n.d.	23.71	23.75	23.73	n.d.	n.d.	n.d.	16	5	4	5	14.2
AT3G02180	SP1L3 SPIRAL1-like3	24.71	n.d.	24.29	25.13	24.71	n.d.	n.d.	n.d.	2	2	2	6	20.5
AT3G02420	unknown protein	24.15	n.d.	23.7	24.25	23.97	n.d.	n.d.	n.d.	5	5	5	10	26.1
AT3G07010	Pectin lyase-like superfamily protein	24.48	n.d.	24.21	24.49	24.35	n.d.	n.d.	n.d.	4	4	3	7	10.8
AT3G09210	PTAC13 plastid transcriptionally active 13	23.52	n.d.	n.d.	23.57	23.57	n.d.	n.d.	n.d.	6	6	6	7	26.7
AT3G09270	ATGSTU8, GSTU8 glutathione S-transferase TAU 8	24.91	n.d.	n.d.	24.74	24.74	n.d.	n.d.	n.d.	4	4	4	10	22.8
AT3G10160	ATDFC, DFC, FPGS2 DHFS-FPGS homolog C	24.1	n.d.	23.62	n.d.	23.62	n.d.	n.d.	n.d.	4	4	4	3	7.2
AT3G11800	unknown protein	23.74	n.d.	23.47	24.01	23.74	n.d.	n.d.	n.d.	4	4	4	7	22
AT3G17930	unknown protein	23.88	n.d.	22.81	24.95	23.88	n.d.	n.d.	n.d.	4	4	4	12	30.5
AT3G21720	ICL isocitrate lyase	27.2	n.d.	26.94	27.09	27.01	n.d.	n.d.	n.d.	13	13	13	39	25
AT3G22520	unknown protein	24.1	n.d.	23.8	24.4	24.1	n.d.	n.d.	n.d.	6	6	6	10	12.3

AT3G28720	unknown protein	23.66	n.d.	23.55	n.d.	23.55	n.d.	n.d.	n.d.	3	3	3	9	9.6
AT3G28730	ATHMG, SSRP1, NFD, HMG high mobility group	23.51	n.d.	23.72	23.22	23.47	n.d.	n.d.	n.d.	2	2	2	6	3.9
AT3G45310	Cysteine proteinases superfamily protein	25.15	n.d.	25.23	25.01	25.12	n.d.	n.d.	n.d.	4	3	3	8	15.4
AT3G45850	P-loop containing nucleoside triphosphate hydrolases superfamily protein	23.2	n.d.	22.9	23.09	23	n.d.	n.d.	n.d.	4	4	3	6	4.8
AT3G46530	RPP13 NB-ARC domain-containing disease resistance protein	22.69	n.d.	22.88	22.67	22.78	n.d.	n.d.	n.d.	2	2	1	7	2.5
AT3G48740	SWEET11, AtSWEET11 Nodulin MtN3 family protein	26.28	n.d.	25.51	26.58	26.04	n.d.	n.d.	n.d.	5	5	5	9	17
AT3G51670	SEC14 cytosolic factor family protein / phosphoglyceride transfer family protein	23.79	n.d.	n.d.	24.21	24.21	n.d.	n.d.	n.d.	3	3	3	9	12
AT3G52850	VSR1, BP-80, ATELP, BP80, BP80B, ATELP1, ATVSR1, GFS1, VSR1;1, BP80-1;1 vacuolar sorting receptor homolog 1	22.57	n.d.	22.49	22.61	22.55	n.d.	n.d.	n.d.	6	4	4	8	12
AT3G53500	RSZ32, RS2Z32, At-RS2Z RNA-binding (RRM/RBD/RNP mot	24.86	n.d.	24.6	25.13	24.86	n.d.	n.d.	n.d.	2	2	2	5	7.4
AT3G58450	Adenine nucleotide alpha hydrolases-like superfamily protein	25.73	n.d.	25.35	25.83	25.59	n.d.	n.d.	n.d.	6	6	6	14	31.5
AT3G61820	Eukaryotic aspartyl protease family protein	26.53	n.d.	26.52	n.d.	26.52	n.d.	n.d.	n.d.	9	9	8	11	20.3
AT3G62240	RING/U-box superfamily protein	23.35	n.d.	22.92	23.16	23.04	n.d.	n.d.	n.d.	4	4	4	14	8.9
AT4G00360	CYP86A2, ATT1 cyto	24.52	n.d.	24.74	24.12	24.43	n.d.	n.d.	n.d.	5	5	3	8	11.2
AT4G00900	ECA2, ATECA2 ER-type Ca2+-ATPase 2	22.66	n.d.	n.d.	22	22	n.d.	n.d.	n.d.	5	5	5	12	6.2
AT4G04200	Microsomal signal peptidase 25 kDa subunit (SPC25)	25.38	n.d.	25.3	n.d.	25.3	n.d.	n.d.	n.d.	2	1	1	15	13.2
AT4G04460	Saposin-like aspartyl protease family protein	24.2	n.d.	23.94	n.d.	23.94	n.d.	n.d.	n.d.	2	2	2	5	5.6
AT4G14110	COP9, CSN8, FUS7, EMB143 COP9 signalosome, subunit CSN8	23.94	n.d.	n.d.	24.03	24.03	n.d.	n.d.	n.d.	2	2	2	14	13.7
**AT4G14890**	**FdC2 2Fe-2S ferredoxin-like superfamily protein**	**24.09**	**n.d.**	**23.92**	**24.29**	**24.1**	**n.d.**	**n.d.**	**n.d.**	**2**	**2**	**2**	**11**	**22.7**
AT4G16160	ATOEP16-2, ATOEP16-S Mitochondrial import inner membra	25.71	n.d.	n.d.	25.35	25.35	n.d.	n.d.	n.d.	5	5	5	9	46.6
AT4G17770	ATTPS5, TPS5 trehalose phosphatase/synthase 5	24.13	n.d.	24.01	24.24	24.13	n.d.	n.d.	n.d.	8	5	5	6	12.4
AT4G18800	ATHSGBP, ATRAB11B, ATRABA1D, RABA1d RAB GTPase homolog A1D	23.25	n.d.	23.49	n.d.	23.49	n.d.	n.d.	n.d.	8	2	2	4	40.7
AT4G21800	QQT2 P-loop containing nucleoside triphosphate hydrolases superfamily protein	23.54	n.d.	n.d.	23.24	23.24	n.d.	n.d.	n.d.	2	2	2	5	2.9
AT4G24780	Pectin lyase-like superfamily protein	24.95	n.d.	25.14	24.76	24.95	n.d.	n.d.	n.d.	2	2	2	19	11.3
AT4G27860	vacuolar iron transporter (VIT) family protein	23.01	n.d.	n.d.	23.14	23.14	n.d.	n.d.	n.d.	3	3	3	4	9.2
AT4G28220	NDB1 NAD(P)H dehydrogenase B1	24.45	n.d.	24.59	n.d.	24.59	n.d.	n.d.	n.d.	4	4	4	12	7.5
AT4G28880	ckl4 casein kinase I-like 4	23.81	n.d.	23.44	23.88	23.66	n.d.	n.d.	n.d.	2	2	1	7	6
AT4G29720	ATPAO5, PAO5 polyamine oxidase 5	24.97	n.d.	n.d.	24.64	24.64	n.d.	n.d.	n.d.	6	6	6	12	13.9
AT4G30170	Peroxidase superfamily protein	25.07	n.d.	24.61	n.d.	24.61	n.d.	n.d.	n.d.	4	4	4	5	13.5
AT4G30210	ATR2, AR2 P450 reductase 2	22.98	n.d.	22.89	23.06	22.98	n.d.	n.d.	n.d.	3	3	3	6	6.5
AT4G33400	Vacuolar import/degradation, Vid27-related protein	22.94	n.d.	22.33	n.d.	22.33	n.d.	n.d.	n.d.	2	2	2	4	4.5
AT4G34640	SQS1, ERG9 squalene synthase 1	23.85	n.d.	24.18	23.82	24	n.d.	n.d.	n.d.	4	4	4	10	17.3
AT4G34980	SLP2 subtilisin-like serine protease 2	24.31	n.d.	24.17	24.44	24.31	n.d.	n.d.	n.d.	6	6	6	9	12.3
AT4G36080	phosphotransferases, alcohol group as acceptor;binding;inositol	22.83	n.d.	n.d.	22.97	22.97	n.d.	n.d.	n.d.	9	2	2	2	2.9
AT4G37130	hydroxyproline-rich glycoprotein family protein	22.8	n.d.	n.d.	22.65	22.65	n.d.	n.d.	n.d.	2	2	2	5	4.5
AT4G37640	ACA2 calcium ATPase 2	25.22	n.d.	25.55	24.9	25.22	n.d.	n.d.	n.d.	10	9	4	17	13.3
AT4G38100	unknown protein	25.72	n.d.	n.d.	25.47	25.47	n.d.	n.d.	n.d.	4	4	4	24	29.5
AT4G38710	glycine-rich protein	25.53	n.d.	25.36	25.73	25.55	n.d.	n.d.	n.d.	5	5	5	13	19.5
AT5G01300	PEBP (phosphatidylethanolamine-binding protein) family protein	24.26	n.d.	24.36	n.d.	24.36	n.d.	n.d.	n.d.	4	4	4	11	29.6
AT5G03340	ATPase, AAA-type, CDC48 protein	25.71	n.d.	25.36	26.1	25.73	n.d.	n.d.	n.d.	59	3	2	9	69
AT5G05270	Chalcone-flavanone isomerase family protein	25.3	n.d.	25.02	25.49	25.26	n.d.	n.d.	n.d.	4	4	4	19	24.4
AT5G07340	Calreticulin family protein	23.85	n.d.	n.d.	23.83	23.83	n.d.	n.d.	n.d.	10	3	3	10	18.6
AT5G10780	CONTAINS InterPro DOMAIN/s: Uncharacterised conserved protein UCP017207	23.38	n.d.	23.4	23.36	23.38	n.d.	n.d.	n.d.	2	2	2	11	13.3
AT5G12410	THUMP domain-containing protein	23.81	n.d.	23.79	23.82	23.81	n.d.	n.d.	n.d.	5	5	5	11	19.4
AT5G13110	G6PD3 glucose-6-phosphate dehydrogenase 3	24.47	n.d.	24.51	24.42	24.46	n.d.	n.d.	n.d.	11	6	6	13	21.6
AT5G13680	ELO2, ABO1 IKI3 family protein	24.19	n.d.	23.92	24.27	24.09	n.d.	n.d.	n.d.	6	6	6	11	6.1
AT5G14060	CARAB-AK-LYS Aspartate kinase family protein	24.32	n.d.	n.d.	24.32	24.32	n.d.	n.d.	n.d.	7	6	4	7	15.3
AT5G15490	UDP-glucose 6-dehydrogenase family protein	24.42	n.d.	n.d.	24.11	24.11	n.d.	n.d.	n.d.	13	3	2	7	29.4
AT5G15610	Proteasome component (PCI) domain protein	25.94	n.d.	25.64	26.16	25.9	n.d.	n.d.	n.d.	11	9	9	8	38.3
AT5G17820	Peroxidase superfamily protein	25.02	n.d.	n.d.	25.04	25.04	n.d.	n.d.	n.d.	3	3	3	12	16.6
AT5G18800	Cox19-like CHCH family protein	24.84	n.d.	25.14	24.55	24.84	n.d.	n.d.	n.d.	3	3	3	9	34.9
AT5G20830	SUS1, ASUS1, atsus1 sucrose synthase 1	22.92	n.d.	22.79	n.d.	22.79	n.d.	n.d.	n.d.	3	3	2	7	4.5
AT5G22470	NAD+ ADP-ribosyltransferases;NAD+ ADP-ribosyltransferases	25.71	n.d.	25.68	25.84	25.76	n.d.	n.d.	n.d.	8	8	8	16	14.9
AT5G41600	BTI3, RTNLB4 VIRB2-interacting protein 3	25.24	n.d.	25.33	25.15	25.24	n.d.	n.d.	n.d.	5	3	3	6	22.2
AT5G42870	ATPAH2, PAH2 phosphatidic acid phosphohydrolase 2	23.23	n.d.	23.08	23.41	23.24	n.d.	n.d.	n.d.	4	4	3	8	7.2
AT5G43600	ATAAH-2, UAH ureidoglycolate amidohydrolase	23.89	n.d.	23.36	24.24	23.8	n.d.	n.d.	n.d.	5	5	5	11	12
AT5G44310	Late embryogenesis abundant protein (LEA) family protein	22.31	n.d.	22.39	22.24	22.31	n.d.	n.d.	n.d.	3	2	2	3	12.9
AT5G45550	Mob1/phocein family protein	28.74	n.d.	n.d.	28.46	28.46	n.d.	n.d.	n.d.	2	2	2	17	14.4
AT5G46790	PYL1, RCAR12 PYR1-like 1	25.39	n.d.	25.6	25.18	25.39	n.d.	n.d.	n.d.	7	6	6	12	29.9
AT5G48485	DIR1 Bifunctional inhibitor/lipid-transfer protein/seed storage 2S albumin superfamily protein	25.21	n.d.	25.37	n.d.	25.37	n.d.	n.d.	n.d.	2	2	2	6	45.1
AT5G48580	FKBP15-2 FK506- and rapamycin-binding protein 15 kD-2	25.28	n.d.	25.01	25.21	25.11	n.d.	n.d.	n.d.	5	5	3	9	30.1
AT5G51750	ATSBT1.3, SBT1.3 subtilase 1.3	24.62	n.d.	24.71	24.78	24.74	n.d.	n.d.	n.d.	5	5	5	10	9.7
AT5G51830	pfkB-like carbohydrate kinase family protein	24.05	n.d.	23.58	n.d.	23.58	n.d.	n.d.	n.d.	5	3	3	7	22.4
AT5G55920	OLI2 S-adenosyl-L-methionine-dependent methyltransferases superfamily protein	24.4	n.d.	24.2	24.6	24.4	n.d.	n.d.	n.d.	5	5	5	5	8.2
AT5G56020	Got1/Sft2-like vescicle transport protein family	23.26	n.d.	n.d.	22.98	22.98	n.d.	n.d.	n.d.	2	2	2	5	14.3
AT5G57800	FLP1, YRE, CER3, WAX2 Fatty acid hydroxylase superfamily	23.26	n.d.	24.15	n.d.	24.15	n.d.	n.d.	n.d.	2	2	2	3	4.3
AT5G58230	MSI1, MEE70, ATMSI1 Transducin/WD40 repeat-like superfamily protein	24.4	n.d.	24.22	24.59	24.4	n.d.	n.d.	n.d.	4	4	4	10	13.4
AT5G58440	SNX2a sorting nexin 2A	24.37	n.d.	24.11	24.63	24.37	n.d.	n.d.	n.d.	7	7	5	14	12.9
AT5G59090	ATSBT4.12, SBT4.12 subtil	26.01	n.d.	n.d.	25.74	25.74	n.d.	n.d.	n.d.	4	4	4	9	7.7
AT5G61130	PDCB1 plasmodesmata callose-binding protein 1	25.76	n.d.	25.72	25.79	25.76	n.d.	n.d.	n.d.	3	3	3	17	25.4
AT5G65260	RNA-binding (RRM/RBD/RNP motifs) family protein	24.32	n.d.	n.d.	24.37	24.37	n.d.	n.d.	n.d.	3	3	3	7	24.5
AT5G65930	ZWI, PKCBP, KCBP kinesin-like calmodulin-binding protein (ZWICHEL)	23.28	n.d.	23.83	22.74	23.28	n.d.	n.d.	n.d.	2	2	2	5	2.9
AT3G15790	MBD11, ATMBD11 methyl-CPG-binding domain 11	n.d.	22.21	n.d.	n.d.	n.d.	n.d.	22.39	22.03	4	3	3	7	24
AT1G67690	Zincin-like metalloproteases family protein	n.d.	22.63	n.d.	n.d.	n.d.	n.d.	22.69	22.56	2	2	2	7	3.1
AT5G59730	ATEXO70H7, EXO70H7 exocyst subunit exo70 family protein H7	n.d.	23.15	n.d.	n.d.	n.d.	n.d.	22.98	23.32	4	4	4	7	10.1
AT4G02050	STP7 sugar transporter protein 7	n.d.	23.26	n.d.	n.d.	n.d.	n.d.	23.09	23.44	2	2	2	5	4.9

AT4G33630	EX1 Protein of unknown function (DUF3506)	n.d.	23.15	n.d.	n.d.	n.d.	22.88	23.1	23.46	4	4	4	4	8
AT5G03070	IMPA-9 importin alpha isoform 9	n.d.	23.2	n.d.	n.d.	n.d.	23.64	23.11	22.83	2	2	2	5	5
AT3G22220	hAT transposon superfamily	n.d.	23.11	n.d.	n.d.	n.d.	n.d.	23.15	23.07	2	2	2	6	3.7
AT4G24490	RGTA1, ATRGTA1 RAB geranylgeranyl transferase alpha subunit 1	n.d.	22.68	n.d.	n.d.	n.d.	21.87	23.5	22.67	3	3	3	7	5.2
AT3G25700	Eukaryotic aspartyl protease family protein	n.d.	23.67	n.d.	n.d.	n.d.	n.d.	23.71	23.64	3	3	3	4	9.5
AT4G18670	Leucine-rich repeat (LRR) family protein	n.d.	23.82	n.d.	n.d.	n.d.	23.95	23.73	23.77	4	2	2	7	4.9
AT5G12290	DGS1 dgd1 suppressor 1	n.d.	24.16	n.d.	n.d.	n.d.	24.5	23.81	n.d.	2	2	2	10	5.1
AT5G10050	NAD(P)-binding Rossmann-fold superfamily protein	n.d.	23.99	n.d.	n.d.	n.d.	n.d.	23.96	24.01	4	4	4	12	19.7
AT1G33610	Leucine-rich repeat (LRR) family protein	n.d.	23.76	n.d.	n.d.	n.d.	n.d.	24.03	23.49	2	2	2	5	3.1
AT4G23570	SGT1A phosphatase-related	n.d.	24.2	n.d.	n.d.	n.d.	23.56	24.17	24.88	4	3	3	8	16.9
AT3G59040	Tetratricopeptide repeat (TPR)-like superfamily protein	n.d.	24.16	n.d.	n.d.	n.d.	n.d.	24.18	24.14	4	4	4	7	10.5
AT5G47540	Mo25 family protein	n.d.	24.47	n.d.	n.d.	n.d.	n.d.	24.19	24.75	5	5	5	11	13.1
AT5G16990	Zinc-binding dehydrogenase family protein	n.d.	24.88	n.d.	n.d.	n.d.	25.41	24.35	n.d.	13	3	3	12	47.8
AT1G51800	Leucine-rich repeat protein kinase family protein	n.d.	24	n.d.	n.d.	n.d.	23.26	24.45	24.27	7	5	5	8	10.2
AT1G60160	Potassium transporter family protein	n.d.	24.71	n.d.	n.d.	n.d.	25.18	24.46	24.49	2	2	2	7	3.3
AT5G66200	ARO2 armadillo repeat only 2	n.d.	23.94	n.d.	n.d.	n.d.	23.39	24.49	n.d.	6	6	6	9	13.1
AT2G37790	NAD(P)-linked oxidoreductase superfamily protein	n.d.	24.74	n.d.	n.d.	n.d.	24.97	24.52	n.d.	7	4	4	7	33.1
AT4G35360	Uncharacterised conserved protein (UCP030210)	n.d.	24.44	n.d.	n.d.	n.d.	24.51	24.58	24.23	4	3	3	14	12.3
AT1G27650	ATU2AF35B, U2AF35B Zinc finger C-x8-C-x5-C-x3-H type family protein	n.d.	25	n.d.	n.d.	n.d.	25.44	24.64	24.92	3	3	3	7	7.8
AT1G29690	CAD1 MAC/Perforin domain-containing protein	n.d.	24.61	n.d.	n.d.	n.d.	n.d.	24.65	24.56	5	5	5	11	15.3
AT1G17170	ATGSTU24, GST, GSTU24 glutathione S-transferase TAU 24	n.d.	24.32	n.d.	n.d.	n.d.	23.77	24.65	24.53	5	3	3	14	17.4
AT3G27110	Peptidase family M48 family protein	n.d.	25.13	n.d.	n.d.	n.d.	25.52	24.74	n.d.	2	2	2	6	6.7
AT4G14130	XTR7, XTH15 xyloglucan endotransglucosylase/hydrolase 15	n.d.	23.44	n.d.	n.d.	n.d.	22.11	24.77	n.d.	3	3	2	4	10.7
AT1G71720	Nucleic acid-binding proteins superfamily	n.d.	25.03	n.d.	n.d.	n.d.	25.28	24.79	n.d.	11	11	11	7	33.8
AT2G44870	unknown protein	n.d.	24.71	n.d.	n.d.	n.d.	n.d.	24.81	24.61	4	4	4	6	19
AT3G53700	MEE40 Pentatricopeptide repeat (PPR) superfamily protein	n.d.	24.87	n.d.	n.d.	n.d.	24.92	24.81	n.d.	6	6	6	8	10.2
AT4G32940	GAMMA-VPE, GAMMAVPE gamma vacuolar processing enzyme	n.d.	25.03	n.d.	n.d.	n.d.	n.d.	24.84	25.23	2	2	2	4	7.1
AT2G45220	Plant invertase/pectin methylesterase inhibitor superfamily	n.d.	25.06	n.d.	n.d.	n.d.	n.d.	25.04	25.08	5	5	5	11	12.7
AT3G60130	BGLU16 beta glucosidase 16	n.d.	24.81	n.d.	n.d.	n.d.	24.57	25.05	n.d.	3	3	3	8	8
AT5G54310	NEV, AGD5 ARF-GAP domain 5	n.d.	25.3	n.d.	n.d.	n.d.	n.d.	25.06	25.54	3	3	3	10	5.2
AT4G01897	unknown protein	n.d.	25.23	n.d.	n.d.	n.d.	25.24	25.17	25.27	5	5	5	6	53.3
AT5G02560	HTA12 histone H2A 12	n.d.	25.32	n.d.	n.d.	n.d.	25.53	25.25	25.19	2	2	2	6	17.6
AT4G25810	XTR6, XTH23 xyloglucan endotransglycosylase 6	n.d.	25.52	n.d.	n.d.	n.d.	25.96	25.3	25.3	5	4	4	13	21
AT5G52190	Sugar isomerase (SIS) family protein	n.d.	25.1	n.d.	n.d.	n.d.	n.d.	25.32	24.89	2	2	2	6	10.1
AT3G30775	ERD5, PRODH, AT-POX, ATPOX, ATPDH, PRO1 Methylenetetrahydrofolate reductase family protein	n.d.	25.52	n.d.	n.d.	n.d.	25.95	25.32	25.28	4	4	4	5	14.2
AT1G30730	FAD-binding Berberine family protein	n.d.	25.32	n.d.	n.d.	n.d.	25.24	25.45	25.25	15	8	8	13	30.2
AT5G44120	CRA1, ATCRA1, CRU1 RmlC-like cupins superfamily protein	n.d.	24.97	n.d.	n.d.	n.d.	n.d.	25.49	24.45	4	4	4	5	14.8
AT1G56140	Leucine-rich repeat transmembrane protein kinase	n.d.	25.41	n.d.	n.d.	n.d.	25.29	25.52	n.d.	5	5	2	9	5.7
AT1G28600	GDSL-like Lipase/Acylhydrolase superfamily protein	n.d.	25.37	n.d.	n.d.	n.d.	25.42	25.55	25.14	6	6	6	13	29.9
AT4G31870	ATGPX7, GPX7 glutathione peroxidase 7	n.d.	26.31	n.d.	n.d.	n.d.	27.43	25.56	25.92	11	5	5	9	46.4
AT3G51000	alpha/beta-Hydrolases superfamily protein	n.d.	25.54	n.d.	n.d.	n.d.	25.6	25.58	25.43	4	4	4	6	19.8
AT2G26560	PLP2, PLA IIA, PLA2A phospholipase A 2A	n.d.	25.71	n.d.	n.d.	n.d.	25.87	25.66	25.59	4	4	4	12	21.1
AT1G52000	Mannose-binding lectin superfamily protein	n.d.	26.56	n.d.	n.d.	n.d.	27.3	25.86	26.51	12	12	12	25	27.5
AT4G27160	SESA2, AT2S2 seed storage albumin 2	n.d.	25.68	n.d.	n.d.	n.d.	n.d.	25.99	25.37	3	3	3	8	18.3
AT4G16690	ATMES16, MES16 methyl esterase 16	n.d.	26.35	n.d.	n.d.	n.d.	26.6	26.44	26.01	3	3	3	10	14.1
AT2G29310	NAD(P)-binding Rossmann-fold superfamily protein	n.d.	26.84	n.d.	n.d.	n.d.	27.19	26.49	n.d.	5	2	1	7	16.2
AT3G28220	TRAF-like family protein	n.d.	27.36	n.d.	n.d.	n.d.	28.05	26.66	27.38	10	10	10	25	29.2
AT3G46280	protein kinase-related	n.d.	26.37	n.d.	n.d.	n.d.	25.82	26.69	26.59	7	7	6	10	17.4
AT5G57560	TCH4, XTH22 Xyloglucan endotransglucosylase/hydrolase family protein	n.d.	26.98	n.d.	n.d.	n.d.	27.23	26.88	26.83	8	7	6	23	31.3
AT5G64380	Inositol monophosphatase family protein	n.d.	26.35	n.d.	n.d.	n.d.	26.48	27.08	25.5	10	10	10	19	32.7
AT4G21620	glycine-rich protein	n.d.	27.17	n.d.	n.d.	n.d.	26.69	27.16	27.65	2	2	2	17	27.6
AT5G09440	EXL4 EXORDIUM like 4	n.d.	27.76	n.d.	n.d.	n.d.	28.12	27.6	27.55	7	6	6	18	25.9
AT5G23760	Copper transport protein family	n.d.	22.77	n.d.	n.d.	n.d.	22.27	n.d.	23.28	2	2	2	5	22.3
AT2G42530	COR15B cold regulated 15b	n.d.	23.6	n.d.	n.d.	n.d.	22.31	n.d.	24.89	3	3	3	5	24.8
AT4G36850	PQ-loop repeat family protein / transmembrane family protein	n.d.	23.42	n.d.	n.d.	n.d.	22.92	n.d.	23.92	3	3	3	3	7.4
AT3G08960	ARM repeat superfamily protein	n.d.	23.08	n.d.	n.d.	n.d.	23.12	n.d.	23.04	2	2	2	4	2.8
AT5G64280	DiT2.2 dicarboxylate transporter 2.2	n.d.	23.51	n.d.	n.d.	n.d.	23.62	n.d.	23.41	3	2	2	7	7.7
AT3G45770	Polyketide synthase, enoylreductase family protein	n.d.	24.12	n.d.	n.d.	n.d.	23.96	n.d.	24.29	2	2	2	10	8.3
AT5G02310	PRT6 proteolysis 6	n.d.	24.14	n.d.	n.d.	n.d.	24.05	n.d.	24.24	4	4	4	7	2.2
AT5G56600	PFN3, PRF3 profilin 3	n.d.	24.5	n.d.	n.d.	n.d.	24.28	n.d.	24.73	2	2	2	21	13.1
AT1G49410	TOM6 translocase of the outer mitochondrial membrane 6	n.d.	24.43	n.d.	n.d.	n.d.	24.72	n.d.	24.15	2	2	2	4	48.1
AT2G01918	PQL3 PsbQ-like 3	n.d.	24.68	n.d.	n.d.	n.d.	24.88	n.d.	24.49	2	2	2	7	12.3
AT2G01600	ENTH/ANTH/VHS superfamily protein	n.d.	24.96	n.d.	n.d.	n.d.	24.91	n.d.	25.01	3	3	3	7	8.1
AT3G16220	Predicted eukaryotic LigT	n.d.	24.54	n.d.	n.d.	n.d.	25.05	n.d.	24.03	2	2	2	4	14.8
AT5G57460	unknown protein	n.d.	25.82	n.d.	n.d.	n.d.	25.59	n.d.	26.04	5	5	5	5	9.6
AT3G53470	unknown protein	n.d.	25.62	n.d.	n.d.	n.d.	25.76	n.d.	25.48	3	3	3	4	25.2
AT1G19530	unknown protein	n.d.	25.55	n.d.	n.d.	n.d.	25.79	n.d.	25.31	4	4	4	10	43.6
AT5G52100	crr1 Dihydrodipicolinate reductase, bacterial/plant	n.d.	25.45	n.d.	n.d.	n.d.	25.95	n.d.	24.95	5	5	5	8	24.8

NB: proteins were considered as absent if they are not detected in 3 replicates of one genotype and if they are detected in at least 2 replicates of the other genotype. n.d., not detected
