# Supplementary material for: A Global Proteomic Approach Sheds New Light on Potential Iron-Sulfur Client Proteins of the Chloroplastic Maturation Factor NFU3
Source: Int J Mol Sci. 2020 Oct 30;21(21):8121. doi: 10.3390/ijms21218121 (PMC7672563; doi:10.3390/ijms21218121)
Supplement: Supplementary file 1 [file ijms-21-08121-s001.zip › ijms-973833 final suppl/Berger_et_al_IJMS_Table_S3_vIII.pdf]

Table S3: detailed localization of variant proteins between *nfu3-2* and WT (NPAS = normalized protein abundance score)

Type of data	Protein ID	NPAS	HCM location	NPAS min	NPAS max
under represented in *nfu3-2*	**AT1G02500**	7.01e-05	cytosol	1.99413e-05	0.000246188
under represented in *nfu3-2*	**AT1G05510**	7.3e-05	cytosol	3.6984e-06	0.00144232
under represented in *nfu3-2*	**AT1G08200**	8,00E-05	cytosol	3.44786e-05	0.000185741
under represented in *nfu3-2*	**AT1G09310**	0.000788	cytosol	0.000167365	0.00370935
under represented in *nfu3-2*	**AT1G10670**	5.51e-05	cytosol	1.67738e-05	0.000181179
under represented in *nfu3-2*	**AT1G10840**	0.000159	cytosol	6.20022e-05	0.000408937
under represented in *nfu3-2*	**AT1G11660**	4.73e-05	cytosol	1.81026e-05	0.000123409
under represented in *nfu3-2*	**AT1G14320**	0.000125	cytosol	3.30566e-05	0.000473918
under represented in *nfu3-2*	**AT1G18540**	0.000341	cytosol	9.45541e-05	0.00123275
under represented in *nfu3-2*	**AT1G20950**	6.97e-05	cytosol	2.34796e-05	0.000207171
under represented in *nfu3-2*	**AT1G22300**	0.000821	cytosol	0.00032099	0.0021014
under represented in *nfu3-2*	**AT1G36730**	2.07e-05	cytosol	8.99613e-06	4.76745e-05
under represented in *nfu3-2*	**AT1G43170**	0.00052	cytosol	9.47542e-05	0.00285679
under represented in *nfu3-2*	**AT1G52100**	9.62e-06	cytosol	2.24861e-06	4.1163e-05
under represented in *nfu3-2*	**AT1G53310**	0.000142	cytosol	4.39562e-05	0.000457982
under represented in *nfu3-2*	**AT1G56070**	0.00208	cytosol	0.000679831	0.00634887
under represented in *nfu3-2*	**AT1G77760**	5.41e-06	cytosol	2.3782e-06	1.2329e-05
under represented in *nfu3-2*	**AT1G78570**	5.91e-05	cytosol	2.00961e-05	0.000174052
under represented in *nfu3-2*	**AT1G79690**	0.00015	cytosol	4.88191e-05	0.000459944
under represented in *nfu3-2*	**AT2G17840**	7.69e-05	cytosol	3.63325e-05	0.000162751
under represented in *nfu3-2*	**AT2G18020**	0.000355	cytosol	0.000122024	0.00103553
under represented in *nfu3-2*	**AT2G27530**	0.000275	cytosol	0.000108817	0.000695879
under represented in *nfu3-2*	**AT2G27860**	8.57e-05	cytosol	3.53579e-05	0.000207628
under represented in *nfu3-2*	**AT2G29560**	2.25e-05	cytosol	7.53912e-06	6.7423e-05
under represented in *nfu3-2*	**AT2G31390**	0.000196	cytosol	4.74584e-05	0.000809757
under represented in *nfu3-2*	**AT2G34480**	0.000653	cytosol	0.000226599	0.00187892
under represented in *nfu3-2*	**AT2G36880**	0.000262	cytosol	6.26158e-05	0.00109937
under represented in *nfu3-2*	**AT2G41740**	8.12e-05	cytosol	2.47912e-05	0.000265969
under represented in *nfu3-2*	**AT3G01420**	4.01e-05	cytosol	4.13648e-06	0.000389544
under represented in *nfu3-2*	**AT3G06650**	7.86e-05	cytosol	2.59746e-05	0.000237988
under represented in *nfu3-2*	**AT3G09200**	0.000837	cytosol	0.000364214	0.00192417
under represented in *nfu3-2*	**AT3G09820**	0.000452	cytosol	0.000199345	0.00102468
under represented in *nfu3-2*	**AT3G11710**	0.000184	cytosol	8.29933e-05	0.000407629
under represented in *nfu3-2*	**AT3G11830**	0.000195	cytosol	6.86709e-05	0.000552478
under represented in *nfu3-2*	**AT3G12110**	5.91e-05	cytosol	1.11228e-05	0.000313992
under represented in *nfu3-2*	**AT3G16450**	0.000272	cytosol	4.42591e-05	0.00166823
under represented in *nfu3-2*	**AT3G17820**	0.000117	cytosol	2.88882e-05	0.000472055
under represented in *nfu3-2*	**AT3G25230**	0.000169	cytosol	6.95009e-05	0.00040982
under represented in *nfu3-2*	**AT3G51160**	6.17e-05	cytosol	2.24529e-05	0.000169736
under represented in *nfu3-2*	**AT3G53750**	5.88e-05	cytosol	1.32073e-05	0.000261624
under represented in *nfu3-2*	**AT3G55620**	6.31e-05	cytosol	2.5675e-05	0.000155276
under represented in *nfu3-2*	**AT3G57290**	0.000216	cytosol	9.04409e-05	0.000517391
under represented in *nfu3-2*	**AT4G02450**	0.00143	cytosol	0.000621026	0.00328192
under represented in *nfu3-2*	**AT4G10480**	0.000398	cytosol	0.000153156	0.00103394
under represented in *nfu3-2*	**AT4G11820**	4.96e-05	cytosol	1.29235e-05	0.000190175
under represented in *nfu3-2*	**AT4G13930**	0.000714	cytosol	0.00025855	0.00197316
under represented in *nfu3-2*	**AT4G14960**	4.14e-05	cytosol	5.59463e-06	0.000305972
under represented in *nfu3-2*	**AT4G15000**	0.000348	cytosol	8.76595e-05	0.00138398
under represented in *nfu3-2*	**AT4G20890**	4.29e-05	cytosol	6.67594e-06	0.000275302
under represented in *nfu3-2*	**AT4G31120**	4.29e-05	cytosol	1.56757e-05	0.000117183
under represented in *nfu3-2*	**AT4G31180**	8.56e-05	cytosol	3.35626e-05	0.0002181
under represented in *nfu3-2*	**AT4G31790**	2.97e-05	cytosol	1.01207e-05	8.72726e-05
under represented in *nfu3-2*	**AT4G34450**	0.000304	cytosol	8.88698e-05	0.00103682
under represented in *nfu3-2*	**AT4G36250**	5.7e-05	cytosol	1.3284e-05	0.000244767
under represented in *nfu3-2*	**AT4G37870**	9.55e-05	cytosol	3.08876e-05	0.000295184
under represented in *nfu3-2*	**AT4G38630**	0.000192	cytosol	8.0804e-05	0.000456854
under represented in *nfu3-2*	**AT4G39330**	0.000548	cytosol	0.000160224	0.00187275

under represented in *nfu3-2*	**AT5G02870**	0.000405	cytosol	0.000126511	0.00129853
under represented in *nfu3-2*	**AT5G03300**	0.000212	cytosol	0.000103317	0.000435985
under represented in *nfu3-2*	**AT5G05980**	1.33e-05	cytosol	5.20072e-06	3.41219e-05
under represented in *nfu3-2*	**AT5G10360**	0.00026	cytosol	7.33444e-05	0.000923154
under represented in *nfu3-2*	**AT5G16730**	2.29e-05	cytosol	9.04665e-06	5.77773e-05
under represented in *nfu3-2*	**AT5G19780**	0.000281	cytosol	0.000111181	0.000712557
under represented in *nfu3-2*	**AT5G20890**	0.000239	cytosol	8.36791e-05	0.000683142
under represented in *nfu3-2*	**AT5G22440**	4.85e-05	cytosol	1.44209e-05	0.000162884
under represented in *nfu3-2*	**AT5G23860**	2.97e-05	cytosol	5.72437e-06	0.000154602
under represented in *nfu3-2*	**AT5G26667**	0.000243	cytosol	0.000100132	0.000590572
under represented in *nfu3-2*	**AT5G27450**	1.46e-05	cytosol	6.46504e-06	3.31015e-05
under represented in *nfu3-2*	**AT5G27470**	0.000318	cytosol	0.000160186	0.000630864
under represented in *nfu3-2*	**AT5G36230**	7.44e-05	cytosol	2.93867e-05	0.000188349
under represented in *nfu3-2*	**AT5G44320**	2.47e-05	cytosol	7.02803e-06	8.65892e-05
under represented in *nfu3-2*	**AT5G47770**	0.000106	cytosol	3.35841e-05	0.000337401
under represented in *nfu3-2*	**AT5G49460**	7.32e-05	cytosol	2.02556e-05	0.000264726
under represented in *nfu3-2*	**AT5G56680**	0.000144	cytosol	5.65907e-05	0.000368619
under represented in *nfu3-2*	**AT5G62700**	0.000177	cytosol	6.50461e-05	0.000480436
under represented in *nfu3-2*	**AT1G09210**	0.000185	endoplasmic reticulum	5.58259e-05	0.000613462
under represented in *nfu3-2*	**AT1G67730**	0.000136	endoplasmic reticulum	4.19626e-05	0.000442004
under represented in *nfu3-2*	**AT2G03120**	6.64e-05	endoplasmic reticulum	2.6446e-05	0.000166543
under represented in *nfu3-2*	**AT4G15760**	5.47e-06	endoplasmic reticulum	1.66511e-06	1.79431e-05
under represented in *nfu3-2*	**AT4G36220**	1.75e-05	endoplasmic reticulum	5.6441e-06	5.45526e-05
under represented in *nfu3-2*	**AT5G56360**	5.78e-05	endoplasmic reticulum	1.39511e-05	0.000239544
under represented in *nfu3-2*	**AT5G58710**	0.00015	endoplasmic reticulum	4.8249e-05	0.000465577
under represented in *nfu3-2*	**AT5G60640**	0.00032	endoplasmic reticulum	0.000109933	0.000931301
under represented in *nfu3-2*	**AT5G63840**	7.38e-05	endoplasmic reticulum	1.78615e-05	0.000304538
under represented in *nfu3-2*	**AT1G68560**	0.000225	extracellular	7.4585e-05	0.00067969
under represented in *nfu3-2*	**AT1G78060**	7.16e-05	extracellular	2.63714e-05	0.000194285
under represented in *nfu3-2*	**AT2G05920**	0.000113	extracellular	3.12479e-05	0.000407911
under represented in *nfu3-2*	**AT2G06850**	0.00018	extracellular	7.07153e-05	0.000459232
under represented in *nfu3-2*	**AT2G28790**	0.000158	extracellular	3.91458e-05	0.000640375
under represented in *nfu3-2*	**AT2G38380**	0.000315	extracellular	5.62031e-05	0.00176428
under represented in *nfu3-2*	**AT3G08030**	0.000382	extracellular	0.000134905	0.00108092
under represented in *nfu3-2*	**AT3G14220**	2.04e-05	extracellular	8.20286e-06	5.06706e-05
under represented in *nfu3-2*	**AT3G20370**	9,00E-05	extracellular	1.66791e-05	0.000485731
under represented in *nfu3-2*	**AT3G54400**	0.000369	extracellular	0.000105036	0.00129795
under represented in *nfu3-2*	**AT4G37800**	0.000112	extracellular	3.07856e-05	0.000405233
under represented in *nfu3-2*	**AT5G07030**	0.000317	extracellular	9.89918e-05	0.00101713
under represented in *nfu3-2*	**AT5G11420**	0.000107	extracellular	3.58474e-05	0.000321674
under represented in *nfu3-2*	**AT5G12950**	2.71e-05	extracellular	8.80622e-06	8.36955e-05
under represented in *nfu3-2*	**AT5G20950**	0.000214	extracellular	6.93346e-05	0.000661811
under represented in *nfu3-2*	**AT5G25460**	7.58e-05	extracellular	2.55253e-05	0.000224962
under represented in *nfu3-2*	**AT5G43060**	9.22e-05	extracellular	2.86912e-05	0.000296376
under represented in *nfu3-2*	**AT5G45950**	2.26e-05	extracellular	7.95162e-06	6.42979e-05
under represented in *nfu3-2*	**AT1G05010**	0.000176	golgi	6.24311e-05	0.000493583
under represented in *nfu3-2*	**AT1G14670**	1.73e-05	golgi	2.80421e-06	0.000106132
under represented in *nfu3-2*	**AT1G26850**	9.76e-05	golgi	2.58948e-05	0.000367549
under represented in *nfu3-2*	**AT1G29470**	7.15e-05	golgi	1.84926e-05	0.000276268
under represented in *nfu3-2*	**AT1G45201**	0.000105	golgi	4.97153e-05	0.000222662
under represented in *nfu3-2*	**AT1G62380**	0.000456	golgi	0.00016984	0.00122449
under represented in *nfu3-2*	**AT2G04280**	1.38e-05	golgi	5.38431e-06	3.52719e-05
under represented in *nfu3-2*	**AT2G30520**	2.2e-05	golgi	7.05238e-06	6.87895e-05
under represented in *nfu3-2*	**AT2G40730**	4.69e-05	golgi	1.34836e-05	0.000163252
under represented in *nfu3-2*	**AT3G27530**	3,00E-05	golgi	9.37546e-06	9.58808e-05
under represented in *nfu3-2*	**AT3G44340**	3.86e-05	golgi	9.75306e-06	0.000153034
under represented in *nfu3-2*	**AT3G49720**	0.000139	golgi	4.89727e-05	0.000395553
under represented in *nfu3-2*	**AT4G12650**	8.43e-05	golgi	2.40108e-05	0.000295624
under represented in *nfu3-2*	**AT4G14360**	2.42e-05	golgi	4.85166e-06	0.000120608
under represented in *nfu3-2*	**AT4G19490**	1.19e-05	golgi	4.61596e-06	3.08965e-05
under represented in *nfu3-2*	**AT4G27640**	3.54e-05	golgi	1.20288e-05	0.000103993

under represented in *nfu3-2*	**AT5G51430**	3.89e-05	golgi	1.52592e-05	9.93613e-05
under represented in *nfu3-2*	**AT2G05710**	0.000421	mitochondrion	0.000130018	0.00136107
under represented in *nfu3-2*	**AT2G21870**	0.000727	mitochondrion	0.000285445	0.00185151
under represented in *nfu3-2*	**AT2G30970**	0.000331	mitochondrion	0.000134297	0.000814872
under represented in *nfu3-2*	**AT3G10370**	7.17e-05	mitochondrion	2.60281e-05	0.000197379
under represented in *nfu3-2*	**AT5G13490**	0.000155	mitochondrion	4.42827e-05	0.000541442
under represented in *nfu3-2*	**AT1G09270**	5.12e-05	nucleus	1.879e-05	0.000139706
under represented in *nfu3-2*	**AT1G62390**	3.72e-05	nucleus	1.29521e-05	0.000106595
under represented in *nfu3-2*	**AT2G21060**	0.000169	nucleus	5.49889e-05	0.000518173
under represented in *nfu3-2*	**AT5G22650**	0.000101	nucleus	2.25951e-05	0.000453291
under represented in *nfu3-2*	**AT5G52310**	9.27e-05	nucleus	1.97535e-05	0.000434884
under represented in *nfu3-2*	**AT5G56950**	8.01e-05	nucleus	3.22495e-05	0.000198745
under represented in *nfu3-2*	**AT5G61780**	0.000273	nucleus	8.15633e-05	0.000911623
under represented in *nfu3-2*	**AT1G31910**	3.87e-05	peroxisome	1.55124e-05	9.64689e-05
under represented in *nfu3-2*	**AT1G13110**	2.84e-05	plasma membrane	9.55086e-06	8.46877e-05
under represented in *nfu3-2*	**AT1G70940**	1.05e-05	plasma membrane	3.12559e-06	3.49577e-05
under represented in *nfu3-2*	**AT2G17980**	4.11e-05	plasma membrane	1.45874e-05	0.000115647
under represented in *nfu3-2*	**AT2G22125**	3.41e-05	plasma membrane	6.56848e-06	0.000177276
under represented in *nfu3-2*	**AT3G28860**	5.01e-05	plasma membrane	1.23483e-05	0.000202873
under represented in *nfu3-2*	**AT4G12730**	0.000174	plasma membrane	7.16893e-05	0.000420849
under represented in *nfu3-2*	**AT4G30190**	7.89e-05	plasma membrane	2.09616e-05	0.000297219
under represented in *nfu3-2*	**AT5G13520**	7.29e-05	plasma membrane	1.76493e-05	0.000301376
under represented in *nfu3-2*	**AT5G16590**	3.47e-05	plasma membrane	1.34122e-05	9.00115e-05
under represented in *nfu3-2*	**AT5G64080**	0.000133	plasma membrane	2.55801e-05	0.000694942
under represented in *nfu3-2*	**AT1G01090**	0.000199	plastid	7.54413e-05	0.000526216
under represented in *nfu3-2*	**AT1G03630**	0.000181	plastid	3.28211e-05	0.00100342
under represented in *nfu3-2*	**AT1G08520**	0.000107	plastid	2.88087e-05	0.000395965
under represented in *nfu3-2*	**AT1G31230**	6.88e-05	plastid	2.62612e-05	0.000180346
under represented in *nfu3-2*	**AT1G31330**	0.000972	plastid	0.000211429	0.00446642
under represented in *nfu3-2*	**AT1G56050**	3.65e-05	plastid	9.72008e-06	0.000137349
under represented in *nfu3-2*	**AT1G58080**	0.000117	plastid	4.09049e-05	0.000332596
under represented in *nfu3-2*	**AT1G61520**	0.00175	plastid	0.000336575	0.00911519
under represented in *nfu3-2*	**AT1G62750**	0.000387	plastid	7.39491e-05	0.00202317
under represented in *nfu3-2*	**AT1G64680**	4.44e-05	plastid	7.45357e-06	0.00026486
under represented in *nfu3-2*	**AT1G69830**	8.05e-05	plastid	2.15371e-05	0.000301043
under represented in *nfu3-2*	**AT1G74030**	0.000121	plastid	3.5258e-05	0.00041553
under represented in *nfu3-2*	**AT1G79560**	2,00E-05	plastid	4.18816e-06	9.58395e-05
under represented in *nfu3-2*	**AT1G80480**	6.77e-05	plastid	2.09673e-05	0.00021842
under represented in *nfu3-2*	**AT2G15620**	0.000287	plastid	7.02506e-05	0.00117638
under represented in *nfu3-2*	**AT2G34640**	2.39e-05	plastid	6.06816e-06	9.44981e-05
under represented in *nfu3-2*	**AT2G34860**	4.75e-05	plastid	1.53851e-05	0.000146732
under represented in *nfu3-2*	**AT2G36250**	6.48e-05	plastid	2.39555e-05	0.000175426
under represented in *nfu3-2*	**AT2G38550**	0.000128	plastid	5.05824e-05	0.000325385
under represented in *nfu3-2*	**AT2G40300**	4.73e-05	plastid	1.57431e-05	0.000142324
under represented in *nfu3-2*	**AT2G43710**	0.00014	plastid	6.1071e-05	0.000319041
under represented in *nfu3-2*	**AT3G01120**	4.21e-05	plastid	1.57726e-05	0.000112405
under represented in *nfu3-2*	**AT3G04870**	4.05e-05	plastid	1.35069e-05	0.000121416
under represented in *nfu3-2*	**AT3G10050**	2.15e-05	plastid	7.95695e-06	5.80719e-05
under represented in *nfu3-2*	**AT3G13070**	7.34e-06	plastid	2.59728e-06	2.0747e-05
under represented in *nfu3-2*	**AT3G13470**	0.000139	plastid	3.67233e-05	0.000527283
under represented in *nfu3-2*	**AT3G14930**	0.000131	plastid	3.52185e-05	0.000484038
under represented in *nfu3-2*	**AT3G16140**	0.000238	plastid	7.34458e-05	0.000772718
under represented in *nfu3-2*	**AT3G24430**	3.11e-05	plastid	7.41083e-06	0.000130881
under represented in *nfu3-2*	**AT3G24590**	4.23e-05	plastid	1.41803e-05	0.00012646
under represented in *nfu3-2*	**AT3G26900**	2.84e-05	plastid	7.74401e-06	0.000103839
under represented in *nfu3-2*	**AT3G46780**	0.000417	plastid	5.94951e-05	0.0029234
under represented in *nfu3-2*	**AT3G48110**	8.06e-05	plastid	2.12018e-05	0.000306139
under represented in *nfu3-2*	**AT3G48560**	0.000102	plastid	3.42915e-05	0.000305021
under represented in *nfu3-2*	**AT3G48730**	0.000137	plastid	3.11855e-05	0.000599653
under represented in *nfu3-2*	**AT3G48870**	9.21e-05	plastid	2.74536e-05	0.000309022
under represented in *nfu3-2*	**AT3G56940**	0.000224	plastid	5.01361e-05	0.000997448

under represented in *nfu3-2*	**AT3G59780**	9.54e-05	plastid	2.53574e-05	0.000359149
under represented in *nfu3-2*	**AT4G02770**	0.000346	plastid	9.40281e-05	0.00127305
under represented in *nfu3-2*	**AT4G03280**	0.000802	plastid	0.000202563	0.0031763
under represented in *nfu3-2*	**AT4G05390**	3.6e-05	plastid	1.56017e-05	8.31264e-05
under represented in *nfu3-2*	**AT4G12800**	0.000445	plastid	9.99233e-05	0.00197785
under represented in *nfu3-2*	**AT4G14210**	4.49e-05	plastid	1.29534e-05	0.000155291
under represented in *nfu3-2*	**AT4G15110**	3.71e-05	plastid	1.20881e-05	0.000113561
under represented in *nfu3-2*	**AT4G18440**	7.44e-05	plastid	2.33345e-05	0.000237209
under represented in *nfu3-2*	**AT4G18480**	0.000192	plastid	6.39098e-05	0.000579553
under represented in *nfu3-2*	**AT4G25080**	0.000203	plastid	5.41333e-05	0.000757605
under represented in *nfu3-2*	**AT4G27440**	0.000251	plastid	5.60046e-05	0.00112054
under represented in *nfu3-2*	**AT4G28750**	0.00102	plastid	0.00022994	0.00449177
under represented in *nfu3-2*	**AT4G30720**	2.1e-05	plastid	5.72751e-06	7.69349e-05
under represented in *nfu3-2*	**AT4G30950**	1.72e-05	plastid	4.28814e-06	6.87302e-05
under represented in *nfu3-2*	**AT5G01600**	0.000142	plastid	3.75569e-05	0.000533166
under represented in *nfu3-2*	**AT5G19940**	0.000116	plastid	3.59295e-05	0.000377441
under represented in *nfu3-2*	**AT5G27560**	2.07e-05	plastid	5.0319e-06	8.47868e-05
under represented in *nfu3-2*	**AT5G28500**	8.37e-05	plastid	1.7838e-05	0.000392492
under represented in *nfu3-2*	**AT5G58250**	0.000248	plastid	6.23051e-05	0.000987391
under represented in *nfu3-2*	**AT5G64040**	0.000369	plastid	7.714e-05	0.00176354
under represented in *nfu3-2*	**AT5G64940**	2.08e-05	plastid	2.88546e-06	0.000149628
under represented in *nfu3-2*	**AT5G67030**	5.25e-05	plastid	1.10442e-05	0.00024937
under represented in *nfu3-2*	**ATCG00340**	0.000638	plastid	0.000111714	0.00364374
under represented in *nfu3-2*	**ATCG00350**	0.000479	plastid	7.81654e-05	0.00293495
under represented in *nfu3-2*	**ATCG01060**	0.000844	plastid	0.000141185	0.0050475
under represented in *nfu3-2*	**AT1G54010**	0.000102	vacuole	2.42556e-05	0.000425431
under represented in *nfu3-2*	**AT1G06000**	7.63e-05		2.22963e-05	0.000261276
under represented in *nfu3-2*	**AT1G07890**	0.00148		0.000247806	0.0088582
under represented in *nfu3-2*	**AT1G09780**	0.000373		0.00013273	0.00104772
under represented in *nfu3-2*	**AT1G11650**	0.000178		8.49224e-05	0.000374614
under represented in *nfu3-2*	**AT1G12000**	0.000107		2.7633e-05	0.000412946
under represented in *nfu3-2*	**AT1G12270**	0.000113		4.33432e-05	0.000294177
under represented in *nfu3-2*	**AT1G14830**	8.34e-05		2.82308e-05	0.000246133
under represented in *nfu3-2*	**AT1G16920**	8.37e-05		3.03503e-05	0.000230796
under represented in *nfu3-2*	**AT1G20010**	0.000129		3.76801e-05	0.000439169
under represented in *nfu3-2*	**AT1G20050**	7.47e-05		2.24898e-05	0.000248263
under represented in *nfu3-2*	**AT1G20330**	6.92e-05		2.28837e-05	0.00020942
under represented in *nfu3-2*	**AT1G21750**	0.000536		0.000185421	0.0015494
under represented in *nfu3-2*	**AT1G24020**	0.000405		7.97002e-05	0.00205397
under represented in *nfu3-2*	**AT1G27090**	0.000105		4.60301e-05	0.000241269
under represented in *nfu3-2*	**AT1G29350**	1.19e-05		4.88985e-06	2.8839e-05
under represented in *nfu3-2*	**AT1G29660**	0.000167		2.957e-05	0.000943515
under represented in *nfu3-2*	**AT1G35720**	0.000616		0.000218881	0.00173627
under represented in *nfu3-2*	**AT1G48410**	4.46e-05		1.26154e-05	0.000157708
under represented in *nfu3-2*	**AT1G48600**	9.79e-05		3.52013e-05	0.000272145
under represented in *nfu3-2*	**AT1G49760**	0.000132		5.84921e-05	0.000300062
under represented in *nfu3-2*	**AT1G50670**	5.53e-05		2.46934e-05	0.000123791
under represented in *nfu3-2*	**AT1G55020**	7.12e-05		1.21611e-05	0.000416779
under represented in *nfu3-2*	**AT1G56340**	0.000311		0.000105814	0.000916629
under represented in *nfu3-2*	**AT1G65010**	2.89e-05		7.37656e-06	0.000113249
under represented in *nfu3-2*	**AT1G67430**	0.00014		4.07695e-05	0.000478686
under represented in *nfu3-2*	**AT1G70770**	0.00014		4.55796e-05	0.00042716
under represented in *nfu3-2*	**AT1G71220**	8.51e-05		2.22871e-05	0.000324681
under represented in *nfu3-2*	**AT1G76860**	8.81e-05		5.28257e-05	0.000146904
under represented in *nfu3-2*	**AT1G77510**	0.000236		8.53709e-05	0.000654639
under represented in *nfu3-2*	**AT1G80410**	8.03e-05		2.77381e-05	0.000232257
under represented in *nfu3-2*	**AT2G01720**	0.000109		4.78479e-05	0.000247967
under represented in *nfu3-2*	**AT2G07050**	3.94e-05		1.56865e-05	9.91046e-05
under represented in *nfu3-2*	**AT2G10940**	0.000562		0.000120491	0.00262098
under represented in *nfu3-2*	**AT2G20190**	2.2e-05		7.98029e-06	6.04391e-05
under represented in *nfu3-2*	**AT2G20610**	8,00E-05		2.81492e-05	0.000227447

under represented in *nfu3-2*	**AT2G20760**	0.000182	5.08735e-05	0.000654168
under represented in *nfu3-2*	**AT2G21160**	0.000404	0.000142391	0.00114581
under represented in *nfu3-2*	**AT2G23350**	0.000145	6.85305e-05	0.00030494
under represented in *nfu3-2*	**AT2G26250**	5.21e-05	1.37334e-05	0.000197862
under represented in *nfu3-2*	**AT2G30050**	0.000113	3.62427e-05	0.000354151
under represented in *nfu3-2*	**AT2G32920**	0.000111	4.34276e-05	0.00028511
under represented in *nfu3-2*	**AT2G35630**	1.46e-05	4.58679e-06	4.6573e-05
under represented in *nfu3-2*	**AT2G35880**	5.84e-05	2.48411e-05	0.000137258
under represented in *nfu3-2*	**AT2G38750**	9.88e-05	3.75081e-05	0.000259988
under represented in *nfu3-2*	**AT2G39310**	8.71e-05	9.64825e-06	0.000786432
under represented in *nfu3-2*	**AT2G45540**	9.61e-06	3.07591e-06	3.00097e-05
under represented in *nfu3-2*	**AT3G04120**	0.00081	0.000251672	0.00260677
under represented in *nfu3-2*	**AT3G04400**	0.000572	0.000232676	0.00140479
under represented in *nfu3-2*	**AT3G07100**	4.51e-05	1.37041e-05	0.000148667
under represented in *nfu3-2*	**AT3G07170**	5.28e-05	2.64019e-05	0.000105748
under represented in *nfu3-2*	**AT3G09260**	0.000669	0.000120823	0.00370727
under represented in *nfu3-2*	**AT3G11400**	0.000151	5.28656e-05	0.0004296
under represented in *nfu3-2*	**AT3G14650**	9.21e-06	4.35856e-06	1.94575e-05
under represented in *nfu3-2*	**AT3G15950**	0.00012	1.4174e-05	0.00102214
under represented in *nfu3-2*	**AT3G16400**	4.31e-05	9.04862e-06	0.000205214
under represented in *nfu3-2*	**AT3G16420**	0.000417	4.40818e-05	0.00394259
under represented in *nfu3-2*	**AT3G16460**	0.000296	5.24836e-05	0.00166948
under represented in *nfu3-2*	**AT3G17390**	0.000274	6.81468e-05	0.00110087
under represented in *nfu3-2*	**AT3G19820**	0.000195	5.30041e-05	0.000718155
under represented in *nfu3-2*	**AT3G20810**	1.23e-05	6.8227e-06	2.20046e-05
under represented in *nfu3-2*	**AT3G24503**	9.35e-05	2.98464e-05	0.000292772
under represented in *nfu3-2*	**AT3G29360**	0.00011	3.68426e-05	0.000331026
under represented in *nfu3-2*	**AT3G43300**	8.05e-05	2.29555e-05	0.000282451
under represented in *nfu3-2*	**AT3G44330**	0.00012	4.63283e-05	0.000312056
under represented in *nfu3-2*	**AT3G52140**	5.85e-05	1.52402e-05	0.000224887
under represented in *nfu3-2*	**AT3G52930**	0.000999	0.000364501	0.00273907
under represented in *nfu3-2*	**AT3G53710**	2.18e-05	9.2951e-06	5.13169e-05
under represented in *nfu3-2*	**AT3G54470**	0.000108	4.29574e-05	0.000272487
under represented in *nfu3-2*	**AT3G55360**	9.44e-05	3.37686e-05	0.000263917
under represented in *nfu3-2*	**AT3G62360**	7.22e-05	1.91009e-05	0.000273007
under represented in *nfu3-2*	**AT4G00400**	1.21e-05	2.44863e-06	6.02256e-05
under represented in *nfu3-2*	**AT4G01850**	0.000127	3.92589e-05	0.000413849
under represented in *nfu3-2*	**AT4G04340**	6.32e-06	1.48006e-06	2.69744e-05
under represented in *nfu3-2*	**AT4G11420**	0.000241	7.35232e-05	0.0007908
under represented in *nfu3-2*	**AT4G14160**	3.82e-05	1.61511e-05	9.02633e-05
under represented in *nfu3-2*	**AT4G16660**	0.000134	4.24302e-05	0.000423504
under represented in *nfu3-2*	**AT4G17520**	0.00018	6.50472e-05	0.000497638
under represented in *nfu3-2*	**AT4G17870**	5.66e-05	2.67972e-05	0.000119474
under represented in *nfu3-2*	**AT4G19210**	8.53e-05	4.01443e-05	0.000181052
under represented in *nfu3-2*	**AT4G21150**	0.000208	4.47856e-05	0.000969169
under represented in *nfu3-2*	**AT4G22690**	4.57e-05	1.14291e-05	0.000182536
under represented in *nfu3-2*	**AT4G23680**	0.000455	0.000110257	0.00187624
under represented in *nfu3-2*	**AT4G24190**	0.000591	0.00018244	0.00191381
under represented in *nfu3-2*	**AT4G24510**	3.84e-05	1.06604e-05	0.000138429
under represented in *nfu3-2*	**AT4G27500**	0.000242	9.86642e-05	0.000592485
under represented in *nfu3-2*	**AT4G29040**	1.07e-05	1.53901e-06	7.39605e-05
under represented in *nfu3-2*	**AT4G31480**	3.25e-05	1.1088e-05	9.49981e-05
under represented in *nfu3-2*	**AT4G31830**	9.5e-05	1.54891e-05	0.000582832
under represented in *nfu3-2*	**AT4G33030**	4.54e-05	1.37078e-05	0.000150543
under represented in *nfu3-2*	**AT4G34110**	0.000132	6.13804e-05	0.000283651
under represented in *nfu3-2*	**AT4G34200**	0.000581	0.000202382	0.00166532
under represented in *nfu3-2*	**AT4G34740**	2.44e-05	9.04559e-06	6.58892e-05
under represented in *nfu3-2*	**AT4G34830**	1.22e-05	3.08213e-06	4.84706e-05
under represented in *nfu3-2*	**AT4G37040**	2.09e-05	5.00667e-06	8.7364e-05
under represented in *nfu3-2*	**AT4G39980**	5.41e-05	1.87575e-05	0.000156191
under represented in *nfu3-2*	**AT5G02490**	9.03e-05	1.38835e-05	0.000587214

under represented in *nfu3-2*	AT5G02500	0.00104		0.000423707	0.00255684
under represented in *nfu3-2*	AT5G05010	0.000258		8.69394e-05	0.0007629
under represented in *nfu3-2*	AT5G07350	0.000214		5.60886e-05	0.000818041
under represented in *nfu3-2*	AT5G08280	0.000442		0.000108632	0.00179484
under represented in *nfu3-2*	AT5G11450	5.56e-05		1.38504e-05	0.000223098
under represented in *nfu3-2*	AT5G11880	2.93e-05		9.56514e-06	8.95699e-05
under represented in *nfu3-2*	AT5G16390	0.000365		0.000165286	0.000806255
under represented in *nfu3-2*	AT5G16620	0.000155		4.54805e-05	0.000527124
under represented in *nfu3-2*	AT5G17920	0.00185		0.000484519	0.00707412
under represented in *nfu3-2*	AT5G18660	6.13e-05		1.81853e-05	0.000206603
under represented in *nfu3-2*	AT5G19690	6.7e-05		2.18751e-05	0.000205027
under represented in *nfu3-2*	AT5G19990	2.36e-05		4.37568e-06	0.000127539
under represented in *nfu3-2*	AT5G20290	0.00158		0.000573611	0.00435312
under represented in *nfu3-2*	AT5G23040	3.46e-05		1.15821e-05	0.000103225
under represented in *nfu3-2*	AT5G24650	0.000134		5.22478e-05	0.000345715
under represented in *nfu3-2*	AT5G25100	3.17e-05		1.0168e-05	9.86564e-05
under represented in *nfu3-2*	AT5G25757	6.93e-05		2.12023e-05	0.000226729
under represented in *nfu3-2*	AT5G27640	2.85e-05		1.14656e-05	7.09584e-05
under represented in *nfu3-2*	AT5G36890	2.63e-05		7.94186e-06	8.72581e-05
under represented in *nfu3-2*	AT5G38470	0.000138		4.94731e-05	0.00038768
under represented in *nfu3-2*	AT5G41670	0.000133		4.63597e-05	0.000380486
under represented in *nfu3-2*	AT5G42020	0.000159		5.52506e-05	0.000457416
under represented in *nfu3-2*	AT5G42950	1.02e-05		3.93041e-06	2.63934e-05
under represented in *nfu3-2*	AT5G44340	0.000164		4.08443e-05	0.000660056
under represented in *nfu3-2*	AT5G46580	1.21e-05		2.30241e-06	6.36694e-05
under represented in *nfu3-2*	AT5G47010	2.83e-05		9.26339e-06	8.66906e-05
under represented in *nfu3-2*	AT5G47210	0.000266		8.56323e-05	0.000823471
under represented in *nfu3-2*	AT5G52970	0.000108		3.46468e-05	0.000337185
under represented in *nfu3-2*	AT5G53460	8.39e-05		2.42622e-05	0.000289971
under represented in *nfu3-2*	AT5G53560	0.000441		0.00018447	0.00105194
under represented in *nfu3-2*	AT5G54770	0.00042		0.00010083	0.00175207
under represented in *nfu3-2*	AT5G54900	0.000106		4.53824e-05	0.000249244
under represented in *nfu3-2*	AT5G56010	3.71e-05		6.45716e-06	0.000213552
under represented in *nfu3-2*	AT5G56630	3.83e-05		1.46286e-05	0.000100491
under represented in *nfu3-2*	AT5G58290	0.000259		0.000107404	0.000624874
under represented in *nfu3-2*	AT5G58590	9.86e-05		2.76418e-05	0.00035174
under represented in *nfu3-2*	AT5G60980	8.74e-05		3.22367e-05	0.000236777
under represented in *nfu3-2*	AT5G61790	0.000413		0.000142248	0.00119901
under represented in *nfu3-2*	AT5G65020	0.000229		7.09687e-05	0.000737013
under represented in *nfu3-2*	AT5G65110	1.39e-05		4.68531e-06	4.15219e-05
under represented in *nfu3-2*	ATCG00190	2.35e-05		4.80519e-06	0.000115084
under represented in *nfu3-2*	ATCG00490	0.0148		0.00213504	0.102513
under represented in *nfu3-2*	ATCG00540	0.000851		0.000144658	0.00500628
under represented in *nfu3-2*	ATCG00720	0.000653		0.00018007	0.00236693
over represented in *nfu3-2*	AT1G02920	0.000116	cytosol	3.5724e-05	0.000376219
over represented in *nfu3-2*	AT1G02930	0.000216	cytosol	6.5045e-05	0.00072008
over represented in *nfu3-2*	AT1G06410	4.62e-05	cytosol	1.86568e-05	0.000114208
over represented in *nfu3-2*	AT1G07110	1.95e-05	cytosol	5.96215e-06	6.37715e-05
over represented in *nfu3-2*	AT1G08980	8.05e-05	cytosol	3.19388e-05	0.000203012
over represented in *nfu3-2*	AT1G12050	4.28e-05	cytosol	1.53598e-05	0.000118999
over represented in *nfu3-2*	AT1G12780	6.48e-05	cytosol	2.29824e-05	0.000182603
over represented in *nfu3-2*	AT1G23440	4.55e-05	cytosol	1.54169e-05	0.000134108
over represented in *nfu3-2*	AT1G27020	4.22e-05	cytosol	1.02774e-05	0.000172957
over represented in *nfu3-2*	AT1G35670	1.56e-05	cytosol	4.40621e-06	5.49443e-05
over represented in *nfu3-2*	AT1G42550	4.77e-05	cytosol	1.12893e-05	0.000201291
over represented in *nfu3-2*	AT1G43670	0.000607	cytosol	0.000215005	0.00171538
over represented in *nfu3-2*	AT1G54100	0.00017	cytosol	4.07789e-05	0.000709717
over represented in *nfu3-2*	AT1G65930	0.00141	cytosol	0.000549296	0.00360368
over represented in *nfu3-2*	AT1G66200	0.000279	cytosol	8.17943e-05	0.000949885
over represented in *nfu3-2*	AT1G69410	8.82e-05	cytosol	2.95003e-05	0.000263405
over represented in *nfu3-2*	AT1G75280	0.000169	cytosol	6.55346e-05	0.00043726

over represented in *nfu3-2*	**AT1G80460**	7.69e-05	cytosol	1.45041e-05	0.000407402
over represented in *nfu3-2*	**AT2G24200**	0.000419	cytosol	0.000160846	0.00109096
over represented in *nfu3-2*	**AT2G29340**	0.000141	cytosol	5.76701e-05	0.000344543
over represented in *nfu3-2*	**AT2G29360**	4.96e-05	cytosol	1.66083e-05	0.000147958
over represented in *nfu3-2*	**AT2G37760**	0.000124	cytosol	4.62841e-05	0.000333242
over represented in *nfu3-2*	**AT3G18060**	5.8e-05	cytosol	1.9733e-05	0.000170185
over represented in *nfu3-2*	**AT3G23570**	0.000139	cytosol	5.34473e-05	0.000362156
over represented in *nfu3-2*	**AT3G26450**	0.000282	cytosol	0.000131407	0.000603437
over represented in *nfu3-2*	**AT3G47340**	3.03e-05	cytosol	5.69494e-06	0.000161013
over represented in *nfu3-2*	**AT3G53180**	4.63e-05	cytosol	1.52881e-05	0.00014011
over represented in *nfu3-2*	**AT3G55610**	2.93e-05	cytosol	1.13685e-05	7.54343e-05
over represented in *nfu3-2*	**AT4G02520**	0.00086	cytosol	0.000235666	0.00313657
over represented in *nfu3-2*	**AT4G10120**	1.66e-05	cytosol	2.76756e-06	9.96034e-05
over represented in *nfu3-2*	**AT4G24220**	7.67e-05	cytosol	3.31541e-05	0.000177318
over represented in *nfu3-2*	**AT4G24810**	1,00E-05	cytosol	3.45172e-06	2.90931e-05
over represented in *nfu3-2*	**AT4G33090**	0.000242	cytosol	6.77936e-05	0.00086722
over represented in *nfu3-2*	**AT4G33150**	1.62e-05	cytosol	3.32155e-06	7.86498e-05
over represented in *nfu3-2*	**AT4G33640**	0.000169	cytosol	7.32036e-05	0.000391547
over represented in *nfu3-2*	**AT4G36760**	9.68e-05	cytosol	3.48465e-05	0.000269121
over represented in *nfu3-2*	**AT5G13370**	2.08e-05	cytosol	8.7116e-06	4.95629e-05
over represented in *nfu3-2*	**AT5G16970**	0.000145	cytosol	5.70442e-05	0.000366309
over represented in *nfu3-2*	**AT5G28050**	0.000118	cytosol	4.95874e-05	0.000280634
over represented in *nfu3-2*	**AT5G36160**	4.92e-05	cytosol	1.84961e-05	0.000130819
over represented in *nfu3-2*	**AT5G39050**	1.63e-05	cytosol	6.03492e-06	4.42325e-05
over represented in *nfu3-2*	**AT5G41790**	3.82e-05	cytosol	8.0085e-06	0.000182364
over represented in *nfu3-2*	**AT5G44020**	0.000222	cytosol	6.61118e-05	0.000744895
over represented in *nfu3-2*	**AT5G48180**	6.62e-05	cytosol	2.88688e-05	0.000151959
over represented in *nfu3-2*	**AT5G49970**	4.9e-05	cytosol	2.04519e-05	0.000117316
over represented in *nfu3-2*	**AT5G51970**	0.000148	cytosol	6.18722e-05	0.000352754
over represented in *nfu3-2*	**AT5G54960**	6.97e-05	cytosol	2.33197e-05	0.000208096
over represented in *nfu3-2*	**AT4G31500**	4.7e-05	endoplasmic reticulum	1.90272e-05	0.000116291
over represented in *nfu3-2*	**AT1G02816**	0.000196	extracellular	5.8232e-05	0.000659324
over represented in *nfu3-2*	**AT1G03220**	0.000203	extracellular	5.67172e-05	0.000728776
over represented in *nfu3-2*	**AT1G03230**	0.000125	extracellular	4.00326e-05	0.000390976
over represented in *nfu3-2*	**AT1G13900**	4.02e-05	extracellular	1.67033e-05	9.67178e-05
over represented in *nfu3-2*	**AT1G17100**	0.000393	extracellular	0.000133028	0.00116322
over represented in *nfu3-2*	**AT1G21670**	5.35e-05	extracellular	1.78827e-05	0.000160159
over represented in *nfu3-2*	**AT1G21680**	8.19e-05	extracellular	3.69097e-05	0.000181869
over represented in *nfu3-2*	**AT1G33590**	0.000325	extracellular	0.000117332	0.000899544
over represented in *nfu3-2*	**AT1G33600**	5.35e-05	extracellular	2.00691e-05	0.000142872
over represented in *nfu3-2*	**AT1G47128**	0.000371	extracellular	9.43656e-05	0.00145885
over represented in *nfu3-2*	**AT1G75750**	0.000125	extracellular	3.63568e-05	0.000431153
over represented in *nfu3-2*	**AT1G76160**	0.000141	extracellular	3.9086e-05	0.000510794
over represented in *nfu3-2*	**AT1G78830**	0.000262	extracellular	7.41418e-05	0.000924172
over represented in *nfu3-2*	**AT1G78850**	0.000125	extracellular	3.79163e-05	0.000410394
over represented in *nfu3-2*	**AT2G04690**	8.95e-05	extracellular	3.91872e-05	0.000204527
over represented in *nfu3-2*	**AT2G05520**	0.000295	extracellular	8.03611e-05	0.00108154
over represented in *nfu3-2*	**AT3G07470**	0.000192	extracellular	8.48765e-05	0.000432539
over represented in *nfu3-2*	**AT3G10740**	0.000181	extracellular	5.82949e-05	0.000561161
over represented in *nfu3-2*	**AT3G13750**	8,00E-05	extracellular	2.46795e-05	0.000259329
over represented in *nfu3-2*	**AT3G13790**	2.99e-05	extracellular	7.6157e-06	0.000117574
over represented in *nfu3-2*	**AT3G16530**	9.3e-05	extracellular	3.17926e-05	0.000272049
over represented in *nfu3-2*	**AT3G22060**	4.59e-05	extracellular	1.6015e-05	0.000131516
over represented in *nfu3-2*	**AT3G47800**	0.000172	extracellular	6.55496e-05	0.000449336
over represented in *nfu3-2*	**AT3G51600**	0.000559	extracellular	0.000159457	0.00195998
over represented in *nfu3-2*	**AT3G52500**	7.24e-05	extracellular	2.66252e-05	0.000196624
over represented in *nfu3-2*	**AT3G55260**	5.68e-05	extracellular	2.08209e-05	0.000155012
over represented in *nfu3-2*	**AT3G56310**	5.68e-05	extracellular	2.01566e-05	0.000160081
over represented in *nfu3-2*	**AT4G00165**	7.08e-05	extracellular	2.06368e-05	0.000242981
over represented in *nfu3-2*	**AT4G13340**	3.4e-05	extracellular	1.24154e-05	9.30017e-05
over represented in *nfu3-2*	**AT4G19410**	0.00011	extracellular	4.35029e-05	0.000279131

over represented in *nfu3-2*	**AT4G20830**	3.7e-05	extracellular	1.17914e-05	0.000116108
over represented in *nfu3-2*	**AT4G25900**	8.21e-05	extracellular	2.47764e-05	0.000272102
over represented in *nfu3-2*	**AT4G30270**	0.000103	extracellular	4.22547e-05	0.000250007
over represented in *nfu3-2*	**AT4G34180**	0.000107	extracellular	4.42158e-05	0.000258372
over represented in *nfu3-2*	**AT5G13980**	0.000102	extracellular	3.72246e-05	0.000278582
over represented in *nfu3-2*	**AT5G19860**	0.000116	extracellular	3.98094e-05	0.000339683
over represented in *nfu3-2*	**AT5G34850**	0.000104	extracellular	4.07488e-05	0.000266743
over represented in *nfu3-2*	**AT5G42240**	5.31e-05	extracellular	1.19199e-05	0.000236184
over represented in *nfu3-2*	**AT5G49360**	0.000111	extracellular	2.04617e-05	0.000597879
over represented in *nfu3-2*	**AT5G56870**	2.18e-05	extracellular	6.23549e-06	7.61571e-05
over represented in *nfu3-2*	**AT5G64260**	0.000127	extracellular	5.84352e-05	0.000274485
over represented in *nfu3-2*	**AT5G64570**	7.5e-05	extracellular	1.67485e-05	0.000335859
over represented in *nfu3-2*	**AT5G39590**	2.03e-05	golgi	7.42658e-06	5.53463e-05
over represented in *nfu3-2*	**AT1G03090**	3.84e-05	mitochondrion	1.49943e-05	9.82808e-05
over represented in *nfu3-2*	**AT1G06570**	3.05e-05	mitochondrion	1.20738e-05	7.68603e-05
over represented in *nfu3-2*	**AT1G21400**	4.73e-06	mitochondrion	4.05091e-07	5.51282e-05
over represented in *nfu3-2*	**AT1G32470**	0.000611	mitochondrion	0.000155553	0.00240095
over represented in *nfu3-2*	**AT1G48030**	0.000511	mitochondrion	0.000215102	0.00121556
over represented in *nfu3-2*	**AT1G54220**	2.66e-05	mitochondrion	1.33757e-05	5.29157e-05
over represented in *nfu3-2*	**AT1G79440**	0.000171	mitochondrion	6.7341e-05	0.000435044
over represented in *nfu3-2*	**AT2G14170**	9.47e-05	mitochondrion	2.9005e-05	0.00030916
over represented in *nfu3-2*	**AT2G26080**	0.000235	mitochondrion	6.88532e-05	0.00080125
over represented in *nfu3-2*	**AT2G35370**	0.000711	mitochondrion	0.000199669	0.00252946
over represented in *nfu3-2*	**AT2G43400**	1.11e-05	mitochondrion	4.64528e-06	2.64216e-05
over represented in *nfu3-2*	**AT2G44350**	0.000283	mitochondrion	0.000100565	0.000793617
over represented in *nfu3-2*	**AT3G06050**	0.000408	mitochondrion	0.000184553	0.000900397
over represented in *nfu3-2*	**AT3G13450**	1.62e-05	mitochondrion	3.56624e-06	7.33464e-05
over represented in *nfu3-2*	**AT3G17240**	0.00021	mitochondrion	7.71875e-05	0.000568656
over represented in *nfu3-2*	**AT3G45300**	7.33e-05	mitochondrion	2.80939e-05	0.000191102
over represented in *nfu3-2*	**AT3G48000**	0.00026	mitochondrion	9.40401e-05	0.000717353
over represented in *nfu3-2*	**AT4G08870**	0.00018	mitochondrion	3.46694e-05	0.000938913
over represented in *nfu3-2*	**AT4G11600**	0.000315	mitochondrion	0.000138615	0.000714725
over represented in *nfu3-2*	**AT4G26910**	4.59e-05	mitochondrion	1.56305e-05	0.000134941
over represented in *nfu3-2*	**AT4G26970**	0.000271	mitochondrion	0.000102154	0.00072066
over represented in *nfu3-2*	**AT4G33010**	0.000535	mitochondrion	9.87645e-05	0.00289642
over represented in *nfu3-2*	**AT4G34030**	3.22e-05	mitochondrion	1.26639e-05	8.18676e-05
over represented in *nfu3-2*	**AT4G37930**	0.000545	mitochondrion	9.61671e-05	0.00308373
over represented in *nfu3-2*	**AT5G07440**	0.000132	mitochondrion	3.21114e-05	0.000544108
over represented in *nfu3-2*	**AT5G14780**	0.000357	mitochondrion	0.000119435	0.00106933
over represented in *nfu3-2*	**AT5G18170**	8.37e-05	mitochondrion	2.42276e-05	0.000289191
over represented in *nfu3-2*	**AT5G25940**	0.000147	mitochondrion	6.95185e-05	0.000312224
over represented in *nfu3-2*	**AT5G32470**	9.1e-06	mitochondrion	2.23805e-06	3.69903e-05
over represented in *nfu3-2*	**AT5G58270**	2.96e-05	mitochondrion	1.35023e-05	6.50973e-05
over represented in *nfu3-2*	**AT5G62530**	0.000166	mitochondrion	7.26457e-05	0.000378133
over represented in *nfu3-2*	**AT5G63620**	3.66e-05	mitochondrion	1.57539e-05	8.51943e-05
over represented in *nfu3-2*	**AT1G27970**	0.000187	nucleus	5.91933e-05	0.000591456
over represented in *nfu3-2*	**AT1G01820**	3.41e-05	peroxisome	1.38465e-05	8.39378e-05
over represented in *nfu3-2*	**AT1G20630**	9.77e-05	peroxisome	2.76372e-05	0.00034517
over represented in *nfu3-2*	**AT1G23310**	0.00027	peroxisome	4.391e-05	0.00166141
over represented in *nfu3-2*	**AT1G70580**	3.79e-05	peroxisome	9.23965e-06	0.000155342
over represented in *nfu3-2*	**AT1G76180**	0.000897	peroxisome	0.000229301	0.00350817
over represented in *nfu3-2*	**AT2G13360**	0.000652	peroxisome	0.000119794	0.00354413
over represented in *nfu3-2*	**AT2G42490**	2.14e-05	peroxisome	8.54578e-06	5.35704e-05
over represented in *nfu3-2*	**AT2G45740**	5.19e-05	peroxisome	1.5441e-05	0.000174775
over represented in *nfu3-2*	**AT3G01910**	0.000119	peroxisome	5.85987e-05	0.000243138
over represented in *nfu3-2*	**AT3G06810**	3.97e-05	peroxisome	1.51529e-05	0.000104263
over represented in *nfu3-2*	**AT3G12800**	9.03e-05	peroxisome	3.26979e-05	0.000249397
over represented in *nfu3-2*	**AT3G14415**	0.000284	peroxisome	4.56353e-05	0.00177154
over represented in *nfu3-2*	**AT3G14420**	0.000355	peroxisome	4.96352e-05	0.00253275
over represented in *nfu3-2*	**AT3G16910**	8.93e-05	peroxisome	3.9689e-05	0.000201048
over represented in *nfu3-2*	**AT3G24170**	0.000209	peroxisome	9.11464e-05	0.000478848

over represented in *nfu3-2*	AT3G47430	2.42e-05	peroxisome	5.41781e-06	0.000108249
over represented in *nfu3-2*	AT3G48170	7,00E-05	peroxisome	2.70498e-05	0.000181242
over represented in *nfu3-2*	AT3G51840	9.62e-05	peroxisome	3.87065e-05	0.000239004
over represented in *nfu3-2*	AT3G56460	0.000135	peroxisome	5.93156e-05	0.000308297
over represented in *nfu3-2*	AT4G04320	1.5e-05	peroxisome	6.84344e-06	3.27668e-05
over represented in *nfu3-2*	AT4G05160	6.71e-05	peroxisome	2.23288e-05	0.000201804
over represented in *nfu3-2*	AT4G29010	0.00018	peroxisome	6.92167e-05	0.000467844
over represented in *nfu3-2*	AT4G35000	0.000252	peroxisome	8.45165e-05	0.000751895
over represented in *nfu3-2*	AT5G11520	0.000107	peroxisome	3.20691e-05	0.000354721
over represented in *nfu3-2*	AT5G16370	1.15e-05	peroxisome	5.29369e-06	2.48152e-05
over represented in *nfu3-2*	AT5G41210	0.000103	peroxisome	4.96912e-05	0.000214493
over represented in *nfu3-2*	AT1G11260	0.000106	plasma membrane	5.14099e-05	0.000216567
over represented in *nfu3-2*	AT1G30360	0.00017	plasma membrane	7.03305e-05	0.000409777
over represented in *nfu3-2*	AT1G59870	8.86e-05	plasma membrane	2.12067e-05	0.000369965
over represented in *nfu3-2*	AT1G74790	2.64e-05	plasma membrane	1.21128e-05	5.75304e-05
over represented in *nfu3-2*	AT2G30930	0.000437	plasma membrane	0.000150091	0.00127518
over represented in *nfu3-2*	AT2G39480	6.97e-06	plasma membrane	2.55851e-06	1.89629e-05
over represented in *nfu3-2*	AT3G01290	0.000144	plasma membrane	3.28249e-05	0.000632119
over represented in *nfu3-2*	AT3G02880	8.46e-05	plasma membrane	2.66432e-05	0.000268793
over represented in *nfu3-2*	AT3G08510	8.27e-05	plasma membrane	2.6466e-05	0.000258312
over represented in *nfu3-2*	AT3G19930	3.34e-05	plasma membrane	7.17881e-06	0.000155117
over represented in *nfu3-2*	AT3G51550	5.35e-05	plasma membrane	2.39897e-05	0.000119125
over represented in *nfu3-2*	AT4G08850	2.79e-05	plasma membrane	1.05624e-05	7.37677e-05
over represented in *nfu3-2*	AT4G12420	0.000253	plasma membrane	0.000114145	0.000561319
over represented in *nfu3-2*	AT4G27520	0.000519	plasma membrane	0.000186078	0.00144929
over represented in *nfu3-2*	AT4G29900	3.81e-05	plasma membrane	1.47841e-05	9.80205e-05
over represented in *nfu3-2*	AT5G20230	0.000131	plasma membrane	2.16644e-05	0.000787019
over represented in *nfu3-2*	AT5G38990	5.04e-06	plasma membrane	1.59524e-06	1.59124e-05
over represented in *nfu3-2*	AT5G58090	4.2e-05	plasma membrane	1.65688e-05	0.000106435
over represented in *nfu3-2*	AT1G03600	0.000642	plastid	0.000154464	0.00266488
over represented in *nfu3-2*	AT1G06430	6.67e-05	plastid	2.01105e-05	0.000221447
over represented in *nfu3-2*	AT1G06690	0.000113	plastid	2.89555e-05	0.000444515
over represented in *nfu3-2*	AT1G07040	9.01e-05	plastid	3.65736e-05	0.000221872
over represented in *nfu3-2*	AT1G08550	7.5e-05	plastid	2.2347e-05	0.00025172
over represented in *nfu3-2*	AT1G09830	4.53e-05	plastid	1.75077e-05	0.000117004
over represented in *nfu3-2*	AT1G12250	0.000137	plastid	3.77895e-05	0.000498097
over represented in *nfu3-2*	AT1G12900	0.000588	plastid	0.000155458	0.00222516
over represented in *nfu3-2*	AT1G16720	7.45e-05	plastid	1.78107e-05	0.000311292
over represented in *nfu3-2*	AT1G16880	0.000533	plastid	0.000116213	0.00244555
over represented in *nfu3-2*	AT1G18170	6.43e-05	plastid	2.0661e-05	0.000200359
over represented in *nfu3-2*	AT1G20340	0.00409	plastid	0.000951788	0.0176172
over represented in *nfu3-2*	AT1G20810	5.77e-05	plastid	1.47445e-05	0.000226105
over represented in *nfu3-2*	AT1G23740	0.000514	plastid	0.000129418	0.00204214
over represented in *nfu3-2*	AT1G31160	0.000103	plastid	4.18357e-05	0.000254234
over represented in *nfu3-2*	AT1G31190	9.84e-05	plastid	2.4177e-05	0.000400345
over represented in *nfu3-2*	AT1G32080	0.000186	plastid	5.1149e-05	0.000678783
over represented in *nfu3-2*	AT1G32220	0.000148	plastid	4.0018e-05	0.000543663
over represented in *nfu3-2*	AT1G34000	0.000186	plastid	5.92189e-05	0.000586097
over represented in *nfu3-2*	AT1G44575	0.00118	plastid	0.000257309	0.00543559
over represented in *nfu3-2*	AT1G50250	5.83e-05	plastid	1.60529e-05	0.000212005
over represented in *nfu3-2*	AT1G54500	0.00016	plastid	6.27913e-05	0.000406132
over represented in *nfu3-2*	AT1G57770	2.65e-05	plastid	6.51022e-06	0.000108238
over represented in *nfu3-2*	AT1G68830	1.82e-05	plastid	2.94363e-06	0.000112735
over represented in *nfu3-2*	AT1G71480	1.59e-05	plastid	3.15941e-06	8.01645e-05
over represented in *nfu3-2*	AT1G71500	0.000287	plastid	6.84702e-05	0.00120179
over represented in *nfu3-2*	AT1G71810	1.13e-05	plastid	2.39816e-06	5.3636e-05
over represented in *nfu3-2*	AT1G73990	3.17e-05	plastid	8.22313e-06	0.000121888
over represented in *nfu3-2*	AT1G74970	0.00031	plastid	8.50721e-05	0.00112644
over represented in *nfu3-2*	AT1G78140	2.25e-05	plastid	8.75185e-06	5.76338e-05
over represented in *nfu3-2*	AT1G78620	6.32e-06	plastid	2.15979e-06	1.85048e-05
over represented in *nfu3-2*	AT1G80380	0.000189	plastid	4.1043e-05	0.000865745

over represented in *nfu3-2*	**AT2G17695**	3.18e-05	plastid	7.19043e-06	0.00014036
over represented in *nfu3-2*	**AT2G21330**	0.00069	plastid	0.000100725	0.00473055
over represented in *nfu3-2*	**AT2G21960**	6.45e-05	plastid	1.87924e-05	0.000221283
over represented in *nfu3-2*	**AT2G24820**	6.64e-05	plastid	1.76829e-05	0.000249472
over represented in *nfu3-2*	**AT2G25080**	0.000283	plastid	8.26495e-05	0.000969238
over represented in *nfu3-2*	**AT2G26930**	4.46e-05	plastid	1.13446e-05	0.000175031
over represented in *nfu3-2*	**AT2G28190**	0.0014	plastid	0.000363091	0.00538377
over represented in *nfu3-2*	**AT2G28605**	4.96e-05	plastid	1.62959e-05	0.000151166
over represented in *nfu3-2*	**AT2G34460**	0.000183	plastid	4.82728e-05	0.000697038
over represented in *nfu3-2*	**AT2G35410**	0.000207	plastid	5.19321e-05	0.000827397
over represented in *nfu3-2*	**AT2G35490**	0.00032	plastid	0.000107275	0.000955476
over represented in *nfu3-2*	**AT2G41040**	2.08e-05	plastid	8.40215e-06	5.16911e-05
over represented in *nfu3-2*	**AT2G41680**	8.77e-05	plastid	2.07474e-05	0.000370309
over represented in *nfu3-2*	**AT2G43180**	2.98e-05	plastid	1.05233e-05	8.46663e-05
over represented in *nfu3-2*	**AT2G43945**	6.19e-05	plastid	2.5704e-05	0.00014926
over represented in *nfu3-2*	**AT2G44050**	0.000109	plastid	3.42881e-05	0.000344584
over represented in *nfu3-2*	**AT2G44920**	0.000341	plastid	8.60221e-05	0.0013501
over represented in *nfu3-2*	**AT2G46910**	2.93e-05	plastid	8.12035e-06	0.000105891
over represented in *nfu3-2*	**AT2G47400**	0.000679	plastid	0.000123328	0.00373409
over represented in *nfu3-2*	**AT2G47730**	0.00113	plastid	0.000385874	0.00329255
over represented in *nfu3-2*	**AT2G47840**	6.61e-05	plastid	2.36218e-05	0.000185045
over represented in *nfu3-2*	**AT3G04790**	0.000923	plastid	0.000244356	0.00348938
over represented in *nfu3-2*	**AT3G08740**	0.000275	plastid	6.64339e-05	0.00114146
over represented in *nfu3-2*	**AT3G08920**	5.87e-05	plastid	2.20601e-05	0.000156091
over represented in *nfu3-2*	**AT3G10060**	0.00012	plastid	2.25083e-05	0.000643433
over represented in *nfu3-2*	**AT3G10130**	1.86e-05	plastid	6.01129e-06	5.74298e-05
over represented in *nfu3-2*	**AT3G12345**	0.000116	plastid	2.85287e-05	0.000467882
over represented in *nfu3-2*	**AT3G12780**	0.00187	plastid	0.000465744	0.00752958
over represented in *nfu3-2*	**AT3G18890**	0.000248	plastid	4.74901e-05	0.0012974
over represented in *nfu3-2*	**AT3G23070**	3.09e-06	plastid	5.27103e-07	1.80654e-05
over represented in *nfu3-2*	**AT3G23400**	0.000736	plastid	0.000273143	0.00198098
over represented in *nfu3-2*	**AT3G23700**	0.000173	plastid	5.36037e-05	0.000558743
over represented in *nfu3-2*	**AT3G25770**	0.000153	plastid	4.32521e-05	0.000539557
over represented in *nfu3-2*	**AT3G26060**	0.000791	plastid	0.000197778	0.0031622
over represented in *nfu3-2*	**AT3G26070**	0.000207	plastid	5.75811e-05	0.000742219
over represented in *nfu3-2*	**AT3G27850**	0.000525	plastid	0.000178792	0.00154266
over represented in *nfu3-2*	**AT3G43520**	8.93e-05	plastid	4.0048e-05	0.000199329
over represented in *nfu3-2*	**AT3G44880**	3.59e-05	plastid	1.29458e-05	9.97986e-05
over represented in *nfu3-2*	**AT3G44890**	0.000496	plastid	9.80873e-05	0.00250369
over represented in *nfu3-2*	**AT3G45140**	0.00147	plastid	0.000463426	0.00463468
over represented in *nfu3-2*	**AT3G48420**	0.000233	plastid	4.65852e-05	0.00116541
over represented in *nfu3-2*	**AT3G50820**	0.000696	plastid	0.000162979	0.00297418
over represented in *nfu3-2*	**AT3G52230**	0.000163	plastid	6.51182e-05	0.00040702
over represented in *nfu3-2*	**AT3G52960**	0.000924	plastid	0.000382679	0.00222908
over represented in *nfu3-2*	**AT3G54050**	0.000768	plastid	0.000183446	0.0032188
over represented in *nfu3-2*	**AT3G54660**	9.6e-05	plastid	3.02031e-05	0.000305453
over represented in *nfu3-2*	**AT3G55330**	0.000284	plastid	6.95537e-05	0.00115873
over represented in *nfu3-2*	**AT3G55400**	2.85e-05	plastid	1.09527e-05	7.40989e-05
over represented in *nfu3-2*	**AT3G56650**	0.000174	plastid	3.3497e-05	0.000907538
over represented in *nfu3-2*	**AT3G58010**	9.75e-05	plastid	2.56352e-05	0.000371051
over represented in *nfu3-2*	**AT3G60750**	0.00204	plastid	0.000382538	0.0108995
over represented in *nfu3-2*	**AT3G62030**	0.00138	plastid	0.000232646	0.00813493
over represented in *nfu3-2*	**AT3G62410**	0.000534	plastid	8.8481e-05	0.0032283
over represented in *nfu3-2*	**AT3G63140**	0.000556	plastid	0.000107881	0.00286989
over represented in *nfu3-2*	**AT3G63190**	0.000655	plastid	0.000227653	0.00188364
over represented in *nfu3-2*	**AT4G00490**	7.43e-05	plastid	2.66106e-05	0.000207357
over represented in *nfu3-2*	**AT4G02530**	0.000457	plastid	9.26167e-05	0.00225767
over represented in *nfu3-2*	**AT4G04020**	0.000338	plastid	0.000106436	0.00107611
over represented in *nfu3-2*	**AT4G04850**	6.45e-06	plastid	2.19228e-06	1.89586e-05
over represented in *nfu3-2*	**AT4G10300**	0.000195	plastid	5.15127e-05	0.000741561
over represented in *nfu3-2*	**AT4G10750**	4.8e-05	plastid	2.0185e-05	0.00011426

over represented in *nfu3-2*	**AT4G13500**	8.66e-05	plastid	3.42676e-05	0.000218639
over represented in *nfu3-2*	**AT4G14070**	2.95e-05	plastid	8.61623e-06	0.000100986
over represented in *nfu3-2*	**AT4G14870**	9.6e-05	plastid	3.02916e-05	0.000304307
over represented in *nfu3-2*	**AT4G17300**	3.48e-05	plastid	1.07215e-05	0.000113198
over represented in *nfu3-2*	**AT4G17560**	0.000238	plastid	6.75549e-05	0.000839737
over represented in *nfu3-2*	**AT4G18240**	9.04e-06	plastid	2.45462e-06	3.3295e-05
over represented in *nfu3-2*	**AT4G18810**	0.000107	plastid	3.54775e-05	0.000321971
over represented in *nfu3-2*	**AT4G19170**	5.52e-05	plastid	1.20533e-05	0.000252865
over represented in *nfu3-2*	**AT4G20360**	0.00172	plastid	0.000468178	0.00635393
over represented in *nfu3-2*	**AT4G20850**	0.000208	plastid	8.19106e-05	0.000526599
over represented in *nfu3-2*	**AT4G21280**	0.00159	plastid	0.000347266	0.00732037
over represented in *nfu3-2*	**AT4G21445**	0.000137	plastid	4.91917e-05	0.000381543
over represented in *nfu3-2*	**AT4G21860**	0.000135	plastid	4.93616e-05	0.000368933
over represented in *nfu3-2*	**AT4G22240**	0.00038	plastid	0.000152708	0.00094489
over represented in *nfu3-2*	**AT4G22890**	0.000294	plastid	6.73369e-05	0.00127932
over represented in *nfu3-2*	**AT4G23100**	0.000247	plastid	8.95644e-05	0.000678427
over represented in *nfu3-2*	**AT4G24620**	0.00029	plastid	0.000129957	0.000648112
over represented in *nfu3-2*	**AT4G25130**	0.000316	plastid	0.000110435	0.000903195
over represented in *nfu3-2*	**AT4G25370**	0.000196	plastid	5.2267e-05	0.000736088
over represented in *nfu3-2*	**AT4G25450**	5.16e-05	plastid	1.3189e-05	0.000202028
over represented in *nfu3-2*	**AT4G25650**	1.32e-05	plastid	6.3165e-06	2.76099e-05
over represented in *nfu3-2*	**AT4G26530**	0.000708	plastid	0.000146421	0.00342228
over represented in *nfu3-2*	**AT4G28730**	5.2e-05	plastid	1.62605e-05	0.000166214
over represented in *nfu3-2*	**AT4G30690**	2.4e-05	plastid	6.56746e-06	8.74791e-05
over represented in *nfu3-2*	**AT4G30910**	1.2e-05	plastid	3.51305e-06	4.06608e-05
over represented in *nfu3-2*	**AT4G30920**	6.84e-05	plastid	2.98342e-05	0.000157019
over represented in *nfu3-2*	**AT4G34240**	2.09e-05	plastid	6.60158e-06	6.64181e-05
over represented in *nfu3-2*	**AT5G02940**	3.18e-05	plastid	5.90365e-06	0.000171152
over represented in *nfu3-2*	**AT5G22510**	2.12e-05	plastid	8.20497e-06	5.48401e-05
over represented in *nfu3-2*	**AT5G23120**	0.00046	plastid	0.000101966	0.00207598
over represented in *nfu3-2*	**AT5G35970**	2.2e-05	plastid	3.68252e-06	0.000131199
over represented in *nfu3-2*	**AT5G42650**	0.000205	plastid	3.1001e-05	0.00136066
over represented in *nfu3-2*	**AT5G64290**	0.000113	plastid	3.88216e-05	0.000329257
over represented in *nfu3-2*	**AT1G12240**	0.000164	vacuole	6.16191e-05	0.000436407
over represented in *nfu3-2*	**AT1G30400**	2.83e-05	vacuole	7.00679e-06	0.000114193
over represented in *nfu3-2*	**AT1G75220**	2.43e-05	vacuole	1.18019e-05	5.02154e-05
over represented in *nfu3-2*	**AT2G21410**	6.25e-05	vacuole	2.36311e-05	0.000165526
over represented in *nfu3-2*	**AT2G41560**	4.94e-05	vacuole	1.36896e-05	0.000178546
over represented in *nfu3-2*	**AT3G62700**	2.59e-05	vacuole	8.9957e-06	7.45087e-05
over represented in *nfu3-2*	**AT3G63520**	6.91e-05	vacuole	2.25784e-05	0.000211294
over represented in *nfu3-2*	**AT4G02620**	0.00021	vacuole	6.73892e-05	0.00065357
over represented in *nfu3-2*	**AT4G39080**	0.000213	vacuole	7.29208e-05	0.000623127
over represented in *nfu3-2*	**AT5G14120**	4.97e-05	vacuole	1.89557e-05	0.000130279
over represented in *nfu3-2*	**AT5G60360**	0.000208	vacuole	8.3519e-05	0.00051917
over represented in *nfu3-2*	**AT1G02475**	3.13e-05		9.19059e-06	0.000106347
over represented in *nfu3-2*	**AT1G05560**	3.02e-05		8.77918e-06	0.000103622
over represented in *nfu3-2*	**AT1G09010**	6.38e-05		2.67373e-05	0.000152008
over represented in *nfu3-2*	**AT1G10360**	4,00E-05		1.37172e-05	0.000116588
over represented in *nfu3-2*	**AT1G10370**	0.000259		0.000102458	0.000657181
over represented in *nfu3-2*	**AT1G12840**	0.000552		0.000252908	0.00120676
over represented in *nfu3-2*	**AT1G17290**	0.000206		8.7703e-05	0.000482878
over represented in *nfu3-2*	**AT1G18270**	7.73e-05		2.16438e-05	0.000276138
over represented in *nfu3-2*	**AT1G20620**	0.00103		0.000263499	0.00400757
over represented in *nfu3-2*	**AT1G20816**	1.32e-05		3.86432e-06	4.51947e-05
over represented in *nfu3-2*	**AT1G49630**	1.72e-05		5.13169e-06	5.79775e-05
over represented in *nfu3-2*	**AT1G49670**	5.35e-05		1.75029e-05	0.000163673
over represented in *nfu3-2*	**AT1G49750**	0.000146		3.29962e-05	0.000644403
over represented in *nfu3-2*	**AT1G52400**	0.000349		5.90019e-05	0.0020655
over represented in *nfu3-2*	**AT1G55850**	1.77e-05		6.76792e-06	4.61187e-05
over represented in *nfu3-2*	**AT1G58270**	4.54e-05		1.43041e-05	0.000143788
over represented in *nfu3-2*	**AT1G63770**	0.000409		0.000103163	0.00161984

over represented in *nfu3-2*	**AT1G65590**	4.44e-05	1.64125e-05	0.000120016
over represented in *nfu3-2*	**AT1G68010**	0.00082	0.000132387	0.00508188
over represented in *nfu3-2*	**AT1G69840**	7.9e-05	2.656e-05	0.000235084
over represented in *nfu3-2*	**AT1G70290**	9.03e-06	4.33598e-06	1.87955e-05
over represented in *nfu3-2*	**AT1G72160**	9.66e-05	3.33783e-05	0.0002797
over represented in *nfu3-2*	**AT1G73650**	5.81e-05	1.98491e-05	0.00016992
over represented in *nfu3-2*	**AT1G76030**	5.79e-05	7.68841e-06	0.000435798
over represented in *nfu3-2*	**AT1G78900**	0.00138	0.000554525	0.00341824
over represented in *nfu3-2*	**AT2G05380**	8.63e-05	1.89413e-05	0.00039284
over represented in *nfu3-2*	**AT2G22990**	0.000107	3.09459e-05	0.000371529
over represented in *nfu3-2*	**AT2G24270**	0.000521	0.000166845	0.00162761
over represented in *nfu3-2*	**AT2G25450**	0.00019	5.16492e-05	0.000700077
over represented in *nfu3-2*	**AT2G26740**	5.05e-05	1.74502e-05	0.000145857
over represented in *nfu3-2*	**AT2G31170**	2.55e-05	1.05714e-05	6.1697e-05
over represented in *nfu3-2*	**AT2G32080**	0.000105	3.43534e-05	0.000323522
over represented in *nfu3-2*	**AT2G34310**	3.82e-05	1.70812e-05	8.53766e-05
over represented in *nfu3-2*	**AT2G35780**	5.22e-05	2.41824e-05	0.00011287
over represented in *nfu3-2*	**AT2G35840**	0.000106	4.61579e-05	0.000244146
over represented in *nfu3-2*	**AT2G37770**	4.83e-05	1.30247e-05	0.000179185
over represented in *nfu3-2*	**AT2G41100**	6.15e-05	1.75704e-05	0.000214981
over represented in *nfu3-2*	**AT2G43820**	4.14e-05	1.5303e-05	0.000112175
over represented in *nfu3-2*	**AT2G44310**	0.000237	5.81127e-05	0.000965359
over represented in *nfu3-2*	**AT3G01520**	6.86e-05	2.89801e-05	0.000162536
over represented in *nfu3-2*	**AT3G03980**	5.84e-05	1.8909e-05	0.000180522
over represented in *nfu3-2*	**AT3G06510**	4.19e-05	1.52916e-05	0.000114562
over represented in *nfu3-2*	**AT3G12010**	9.32e-06	3.6392e-06	2.38762e-05
over represented in *nfu3-2*	**AT3G12580**	0.00012	2.77031e-05	0.000517346
over represented in *nfu3-2*	**AT3G14067**	0.000268	0.0001039	0.000691718
over represented in *nfu3-2*	**AT3G19170**	0.000228	4.722e-05	0.00109644
over represented in *nfu3-2*	**AT3G21790**	1.83e-05	7.03641e-06	4.75328e-05
over represented in *nfu3-2*	**AT3G22200**	0.000269	8.28229e-05	0.000872613
over represented in *nfu3-2*	**AT3G23490**	0.000285	9.31419e-05	0.000870981
over represented in *nfu3-2*	**AT3G23920**	2.61e-05	9.34331e-06	7.311e-05
over represented in *nfu3-2*	**AT3G26720**	5.08e-05	1.07157e-05	0.000240933
over represented in *nfu3-2*	**AT3G27820**	2,00E-05	8.77836e-06	4.57428e-05
over represented in *nfu3-2*	**AT3G27890**	0.000221	8.30666e-05	0.000586418
over represented in *nfu3-2*	**AT3G28270**	9.11e-05	3.14902e-05	0.000263524
over represented in *nfu3-2*	**AT3G42050**	0.000212	9.30814e-05	0.000481194
over represented in *nfu3-2*	**AT3G44300**	9.66e-05	3.42767e-05	0.000272033
over represented in *nfu3-2*	**AT3G48990**	0.000337	0.00011761	0.000963891
over represented in *nfu3-2*	**AT3G54440**	4.03e-05	1.25986e-05	0.000129209
over represented in *nfu3-2*	**AT3G56290**	5.24e-05	1.2526e-05	0.000219143
over represented in *nfu3-2*	**AT3G58730**	0.000271	0.000104187	0.000706106
over represented in *nfu3-2*	**AT3G61220**	0.00018	5.72052e-05	0.000566677
over represented in *nfu3-2*	**AT4G01870**	4.25e-05	1.38189e-05	0.000130594
over represented in *nfu3-2*	**AT4G10060**	2.75e-05	6.31883e-06	0.00011951
over represented in *nfu3-2*	**AT4G11150**	0.000478	0.000125249	0.00182543
over represented in *nfu3-2*	**AT4G15530**	7.14e-05	9.69429e-06	0.000525906
over represented in *nfu3-2*	**AT4G19880**	4.77e-05	9.90229e-06	0.000229757
over represented in *nfu3-2*	**AT4G20260**	0.0015	0.000273642	0.00826344
over represented in *nfu3-2*	**AT4G20860**	3.79e-05	1.53366e-05	9.36837e-05
over represented in *nfu3-2*	**AT4G30310**	3.16e-05	1.42797e-05	6.98558e-05
over represented in *nfu3-2*	**AT4G32770**	3,00E-05	8.26698e-06	0.000109144
over represented in *nfu3-2*	**AT4G34120**	0.000146	4.68374e-05	0.000456146
over represented in *nfu3-2*	**AT4G34138**	2.7e-05	4.00296e-06	0.000182289
over represented in *nfu3-2*	**AT4G35090**	0.000606	0.000140731	0.00261263
over represented in *nfu3-2*	**AT4G35760**	3.12e-05	1.22514e-05	7.92423e-05
over represented in *nfu3-2*	**AT4G38810**	0.000122	4.87215e-05	0.000305283
over represented in *nfu3-2*	**AT4G39710**	9.46e-05	3.70628e-05	0.000241499
over represented in *nfu3-2*	**AT4G39730**	0.000539	0.000232085	0.00125316
over represented in *nfu3-2*	**AT5G04140**	0.000743	0.000118498	0.00465431

over represented in *nfu3-2*	**AT5G07020**	0.000287	8.66596e-05	0.000950564
over represented in *nfu3-2*	**AT5G08410**	0.000134	4.06499e-05	0.000440374
over represented in *nfu3-2*	**AT5G08740**	4.06e-05	1.12787e-05	0.000146436
over represented in *nfu3-2*	**AT5G09660**	0.00112	0.000175716	0.00720207
over represented in *nfu3-2*	**AT5G10470**	4.11e-05	9.60284e-06	0.000175715
over represented in *nfu3-2*	**AT5G11720**	3.62e-05	1.33458e-05	9.80553e-05
over represented in *nfu3-2*	**AT5G14740**	0.000683	6.91034e-05	0.00674908
over represented in *nfu3-2*	**AT5G16150**	9.08e-05	3.25941e-05	0.000252771
over represented in *nfu3-2*	**AT5G17170**	0.00021	5.61892e-05	0.000784151
over represented in *nfu3-2*	**AT5G17380**	7.04e-05	2.82439e-05	0.000175238
over represented in *nfu3-2*	**AT5G19220**	0.000157	3.05054e-05	0.000803483
over represented in *nfu3-2*	**AT5G20250**	3.41e-05	6.41102e-06	0.000181812
over represented in *nfu3-2*	**AT5G25980**	0.000894	0.000115242	0.00694018
over represented in *nfu3-2*	**AT5G27380**	6.39e-05	2.73015e-05	0.000149338
over represented in *nfu3-2*	**AT5G27390**	3.89e-05	9.28455e-06	0.000163129
over represented in *nfu3-2*	**AT5G35790**	3.23e-05	1.13213e-05	9.22061e-05
over represented in *nfu3-2*	**AT5G40370**	0.000666	0.000217848	0.00203574
over represented in *nfu3-2*	**AT5G40950**	0.000179	4.43625e-05	0.000719381
over represented in *nfu3-2*	**AT5G42270**	0.000174	4.15013e-05	0.00072798
over represented in *nfu3-2*	**AT5G42980**	0.00108	0.000331089	0.00353634
over represented in *nfu3-2*	**AT5G44130**	0.000133	3.74841e-05	0.000475365
over represented in *nfu3-2*	**AT5G46110**	0.000176	4.06504e-05	0.000762384
over represented in *nfu3-2*	**AT5G48300**	0.00029	0.000101817	0.000827971
over represented in *nfu3-2*	**AT5G49910**	0.000293	9.0129e-05	0.000951068
over represented in *nfu3-2*	**AT5G50640**	1.04e-05	2.67471e-06	4.03761e-05
over represented in *nfu3-2*	**AT5G50920**	0.000548	0.000145175	0.00206754
over represented in *nfu3-2*	**AT5G51070**	1.84e-05	3.45042e-06	9.82574e-05
over represented in *nfu3-2*	**AT5G51820**	0.00019	5.33616e-05	0.000673271
over represented in *nfu3-2*	**AT5G54500**	0.000312	0.000111394	0.000875542
over represented in *nfu3-2*	**AT5G57170**	6.93e-05	1.10253e-05	0.00043585
over represented in *nfu3-2*	**AT5G57655**	0.00023	6.20478e-05	0.000855421
over represented in *nfu3-2*	**AT5G58330**	0.000344	0.000120133	0.000987458
over represented in *nfu3-2*	**AT5G59250**	1.86e-05	6.33609e-06	5.4565e-05
over represented in *nfu3-2*	**AT5G59750**	1.04e-05	3.63459e-06	2.99623e-05
over represented in *nfu3-2*	**AT5G64250**	7.84e-05	3.56564e-05	0.000172439
over represented in *nfu3-2*	**AT5G65010**	7.9e-05	2.03492e-05	0.00030661
over represented in *nfu3-2*	**AT5G66570**	0.000934	0.000186144	0.00469067
over represented in *nfu3-2*	**AT5G67370**	8.19e-06	2.01284e-06	3.33632e-05
over represented in *nfu3-2*	**ATCG00120**	0.00291	0.000378807	0.0223987
over represented in *nfu3-2*	**ATCG00140**	0.00134	0.000348585	0.00513399
over represented in *nfu3-2*	**ATCG00280**	0.00152	0.000254511	0.00910645
over represented in *nfu3-2*	**ATCG00480**	0.0029	0.000450604	0.0186045
over represented in *nfu3-2*	**ATCG00750**	0.000226	6.2548e-05	0.000814872
over represented in *nfu3-2*	**ATCG00770**	0.000338	8.70392e-05	0.00131574
over represented in *nfu3-2*	**ATCG00810**	0.000111	2.56982e-05	0.000478251
over represented in *nfu3-2*	**ATCG00820**	0.00021	4.38899e-05	0.00100596
