# Supplementary material for: A Global Proteomic Approach Sheds New Light on Potential Iron-Sulfur Client Proteins of the Chloroplastic Maturation Factor NFU3
Source: Int J Mol Sci. 2020 Oct 30;21(21):8121. doi: 10.3390/ijms21218121 (PMC7672563; doi:10.3390/ijms21218121)
Supplement: Supplementary file 1 [file ijms-21-08121-s001.zip › ijms-973833 final suppl/Berger_et_al_IJMS_Table_S4_vIII.pdf]

**Table S4: detailed localization of proteins under accumulated specifically in *nfu3-2, or nfu2-1* or in both mutants. (NPAS = normalized protein abundance score)**

| Type of data | Protein ID | NPAS | HCM location | NPAS min | NPAS max |
|---|---|---|---|---|---|
| *nfu3-2* specific | AT1G02500 | 7.01e-05 | cytosol | 1.99413e-05 | 0.000246188 |
| *nfu3-2* specific | AT1G05510 | 7.3e-05 | cytosol | 3.6984e-06 | 0.00144232 |
| *nfu3-2* specific | AT1G08200 | 0.00008 | cytosol | 3.44786e-05 | 0.000185741 |
| *nfu3-2* specific | AT1G09310 | 0.000788 | cytosol | 0.000167365 | 0.00370935 |
| *nfu3-2* specific | AT1G10670 | 5.51e-05 | cytosol | 1.67738e-05 | 0.000181179 |
| *nfu3-2* specific | AT1G10840 | 0.000159 | cytosol | 6.20022e-05 | 0.000408937 |
| *nfu3-2* specific | AT1G11660 | 4.73e-05 | cytosol | 1.81026e-05 | 0.000123409 |
| *nfu3-2* specific | AT1G14320 | 0.000125 | cytosol | 3.30566e-05 | 0.000473918 |
| *nfu3-2* specific | AT1G18540 | 0.000341 | cytosol | 9.45541e-05 | 0.00123275 |
| *nfu3-2* specific | AT1G20950 | 6.97e-05 | cytosol | 2.34796e-05 | 0.000207171 |
| *nfu3-2* specific | AT1G22300 | 0.000821 | cytosol | 0.00032099 | 0.0021014 |
| *nfu3-2* specific | AT1G36730 | 2.07e-05 | cytosol | 8.99613e-06 | 4.76745e-05 |
| *nfu3-2* specific | AT1G43170 | 0.00052 | cytosol | 9.47542e-05 | 0.00285679 |
| *nfu3-2* specific | AT1G52100 | 9.62e-06 | cytosol | 2.24861e-06 | 4.1163e-05 |
| *nfu3-2* specific | AT1G53310 | 0.000142 | cytosol | 4.39562e-05 | 0.000457982 |
| *nfu3-2* specific | AT1G56070 | 0.00208 | cytosol | 0.000679831 | 0.00634887 |
| *nfu3-2* specific | AT1G77760 | 5.41e-06 | cytosol | 2.3782e-06 | 1.2329e-05 |
| *nfu3-2* specific | AT1G78570 | 5.91e-05 | cytosol | 2.00961e-05 | 0.000174052 |
| *nfu3-2* specific | AT1G79690 | 0.00015 | cytosol | 4.88191e-05 | 0.000459944 |
| *nfu3-2* specific | AT2G18020 | 0.000355 | cytosol | 0.000122024 | 0.00103553 |
| *nfu3-2* specific | AT2G27530 | 0.000275 | cytosol | 0.000108817 | 0.000695879 |
| *nfu3-2* specific | AT2G27860 | 8.57e-05 | cytosol | 3.53579e-05 | 0.000207628 |
| *nfu3-2* specific | AT2G29560 | 2.25e-05 | cytosol | 7.53912e-06 | 6.7423e-05 |
| *nfu3-2* specific | AT2G31390 | 0.000196 | cytosol | 4.74584e-05 | 0.000809757 |
| *nfu3-2* specific | AT2G34480 | 0.000653 | cytosol | 0.000226599 | 0.00187892 |
| *nfu3-2* specific | AT2G36880 | 0.000262 | cytosol | 6.26158e-05 | 0.00109937 |
| *nfu3-2* specific | AT3G01420 | 4.01e-05 | cytosol | 4.13648e-06 | 0.000389544 |
| *nfu3-2* specific | AT3G06650 | 7.86e-05 | cytosol | 2.59746e-05 | 0.000237988 |
| *nfu3-2* specific | AT3G09200 | 0.000837 | cytosol | 0.000364214 | 0.00192417 |
| *nfu3-2* specific | AT3G09820 | 0.000452 | cytosol | 0.000199345 | 0.00102468 |
| *nfu3-2* specific | AT3G11710 | 0.000184 | cytosol | 8.29933e-05 | 0.000407629 |
| *nfu3-2* specific | AT3G11830 | 0.000195 | cytosol | 6.86709e-05 | 0.000552478 |
| *nfu3-2* specific | AT3G12110 | 5.91e-05 | cytosol | 1.11228e-05 | 0.000313992 |
| *nfu3-2* specific | AT3G16450 | 0.000272 | cytosol | 4.42591e-05 | 0.00166823 |
| *nfu3-2* specific | AT3G17820 | 0.000117 | cytosol | 2.88882e-05 | 0.000472055 |
| *nfu3-2* specific | AT3G25230 | 0.000169 | cytosol | 6.95009e-05 | 0.00040982 |
| *nfu3-2* specific | AT3G51160 | 6.17e-05 | cytosol | 2.24529e-05 | 0.000169736 |
| *nfu3-2* specific | AT3G55620 | 6.31e-05 | cytosol | 2.5675e-05 | 0.000155276 |
| *nfu3-2* specific | AT3G57290 | 0.000216 | cytosol | 9.04409e-05 | 0.000517391 |
| *nfu3-2* specific | AT4G02450 | 0.00143 | cytosol | 0.000621026 | 0.00328192 |
| *nfu3-2* specific | AT4G10480 | 0.000398 | cytosol | 0.000153156 | 0.00103394 |
| *nfu3-2* specific | AT4G11820 | 4.96e-05 | cytosol | 1.29235e-05 | 0.000190175 |
| *nfu3-2* specific | AT4G13930 | 0.000714 | cytosol | 0.00025855 | 0.00197316 |
| *nfu3-2* specific | AT4G14960 | 4.14e-05 | cytosol | 5.59463e-06 | 0.000305972 |
| *nfu3-2* specific | AT4G15000 | 0.000348 | cytosol | 8.76595e-05 | 0.00138398 |
| *nfu3-2* specific | AT4G31120 | 4.29e-05 | cytosol | 1.56757e-05 | 0.000117183 |
| *nfu3-2* specific | AT4G31180 | 8.56e-05 | cytosol | 3.35626e-05 | 0.0002181 |
| *nfu3-2* specific | AT4G31790 | 2.97e-05 | cytosol | 1.01207e-05 | 8.72726e-05 |
| *nfu3-2* specific | AT4G34450 | 0.000304 | cytosol | 8.88698e-05 | 0.00103682 |
| *nfu3-2* specific | AT4G37870 | 9.55e-05 | cytosol | 3.08876e-05 | 0.000295184 |
| *nfu3-2* specific | AT4G38630 | 0.000192 | cytosol | 8.0804e-05 | 0.000456854 |
| *nfu3-2* specific | AT4G39330 | 0.000548 | cytosol | 0.000160224 | 0.00187275 |
| *nfu3-2* specific | AT5G02870 | 0.000405 | cytosol | 0.000126511 | 0.00129853 |
| *nfu3-2* specific | AT5G03300 | 0.000212 | cytosol | 0.000103317 | 0.000435985 |
| *nfu3-2* specific | AT5G05980 | 1.33e-05 | cytosol | 5.20072e-06 | 3.41219e-05 |
| *nfu3-2* specific | AT5G10360 | 0.00026 | cytosol | 7.33444e-05 | 0.000923154 |
| *nfu3-2* specific | AT5G16730 | 2.29e-05 | cytosol | 9.04665e-06 | 5.77773e-05 |

| | | | | | |
|---|---|---|---|---|---|
| *nfu3-2* specific | AT5G22440 | 4.85e-05 | cytosol | 1.44209e-05 | 0.000162884 |
| *nfu3-2* specific | AT5G23860 | 2.97e-05 | cytosol | 5.72437e-06 | 0.000154602 |
| *nfu3-2* specific | AT5G26667 | 0.000243 | cytosol | 0.000100132 | 0.000590572 |
| *nfu3-2* specific | AT5G27450 | 1.46e-05 | cytosol | 6.46504e-06 | 3.31015e-05 |
| *nfu3-2* specific | AT5G36230 | 7.44e-05 | cytosol | 2.93867e-05 | 0.000188349 |
| *nfu3-2* specific | AT5G44320 | 2.47e-05 | cytosol | 7.02803e-06 | 8.65892e-05 |
| *nfu3-2* specific | AT5G47770 | 0.000106 | cytosol | 3.35841e-05 | 0.000337401 |
| *nfu3-2* specific | AT5G49460 | 7.32e-05 | cytosol | 2.02556e-05 | 0.000264726 |
| *nfu3-2* specific | AT5G56680 | 0.000144 | cytosol | 5.65907e-05 | 0.000368619 |
| *nfu3-2* specific | AT1G09210 | 0.000185 | endoplasmic reticulum | 5.58259e-05 | 0.000613462 |
| *nfu3-2* specific | AT1G67730 | 0.000136 | endoplasmic reticulum | 4.19626e-05 | 0.000442004 |
| *nfu3-2* specific | AT2G03120 | 6.64e-05 | endoplasmic reticulum | 2.6446e-05 | 0.000166543 |
| *nfu3-2* specific | AT4G15760 | 5.47e-06 | endoplasmic reticulum | 1.66511e-06 | 1.79431e-05 |
| *nfu3-2* specific | AT4G36220 | 1.75e-05 | endoplasmic reticulum | 5.6441e-06 | 5.45526e-05 |
| *nfu3-2* specific | AT5G58710 | 0.00015 | endoplasmic reticulum | 4.8249e-05 | 0.000465577 |
| *nfu3-2* specific | AT5G63840 | 7.38e-05 | endoplasmic reticulum | 1.78615e-05 | 0.000304538 |
| *nfu3-2* specific | AT1G78060 | 7.16e-05 | extracellular | 2.63714e-05 | 0.000194285 |
| *nfu3-2* specific | AT2G05920 | 0.000113 | extracellular | 3.12479e-05 | 0.000407911 |
| *nfu3-2* specific | AT2G06850 | 0.00018 | extracellular | 7.07153e-05 | 0.000459232 |
| *nfu3-2* specific | AT2G28790 | 0.000158 | extracellular | 3.91458e-05 | 0.000640375 |
| *nfu3-2* specific | AT2G38380 | 0.000315 | extracellular | 5.62031e-05 | 0.00176428 |
| *nfu3-2* specific | AT3G08030 | 0.000382 | extracellular | 0.000134905 | 0.00108092 |
| *nfu3-2* specific | AT3G14220 | 2.04e-05 | extracellular | 8.20286e-06 | 5.06706e-05 |
| *nfu3-2* specific | AT3G20370 | 9,00E-05 | extracellular | 1.66791e-05 | 0.000485731 |
| *nfu3-2* specific | AT3G54400 | 0.000369 | extracellular | 0.000105036 | 0.00129795 |
| *nfu3-2* specific | AT4G37800 | 0.000112 | extracellular | 3.07856e-05 | 0.000405233 |
| *nfu3-2* specific | AT5G07030 | 0.000317 | extracellular | 9.89918e-05 | 0.00101713 |
| *nfu3-2* specific | AT5G11420 | 0.000107 | extracellular | 3.58474e-05 | 0.000321674 |
| *nfu3-2* specific | AT5G12950 | 2.71e-05 | extracellular | 8.80622e-06 | 8.36955e-05 |
| *nfu3-2* specific | AT5G20950 | 0.000214 | extracellular | 6.93346e-05 | 0.000661811 |
| *nfu3-2* specific | AT5G43060 | 9.22e-05 | extracellular | 2.86912e-05 | 0.000296376 |
| *nfu3-2* specific | AT5G45950 | 2.26e-05 | extracellular | 7.95162e-06 | 6.42979e-05 |
| *nfu3-2* specific | AT1G14670 | 1.73e-05 | golgi | 2.80421e-06 | 0.000106132 |
| *nfu3-2* specific | AT1G26850 | 9.76e-05 | golgi | 2.58948e-05 | 0.000367549 |
| *nfu3-2* specific | AT1G29470 | 7.15e-05 | golgi | 1.84926e-05 | 0.000276268 |
| *nfu3-2* specific | AT1G45201 | 0.000105 | golgi | 4.97153e-05 | 0.000222662 |
| *nfu3-2* specific | AT1G62380 | 0.000456 | golgi | 0.00016984 | 0.00122449 |
| *nfu3-2* specific | AT2G04280 | 1.38e-05 | golgi | 5.38431e-06 | 3.52719e-05 |
| *nfu3-2* specific | AT2G40730 | 4.69e-05 | golgi | 1.34836e-05 | 0.000163252 |
| *nfu3-2* specific | AT3G27530 | 3,00E-05 | golgi | 9.37546e-06 | 9.58808e-05 |
| *nfu3-2* specific | AT3G44340 | 3.86e-05 | golgi | 9.75306e-06 | 0.000153034 |
| *nfu3-2* specific | AT3G49720 | 0.000139 | golgi | 4.89727e-05 | 0.000395553 |
| *nfu3-2* specific | AT4G12650 | 8.43e-05 | golgi | 2.40108e-05 | 0.000295624 |
| *nfu3-2* specific | AT4G14360 | 2.42e-05 | golgi | 4.85166e-06 | 0.000120608 |
| *nfu3-2* specific | AT4G19490 | 1.19e-05 | golgi | 4.61596e-06 | 3.08965e-05 |
| *nfu3-2* specific | AT4G27640 | 3.54e-05 | golgi | 1.20288e-05 | 0.000103993 |
| *nfu3-2* specific | AT5G51430 | 3.89e-05 | golgi | 1.52592e-05 | 9.93613e-05 |
| *nfu3-2* specific | AT2G21870 | 0.000727 | mitochondrion | 0.000285445 | 0.00185151 |
| *nfu3-2* specific | AT2G30970 | 0.000331 | mitochondrion | 0.000134297 | 0.000814872 |
| *nfu3-2* specific | AT3G10370 | 7.17e-05 | mitochondrion | 2.60281e-05 | 0.000197379 |
| *nfu3-2* specific | AT5G13490 | 0.000155 | mitochondrion | 4.42827e-05 | 0.000541442 |
| *nfu3-2* specific | AT1G09270 | 5.12e-05 | nucleus | 1.879e-05 | 0.000139706 |
| *nfu3-2* specific | AT1G62390 | 3.72e-05 | nucleus | 1.29521e-05 | 0.000106595 |
| *nfu3-2* specific | AT2G21060 | 0.000169 | nucleus | 5.49889e-05 | 0.000518173 |
| *nfu3-2* specific | AT5G22650 | 0.000101 | nucleus | 2.25951e-05 | 0.000453291 |
| *nfu3-2* specific | AT5G56950 | 8.01e-05 | nucleus | 3.22495e-05 | 0.000198745 |
| *nfu3-2* specific | AT5G61780 | 0.000273 | nucleus | 8.15633e-05 | 0.000911623 |
| *nfu3-2* specific | AT1G31910 | 3.87e-05 | peroxisome | 1.55124e-05 | 9.64689e-05 |
| *nfu3-2* specific | AT1G13110 | 2.84e-05 | plasma membrane | 9.55086e-06 | 8.46877e-05 |
| *nfu3-2* specific | AT2G17980 | 4.11e-05 | plasma membrane | 1.45874e-05 | 0.000115647 |
| *nfu3-2* specific | AT2G22125 | 3.41e-05 | plasma membrane | 6.56848e-06 | 0.000177276 |

| | | | | | |
|---|---|---|---|---|---|
| *nfu3-2* specific | **AT3G28860** | 5.01e-05 | plasma membrane | 1.23483e-05 | 0.000202873 |
| *nfu3-2* specific | **AT4G12730** | 0.000174 | plasma membrane | 7.16893e-05 | 0.000420849 |
| *nfu3-2* specific | **AT4G30190** | 7.89e-05 | plasma membrane | 2.09616e-05 | 0.000297219 |
| *nfu3-2* specific | **AT5G13520** | 7.29e-05 | plasma membrane | 1.76493e-05 | 0.000301376 |
| *nfu3-2* specific | **AT5G64080** | 0.000133 | plasma membrane | 2.55801e-05 | 0.000694942 |
| *nfu3-2* specific | **AT1G01090** | 0.000199 | plastid | 7.54413e-05 | 0.000526216 |
| *nfu3-2* specific | **AT1G03630** | 0.000181 | plastid | 3.28211e-05 | 0.00100342 |
| *nfu3-2* specific | **AT1G08520** | 0.000107 | plastid | 2.88087e-05 | 0.000395965 |
| *nfu3-2* specific | **AT1G31230** | 6.88e-05 | plastid | 2.62612e-05 | 0.000180346 |
| *nfu3-2* specific | **AT1G56050** | 3.65e-05 | plastid | 9.72008e-06 | 0.000137349 |
| *nfu3-2* specific | **AT1G58080** | 0.000117 | plastid | 4.09049e-05 | 0.000332596 |
| *nfu3-2* specific | **AT1G62750** | 0.000387 | plastid | 7.39491e-05 | 0.00202317 |
| *nfu3-2* specific | **AT1G64680** | 4.44e-05 | plastid | 7.45357e-06 | 0.00026486 |
| *nfu3-2* specific | **AT1G74030** | 0.000121 | plastid | 3.5258e-05 | 0.00041553 |
| *nfu3-2* specific | **AT1G79560** | 2,00E-05 | plastid | 4.18816e-06 | 9.58395e-05 |
| *nfu3-2* specific | **AT1G80480** | 6.77e-05 | plastid | 2.09673e-05 | 0.00021842 |
| *nfu3-2* specific | **AT2G15620** | 0.000287 | plastid | 7.02506e-05 | 0.00117638 |
| *nfu3-2* specific | **AT2G34640** | 2.39e-05 | plastid | 6.06816e-06 | 9.44981e-05 |
| *nfu3-2* specific | **AT2G34860** | 4.75e-05 | plastid | 1.53851e-05 | 0.000146732 |
| *nfu3-2* specific | **AT2G38550** | 0.000128 | plastid | 5.05824e-05 | 0.000325385 |
| *nfu3-2* specific | **AT2G40300** | 4.73e-05 | plastid | 1.57431e-05 | 0.000142324 |
| *nfu3-2* specific | **AT2G43710** | 0.00014 | plastid | 6.1071e-05 | 0.000319041 |
| *nfu3-2* specific | **AT3G01120** | 4.21e-05 | plastid | 1.57726e-05 | 0.000112405 |
| *nfu3-2* specific | **AT3G10050** | 2.15e-05 | plastid | 7.95695e-06 | 5.80719e-05 |
| *nfu3-2* specific | **AT3G13070** | 7.34e-06 | plastid | 2.59728e-06 | 2.0747e-05 |
| *nfu3-2* specific | **AT3G13470** | 0.000139 | plastid | 3.67233e-05 | 0.000527283 |
| *nfu3-2* specific | **AT3G16140** | 0.000238 | plastid | 7.34458e-05 | 0.000772718 |
| *nfu3-2* specific | **AT3G24430** | 3.11e-05 | plastid | 7.41083e-06 | 0.000130881 |
| *nfu3-2* specific | **AT3G24590** | 4.23e-05 | plastid | 1.41803e-05 | 0.00012646 |
| *nfu3-2* specific | **AT3G26900** | 2.84e-05 | plastid | 7.74401e-06 | 0.000103839 |
| *nfu3-2* specific | **AT3G48110** | 8.06e-05 | plastid | 2.12018e-05 | 0.000306139 |
| *nfu3-2* specific | **AT3G48560** | 0.000102 | plastid | 3.42915e-05 | 0.000305021 |
| *nfu3-2* specific | **AT3G48730** | 0.000137 | plastid | 3.11855e-05 | 0.000599653 |
| *nfu3-2* specific | **AT3G48870** | 9.21e-05 | plastid | 2.74536e-05 | 0.000309022 |
| *nfu3-2* specific | **AT3G56940** | 0.000224 | plastid | 5.01361e-05 | 0.000997448 |
| *nfu3-2* specific | **AT4G02770** | 0.000346 | plastid | 9.40281e-05 | 0.00127305 |
| *nfu3-2* specific | **AT4G03280** | 0.000802 | plastid | 0.000202563 | 0.0031763 |
| *nfu3-2* specific | **AT4G05390** | 3.6e-05 | plastid | 1.56017e-05 | 8.31264e-05 |
| *nfu3-2* specific | **AT4G18440** | 7.44e-05 | plastid | 2.33345e-05 | 0.000237209 |
| *nfu3-2* specific | **AT4G18480** | 0.000192 | plastid | 6.39098e-05 | 0.000579553 |
| *nfu3-2* specific | **AT4G30720** | 2.1e-05 | plastid | 5.72751e-06 | 7.69349e-05 |
| *nfu3-2* specific | **AT4G30950** | 1.72e-05 | plastid | 4.28814e-06 | 6.87302e-05 |
| *nfu3-2* specific | **AT5G01600** | 0.000142 | plastid | 3.75569e-05 | 0.000533166 |
| *nfu3-2* specific | **AT5G19940** | 0.000116 | plastid | 3.59295e-05 | 0.000377441 |
| *nfu3-2* specific | **AT5G27560** | 2.07e-05 | plastid | 5.0319e-06 | 8.47868e-05 |
| *nfu3-2* specific | **AT5G28500** | 8.37e-05 | plastid | 1.7838e-05 | 0.000392492 |
| *nfu3-2* specific | **AT5G58250** | 0.000248 | plastid | 6.23051e-05 | 0.000987391 |
| *nfu3-2* specific | **AT1G54010** | 0.000102 | vacuole | 2.42556e-05 | 0.000425431 |
| *nfu3-2* specific | **AT1G06000** | 7.63e-05 | | 2.22963e-05 | 0.000261276 |
| *nfu3-2* specific | **AT1G09780** | 0.000373 | | 0.00013273 | 0.00104772 |
| *nfu3-2* specific | **AT1G11650** | 0.000178 | | 8.49224e-05 | 0.000374614 |
| *nfu3-2* specific | **AT1G12000** | 0.000107 | | 2.7633e-05 | 0.000412946 |
| *nfu3-2* specific | **AT1G12270** | 0.000113 | | 4.33432e-05 | 0.000294177 |
| *nfu3-2* specific | **AT1G14830** | 8.34e-05 | | 2.82308e-05 | 0.000246133 |
| *nfu3-2* specific | **AT1G16920** | 8.37e-05 | | 3.03503e-05 | 0.000230796 |
| *nfu3-2* specific | **AT1G20050** | 7.47e-05 | | 2.24898e-05 | 0.000248263 |
| *nfu3-2* specific | **AT1G21750** | 0.000536 | | 0.000185421 | 0.0015494 |
| *nfu3-2* specific | **AT1G24020** | 0.000405 | | 7.97002e-05 | 0.00205397 |
| *nfu3-2* specific | **AT1G27090** | 0.000105 | | 4.60301e-05 | 0.000241269 |
| *nfu3-2* specific | **AT1G29350** | 1.19e-05 | | 4.88985e-06 | 2.8839e-05 |
| *nfu3-2* specific | **AT1G48410** | 4.46e-05 | | 1.26154e-05 | 0.000157708 |

| | | | | |
|---|---|---|---|---|
| *nfu3-2* specific | **AT1G48600** | 9.79e-05 | 3.52013e-05 | 0.000272145 |
| *nfu3-2* specific | **AT1G49760** | 0.000132 | 5.84921e-05 | 0.000300062 |
| *nfu3-2* specific | **AT1G50670** | 5.53e-05 | 2.46934e-05 | 0.000123791 |
| *nfu3-2* specific | **AT1G55020** | 7.12e-05 | 1.21611e-05 | 0.000416779 |
| *nfu3-2* specific | **AT1G56340** | 0.000311 | 0.000105814 | 0.000916629 |
| *nfu3-2* specific | **AT1G65010** | 2.89e-05 | 7.37656e-06 | 0.000113249 |
| *nfu3-2* specific | **AT1G67430** | 0.00014 | 4.07695e-05 | 0.000478686 |
| *nfu3-2* specific | **AT1G70770** | 0.00014 | 4.55796e-05 | 0.00042716 |
| *nfu3-2* specific | **AT1G71220** | 8.51e-05 | 2.22871e-05 | 0.000324681 |
| *nfu3-2* specific | **AT1G76860** | 8.81e-05 | 5.28257e-05 | 0.000146904 |
| *nfu3-2* specific | **AT1G77510** | 0.000236 | 8.53709e-05 | 0.000654639 |
| *nfu3-2* specific | **AT1G80410** | 8.03e-05 | 2.77381e-05 | 0.000232257 |
| *nfu3-2* specific | **AT2G01720** | 0.000109 | 4.78479e-05 | 0.000247967 |
| *nfu3-2* specific | **AT2G10940** | 0.000562 | 0.000120491 | 0.00262098 |
| *nfu3-2* specific | **AT2G20610** | 8,00E-05 | 2.81492e-05 | 0.000227447 |
| *nfu3-2* specific | **AT2G20760** | 0.000182 | 5.08735e-05 | 0.000654168 |
| *nfu3-2* specific | **AT2G23350** | 0.000145 | 6.85305e-05 | 0.00030494 |
| *nfu3-2* specific | **AT2G26250** | 5.21e-05 | 1.37334e-05 | 0.000197862 |
| *nfu3-2* specific | **AT2G30050** | 0.000113 | 3.62427e-05 | 0.000354151 |
| *nfu3-2* specific | **AT2G32920** | 0.000111 | 4.34276e-05 | 0.00028511 |
| *nfu3-2* specific | **AT2G35630** | 1.46e-05 | 4.58679e-06 | 4.6573e-05 |
| *nfu3-2* specific | **AT2G35880** | 5.84e-05 | 2.48411e-05 | 0.000137258 |
| *nfu3-2* specific | **AT2G38750** | 9.88e-05 | 3.75081e-05 | 0.000259988 |
| *nfu3-2* specific | **AT2G39310** | 8.71e-05 | 9.64825e-06 | 0.000786432 |
| *nfu3-2* specific | **AT2G45540** | 9.61e-06 | 3.07591e-06 | 3.00097e-05 |
| *nfu3-2* specific | **AT3G04120** | 0.00081 | 0.000251672 | 0.00260677 |
| *nfu3-2* specific | **AT3G04400** | 0.000572 | 0.000232676 | 0.00140479 |
| *nfu3-2* specific | **AT3G07100** | 4.51e-05 | 1.37041e-05 | 0.000148667 |
| *nfu3-2* specific | **AT3G07170** | 5.28e-05 | 2.64019e-05 | 0.000105748 |
| *nfu3-2* specific | **AT3G09260** | 0.000669 | 0.000120823 | 0.00370727 |
| *nfu3-2* specific | **AT3G11400** | 0.000151 | 5.28656e-05 | 0.0004296 |
| *nfu3-2* specific | **AT3G14650** | 9.21e-06 | 4.35856e-06 | 1.94575e-05 |
| *nfu3-2* specific | **AT3G15950** | 0.00012 | 1.4174e-05 | 0.00102214 |
| *nfu3-2* specific | **AT3G16400** | 4.31e-05 | 9.04862e-06 | 0.000205214 |
| *nfu3-2* specific | **AT3G16420** | 0.000417 | 4.40818e-05 | 0.00394259 |
| *nfu3-2* specific | **AT3G16460** | 0.000296 | 5.24836e-05 | 0.00166948 |
| *nfu3-2* specific | **AT3G17390** | 0.000274 | 6.81468e-05 | 0.00110087 |
| *nfu3-2* specific | **AT3G19820** | 0.000195 | 5.30041e-05 | 0.000718155 |
| *nfu3-2* specific | **AT3G20810** | 1.23e-05 | 6.8227e-06 | 2.20046e-05 |
| *nfu3-2* specific | **AT3G24503** | 9.35e-05 | 2.98464e-05 | 0.000292772 |
| *nfu3-2* specific | **AT3G43300** | 8.05e-05 | 2.29555e-05 | 0.000282451 |
| *nfu3-2* specific | **AT3G44330** | 0.00012 | 4.63283e-05 | 0.000312056 |
| *nfu3-2* specific | **AT3G52140** | 5.85e-05 | 1.52402e-05 | 0.000224887 |
| *nfu3-2* specific | **AT3G52930** | 0.000999 | 0.000364501 | 0.00273907 |
| *nfu3-2* specific | **AT3G53710** | 2.18e-05 | 9.2951e-06 | 5.13169e-05 |
| *nfu3-2* specific | **AT3G55360** | 9.44e-05 | 3.37686e-05 | 0.000263917 |
| *nfu3-2* specific | **AT3G62360** | 7.22e-05 | 1.91009e-05 | 0.000273007 |
| *nfu3-2* specific | **AT4G00400** | 1.21e-05 | 2.44863e-06 | 6.02256e-05 |
| *nfu3-2* specific | **AT4G01850** | 0.000127 | 3.92589e-05 | 0.000413849 |
| *nfu3-2* specific | **AT4G04340** | 6.32e-06 | 1.48006e-06 | 2.69744e-05 |
| *nfu3-2* specific | **AT4G11420** | 0.000241 | 7.35232e-05 | 0.0007908 |
| *nfu3-2* specific | **AT4G14160** | 3.82e-05 | 1.61511e-05 | 9.02633e-05 |
| *nfu3-2* specific | **AT4G16660** | 0.000134 | 4.24302e-05 | 0.000423504 |
| *nfu3-2* specific | **AT4G17520** | 0.00018 | 6.50472e-05 | 0.000497638 |
| *nfu3-2* specific | **AT4G17870** | 5.66e-05 | 2.67972e-05 | 0.000119474 |
| *nfu3-2* specific | **AT4G19210** | 8.53e-05 | 4.01443e-05 | 0.000181052 |
| *nfu3-2* specific | **AT4G21150** | 0.000208 | 4.47856e-05 | 0.000969169 |
| *nfu3-2* specific | **AT4G22690** | 4.57e-05 | 1.14291e-05 | 0.000182536 |
| *nfu3-2* specific | **AT4G23680** | 0.000455 | 0.000110257 | 0.00187624 |
| *nfu3-2* specific | **AT4G24510** | 3.84e-05 | 1.06604e-05 | 0.000138429 |
| *nfu3-2* specific | **AT4G27500** | 0.000242 | 9.86642e-05 | 0.000592485 |

| | | | | | |
|---|---|---|---|---|---|
| *nfu3-2* specific | **AT4G31480** | 3.25e-05 | | 1.1088e-05 | 9.49981e-05 |
| *nfu3-2* specific | **AT4G31830** | 9.5e-05 | | 1.54891e-05 | 0.000582832 |
| *nfu3-2* specific | **AT4G33030** | 4.54e-05 | | 1.37078e-05 | 0.000150543 |
| *nfu3-2* specific | **AT4G34110** | 0.000132 | | 6.13804e-05 | 0.000283651 |
| *nfu3-2* specific | **AT4G34740** | 2.44e-05 | | 9.04559e-06 | 6.58892e-05 |
| *nfu3-2* specific | **AT4G34830** | 1.22e-05 | | 3.08213e-06 | 4.84706e-05 |
| *nfu3-2* specific | **AT4G37040** | 2.09e-05 | | 5.00667e-06 | 8.7364e-05 |
| *nfu3-2* specific | **AT4G39980** | 5.41e-05 | | 1.87575e-05 | 0.000156191 |
| *nfu3-2* specific | **AT5G02490** | 9.03e-05 | | 1.38835e-05 | 0.000587214 |
| *nfu3-2* specific | **AT5G02500** | 0.00104 | | 0.000423707 | 0.00255684 |
| *nfu3-2* specific | **AT5G05010** | 0.000258 | | 8.69394e-05 | 0.0007629 |
| *nfu3-2* specific | **AT5G07350** | 0.000214 | | 5.60886e-05 | 0.000818041 |
| *nfu3-2* specific | **AT5G08280** | 0.000442 | | 0.000108632 | 0.00179484 |
| *nfu3-2* specific | **AT5G11880** | 2.93e-05 | | 9.56514e-06 | 8.95699e-05 |
| *nfu3-2* specific | **AT5G16390** | 0.000365 | | 0.000165286 | 0.000806255 |
| *nfu3-2* specific | **AT5G16620** | 0.000155 | | 4.54805e-05 | 0.000527124 |
| *nfu3-2* specific | **AT5G17920** | 0.00185 | | 0.000484519 | 0.00707412 |
| *nfu3-2* specific | **AT5G18660** | 6.13e-05 | | 1.81853e-05 | 0.000206603 |
| *nfu3-2* specific | **AT5G19990** | 2.36e-05 | | 4.37568e-06 | 0.000127539 |
| *nfu3-2* specific | **AT5G20290** | 0.00158 | | 0.000573611 | 0.00435312 |
| *nfu3-2* specific | **AT5G23040** | 3.46e-05 | | 1.15821e-05 | 0.000103225 |
| *nfu3-2* specific | **AT5G24650** | 0.000134 | | 5.22478e-05 | 0.000345715 |
| *nfu3-2* specific | **AT5G25100** | 3.17e-05 | | 1.0168e-05 | 9.86564e-05 |
| *nfu3-2* specific | **AT5G25757** | 6.93e-05 | | 2.12023e-05 | 0.000226729 |
| *nfu3-2* specific | **AT5G27640** | 2.85e-05 | | 1.14656e-05 | 7.09584e-05 |
| *nfu3-2* specific | **AT5G36890** | 2.63e-05 | | 7.94186e-06 | 8.72581e-05 |
| *nfu3-2* specific | **AT5G38470** | 0.000138 | | 4.94731e-05 | 0.00038768 |
| *nfu3-2* specific | **AT5G41670** | 0.000133 | | 4.63597e-05 | 0.000380486 |
| *nfu3-2* specific | **AT5G42950** | 1.02e-05 | | 3.93041e-06 | 2.63934e-05 |
| *nfu3-2* specific | **AT5G46580** | 1.21e-05 | | 2.30241e-06 | 6.36694e-05 |
| *nfu3-2* specific | **AT5G47010** | 2.83e-05 | | 9.26339e-06 | 8.66906e-05 |
| *nfu3-2* specific | **AT5G47210** | 0.000266 | | 8.56323e-05 | 0.000823471 |
| *nfu3-2* specific | **AT5G53560** | 0.000441 | | 0.00018447 | 0.00105194 |
| *nfu3-2* specific | **AT5G54900** | 0.000106 | | 4.53824e-05 | 0.000249244 |
| *nfu3-2* specific | **AT5G56010** | 3.71e-05 | | 6.45716e-06 | 0.000213552 |
| *nfu3-2* specific | **AT5G56630** | 3.83e-05 | | 1.46286e-05 | 0.000100491 |
| *nfu3-2* specific | **AT5G58290** | 0.000259 | | 0.000107404 | 0.000624874 |
| *nfu3-2* specific | **AT5G58590** | 9.86e-05 | | 2.76418e-05 | 0.00035174 |
| *nfu3-2* specific | **AT5G60980** | 8.74e-05 | | 3.22367e-05 | 0.000236777 |
| *nfu3-2* specific | **AT5G61790** | 0.000413 | | 0.000142248 | 0.00119901 |
| *nfu3-2* specific | **AT5G65020** | 0.000229 | | 7.09687e-05 | 0.000737013 |
| *nfu3-2* specific | **AT5G65110** | 1.39e-05 | | 4.68531e-06 | 4.15219e-05 |
| *nfu3-2* specific | **ATCG00190** | 2.35e-05 | | 4.80519e-06 | 0.000115084 |
| *nfu3-2* specific | **ATCG00490** | 0.0148 | | 0.00213504 | 0.102513 |
| *nfu3-2* specific | **ATCG00540** | 0.000851 | | 0.000144658 | 0.00500628 |
| *nfu3-2* specific | **ATCG00720** | 0.000653 | | 0.00018007 | 0.00236693 |
| *nfu2-1* specific | **AT1G30690** | 0.000282 | cytosol | 0.000120569 | 0.000659798 |
| *nfu2-1* specific | **AT1G50010** | 5.99e-05 | cytosol | 1.58401e-05 | 0.00022646 |
| *nfu2-1* specific | **AT1G55260** | 3.56e-05 | cytosol | 1.56846e-05 | 8.06246e-05 |
| *nfu2-1* specific | **AT2G43790** | 6.53e-05 | cytosol | 2.34744e-05 | 0.000181535 |
| *nfu2-1* specific | **AT3G18860** | 5.95e-05 | cytosol | 2.38551e-05 | 0.000148488 |
| *nfu2-1* specific | **AT4G16830** | 7.96e-05 | cytosol | 1.6361e-05 | 0.000387674 |
| *nfu2-1* specific | **AT4G17100** | 3.1e-05 | cytosol | 1.19626e-05 | 8.03799e-05 |
| *nfu2-1* specific | **AT4G30160** | 4.17e-05 | cytosol | 1.19154e-05 | 0.000146151 |
| *nfu2-1* specific | **AT5G09810** | 0.00056 | cytosol | 0.000185525 | 0.00169005 |
| *nfu2-1* specific | **AT5G11670** | 0.000227 | cytosol | 6.09933e-05 | 0.000842454 |
| *nfu2-1* specific | **AT5G11950** | 6.29e-05 | cytosol | 2.32737e-05 | 0.000170019 |
| *nfu2-1* specific | **AT5G12250** | 0.000114 | cytosol | 3.18286e-05 | 0.000410713 |
| *nfu2-1* specific | **AT5G38830** | 4.6e-05 | cytosol | 2.20397e-05 | 9.60512e-05 |
| *nfu2-1* specific | **AT2G28470** | 0.000112 | extracellular | 3.26738e-05 | 0.000383355 |
| *nfu2-1* specific | **AT3G14310** | 0.000166 | extracellular | 5.30633e-05 | 0.000518907 |

| | | | | | |
|---|---|---|---|---|---|
| *nfu2-1* specific | **AT3G18080** | 0.000146 | extracellular | 4.77579e-05 | 0.000446881 |
| *nfu2-1* specific | **AT5G55730** | 0.000169 | extracellular | 4.06342e-05 | 0.000706292 |
| *nfu2-1* specific | **AT1G10290** | 7.6e-05 | golgi | 2.67006e-05 | 0.000216121 |
| *nfu2-1* specific | **AT3G23300** | 2.34e-05 | golgi | 4.1578e-06 | 0.000131744 |
| *nfu2-1* specific | **AT1G51980** | 0.00039 | mitochondrion | 0.000150962 | 0.00100603 |
| *nfu2-1* specific | **AT4G35260** | 0.000113 | mitochondrion | 4.43218e-05 | 0.000288218 |
| *nfu2-1* specific | **AT5G65750** | 5.15e-05 | mitochondrion | 1.53827e-05 | 0.00017247 |
| *nfu2-1* specific | **ATMG00510** | 6.61e-05 | mitochondrion | 2.40969e-05 | 0.000181236 |
| *nfu2-1* specific | **AT2G19520** | 9.21e-05 | nucleus | 3.19218e-05 | 0.000265982 |
| *nfu2-1* specific | **AT3G54540** | 6.24e-05 | nucleus | 2.71882e-05 | 0.000143025 |
| *nfu2-1* specific | **AT4G31880** | 5.53e-05 | nucleus | 1.28882e-05 | 0.000237683 |
| *nfu2-1* specific | **AT5G43960** | 5.26e-05 | nucleus | 2.36162e-05 | 0.000117232 |
| *nfu2-1* specific | **AT4G05530** | 0.000152 | peroxisome | 6.66405e-05 | 0.000347105 |
| *nfu2-1* specific | **AT1G04690** | 0.00038 | plasma membrane | 0.000176488 | 0.000820129 |
| *nfu2-1* specific | **AT1G06700** | 3.27e-05 | plasma membrane | 1.48498e-05 | 7.20065e-05 |
| *nfu2-1* specific | **AT1G59610** | 0.000106 | plasma membrane | 4.10744e-05 | 0.000275684 |
| *nfu2-1* specific | **AT3G07390** | 0.000324 | plasma membrane | 0.000101683 | 0.00103158 |
| *nfu2-1* specific | **AT3G10380** | 4.05e-05 | plasma membrane | 1.52953e-05 | 0.000107168 |
| *nfu2-1* specific | **AT3G14840** | 3.72e-05 | plasma membrane | 1.30125e-05 | 0.000106436 |
| *nfu2-1* specific | **AT3G26290** | 1.32e-05 | plasma membrane | 5.25588e-06 | 3.31805e-05 |
| *nfu2-1* specific | **AT3G45190** | 2.32e-05 | plasma membrane | 9.11838e-06 | 5.90768e-05 |
| *nfu2-1* specific | **AT4G35790** | 4.28e-05 | plasma membrane | 1.19222e-05 | 0.00015357 |
| *nfu2-1* specific | **AT5G58140** | 4.93e-05 | plasma membrane | 1.18663e-05 | 0.00020443 |
| *nfu2-1* specific | **AT1G03130** | 0.000127 | plastid | 2.799e-05 | 0.000575194 |
| *nfu2-1* specific | **AT1G08380** | 0.000381 | plastid | 0.000123968 | 0.00116902 |
| *nfu2-1* specific | **AT1G15980** | 0.000117 | plastid | 3.30342e-05 | 0.000411442 |
| *nfu2-1* specific | **AT1G28140** | 1.41e-05 | plastid | 3.30324e-06 | 5.98335e-05 |
| *nfu2-1* specific | **AT1G29900** | 0.000261 | plastid | 8.24677e-05 | 0.000825575 |
| *nfu2-1* specific | **AT1G30380** | 0.000121 | plastid | 3.06052e-05 | 0.000476807 |
| *nfu2-1* specific | **AT1G36280** | 2.03e-05 | plastid | 7.23823e-06 | 5.67216e-05 |
| *nfu2-1* specific | **AT1G52230** | 0.00027 | plastid | 4.62188e-05 | 0.00157422 |
| *nfu2-1* specific | **AT1G55670** | 0.000213 | plastid | 3.91431e-05 | 0.00116149 |
| *nfu2-1* specific | **AT1G66430** | 0.00014 | plastid | 3.58363e-05 | 0.00054781 |
| *nfu2-1* specific | **AT1G70820** | 8.95e-05 | plastid | 2.40446e-05 | 0.000333339 |
| *nfu2-1* specific | **AT1G74960** | 4.88e-05 | plastid | 1.70767e-05 | 0.00013971 |
| *nfu2-1* specific | **AT1G77590** | 7.51e-05 | plastid | 1.90294e-05 | 0.000296004 |
| *nfu2-1* specific | **AT2G04039** | 5.42e-05 | plastid | 1.38257e-05 | 0.000212768 |
| *nfu2-1* specific | **AT2G05070** | 0.000445 | plastid | 0.000130556 | 0.00151919 |
| *nfu2-1* specific | **AT2G20260** | 0.00145 | plastid | 0.000576925 | 0.00362333 |
| *nfu2-1* specific | **AT2G30170** | 4.29e-05 | plastid | 1.40628e-05 | 0.000130966 |
| *nfu2-1* specific | **AT3G27740** | 8.45e-05 | plastid | 3.69013e-05 | 0.000193508 |
| *nfu2-1* specific | **AT3G27925** | 0.000171 | plastid | 3.95179e-05 | 0.000740039 |
| *nfu2-1* specific | **AT3G44880** | 3.59e-05 | plastid | 1.29458e-05 | 9.97986e-05 |
| *nfu2-1* specific | **AT3G47070** | 0.00085 | plastid | 0.000132394 | 0.00546269 |
| *nfu2-1* specific | **AT3G47470** | 0.00118 | plastid | 0.000310658 | 0.00449619 |
| *nfu2-1* specific | **AT3G52180** | 5.95e-05 | plastid | 2.00499e-05 | 0.000176868 |
| *nfu2-1* specific | **AT3G54890** | 0.001 | plastid | 0.000199989 | 0.00501008 |
| *nfu2-1* specific | **AT3G61470** | 0.000759 | plastid | 0.000189799 | 0.00303622 |
| *nfu2-1* specific | **AT3G63540** | 0.000353 | plastid | 9.48329e-05 | 0.0013117 |
| *nfu2-1* specific | **AT4G19170** | 5.52e-05 | plastid | 1.20533e-05 | 0.000252865 |
| *nfu2-1* specific | **AT4G23890** | 0.000161 | plastid | 5.3925e-05 | 0.000483361 |
| *nfu2-1* specific | **AT4G31530** | 4.33e-05 | plastid | 1.14501e-05 | 0.000163486 |
| *nfu2-1* specific | **AT4G33500** | 3.59e-05 | plastid | 1.10517e-05 | 0.00011692 |
| *nfu2-1* specific | **AT5G10920** | 0.000133 | plastid | 6.16623e-05 | 0.000286617 |
| *nfu2-1* specific | **AT5G23890** | 0.000113 | plastid | 3.38235e-05 | 0.00037511 |
| *nfu2-1* specific | **AT5G35170** | 8.66e-05 | plastid | 1.71062e-05 | 0.000438871 |
| *nfu2-1* specific | **AT5G39830** | 6.08e-05 | plastid | 1.95432e-05 | 0.000189394 |
| *nfu2-1* specific | **AT5G42765** | 7.47e-05 | plastid | 1.67854e-05 | 0.000332147 |
| *nfu2-1* specific | **AT5G44650** | 8.55e-05 | plastid | 2.97084e-05 | 0.000246163 |
| *nfu2-1* specific | **AT3G14210** | 0.000651 | vacuole | 7.8886e-05 | 0.00536748 |
| *nfu2-1* specific | **AT5G46860** | 0.000101 | vacuole | 4.97648e-05 | 0.000204779 |

| | | | | | |
|---|---|---|---|---|---|
| *nfu2-1* specific | **AT1G13280** | 0.000252 | | 8.01179e-05 | 0.000793334 |
| *nfu2-1* specific | **AT1G19870** | 7.65e-05 | | 2.39437e-05 | 0.000244223 |
| *nfu2-1* specific | **AT1G20440** | 0.000427 | | 8.2628e-05 | 0.0022057 |
| *nfu2-1* specific | **AT1G20450** | 0.000338 | | 0.000106741 | 0.00106923 |
| *nfu2-1* specific | **AT1G20620** | 0.00103 | | 0.000263499 | 0.00400757 |
| *nfu2-1* specific | **AT1G22530** | 0.000257 | | 5.8518e-05 | 0.00113087 |
| *nfu2-1* specific | **AT1G31730** | 3.62e-05 | | 1.904e-05 | 6.86991e-05 |
| *nfu2-1* specific | **AT1G50500** | 2.86e-05 | | 1.11883e-05 | 7.28574e-05 |
| *nfu2-1* specific | **AT1G58270** | 4.54e-05 | | 1.43041e-05 | 0.000143788 |
| *nfu2-1* specific | **AT1G72150** | 0.000868 | | 0.000242336 | 0.00311071 |
| *nfu2-1* specific | **AT1G72160** | 9.66e-05 | | 3.33783e-05 | 0.0002797 |
| *nfu2-1* specific | **AT2G07698** | 0.00035 | | 7.39928e-05 | 0.00165575 |
| *nfu2-1* specific | **AT2G20990** | 8.08e-05 | | 3.09265e-05 | 0.000211156 |
| *nfu2-1* specific | **AT2G21620** | 0.000248 | | 9.13192e-05 | 0.000672087 |
| *nfu2-1* specific | **AT2G29550** | 9.98e-05 | | 2.10982e-05 | 0.000472348 |
| *nfu2-1* specific | **AT2G45470** | 0.00101 | | 0.000355277 | 0.00285071 |
| *nfu2-1* specific | **AT3G01310** | 1.35e-05 | | 5.07462e-06 | 3.57907e-05 |
| *nfu2-1* specific | **AT3G01670** | 2.98e-05 | | 1.11612e-05 | 7.94185e-05 |
| *nfu2-1* specific | **AT3G04610** | 6.24e-05 | | 2.53935e-05 | 0.000153429 |
| *nfu2-1* specific | **AT3G13460** | 4.69e-05 | | 1.92422e-05 | 0.000114431 |
| *nfu2-1* specific | **AT3G16470** | 0.00044 | | 0.000120462 | 0.00160458 |
| *nfu2-1* specific | **AT3G49870** | 9.37e-05 | | 3.77018e-05 | 0.000233115 |
| *nfu2-1* specific | **AT3G54760** | 3.1e-05 | | 1.30373e-05 | 7.35923e-05 |
| *nfu2-1* specific | **AT4G02570** | 2.49e-05 | | 9.59983e-06 | 6.43452e-05 |
| *nfu2-1* specific | **AT4G35630** | 0.000152 | | 6.54753e-05 | 0.000351332 |
| *nfu2-1* specific | **AT4G35830** | 0.000361 | | 0.000162799 | 0.000800063 |
| *nfu2-1* specific | **AT4G37925** | 0.0001 | | 2.71019e-05 | 0.000368919 |
| *nfu2-1* specific | **AT5G21160** | 1.28e-05 | | 4.81582e-06 | 3.38981e-05 |
| *nfu2-1* specific | **AT5G21430** | 0.000172 | | 5.80705e-05 | 0.00051194 |
| *nfu2-1* specific | **AT5G25980** | 0.000894 | | 0.000115242 | 0.00694018 |
| *nfu2-1* specific | **AT5G26000** | 0.00119 | | 0.000174216 | 0.00818781 |
| *nfu2-1* specific | **AT5G26570** | 8.4e-05 | | 2.00975e-05 | 0.000351055 |
| *nfu2-1* specific | **AT5G35180** | 1.6e-05 | | 6.27375e-06 | 4.0927e-05 |
| *nfu2-1* specific | **AT5G36880** | 6.21e-05 | | 2.04635e-05 | 0.000188422 |
| *nfu2-1* specific | **AT5G55230** | 9.71e-05 | | 3.36872e-05 | 0.000280045 |
| *nfu2-1* specific | **AT5G58260** | 8.3e-05 | | 2.68071e-05 | 0.000256713 |
| *nfu2-1* specific | **ATCG00740** | 3.79e-05 | | 1.2589e-05 | 0.000114218 |
| *nfu3-2* + *nfu2-1* | **AT2G17840** | 7.69e-05 | cytosol | 3.63325e-05 | 0.000162751 |
| *nfu3-2* + *nfu2-1* | **AT2G41740** | 8.12e-05 | cytosol | 2.47912e-05 | 0.000265969 |
| *nfu3-2* + *nfu2-1* | **AT3G53750** | 5.88e-05 | cytosol | 1.32073e-05 | 0.000261624 |
| *nfu3-2* + *nfu2-1* | **AT4G20890** | 4.29e-05 | cytosol | 6.67594e-06 | 0.000275302 |
| *nfu3-2* + *nfu2-1* | **AT4G36250** | 5.7e-05 | cytosol | 1.3284e-05 | 0.000244767 |
| *nfu3-2* + *nfu2-1* | **AT5G19780** | 0.000281 | cytosol | 0.000111181 | 0.000712557 |
| *nfu3-2* + *nfu2-1* | **AT5G20890** | 0.000239 | cytosol | 8.36791e-05 | 0.000683142 |
| *nfu3-2* + *nfu2-1* | **AT5G27470** | 0.000318 | cytosol | 0.000160186 | 0.000630864 |
| *nfu3-2* + *nfu2-1* | **AT5G62700** | 0.000177 | cytosol | 6.50461e-05 | 0.000480436 |
| *nfu3-2* + *nfu2-1* | **AT5G56360** | 5.78e-05 | endoplasmic reticulum | 1.39511e-05 | 0.000239544 |
| *nfu3-2* + *nfu2-1* | **AT5G60640** | 0.00032 | endoplasmic reticulum | 0.000109933 | 0.000931301 |
| *nfu3-2* + *nfu2-1* | **AT1G68560** | 0.000225 | extracellular | 7.4585e-05 | 0.00067969 |
| *nfu3-2* + *nfu2-1* | **AT5G25460** | 7.58e-05 | extracellular | 2.55253e-05 | 0.000224962 |
| *nfu3-2* + *nfu2-1* | **AT1G05010** | 0.000176 | golgi | 6.24311e-05 | 0.000493583 |
| *nfu3-2* + *nfu2-1* | **AT2G30520** | 2.2e-05 | golgi | 7.05238e-06 | 6.87895e-05 |
| *nfu3-2* + *nfu2-1* | **AT2G05710** | 0.000421 | mitochondrion | 0.000130018 | 0.00136107 |
| *nfu3-2* + *nfu2-1* | **AT5G52310** | 9.27e-05 | nucleus | 1.97535e-05 | 0.000434884 |
| *nfu3-2* + *nfu2-1* | **AT1G70940** | 1.05e-05 | plasma membrane | 3.12559e-06 | 3.49577e-05 |
| *nfu3-2* + *nfu2-1* | **AT5G16590** | 3.47e-05 | plasma membrane | 1.34122e-05 | 9.00115e-05 |
| *nfu3-2* + *nfu2-1* | **AT1G31330** | 0.000972 | plastid | 0.000211429 | 0.00446642 |
| *nfu3-2* + *nfu2-1* | **AT1G61520** | 0.00175 | plastid | 0.000336575 | 0.00911519 |
| *nfu3-2* + *nfu2-1* | **AT1G69830** | 8.05e-05 | plastid | 2.15371e-05 | 0.000301043 |
| *nfu3-2* + *nfu2-1* | **AT2G36250** | 6.48e-05 | plastid | 2.39555e-05 | 0.000175426 |
| *nfu3-2* + *nfu2-1* | **AT3G04870** | 4.05e-05 | plastid | 1.35069e-05 | 0.000121416 |

| | | | | | |
|---|---|---|---|---|---|
| *nfu3-2 + nfu2-1* | AT3G14930 | 0.000131 | plastid | 3.52185e-05 | 0.000484038 |
| *nfu3-2 + nfu2-1* | AT3G46780 | 0.000417 | plastid | 5.94951e-05 | 0.0029234 |
| *nfu3-2 + nfu2-1* | AT3G59780 | 9.54e-05 | plastid | 2.53574e-05 | 0.000359149 |
| *nfu3-2 + nfu2-1* | AT4G12800 | 0.000445 | plastid | 9.99233e-05 | 0.00197785 |
| *nfu3-2 + nfu2-1* | AT4G14210 | 4.49e-05 | plastid | 1.29534e-05 | 0.000155291 |
| *nfu3-2 + nfu2-1* | AT4G15110 | 3.71e-05 | plastid | 1.20881e-05 | 0.000113561 |
| *nfu3-2 + nfu2-1* | AT4G25080 | 0.000203 | plastid | 5.41333e-05 | 0.000757605 |
| *nfu3-2 + nfu2-1* | AT4G27440 | 0.000251 | plastid | 5.60046e-05 | 0.00112054 |
| *nfu3-2 + nfu2-1* | AT4G28750 | 0.00102 | plastid | 0.00022994 | 0.00449177 |
| *nfu3-2 + nfu2-1* | AT5G64040 | 0.000369 | plastid | 7.714e-05 | 0.00176354 |
| *nfu3-2 + nfu2-1* | AT5G64940 | 2.08e-05 | plastid | 2.88546e-06 | 0.000149628 |
| *nfu3-2 + nfu2-1* | AT5G67030 | 5.25e-05 | plastid | 1.10442e-05 | 0.00024937 |
| *nfu3-2 + nfu2-1* | ATCG00340 | 0.000638 | plastid | 0.000111714 | 0.00364374 |
| *nfu3-2 + nfu2-1* | ATCG00350 | 0.000479 | plastid | 7.81654e-05 | 0.00293495 |
| *nfu3-2 + nfu2-1* | ATCG01060 | 0.000844 | plastid | 0.000141185 | 0.0050475 |
| *nfu3-2 + nfu2-1* | AT1G07890 | 0.00148 | | 0.000247806 | 0.0088582 |
| *nfu3-2 + nfu2-1* | AT1G20010 | 0.000129 | | 3.76801e-05 | 0.000439169 |
| *nfu3-2 + nfu2-1* | AT1G20330 | 6.92e-05 | | 2.28837e-05 | 0.00020942 |
| *nfu3-2 + nfu2-1* | AT1G29660 | 0.000167 | | 2.957e-05 | 0.000943515 |
| *nfu3-2 + nfu2-1* | AT1G35720 | 0.000616 | | 0.000218881 | 0.00173627 |
| *nfu3-2 + nfu2-1* | AT2G07050 | 3.94e-05 | | 1.56865e-05 | 9.91046e-05 |
| *nfu3-2 + nfu2-1* | AT2G20190 | 2.2e-05 | | 7.98029e-06 | 6.04391e-05 |
| *nfu3-2 + nfu2-1* | AT2G21160 | 0.000404 | | 0.000142391 | 0.00114581 |
| *nfu3-2 + nfu2-1* | AT3G29360 | 0.00011 | | 3.68426e-05 | 0.000331026 |
| *nfu3-2 + nfu2-1* | AT3G54470 | 0.000108 | | 4.29574e-05 | 0.000272487 |
| *nfu3-2 + nfu2-1* | AT4G24190 | 0.000591 | | 0.00018244 | 0.00191381 |
| *nfu3-2 + nfu2-1* | AT4G29040 | 1.07e-05 | | 1.53901e-06 | 7.39605e-05 |
| *nfu3-2 + nfu2-1* | AT4G34200 | 0.000581 | | 0.000202382 | 0.00166532 |
| *nfu3-2 + nfu2-1* | AT5G11450 | 5.56e-05 | | 1.38504e-05 | 0.000223098 |
| *nfu3-2 + nfu2-1* | AT5G19690 | 6.7e-05 | | 2.18751e-05 | 0.000205027 |
| *nfu3-2 + nfu2-1* | AT5G42020 | 0.000159 | | 5.52506e-05 | 0.000457416 |
| *nfu3-2 + nfu2-1* | AT5G44340 | 0.000164 | | 4.08443e-05 | 0.000660056 |
| *nfu3-2 + nfu2-1* | AT5G52970 | 0.000108 | | 3.46468e-05 | 0.000337185 |
| *nfu3-2 + nfu2-1* | AT5G53460 | 8.39e-05 | | 2.42622e-05 | 0.000289971 |
| *nfu3-2 + nfu2-1* | AT5G54770 | 0.00042 | | 0.00010083 | 0.00175207 |
