# Supplementary material for: A Global Proteomic Approach Sheds New Light on Potential Iron-Sulfur Client Proteins of the Chloroplastic Maturation Factor NFU3
Source: Int J Mol Sci. 2020 Oct 30;21(21):8121. doi: 10.3390/ijms21218121 (PMC7672563; doi:10.3390/ijms21218121)
Supplement: Supplementary file 1 [file ijms-21-08121-s001.zip › ijms-973833 final suppl/Berger_et_al_IJMS_Table_S5_vIII.pdf]

Table S5: detailed localization of proteins under accumulated specifically in *nfu3-2*, or nfu2-1 or in both mutants. (NPAS = normalized protein abundance score)

Type of data	Protein ID	NPAS	HCM location	NPAS min	NPAS max
nfu3-2 specific	**AT1G02920**	0.000116	cytosol	3.5724e-05	0.000376219
nfu3-2 specific	**AT1G02930**	0.000216	cytosol	6.5045e-05	0.00072008
nfu3-2 specific	**AT1G06410**	4.62e-05	cytosol	1.86568e-05	0.000114208
nfu3-2 specific	**AT1G07110**	1.95e-05	cytosol	5.96215e-06	6.37715e-05
nfu3-2 specific	**AT1G12050**	4.28e-05	cytosol	1.53598e-05	0.000118999
nfu3-2 specific	**AT1G12780**	6.48e-05	cytosol	2.29824e-05	0.000182603
nfu3-2 specific	**AT1G23440**	4.55e-05	cytosol	1.54169e-05	0.000134108
nfu3-2 specific	**AT1G27020**	4.22e-05	cytosol	1.02774e-05	0.000172957
nfu3-2 specific	**AT1G35670**	1.56e-05	cytosol	4.40621e-06	5.49443e-05
nfu3-2 specific	**AT1G42550**	4.77e-05	cytosol	1.12893e-05	0.000201291
nfu3-2 specific	**AT1G54100**	0.00017	cytosol	4.07789e-05	0.000709717
nfu3-2 specific	**AT1G66200**	0.000279	cytosol	8.17943e-05	0.000949885
nfu3-2 specific	**AT1G69410**	8.82e-05	cytosol	2.95003e-05	0.000263405
nfu3-2 specific	**AT1G75280**	0.000169	cytosol	6.55346e-05	0.00043726
nfu3-2 specific	**AT1G80460**	7.69e-05	cytosol	1.45041e-05	0.000407402
nfu3-2 specific	**AT2G24200**	0.000419	cytosol	0.000160846	0.00109096
nfu3-2 specific	**AT2G29340**	0.000141	cytosol	5.76701e-05	0.000344543
nfu3-2 specific	**AT2G29360**	4.96e-05	cytosol	1.66083e-05	0.000147958
nfu3-2 specific	**AT3G18060**	5.8e-05	cytosol	1.9733e-05	0.000170185
nfu3-2 specific	**AT3G23570**	0.000139	cytosol	5.34473e-05	0.000362156
nfu3-2 specific	**AT3G26450**	0.000282	cytosol	0.000131407	0.000603437
nfu3-2 specific	**AT3G53180**	4.63e-05	cytosol	1.52881e-05	0.00014011
nfu3-2 specific	**AT3G55610**	2.93e-05	cytosol	1.13685e-05	7.54343e-05
nfu3-2 specific	**AT4G02520**	0.00086	cytosol	0.000235666	0.00313657
nfu3-2 specific	**AT4G24220**	7.67e-05	cytosol	3.31541e-05	0.000177318
nfu3-2 specific	**AT4G24810**	0.00001	cytosol	3.45172e-06	2.90931e-05
nfu3-2 specific	**AT4G33090**	0.000242	cytosol	6.77936e-05	0.00086722
nfu3-2 specific	**AT4G33640**	0.000169	cytosol	7.32036e-05	0.000391547
nfu3-2 specific	**AT4G36760**	9.68e-05	cytosol	3.48465e-05	0.000269121
nfu3-2 specific	**AT5G13370**	2.08e-05	cytosol	8.7116e-06	4.95629e-05
nfu3-2 specific	**AT5G16970**	0.000145	cytosol	5.70442e-05	0.000366309
nfu3-2 specific	**AT5G36160**	4.92e-05	cytosol	1.84961e-05	0.000130819
nfu3-2 specific	**AT5G39050**	1.63e-05	cytosol	6.03492e-06	4.42325e-05
nfu3-2 specific	**AT5G44020**	0.000222	cytosol	6.61118e-05	0.000744895
nfu3-2 specific	**AT5G49970**	4.9e-05	cytosol	2.04519e-05	0.000117316
nfu3-2 specific	**AT5G54960**	6.97e-05	cytosol	2.33197e-05	0.000208096
nfu3-2 specific	**AT4G31500**	4.7e-05	endoplasmic reticulur	1.90272e-05	0.000116291
nfu3-2 specific	**AT1G03220**	0.000203	extracellular	5.67172e-05	0.000728776
nfu3-2 specific	**AT1G03230**	0.000125	extracellular	4.00326e-05	0.000390976
nfu3-2 specific	**AT1G13900**	4.02e-05	extracellular	1.67033e-05	9.67178e-05
nfu3-2 specific	**AT1G21670**	5.35e-05	extracellular	1.78827e-05	0.000160159
nfu3-2 specific	**AT1G33590**	0.000325	extracellular	0.000117332	0.000899544
nfu3-2 specific	**AT1G33600**	5.35e-05	extracellular	2.00691e-05	0.000142872
nfu3-2 specific	**AT1G75750**	0.000125	extracellular	3.63568e-05	0.000431153

nfu3-2 specific	**AT1G76160**	0.000141	extracellular	3.9086e-05	0.000510794
nfu3-2 specific	**AT1G78830**	0.000262	extracellular	7.41418e-05	0.000924172
nfu3-2 specific	**AT1G78850**	0.000125	extracellular	3.79163e-05	0.000410394
nfu3-2 specific	**AT2G04690**	8.95e-05	extracellular	3.91872e-05	0.000204527
nfu3-2 specific	**AT2G05520**	0.000295	extracellular	8.03611e-05	0.00108154
nfu3-2 specific	**AT3G10740**	0.000181	extracellular	5.82949e-05	0.000561161
nfu3-2 specific	**AT3G13750**	0.00008	extracellular	2.46795e-05	0.000259329
nfu3-2 specific	**AT3G13790**	2.99e-05	extracellular	7.6157e-06	0.000117574
nfu3-2 specific	**AT3G16530**	9.3e-05	extracellular	3.17926e-05	0.000272049
nfu3-2 specific	**AT3G47800**	0.000172	extracellular	6.55496e-05	0.000449336
nfu3-2 specific	**AT3G51600**	0.000559	extracellular	0.000159457	0.00195998
nfu3-2 specific	**AT3G52500**	7.24e-05	extracellular	2.66252e-05	0.000196624
nfu3-2 specific	**AT3G55260**	5.68e-05	extracellular	2.08209e-05	0.000155012
nfu3-2 specific	**AT3G56310**	5.68e-05	extracellular	2.01566e-05	0.000160081
nfu3-2 specific	**AT4G00165**	7.08e-05	extracellular	2.06368e-05	0.000242981
nfu3-2 specific	**AT4G13340**	3.4e-05	extracellular	1.24154e-05	9.30017e-05
nfu3-2 specific	**AT4G19410**	0.00011	extracellular	4.35029e-05	0.000279131
nfu3-2 specific	**AT4G20830**	3.7e-05	extracellular	1.17914e-05	0.000116108
nfu3-2 specific	**AT4G25900**	8.21e-05	extracellular	2.47764e-05	0.000272102
nfu3-2 specific	**AT4G34180**	0.000107	extracellular	4.42158e-05	0.000258372
nfu3-2 specific	**AT5G13980**	0.000102	extracellular	3.72246e-05	0.000278582
nfu3-2 specific	**AT5G19860**	0.000116	extracellular	3.98094e-05	0.000339683
nfu3-2 specific	**AT5G34850**	0.000104	extracellular	4.07488e-05	0.000266743
nfu3-2 specific	**AT5G42240**	5.31e-05	extracellular	1.19199e-05	0.000236184
nfu3-2 specific	**AT5G64570**	7.5e-05	extracellular	1.67485e-05	0.000335859
nfu3-2 specific	**AT5G39590**	2.03e-05	golgi	7.42658e-06	5.53463e-05
nfu3-2 specific	**AT1G03090**	3.84e-05	mitochondrion	1.49943e-05	9.82808e-05
nfu3-2 specific	**AT1G06570**	3.05e-05	mitochondrion	1.20738e-05	7.68603e-05
nfu3-2 specific	**AT1G21400**	4.73e-06	mitochondrion	4.05091e-07	5.51282e-05
nfu3-2 specific	**AT1G54220**	2.66e-05	mitochondrion	1.33757e-05	5.29157e-05
nfu3-2 specific	**AT1G79440**	0.000171	mitochondrion	6.7341e-05	0.000435044
nfu3-2 specific	**AT2G43400**	1.11e-05	mitochondrion	4.64528e-06	2.64216e-05
nfu3-2 specific	**AT2G44350**	0.000283	mitochondrion	0.000100565	0.000793617
nfu3-2 specific	**AT3G13450**	1.62e-05	mitochondrion	3.56624e-06	7.33464e-05
nfu3-2 specific	**AT3G17240**	0.00021	mitochondrion	7.71875e-05	0.000568656
nfu3-2 specific	**AT3G48000**	0.00026	mitochondrion	9.40401e-05	0.000717353
nfu3-2 specific	**AT4G11600**	0.000315	mitochondrion	0.000138615	0.000714725
nfu3-2 specific	**AT4G26910**	4.59e-05	mitochondrion	1.56305e-05	0.000134941
nfu3-2 specific	**AT4G26970**	0.000271	mitochondrion	0.000102154	0.00072066
nfu3-2 specific	**AT4G34030**	3.22e-05	mitochondrion	1.26639e-05	8.18676e-05
nfu3-2 specific	**AT5G14780**	0.000357	mitochondrion	0.000119435	0.00106933
nfu3-2 specific	**AT5G18170**	8.37e-05	mitochondrion	2.42276e-05	0.000289191
nfu3-2 specific	**AT5G25940**	0.000147	mitochondrion	6.95185e-05	0.000312224
nfu3-2 specific	**AT5G32470**	9.1e-06	mitochondrion	2.23805e-06	3.69903e-05
nfu3-2 specific	**AT5G58270**	2.96e-05	mitochondrion	1.35023e-05	6.50973e-05
nfu3-2 specific	**AT5G62530**	0.000166	mitochondrion	7.26457e-05	0.000378133
nfu3-2 specific	**AT5G63620**	3.66e-05	mitochondrion	1.57539e-05	8.51943e-05
nfu3-2 specific	**AT1G27970**	0.000187	nucleus	5.91933e-05	0.000591456
nfu3-2 specific	**AT1G01820**	3.41e-05	peroxisome	1.38465e-05	8.39378e-05

nfu3-2 specific	**AT1G20630**	9.77e-05	peroxisome	2.76372e-05	0.00034517
nfu3-2 specific	**AT1G70580**	3.79e-05	peroxisome	9.23965e-06	0.000155342
nfu3-2 specific	**AT1G76180**	0.000897	peroxisome	0.000229301	0.00350817
nfu3-2 specific	**AT2G13360**	0.000652	peroxisome	0.000119794	0.00354413
nfu3-2 specific	**AT2G42490**	2.14e-05	peroxisome	8.54578e-06	5.35704e-05
nfu3-2 specific	**AT3G06810**	3.97e-05	peroxisome	1.51529e-05	0.000104263
nfu3-2 specific	**AT3G12800**	9.03e-05	peroxisome	3.26979e-05	0.000249397
nfu3-2 specific	**AT3G24170**	0.000209	peroxisome	9.11464e-05	0.000478848
nfu3-2 specific	**AT3G47430**	2.42e-05	peroxisome	5.41781e-06	0.000108249
nfu3-2 specific	**AT3G48170**	7,00E-05	peroxisome	2.70498e-05	0.000181242
nfu3-2 specific	**AT3G51840**	9.62e-05	peroxisome	3.87065e-05	0.000239004
nfu3-2 specific	**AT3G56460**	0.000135	peroxisome	5.93156e-05	0.000308297
nfu3-2 specific	**AT4G04320**	1.5e-05	peroxisome	6.84344e-06	3.27668e-05
nfu3-2 specific	**AT4G05160**	6.71e-05	peroxisome	2.23288e-05	0.000201804
nfu3-2 specific	**AT4G29010**	0.00018	peroxisome	6.92167e-05	0.000467844
nfu3-2 specific	**AT4G35000**	0.000252	peroxisome	8.45165e-05	0.000751895
nfu3-2 specific	**AT5G41210**	0.000103	peroxisome	4.96912e-05	0.000214493
nfu3-2 specific	**AT1G30360**	0.00017	plasma membrane	7.03305e-05	0.000409777
nfu3-2 specific	**AT1G59870**	8.86e-05	plasma membrane	2.12067e-05	0.000369965
nfu3-2 specific	**AT1G74790**	2.64e-05	plasma membrane	1.21128e-05	5.75304e-05
nfu3-2 specific	**AT2G30930**	0.000437	plasma membrane	0.000150091	0.00127518
nfu3-2 specific	**AT2G39480**	6.97e-06	plasma membrane	2.55851e-06	1.89629e-05
nfu3-2 specific	**AT3G02880**	8.46e-05	plasma membrane	2.66432e-05	0.000268793
nfu3-2 specific	**AT3G08510**	8.27e-05	plasma membrane	2.6466e-05	0.000258312
nfu3-2 specific	**AT3G19930**	3.34e-05	plasma membrane	7.17881e-06	0.000155117
nfu3-2 specific	**AT3G51550**	5.35e-05	plasma membrane	2.39897e-05	0.000119125
nfu3-2 specific	**AT4G08850**	2.79e-05	plasma membrane	1.05624e-05	7.37677e-05
nfu3-2 specific	**AT4G12420**	0.000253	plasma membrane	0.000114145	0.000561319
nfu3-2 specific	**AT4G27520**	0.000519	plasma membrane	0.000186078	0.00144929
nfu3-2 specific	**AT4G29900**	3.81e-05	plasma membrane	1.47841e-05	9.80205e-05
nfu3-2 specific	**AT5G20230**	0.000131	plasma membrane	2.16644e-05	0.000787019
nfu3-2 specific	**AT5G38990**	5.04e-06	plasma membrane	1.59524e-06	1.59124e-05
nfu3-2 specific	**AT5G58090**	4.2e-05	plasma membrane	1.65688e-05	0.000106435
nfu3-2 specific	**AT1G03600**	0.000642	plastid	0.000154464	0.00266488
nfu3-2 specific	**AT1G09830**	4.53e-05	plastid	1.75077e-05	0.000117004
nfu3-2 specific	**AT1G12900**	0.000588	plastid	0.000155458	0.00222516
nfu3-2 specific	**AT1G18170**	6.43e-05	plastid	2.0661e-05	0.000200359
nfu3-2 specific	**AT1G20340**	0.00409	plastid	0.000951788	0.0176172
nfu3-2 specific	**AT1G20810**	5.77e-05	plastid	1.47445e-05	0.000226105
nfu3-2 specific	**AT1G31160**	0.000103	plastid	4.18357e-05	0.000254234
nfu3-2 specific	**AT1G31190**	9.84e-05	plastid	2.4177e-05	0.000400345
nfu3-2 specific	**AT1G50250**	5.83e-05	plastid	1.60529e-05	0.000212005
nfu3-2 specific	**AT1G54500**	0.00016	plastid	6.27913e-05	0.000406132
nfu3-2 specific	**AT1G68830**	1.82e-05	plastid	2.94363e-06	0.000112735
nfu3-2 specific	**AT1G71480**	1.59e-05	plastid	3.15941e-06	8.01645e-05
nfu3-2 specific	**AT1G71810**	1.13e-05	plastid	2.39816e-06	5.3636e-05
nfu3-2 specific	**AT1G78620**	6.32e-06	plastid	2.15979e-06	1.85048e-05
nfu3-2 specific	**AT2G21960**	6.45e-05	plastid	1.87924e-05	0.000221283
nfu3-2 specific	**AT2G24820**	6.64e-05	plastid	1.76829e-05	0.000249472

nfu3-2 specific	**AT2G25080**	0.000283	plastid	8.26495e-05	0.000969238
nfu3-2 specific	**AT2G26930**	4.46e-05	plastid	1.13446e-05	0.000175031
nfu3-2 specific	**AT2G28605**	4.96e-05	plastid	1.62959e-05	0.000151166
nfu3-2 specific	**AT2G34460**	0.000183	plastid	4.82728e-05	0.000697038
nfu3-2 specific	**AT2G35490**	0.00032	plastid	0.000107275	0.000955476
nfu3-2 specific	**AT2G41040**	2.08e-05	plastid	8.40215e-06	5.16911e-05
nfu3-2 specific	**AT2G43180**	2.98e-05	plastid	1.05233e-05	8.46663e-05
nfu3-2 specific	**AT2G43945**	6.19e-05	plastid	2.5704e-05	0.00014926
nfu3-2 specific	**AT2G44050**	0.000109	plastid	3.42881e-05	0.000344584
nfu3-2 specific	**AT2G44920**	0.000341	plastid	8.60221e-05	0.0013501
nfu3-2 specific	**AT2G46910**	2.93e-05	plastid	8.12035e-06	0.000105891
nfu3-2 specific	**AT2G47730**	0.00113	plastid	0.000385874	0.00329255
nfu3-2 specific	**AT2G47840**	6.61e-05	plastid	2.36218e-05	0.000185045
nfu3-2 specific	**AT3G08920**	5.87e-05	plastid	2.20601e-05	0.000156091
nfu3-2 specific	**AT3G10060**	0.00012	plastid	2.25083e-05	0.000643433
nfu3-2 specific	**AT3G10130**	1.86e-05	plastid	6.01129e-06	5.74298e-05
nfu3-2 specific	**AT3G18890**	0.000248	plastid	4.74901e-05	0.0012974
nfu3-2 specific	**AT3G23070**	3.09e-06	plastid	5.27103e-07	1.80654e-05
nfu3-2 specific	**AT3G23700**	0.000173	plastid	5.36037e-05	0.000558743
nfu3-2 specific	**AT3G26070**	0.000207	plastid	5.75811e-05	0.000742219
nfu3-2 specific	**AT3G43520**	8.93e-05	plastid	4.0048e-05	0.000199329
nfu3-2 specific	**AT3G44880**	3.59e-05	plastid	1.29458e-05	9.97986e-05
nfu3-2 specific	**AT3G45140**	0.00147	plastid	0.000463426	0.00463468
nfu3-2 specific	**AT3G50820**	0.000696	plastid	0.000162979	0.00297418
nfu3-2 specific	**AT3G52960**	0.000924	plastid	0.000382679	0.00222908
nfu3-2 specific	**AT3G54660**	9.6e-05	plastid	3.02031e-05	0.000305453
nfu3-2 specific	**AT3G55330**	0.000284	plastid	6.95537e-05	0.00115873
nfu3-2 specific	**AT3G56650**	0.000174	plastid	3.3497e-05	0.000907538
nfu3-2 specific	**AT3G58010**	9.75e-05	plastid	2.56352e-05	0.000371051
nfu3-2 specific	**AT3G60750**	0.00204	plastid	0.000382538	0.0108995
nfu3-2 specific	**AT3G62030**	0.00138	plastid	0.000232646	0.00813493
nfu3-2 specific	**AT4G00490**	7.43e-05	plastid	2.66106e-05	0.000207357
nfu3-2 specific	**AT4G04850**	6.45e-06	plastid	2.19228e-06	1.89586e-05
nfu3-2 specific	**AT4G10300**	0.000195	plastid	5.15127e-05	0.000741561
nfu3-2 specific	**AT4G10750**	4.8e-05	plastid	2.0185e-05	0.00011426
nfu3-2 specific	**AT4G13500**	8.66e-05	plastid	3.42676e-05	0.000218639
nfu3-2 specific	**AT4G14070**	2.95e-05	plastid	8.61623e-06	0.000100986
nfu3-2 specific	**AT4G14870**	9.6e-05	plastid	3.02916e-05	0.000304307
nfu3-2 specific	**AT4G18240**	9.04e-06	plastid	2.45462e-06	3.3295e-05
nfu3-2 specific	**AT4G19170**	5.52e-05	plastid	1.20533e-05	0.000252865
nfu3-2 specific	**AT4G20850**	0.000208	plastid	8.19106e-05	0.000526599
nfu3-2 specific	**AT4G21280**	0.00159	plastid	0.000347266	0.00732037
nfu3-2 specific	**AT4G21445**	0.000137	plastid	4.91917e-05	0.000381543
nfu3-2 specific	**AT4G22240**	0.00038	plastid	0.000152708	0.00094489
nfu3-2 specific	**AT4G22890**	0.000294	plastid	6.73369e-05	0.00127932
nfu3-2 specific	**AT4G23100**	0.000247	plastid	8.95644e-05	0.000678427
nfu3-2 specific	**AT4G24620**	0.00029	plastid	0.000129957	0.000648112
nfu3-2 specific	**AT4G25450**	5.16e-05	plastid	1.3189e-05	0.000202028
nfu3-2 specific	**AT4G25650**	1.32e-05	plastid	6.3165e-06	2.76099e-05

nfu3-2 specific	**AT4G30690**	2.4e-05	plastid	6.56746e-06	8.74791e-05
nfu3-2 specific	**AT4G30910**	1.2e-05	plastid	3.51305e-06	4.06608e-05
nfu3-2 specific	**AT4G30920**	6.84e-05	plastid	2.98342e-05	0.000157019
nfu3-2 specific	**AT4G34240**	2.09e-05	plastid	6.60158e-06	6.64181e-05
nfu3-2 specific	**AT5G02940**	3.18e-05	plastid	5.90365e-06	0.000171152
nfu3-2 specific	**AT5G22510**	2.12e-05	plastid	8.20497e-06	5.48401e-05
nfu3-2 specific	**AT5G23120**	0.00046	plastid	0.000101966	0.00207598
nfu3-2 specific	**AT5G42650**	0.000205	plastid	3.1001e-05	0.00136066
nfu3-2 specific	**AT1G30400**	2.83e-05	vacuole	7.00679e-06	0.000114193
nfu3-2 specific	**AT1G75220**	2.43e-05	vacuole	1.18019e-05	5.02154e-05
nfu3-2 specific	**AT2G21410**	6.25e-05	vacuole	2.36311e-05	0.000165526
nfu3-2 specific	**AT2G41560**	4.94e-05	vacuole	1.36896e-05	0.000178546
nfu3-2 specific	**AT3G62700**	2.59e-05	vacuole	8.9957e-06	7.45087e-05
nfu3-2 specific	**AT4G02620**	0.00021	vacuole	6.73892e-05	0.00065357
nfu3-2 specific	**AT4G39080**	0.000213	vacuole	7.29208e-05	0.000623127
nfu3-2 specific	**AT5G14120**	4.97e-05	vacuole	1.89557e-05	0.000130279
nfu3-2 specific	**AT5G60360**	0.000208	vacuole	8.3519e-05	0.00051917
nfu3-2 specific	**AT1G02475**	3.13e-05		9.19059e-06	0.000106347
nfu3-2 specific	**AT1G05560**	3.02e-05		8.77918e-06	0.000103622
nfu3-2 specific	**AT1G09010**	6.38e-05		2.67373e-05	0.000152008
nfu3-2 specific	**AT1G10360**	4,00E-05		1.37172e-05	0.000116588
nfu3-2 specific	**AT1G10370**	0.000259		0.000102458	0.000657181
nfu3-2 specific	**AT1G12840**	0.000552		0.000252908	0.00120676
nfu3-2 specific	**AT1G17290**	0.000206		8.7703e-05	0.000482878
nfu3-2 specific	**AT1G18270**	7.73e-05		2.16438e-05	0.000276138
nfu3-2 specific	**AT1G20620**	0.00103		0.000263499	0.00400757
nfu3-2 specific	**AT1G20816**	1.32e-05		3.86432e-06	4.51947e-05
nfu3-2 specific	**AT1G49630**	1.72e-05		5.13169e-06	5.79775e-05
nfu3-2 specific	**AT1G49670**	5.35e-05		1.75029e-05	0.000163673
nfu3-2 specific	**AT1G49750**	0.000146		3.29962e-05	0.000644403
nfu3-2 specific	**AT1G52400**	0.000349		5.90019e-05	0.0020655
nfu3-2 specific	**AT1G55850**	1.77e-05		6.76792e-06	4.61187e-05
nfu3-2 specific	**AT1G58270**	4.54e-05		1.43041e-05	0.000143788
nfu3-2 specific	**AT1G65590**	4.44e-05		1.64125e-05	0.000120016
nfu3-2 specific	**AT1G69840**	7.9e-05		2.656e-05	0.000235084
nfu3-2 specific	**AT1G70290**	9.03e-06		4.33598e-06	1.87955e-05
nfu3-2 specific	**AT1G72160**	9.66e-05		3.33783e-05	0.0002797
nfu3-2 specific	**AT1G73650**	5.81e-05		1.98491e-05	0.00016992
nfu3-2 specific	**AT1G76030**	5.79e-05		7.68841e-06	0.000435798
nfu3-2 specific	**AT1G78900**	0.00138		0.000554525	0.00341824
nfu3-2 specific	**AT2G05380**	8.63e-05		1.89413e-05	0.00039284
nfu3-2 specific	**AT2G22990**	0.000107		3.09459e-05	0.000371529
nfu3-2 specific	**AT2G24270**	0.000521		0.000166845	0.00162761
nfu3-2 specific	**AT2G26740**	5.05e-05		1.74502e-05	0.000145857
nfu3-2 specific	**AT2G31170**	2.55e-05		1.05714e-05	6.1697e-05
nfu3-2 specific	**AT2G32080**	0.000105		3.43534e-05	0.000323522
nfu3-2 specific	**AT2G34310**	3.82e-05		1.70812e-05	8.53766e-05
nfu3-2 specific	**AT2G35780**	5.22e-05		2.41824e-05	0.00011287
nfu3-2 specific	**AT2G35840**	0.000106		4.61579e-05	0.000244146

nfu3-2 specific	**AT2G37770**	4.83e-05	1.30247e-05	0.000179185
nfu3-2 specific	**AT2G41100**	6.15e-05	1.75704e-05	0.000214981
nfu3-2 specific	**AT2G43820**	4.14e-05	1.5303e-05	0.000112175
nfu3-2 specific	**AT2G44310**	0.000237	5.81127e-05	0.000965359
nfu3-2 specific	**AT3G01520**	6.86e-05	2.89801e-05	0.000162536
nfu3-2 specific	**AT3G03980**	5.84e-05	1.8909e-05	0.000180522
nfu3-2 specific	**AT3G06510**	4.19e-05	1.52916e-05	0.000114562
nfu3-2 specific	**AT3G12010**	9.32e-06	3.6392e-06	2.38762e-05
nfu3-2 specific	**AT3G21790**	1.83e-05	7.03641e-06	4.75328e-05
nfu3-2 specific	**AT3G22200**	0.000269	8.28229e-05	0.000872613
nfu3-2 specific	**AT3G23490**	0.000285	9.31419e-05	0.000870981
nfu3-2 specific	**AT3G23920**	2.61e-05	9.34331e-06	7.311e-05
nfu3-2 specific	**AT3G26720**	5.08e-05	1.07157e-05	0.000240933
nfu3-2 specific	**AT3G27820**	2,00E-05	8.77836e-06	4.57428e-05
nfu3-2 specific	**AT3G27890**	0.000221	8.30666e-05	0.000586418
nfu3-2 specific	**AT3G42050**	0.000212	9.30814e-05	0.000481194
nfu3-2 specific	**AT3G44300**	9.66e-05	3.42767e-05	0.000272033
nfu3-2 specific	**AT3G48990**	0.000337	0.00011761	0.000963891
nfu3-2 specific	**AT3G54440**	4.03e-05	1.25986e-05	0.000129209
nfu3-2 specific	**AT3G58730**	0.000271	0.000104187	0.000706106
nfu3-2 specific	**AT4G01870**	4.25e-05	1.38189e-05	0.000130594
nfu3-2 specific	**AT4G10060**	2.75e-05	6.31883e-06	0.00011951
nfu3-2 specific	**AT4G11150**	0.000478	0.000125249	0.00182543
nfu3-2 specific	**AT4G15530**	7.14e-05	9.69429e-06	0.000525906
nfu3-2 specific	**AT4G19880**	4.77e-05	9.90229e-06	0.000229757
nfu3-2 specific	**AT4G20260**	0.0015	0.000273642	0.00826344
nfu3-2 specific	**AT4G20860**	3.79e-05	1.53366e-05	9.36837e-05
nfu3-2 specific	**AT4G30310**	3.16e-05	1.42797e-05	6.98558e-05
nfu3-2 specific	**AT4G32770**	3,00E-05	8.26698e-06	0.000109144
nfu3-2 specific	**AT4G34120**	0.000146	4.68374e-05	0.000456146
nfu3-2 specific	**AT4G34138**	2.7e-05	4.00296e-06	0.000182289
nfu3-2 specific	**AT4G35760**	3.12e-05	1.22514e-05	7.92423e-05
nfu3-2 specific	**AT4G38810**	0.000122	4.87215e-05	0.000305283
nfu3-2 specific	**AT4G39710**	9.46e-05	3.70628e-05	0.000241499
nfu3-2 specific	**AT4G39730**	0.000539	0.000232085	0.00125316
nfu3-2 specific	**AT5G07020**	0.000287	8.66596e-05	0.000950564
nfu3-2 specific	**AT5G08410**	0.000134	4.06499e-05	0.000440374
nfu3-2 specific	**AT5G08740**	4.06e-05	1.12787e-05	0.000146436
nfu3-2 specific	**AT5G10470**	4.11e-05	9.60284e-06	0.000175715
nfu3-2 specific	**AT5G11720**	3.62e-05	1.33458e-05	9.80553e-05
nfu3-2 specific	**AT5G16150**	9.08e-05	3.25941e-05	0.000252771
nfu3-2 specific	**AT5G17170**	0.00021	5.61892e-05	0.000784151
nfu3-2 specific	**AT5G17380**	7.04e-05	2.82439e-05	0.000175238
nfu3-2 specific	**AT5G19220**	0.000157	3.05054e-05	0.000803483
nfu3-2 specific	**AT5G25980**	0.000894	0.000115242	0.00694018
nfu3-2 specific	**AT5G27380**	6.39e-05	2.73015e-05	0.000149338
nfu3-2 specific	**AT5G27390**	3.89e-05	9.28455e-06	0.000163129
nfu3-2 specific	**AT5G42270**	0.000174	4.15013e-05	0.00072798
nfu3-2 specific	**AT5G42980**	0.00108	0.000331089	0.00353634

nfu3-2 specific	**AT5G44130**	0.000133		3.74841e-05	0.000475365
nfu3-2 specific	**AT5G46110**	0.000176		4.06504e-05	0.000762384
nfu3-2 specific	**AT5G48300**	0.00029		0.000101817	0.000827971
nfu3-2 specific	**AT5G50640**	1.04e-05		2.67471e-06	4.03761e-05
nfu3-2 specific	**AT5G51070**	1.84e-05		3.45042e-06	9.82574e-05
nfu3-2 specific	**AT5G51820**	0.00019		5.33616e-05	0.000673271
nfu3-2 specific	**AT5G54500**	0.000312		0.000111394	0.000875542
nfu3-2 specific	**AT5G57170**	6.93e-05		1.10253e-05	0.00043585
nfu3-2 specific	**AT5G57655**	0.00023		6.20478e-05	0.000855421
nfu3-2 specific	**AT5G58330**	0.000344		0.000120133	0.000987458
nfu3-2 specific	**AT5G59250**	1.86e-05		6.33609e-06	5.4565e-05
nfu3-2 specific	**AT5G59750**	1.04e-05		3.63459e-06	2.99623e-05
nfu3-2 specific	**AT5G64250**	7.84e-05		3.56564e-05	0.000172439
nfu3-2 specific	**AT5G65010**	7.9e-05		2.03492e-05	0.00030661
nfu3-2 specific	**AT5G66570**	0.000934		0.000186144	0.00469067
nfu3-2 specific	**AT5G67370**	8.19e-06		2.01284e-06	3.33632e-05
nfu3-2 specific	**ATCG00120**	0.00291		0.000378807	0.0223987
nfu3-2 specific	**ATCG00280**	0.00152		0.000254511	0.00910645
nfu3-2 specific	**ATCG00480**	0.0029		0.000450604	0.0186045
nfu3-2 specific	**ATCG00810**	0.000111		2.56982e-05	0.000478251
nfu3-2 specific	**ATCG00820**	0.00021		4.38899e-05	0.00100596
nfu2-1 specific	**AT1G12310**	0.0002	cytosol	5.31642e-05	0.000755529
nfu2-1 specific	**AT1G17890**	9.3e-05	cytosol	3.49157e-05	0.000247775
nfu2-1 specific	**AT1G19670**	0.000178	cytosol	6.64011e-05	0.000477066
nfu2-1 specific	**AT1G22430**	1.96e-05	cytosol	7.94215e-06	4.82061e-05
nfu2-1 specific	**AT1G52100**	9.62e-06	cytosol	2.24861e-06	4.1163e-05
nfu2-1 specific	**AT1G69620**	0.00032	cytosol	0.000144498	0.000708149
nfu2-1 specific	**AT1G77940**	0.000171	cytosol	7.9995e-05	0.000363486
nfu2-1 specific	**AT2G05220**	0.000326	cytosol	0.000116776	0.000909978
nfu2-1 specific	**AT2G20450**	0.000146	cytosol	5.7819e-05	0.00036707
nfu2-1 specific	**AT2G27710**	0.000901	cytosol	0.000163313	0.00496985
nfu2-1 specific	**AT2G35810**	0.000125	cytosol	4.42652e-05	0.000352831
nfu2-1 specific	**AT2G38230**	0.000201	cytosol	5.41457e-05	0.000743326
nfu2-1 specific	**AT2G45710**	0.000334	cytosol	0.000138588	0.00080703
nfu2-1 specific	**AT3G02560**	0.00066	cytosol	0.000258282	0.00168447
nfu2-1 specific	**AT3G03780**	0.000478	cytosol	0.000133548	0.0017099
nfu2-1 specific	**AT3G08590**	0.000351	cytosol	0.000151115	0.00081733
nfu2-1 specific	**AT3G18740**	7,00E-05	cytosol	1.60403e-05	0.000305395
nfu2-1 specific	**AT3G48690**	0.000119	cytosol	5.03479e-05	0.000283027
nfu2-1 specific	**AT3G49910**	0.00108	cytosol	0.000365076	0.00321962
nfu2-1 specific	**AT3G53430**	0.000103	cytosol	3.40249e-05	0.000309303
nfu2-1 specific	**AT4G09670**	0.000108	cytosol	4.78746e-05	0.000244461
nfu2-1 specific	**AT4G13930**	0.000714	cytosol	0.00025855	0.00197316
nfu2-1 specific	**AT4G14880**	0.000875	cytosol	0.000322701	0.00236986
nfu2-1 specific	**AT4G15000**	0.000348	cytosol	8.76595e-05	0.00138398
nfu2-1 specific	**AT4G23670**	0.00184	cytosol	0.000570914	0.00593872
nfu2-1 specific	**AT5G01410**	0.00016	cytosol	4.59645e-05	0.000557239
nfu2-1 specific	**AT5G06060**	0.00016	cytosol	7.66256e-05	0.000334344
nfu2-1 specific	**AT5G17310**	0.000146	cytosol	3.98473e-05	0.000533518

nfu2-1 specific	**AT5G28840**	0.000343	cytosol	0.000145017	0.000810489
nfu2-1 specific	**AT5G42850**	7.44e-05	cytosol	3.04968e-05	0.000181468
nfu2-1 specific	**AT5G54160**	0.000193	cytosol	5.84161e-05	0.000640624
nfu2-1 specific	**AT5G54760**	5.03e-05	cytosol	1.98939e-05	0.000127233
nfu2-1 specific	**AT5G59850**	0.000137	cytosol	4.406e-05	0.000425113
nfu2-1 specific	**AT1G71950**	0.000188	extracellular	6.25825e-05	0.000566131
nfu2-1 specific	**AT2G05920**	0.000113	extracellular	3.12479e-05	0.000407911
nfu2-1 specific	**AT3G44100**	0.000445	extracellular	0.000201698	0.000983364
nfu2-1 specific	**AT5G23210**	4.01e-05	extracellular	1.21529e-05	0.00013263
nfu2-1 specific	**AT5G23820**	0.000108	extracellular	3.79341e-05	0.000310223
nfu2-1 specific	**AT5G57560**	1.1e-05	extracellular	1.49311e-06	8.17402e-05
nfu2-1 specific	**AT2G40765**	0.000164	mitochondrion	5.54405e-05	0.000486532
nfu2-1 specific	**AT2G42210**	0.000173	mitochondrion	4.38437e-05	0.00068006
nfu2-1 specific	**AT3G15090**	7.58e-05	mitochondrion	2.70209e-05	0.000212773
nfu2-1 specific	**AT3G52730**	0.000251	mitochondrion	0.000104879	0.000601
nfu2-1 specific	**AT3G62530**	0.000252	mitochondrion	9.35377e-05	0.000678813
nfu2-1 specific	**AT4G00860**	0.000146	mitochondrion	5.44885e-05	0.000389816
nfu2-1 specific	**AT4G32470**	0.000738	mitochondrion	0.000336064	0.00162184
nfu2-1 specific	**AT5G43970**	0.000169	mitochondrion	7.03855e-05	0.000406118
nfu2-1 specific	**AT5G46800**	0.00022	mitochondrion	9.89914e-05	0.000491095
nfu2-1 specific	**AT5G52840**	0.000268	mitochondrion	0.000111662	0.000641688
nfu2-1 specific	**AT1G09760**	0.000199	nucleus	7.59225e-05	0.000520678
nfu2-1 specific	**AT1G73230**	0.000838	nucleus	0.000343093	0.0020492
nfu2-1 specific	**AT5G04280**	5.85e-05	nucleus	2.31846e-05	0.000147374
nfu2-1 specific	**AT2G42770**	1.63e-05	peroxisome	4.24578e-06	6.2903e-05
nfu2-1 specific	**AT3G15450**	5.09e-05	peroxisome	1.15601e-05	0.000224289
nfu2-1 specific	**AT1G18210**	0.000213	plasma membrane	6.35415e-05	0.000711837
nfu2-1 specific	**AT1G01790**	3.56e-05	plastid	6.92454e-06	0.000183215
nfu2-1 specific	**AT1G03680**	0.000815	plastid	0.000267106	0.00248896
nfu2-1 specific	**AT1G04420**	0.000165	plastid	3.67486e-05	0.000739837
nfu2-1 specific	**AT1G05190**	0.000564	plastid	0.000146063	0.00218102
nfu2-1 specific	**AT1G06950**	0.00026	plastid	7.32513e-05	0.000920964
nfu2-1 specific	**AT1G07320**	0.000401	plastid	7.63961e-05	0.00210244
nfu2-1 specific	**AT1G09130**	8.99e-05	plastid	2.83899e-05	0.000284722
nfu2-1 specific	**AT1G09340**	0.000762	plastid	0.000103088	0.00563677
nfu2-1 specific	**AT1G11430**	0.000152	plastid	4.60591e-05	0.00050446
nfu2-1 specific	**AT1G11750**	0.000108	plastid	3.75243e-05	0.000311829
nfu2-1 specific	**AT1G12410**	0.000202	plastid	6.5808e-05	0.00062241
nfu2-1 specific	**AT1G14345**	4.77e-05	plastid	1.26114e-05	0.000180053
nfu2-1 specific	**AT1G15140**	0.000244	plastid	6.79807e-05	0.000877429
nfu2-1 specific	**AT1G16080**	0.000301	plastid	9.68353e-05	0.000938245
nfu2-1 specific	**AT1G18060**	7.02e-05	plastid	1.61839e-05	0.0003047
nfu2-1 specific	**AT1G22700**	4.41e-05	plastid	1.08692e-05	0.000178646
nfu2-1 specific	**AT1G32060**	0.00107	plastid	0.000172194	0.00660125
nfu2-1 specific	**AT1G32990**	0.000302	plastid	6.1682e-05	0.00147593
nfu2-1 specific	**AT1G35680**	0.000311	plastid	8.59157e-05	0.00112475
nfu2-1 specific	**AT1G42970**	0.00114	plastid	0.000205146	0.00633597
nfu2-1 specific	**AT1G48350**	0.000203	plastid	3.95457e-05	0.00104679
nfu2-1 specific	**AT1G55490**	0.000157	plastid	3.43654e-05	0.000712983

nfu2-1 specific	**AT1G56190**	0.000426	plastid	0.000118238	0.00153799
nfu2-1 specific	**AT1G62780**	0.000342	plastid	0.000104262	0.00112356
nfu2-1 specific	**AT1G63970**	9.48e-05	plastid	2.75052e-05	0.000326914
nfu2-1 specific	**AT1G66670**	0.000168	plastid	5.73804e-05	0.000493616
nfu2-1 specific	**AT1G68590**	0.000162	plastid	4.64821e-05	0.000563103
nfu2-1 specific	**AT1G70070**	1.45e-05	plastid	2.88182e-06	7.31078e-05
nfu2-1 specific	**AT1G74470**	0.000408	plastid	8.61773e-05	0.00192931
nfu2-1 specific	**AT1G75350**	0.000196	plastid	4.72962e-05	0.0008097
nfu2-1 specific	**AT1G75460**	1.41e-05	plastid	2.81152e-06	7.07453e-05
nfu2-1 specific	**AT1G77060**	3.5e-05	plastid	8.61724e-06	0.000142251
nfu2-1 specific	**AT1G78630**	0.000322	plastid	9.92129e-05	0.00104716
nfu2-1 specific	**AT1G80300**	5.55e-05	plastid	1.89284e-05	0.000162449
nfu2-1 specific	**AT2G04030**	0.000473	plastid	0.000148716	0.00150185
nfu2-1 specific	**AT2G04700**	0.000118	plastid	3.49741e-05	0.000395057
nfu2-1 specific	**AT2G14880**	0.000194	plastid	7.47266e-05	0.000503819
nfu2-1 specific	**AT2G18710**	5.22e-05	plastid	1.0403e-05	0.000261831
nfu2-1 specific	**AT2G21370**	3.02e-05	plastid	7.93267e-06	0.000114742
nfu2-1 specific	**AT2G22360**	2.77e-05	plastid	7.11563e-06	0.000107729
nfu2-1 specific	**AT2G24020**	0.000203	plastid	5.51497e-05	0.000744725
nfu2-1 specific	**AT2G26540**	7.72e-05	plastid	1.27421e-05	0.000468238
nfu2-1 specific	**AT2G27290**	3.9e-05	plastid	1.61546e-05	9.39665e-05
nfu2-1 specific	**AT2G28000**	0.00156	plastid	0.000352564	0.00690561
nfu2-1 specific	**AT2G33450**	7.32e-05	plastid	2.20887e-05	0.000242785
nfu2-1 specific	**AT2G33800**	0.000456	plastid	0.000105029	0.00197548
nfu2-1 specific	**AT2G37220**	0.000917	plastid	0.000186883	0.00450426
nfu2-1 specific	**AT2G38140**	0.000145	plastid	3.43431e-05	0.000610762
nfu2-1 specific	**AT2G40100**	0.000213	plastid	5.08171e-05	0.000895066
nfu2-1 specific	**AT2G43030**	0.000427	plastid	0.000124201	0.001465
nfu2-1 specific	**AT2G45300**	2.69e-05	plastid	1.04826e-05	6.92621e-05
nfu2-1 specific	**AT3G01500**	0.00109	plastid	0.000144104	0.00821957
nfu2-1 specific	**AT3G02730**	0.000279	plastid	6.36369e-05	0.00121922
nfu2-1 specific	**AT3G02900**	3.29e-05	plastid	1.39526e-05	7.76161e-05
nfu2-1 specific	**AT3G06730**	5.38e-05	plastid	1.2551e-05	0.000230411
nfu2-1 specific	**AT3G13120**	0.000196	plastid	5.7812e-05	0.000665777
nfu2-1 specific	**AT3G13470**	0.000139	plastid	3.67233e-05	0.000527283
nfu2-1 specific	**AT3G15190**	0.000197	plastid	5.45541e-05	0.000712454
nfu2-1 specific	**AT3G18390**	6.47e-06	plastid	1.48263e-06	2.82092e-05
nfu2-1 specific	**AT3G25920**	0.000309	plastid	8.07725e-05	0.00118063
nfu2-1 specific	**AT3G26900**	2.84e-05	plastid	7.74401e-06	0.000103839
nfu2-1 specific	**AT3G52150**	0.000744	plastid	0.000186556	0.00296704
nfu2-1 specific	**AT3G54210**	9.84e-05	plastid	2.14544e-05	0.000451533
nfu2-1 specific	**AT3G54900**	0.000154	plastid	5.41317e-05	0.000438509
nfu2-1 specific	**AT3G55040**	0.00019	plastid	7.09115e-05	0.000509574
nfu2-1 specific	**AT3G55800**	0.000839	plastid	0.000118319	0.00594876
nfu2-1 specific	**AT3G56910**	0.000132	plastid	4.08174e-05	0.000430023
nfu2-1 specific	**AT3G59980**	0.000136	plastid	5.40153e-05	0.000340989
nfu2-1 specific	**AT3G63160**	0.000411	plastid	0.00014402	0.00117474
nfu2-1 specific	**AT3G63490**	0.00047	plastid	9.27376e-05	0.0023773
nfu2-1 specific	**AT4G01150**	0.000466	plastid	0.000170488	0.00127396

nfu2-1 specific	**AT4G01310**	0.000543	plastid	0.000164984	0.00178838
nfu2-1 specific	**AT4G01690**	0.000102	plastid	2.59577e-05	0.00040046
nfu2-1 specific	**AT4G04350**	3.11e-05	plastid	7.16652e-06	0.000134556
nfu2-1 specific	**AT4G09040**	0.000136	plastid	4.15529e-05	0.000444691
nfu2-1 specific	**AT4G12060**	0.000227	plastid	7.40345e-05	0.000698633
nfu2-1 specific	**AT4G21210**	9.82e-05	plastid	3.34015e-05	0.000288966
nfu2-1 specific	**AT4G24280**	0.000397	plastid	0.000124965	0.00126261
nfu2-1 specific	**AT4G28660**	0.000181	plastid	4.97359e-05	0.000661582
nfu2-1 specific	**AT4G29590**	5.31e-05	plastid	1.95097e-05	0.000144494
nfu2-1 specific	**AT4G29670**	7.6e-05	plastid	2.09128e-05	0.000275931
nfu2-1 specific	**AT4G30950**	1.72e-05	plastid	4.28814e-06	6.87302e-05
nfu2-1 specific	**AT4G33510**	9.14e-05	plastid	3.7661e-05	0.000221839
nfu2-1 specific	**AT5G14320**	0.00017	plastid	3.70259e-05	0.000784991
nfu2-1 specific	**AT5G14910**	0.00054	plastid	0.000148102	0.00196961
nfu2-1 specific	**AT5G16660**	0.000106	plastid	3.92779e-05	0.000284654
nfu2-1 specific	**AT5G23440**	0.000115	plastid	4.46829e-05	0.000293908
nfu2-1 specific	**AT5G24490**	0.000433	plastid	0.000174482	0.00107568
nfu2-1 specific	**AT5G55220**	0.000255	plastid	7.09245e-05	0.00091714
nfu2-1 specific	**AT5G62140**	7.61e-05	plastid	2.29992e-05	0.000251977
nfu2-1 specific	**AT5G65220**	0.000265	plastid	7.82958e-05	0.00089927
nfu2-1 specific	**AT5G66120**	8.06e-05	plastid	3.29992e-05	0.000197055
nfu2-1 specific	**ATCG00380**	0.000282	plastid	6.08579e-05	0.00130672
nfu2-1 specific	**ATCG01120**	0.000422	plastid	0.000122742	0.00145407
nfu2-1 specific	**AT1G11910**	0.000436	vacuole	0.000180377	0.00105533
nfu2-1 specific	**AT1G15690**	0.000639	vacuole	0.00018547	0.00220338
nfu2-1 specific	**AT1G54010**	0.000102	vacuole	2.42556e-05	0.000425431
nfu2-1 specific	**AT1G11860**	0.000813		0.000182098	0.00363189
nfu2-1 specific	**AT1G15500**	4.79e-05		1.48282e-05	0.000154584
nfu2-1 specific	**AT1G26550**	0.000154		7.30394e-05	0.000326425
nfu2-1 specific	**AT1G70410**	0.000249		6.62321e-05	0.000939807
nfu2-1 specific	**AT1G78915**	3.77e-05		1.15336e-05	0.000123542
nfu2-1 specific	**AT2G26340**	0.000108		2.2938e-05	0.000504468
nfu2-1 specific	**AT2G29290**	4.46e-05		1.03214e-05	0.000192601
nfu2-1 specific	**AT3G16420**	0.000417		4.40818e-05	0.00394259
nfu2-1 specific	**AT3G19450**	0.000117		4.19558e-05	0.00032815
nfu2-1 specific	**AT3G43600**	8.53e-06		3.18151e-06	2.28927e-05
nfu2-1 specific	**AT4G15545**	5.35e-05		1.81519e-05	0.000157463
nfu2-1 specific	**AT4G20760**	3,00E-05		7.50721e-06	0.00011982
nfu2-1 specific	**AT4G33350**	6.87e-05		2.48729e-05	0.000189563
nfu2-1 specific	**AT4G34620**	0.000419		0.000121326	0.00144747
nfu2-1 specific	**AT4G34730**	4.63e-05		1.76844e-05	0.00012132
nfu2-1 specific	**AT4G35250**	0.00011		2.40055e-05	0.000499951
nfu2-1 specific	**AT4G35770**	1.38e-05		5.06321e-06	3.784e-05
nfu2-1 specific	**AT4G38225**	3.12e-05		8.4805e-06	0.000114585
nfu2-1 specific	**AT4G38970**	0.00192		0.000280715	0.013163
nfu2-1 specific	**AT5G02120**	6.14e-05		2.24964e-05	0.000167529
nfu2-1 specific	**AT5G04830**	7.71e-05		2.73457e-05	0.000217452
nfu2-1 specific	**AT5G08610**	1.02e-05		2.40431e-06	4.33974e-05
nfu2-1 specific	**AT5G12860**	4.51e-05		1.89416e-05	0.000107352

nfu2-1 specific	**AT5G13280**	9.92e-06		3.97557e-06	2.47435e-05
nfu2-1 specific	**AT5G13510**	0.000399		9.71979e-05	0.00164117
nfu2-1 specific	**AT5G13650**	0.000124		2.98432e-05	0.00051333
nfu2-1 specific	**AT5G14660**	5.95e-05		1.32955e-05	0.000266466
nfu2-1 specific	**AT5G15450**	0.000164		5.10286e-05	0.000527186
nfu2-1 specific	**AT5G19760**	0.000476		0.000129109	0.00175264
nfu2-1 specific	**AT5G20720**	0.00145		0.000419265	0.00501601
nfu2-1 specific	**AT5G21274**	0.000243		3.29789e-05	0.00179303
nfu2-1 specific	**AT5G26280**	0.000159		1.43616e-05	0.0017622
nfu2-1 specific	**AT5G30510**	0.000295		6.82347e-05	0.00127688
nfu2-1 specific	**AT5G36790**	0.000403		5.59185e-05	0.00290317
nfu2-1 specific	**AT5G37850**	0.000106		3.81298e-05	0.000295667
nfu2-1 specific	**AT5G42960**	8.9e-05		3.16141e-05	0.000250684
nfu2-1 specific	**AT5G45390**	0.000616		0.000208426	0.0018214
nfu2-1 specific	**AT5G45680**	0.000163		4.94801e-05	0.000539935
nfu2-1 specific	**AT5G47190**	0.000433		0.000147598	0.00127172
nfu2-1 specific	**AT5G54600**	0.000174		4.38111e-05	0.000690619
nfu2-1 specific	**AT5G58770**	4.05e-05		9.10311e-06	0.000180442
nfu2-1 specific	**AT5G63310**	0.000337		9.79257e-05	0.00115925
nfu2-1 specific	**AT5G63380**	1.8e-05		4.89431e-06	6.60864e-05
nfu2-1 specific	**AT5G63860**	4.58e-05		2.0384e-05	0.000102945
nfu2-1 specific	**AT5G64840**	1.02e-05		2.49505e-06	4.13033e-05
nfu2-1 specific	**ATCG00130**	0.00142		0.00037526	0.00534288
nfu2-1 specific	**ATCG00360**	1.3e-05		2.42008e-06	6.96652e-05
nfu2-1 specific	**ATCG00640**	0.000208		5.44353e-05	0.000797842
nfu2-1 specific	**ATCG00650**	0.000716		0.000190891	0.00268398
nfu2-1 specific	**ATCG00660**	0.00018		6.41783e-05	0.000503359
nfu2-1 specific	**ATCG00780**	0.000508		0.000129702	0.00198921
nfu2-1 specific	**ATCG00790**	0.000284		7.39029e-05	0.00109304
nfu2-1 specific	**ATCG00800**	0.000669		0.000161531	0.00277397
nfu2-1 specific	**ATCG01230**	0.000133		1.74732e-05	0.00101388
nfu2-1 specific	**ATCG01240**	0.000382		9.39902e-05	0.00154941
nfu2-1 specific	**ATCG01300**	0.000122		3.1518e-05	0.000471347
nfu2-1 specific	**ATCG01310**	0.000368		0.000102058	0.00133022
nfu3-2 + *nfu2-1*	**AT1G08980**	8.05e-05	cytosol	3.19388e-05	0.000203012
nfu3-2 + *nfu2-1*	**AT1G43670**	0.000607	cytosol	0.000215005	0.00171538
nfu3-2 + *nfu2-1*	**AT1G65930**	0.00141	cytosol	0.000549296	0.00360368
nfu3-2 + *nfu2-1*	**AT2G37760**	0.000124	cytosol	4.62841e-05	0.000333242
nfu3-2 + *nfu2-1*	**AT3G47340**	3.03e-05	cytosol	5.69494e-06	0.000161013
nfu3-2 + *nfu2-1*	**AT4G10120**	1.66e-05	cytosol	2.76756e-06	9.96034e-05
nfu3-2 + *nfu2-1*	**AT4G33150**	1.62e-05	cytosol	3.32155e-06	7.86498e-05
nfu3-2 + *nfu2-1*	**AT5G28050**	0.000118	cytosol	4.95874e-05	0.000280634
nfu3-2 + *nfu2-1*	**AT5G41790**	3.82e-05	cytosol	8.0085e-06	0.000182364
nfu3-2 + *nfu2-1*	**AT5G48180**	6.62e-05	cytosol	2.88688e-05	0.000151959
nfu3-2 + *nfu2-1*	**AT5G51970**	0.000148	cytosol	6.18722e-05	0.000352754
nfu3-2 + *nfu2-1*	**AT1G02816**	0.000196	extracellular	5.8232e-05	0.000659324
nfu3-2 + *nfu2-1*	**AT1G17100**	0.000393	extracellular	0.000133028	0.00116322
nfu3-2 + *nfu2-1*	**AT1G21680**	8.19e-05	extracellular	3.69097e-05	0.000181869
nfu3-2 + *nfu2-1*	**AT1G47128**	0.000371	extracellular	9.43656e-05	0.00145885

nfu3-2 + nfu2-1	AT3G07470	0.000192	extracellular	8.48765e-05	0.000432539
nfu3-2 + nfu2-1	AT3G22060	4.59e-05	extracellular	1.6015e-05	0.000131516
nfu3-2 + nfu2-1	AT4G30270	0.000103	extracellular	4.22547e-05	0.000250007
nfu3-2 + nfu2-1	AT5G49360	0.000111	extracellular	2.04617e-05	0.000597879
nfu3-2 + nfu2-1	AT5G56870	2.18e-05	extracellular	6.23549e-06	7.61571e-05
nfu3-2 + nfu2-1	AT5G64260	0.000127	extracellular	5.84352e-05	0.000274485
nfu3-2 + nfu2-1	AT1G32470	0.000611	mitochondrion	0.000155553	0.00240095
nfu3-2 + nfu2-1	AT1G48030	0.000511	mitochondrion	0.000215102	0.00121556
nfu3-2 + nfu2-1	AT2G14170	9.47e-05	mitochondrion	2.9005e-05	0.00030916
nfu3-2 + nfu2-1	AT2G26080	0.000235	mitochondrion	6.88532e-05	0.00080125
nfu3-2 + nfu2-1	AT2G35370	0.000711	mitochondrion	0.000199669	0.00252946
nfu3-2 + nfu2-1	AT3G06050	0.000408	mitochondrion	0.000184553	0.000900397
nfu3-2 + nfu2-1	AT3G45300	7.33e-05	mitochondrion	2.80939e-05	0.000191102
nfu3-2 + nfu2-1	AT4G08870	0.00018	mitochondrion	3.46694e-05	0.000938913
nfu3-2 + nfu2-1	AT4G33010	0.000535	mitochondrion	9.87645e-05	0.00289642
nfu3-2 + nfu2-1	AT4G37930	0.000545	mitochondrion	9.61671e-05	0.00308373
nfu3-2 + nfu2-1	AT5G07440	0.000132	mitochondrion	3.21114e-05	0.000544108
nfu3-2 + nfu2-1	AT1G23310	0.00027	peroxisome	4.391e-05	0.00166141
nfu3-2 + nfu2-1	AT2G45740	5.19e-05	peroxisome	1.5441e-05	0.000174775
nfu3-2 + nfu2-1	AT3G01910	0.000119	peroxisome	5.85987e-05	0.000243138
nfu3-2 + nfu2-1	AT3G14415	0.000284	peroxisome	4.56353e-05	0.00177154
nfu3-2 + nfu2-1	AT3G14420	0.000355	peroxisome	4.96352e-05	0.00253275
nfu3-2 + nfu2-1	AT3G16910	8.93e-05	peroxisome	3.9689e-05	0.000201048
nfu3-2 + nfu2-1	AT5G11520	0.000107	peroxisome	3.20691e-05	0.000354721
nfu3-2 + nfu2-1	AT5G16370	1.15e-05	peroxisome	5.29369e-06	2.48152e-05
nfu3-2 + nfu2-1	AT1G11260	0.000106	plasma membrane	5.14099e-05	0.000216567
nfu3-2 + nfu2-1	AT3G01290	0.000144	plasma membrane	3.28249e-05	0.000632119
nfu3-2 + nfu2-1	AT1G06430	6.67e-05	plastid	2.01105e-05	0.000221447
nfu3-2 + nfu2-1	AT1G06690	0.000113	plastid	2.89555e-05	0.000444515
nfu3-2 + nfu2-1	AT1G07040	9.01e-05	plastid	3.65736e-05	0.000221872
nfu3-2 + nfu2-1	AT1G08550	7.5e-05	plastid	2.2347e-05	0.00025172
nfu3-2 + nfu2-1	AT1G12250	0.000137	plastid	3.77895e-05	0.000498097
nfu3-2 + nfu2-1	AT1G16720	7.45e-05	plastid	1.78107e-05	0.000311292
nfu3-2 + nfu2-1	AT1G16880	0.000533	plastid	0.000116213	0.00244555
nfu3-2 + nfu2-1	AT1G23740	0.000514	plastid	0.000129418	0.00204214
nfu3-2 + nfu2-1	AT1G32080	0.000186	plastid	5.1149e-05	0.000678783
nfu3-2 + nfu2-1	AT1G32220	0.000148	plastid	4.0018e-05	0.000543663
nfu3-2 + nfu2-1	AT1G34000	0.000186	plastid	5.92189e-05	0.000586097
nfu3-2 + nfu2-1	AT1G44575	0.00118	plastid	0.000257309	0.00543559
nfu3-2 + nfu2-1	AT1G57770	2.65e-05	plastid	6.51022e-06	0.000108238
nfu3-2 + nfu2-1	AT1G71500	0.000287	plastid	6.84702e-05	0.00120179
nfu3-2 + nfu2-1	AT1G73990	3.17e-05	plastid	8.22313e-06	0.000121888
nfu3-2 + nfu2-1	AT1G74970	0.00031	plastid	8.50721e-05	0.00112644
nfu3-2 + nfu2-1	AT1G78140	2.25e-05	plastid	8.75185e-06	5.76338e-05
nfu3-2 + nfu2-1	AT1G80380	0.000189	plastid	4.1043e-05	0.000865745
nfu3-2 + nfu2-1	AT2G17695	3.18e-05	plastid	7.19043e-06	0.00014036
nfu3-2 + nfu2-1	AT2G21330	0.00069	plastid	0.000100725	0.00473055
nfu3-2 + nfu2-1	AT2G28190	0.0014	plastid	0.000363091	0.00538377
nfu3-2 + nfu2-1	AT2G35410	0.000207	plastid	5.19321e-05	0.000827397

nfu3-2 + nfu2-1	AT2G41680	8.77e-05	plastid	2.07474e-05	0.000370309
nfu3-2 + nfu2-1	AT2G47400	0.000679	plastid	0.000123328	0.00373409
nfu3-2 + nfu2-1	AT3G04790	0.000923	plastid	0.000244356	0.00348938
nfu3-2 + nfu2-1	AT3G08740	0.000275	plastid	6.64339e-05	0.00114146
nfu3-2 + nfu2-1	AT3G12345	0.000116	plastid	2.85287e-05	0.000467882
nfu3-2 + nfu2-1	AT3G12780	0.00187	plastid	0.000465744	0.00752958
nfu3-2 + nfu2-1	AT3G23400	0.000736	plastid	0.000273143	0.00198098
nfu3-2 + nfu2-1	AT3G25770	0.000153	plastid	4.32521e-05	0.000539557
nfu3-2 + nfu2-1	AT3G26060	0.000791	plastid	0.000197778	0.0031622
nfu3-2 + nfu2-1	AT3G27850	0.000525	plastid	0.000178792	0.00154266
nfu3-2 + nfu2-1	AT3G44890	0.000496	plastid	9.80873e-05	0.00250369
nfu3-2 + nfu2-1	AT3G48420	0.000233	plastid	4.65852e-05	0.00116541
nfu3-2 + nfu2-1	AT3G52230	0.000163	plastid	6.51182e-05	0.00040702
nfu3-2 + nfu2-1	AT3G54050	0.000768	plastid	0.000183446	0.0032188
nfu3-2 + nfu2-1	AT3G55400	2.85e-05	plastid	1.09527e-05	7.40989e-05
nfu3-2 + nfu2-1	AT3G62410	0.000534	plastid	8.8481e-05	0.0032283
nfu3-2 + nfu2-1	AT3G63140	0.000556	plastid	0.000107881	0.00286989
nfu3-2 + nfu2-1	AT3G63190	0.000655	plastid	0.000227653	0.00188364
nfu3-2 + nfu2-1	AT4G02530	0.000457	plastid	9.26167e-05	0.00225767
nfu3-2 + nfu2-1	AT4G04020	0.000338	plastid	0.000106436	0.00107611
nfu3-2 + nfu2-1	AT4G17300	3.48e-05	plastid	1.07215e-05	0.000113198
nfu3-2 + nfu2-1	AT4G17560	0.000238	plastid	6.75549e-05	0.000839737
nfu3-2 + nfu2-1	AT4G18810	0.000107	plastid	3.54775e-05	0.000321971
nfu3-2 + nfu2-1	AT4G20360	0.00172	plastid	0.000468178	0.00635393
nfu3-2 + nfu2-1	AT4G21860	0.000135	plastid	4.93616e-05	0.000368933
nfu3-2 + nfu2-1	AT4G25130	0.000316	plastid	0.000110435	0.000903195
nfu3-2 + nfu2-1	AT4G25370	0.000196	plastid	5.2267e-05	0.000736088
nfu3-2 + nfu2-1	AT4G26530	0.000708	plastid	0.000146421	0.00342228
nfu3-2 + nfu2-1	AT4G28730	5.2e-05	plastid	1.62605e-05	0.000166214
nfu3-2 + nfu2-1	AT5G35970	2.2e-05	plastid	3.68252e-06	0.000131199
nfu3-2 + nfu2-1	AT5G64290	0.000113	plastid	3.88216e-05	0.000329257
nfu3-2 + nfu2-1	AT1G12240	0.000164	vacuole	6.16191e-05	0.000436407
nfu3-2 + nfu2-1	AT3G63520	6.91e-05	vacuole	2.25784e-05	0.000211294
nfu3-2 + nfu2-1	AT1G63770	0.000409		0.000103163	0.00161984
nfu3-2 + nfu2-1	AT1G68010	0.00082		0.000132387	0.00508188
nfu3-2 + nfu2-1	AT2G25450	0.00019		5.16492e-05	0.000700077
nfu3-2 + nfu2-1	AT3G12580	0.00012		2.77031e-05	0.000517346
nfu3-2 + nfu2-1	AT3G14067	0.000268		0.0001039	0.000691718
nfu3-2 + nfu2-1	AT3G19170	0.000228		4.722e-05	0.00109644
nfu3-2 + nfu2-1	AT3G28270	9.11e-05		3.14902e-05	0.000263524
nfu3-2 + nfu2-1	AT3G56290	5.24e-05		1.2526e-05	0.000219143
nfu3-2 + nfu2-1	AT3G61220	0.00018		5.72052e-05	0.000566677
nfu3-2 + nfu2-1	AT4G35090	0.000606		0.000140731	0.00261263
nfu3-2 + nfu2-1	AT5G04140	0.000743		0.000118498	0.00465431
nfu3-2 + nfu2-1	AT5G09660	0.00112		0.000175716	0.00720207
nfu3-2 + nfu2-1	AT5G14740	0.000683		6.91034e-05	0.00674908
nfu3-2 + nfu2-1	AT5G20250	3.41e-05		6.41102e-06	0.000181812
nfu3-2 + nfu2-1	AT5G35790	3.23e-05		1.13213e-05	9.22061e-05
nfu3-2 + nfu2-1	AT5G40370	0.000666		0.000217848	0.00203574

nfu3-2 + *nfu2-1*	**AT5G40950**	0.000179		4.43625e-05	0.000719381
nfu3-2 + *nfu2-1*	**AT5G49910**	0.000293		9.0129e-05	0.000951068
nfu3-2 + *nfu2-1*	**AT5G50920**	0.000548		0.000145175	0.00206754
nfu3-2 + *nfu2-1*	**ATCG00140**	0.00134		0.000348585	0.00513399
nfu3-2 + *nfu2-1*	**ATCG00750**	0.000226		6.2548e-05	0.000814872
nfu3-2 + *nfu2-1*	**ATCG00770**	0.000338		8.70392e-05	0.00131574
