# Supplementary material for: A Global Proteomic Approach Sheds New Light on Potential Iron-Sulfur Client Proteins of the Chloroplastic Maturation Factor NFU3
Source: Int J Mol Sci. 2020 Oct 30;21(21):8121. doi: 10.3390/ijms21218121 (PMC7672563; doi:10.3390/ijms21218121)
Supplement: Supplementary file 1 [file ijms-21-08121-s001.zip › ijms-973833 final suppl/Berger_et_al_IJMS_Table_S6_vIII.pdf]

Table S6. List of primers used in this study

Name	Sequence	Use
AttB1pNFU1	GGGGACAAGTTTGTACAAAAAAGCAGGCTCGCAGTACCCTAAACCATTG	cloning in pGWB4 and pGWB3
AttB2NFU1-STOP	GGGGACCACTTTGTACAAGAAAGCTGGCTAGCTTGTAAAGGTTAC	cloning in pGWB4 and pGWB3
AttB1pNFU2	GGGGACAAAAGTTTGTACAAAAAAGCAGGCTGTGTGTTGGGCCGACTAGAA	cloning in pGWB4 and pGWB3
AttB2NFU2-STOP	GGGGACCACTTTGTACAAGAAAGCTGGTATAAGTTGAACAGCTGCG	cloning in pGWB4 and pGWB3
AttB1pNFU3	GGGGACAAGTTTGTACAAAAAAGCAGGCTGCTCTGGTGGTTGCAGTAGT	cloning in pGWB4 and pGWB3
AttB2NFU3-STOP	GGGGACCACTTTGTACAAGAAAGCTGGCTCTAGAAGCTGGACTGCACC	cloning in pGWB4 and pGWB3
QNFU1-F	GTGGAGGCAGTGAATGCTCA	qRT-PCR
QNFU1-R	CAATCGACTCCGGTCCAACA	qRT-PCR
QNFU2F	TGTAGCTAGGCCTTGTAATCCAC	qRT-PCR
QNFU2R	GGTCTGGGGTTGCTACTGC	qRT-PCR
QNFU3-F	TGAGTTAAGGCCGTACCTATC	qRT-PCR
QNFU3-R	GCTGGACTGCACCTATAGAAG	qRT-PCR
Q1_DWARF27.2	TGGACACTTGTAAGTGGTAAGGT	qRT-PCR
Q2_DWARF27.2	TCGGCTCAGCAATTCCACAT	qRT-PCR
Q1_GLT1	AGTTGGGAGAAGGATGAAACCGGGAGG	qRT-PCR
Q2_GLT1	GTGATAGTGTTGTGTTTCATCTGGTTAAGG	qRT-PCR
Q1_NIR1	AGCGATTCCTCTTGATGC	qRT-PCR
Q2_NIR1	GTTCGTCGATAAGCCACA	qRT-PCR
Q1_petC	TATGTGGAAGCAGAGGCTGG	qRT-PCR
Q2_petC	GCTGAGTAGCAGGGGAAAGG	qRT-PCR
Q1_FdC1	ccgtaacgcaacactctcaa	qRT-PCR
Q2_FdC1	acgagtttcgctggacaagt	qRT-PCR
Q1_FdC2	tccttcgcacaaagtcactg	qRT-PCR
Q2_FdC2	tgcctcagctctccagattt	qRT-PCR
Q1_HCAR	CGGTACTCGTTGGACTACCAT	qRT-PCR
Q2_HCAR	CTATTTGGCCGTTTTTTGTTGT	qRT-PCR
Q1_THIC	TAGCTTACCACAAGGAGAA	qRT-PCR
Q2_THIC	CCAATGGAAAGAGCCACATCA	qRT-PCR
Q1_NFU3	TGAGTTAAGGCCGTACCTATC	qRT-PCR
Q2_NFU3	GCTGGACTGCACCTATAGAAG	qRT-PCR
